# Supplementary material for: Enantioselective desymmetrization of cyclohexadienones via an intramolecular Rauhut–Currier reaction of allenoates
Source: Nat Commun. 2016 Oct 4;7:13024. doi: 10.1038/ncomms13024 (PMC5059449; doi:10.1038/ncomms13024)
Supplement: Supplementary Information — Supplementary Figures 1-82, Supplementary Tables 1-3, Supplementary Note 1, Supplementary Methods and Supplementary References [file ncomms13024-s1.pdf]

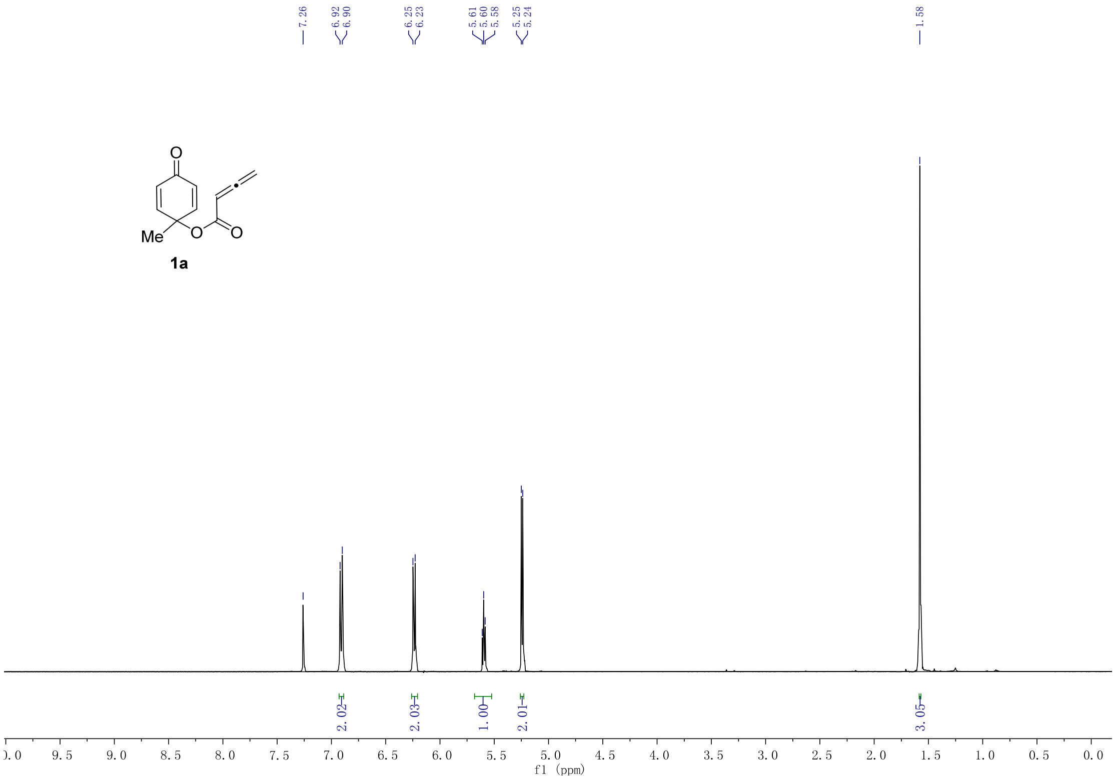

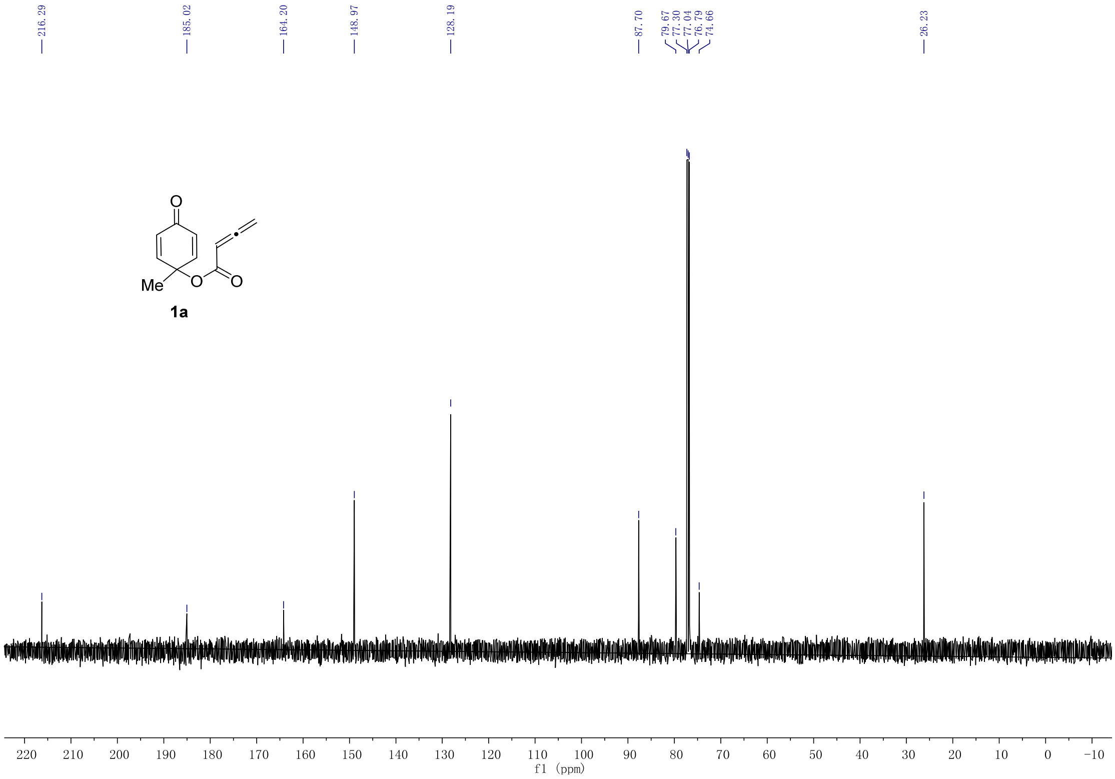

Supplementary Figure 1. ^{1}H and ^{13}C NMR spectra for compound **1a**

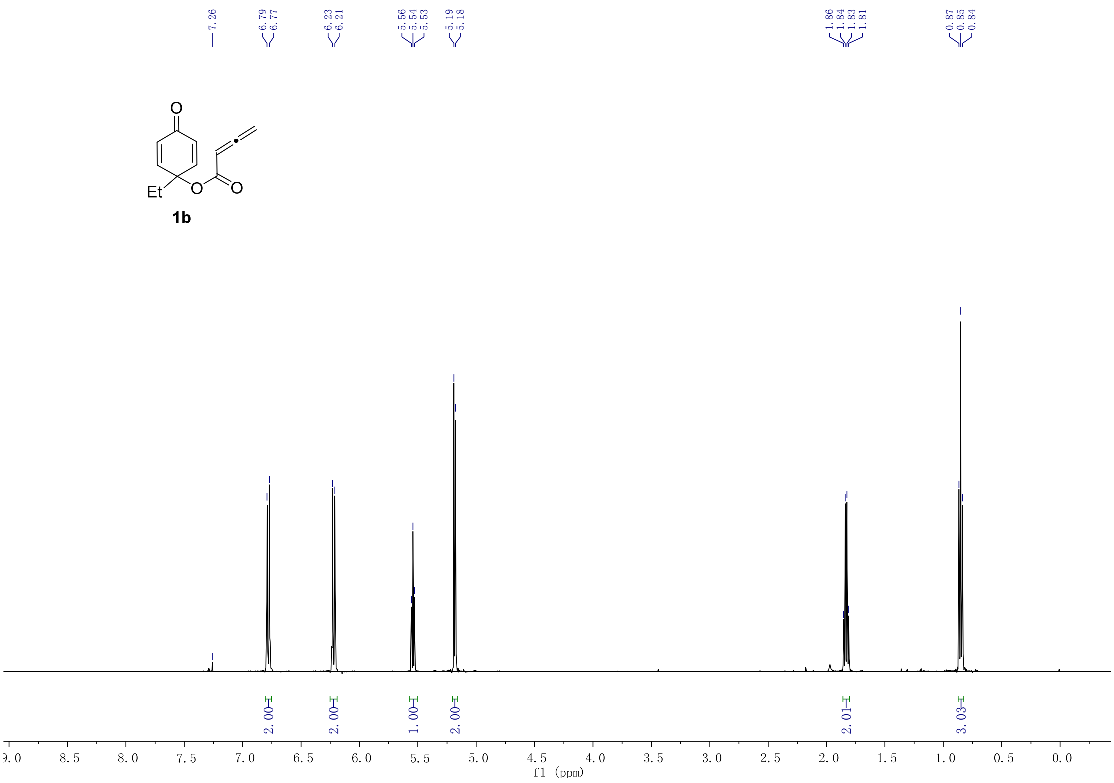

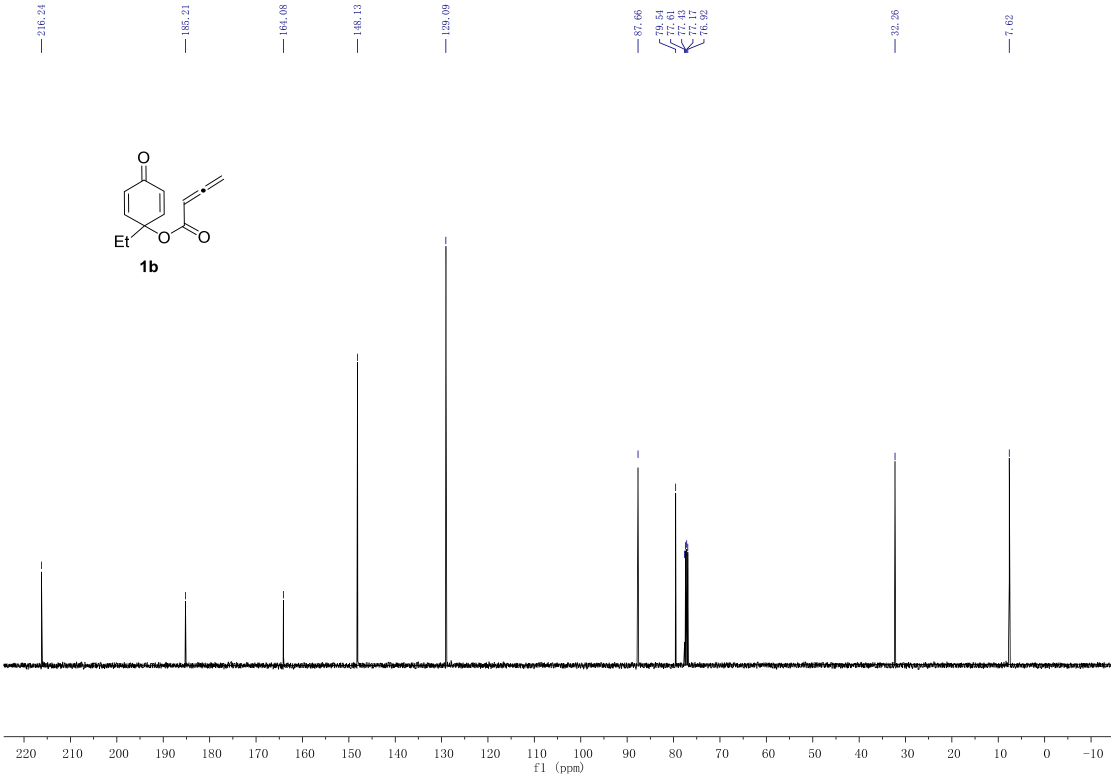

Supplementary Figure 2. ^{1}H and ^{13}C NMR spectra for compound **1b**

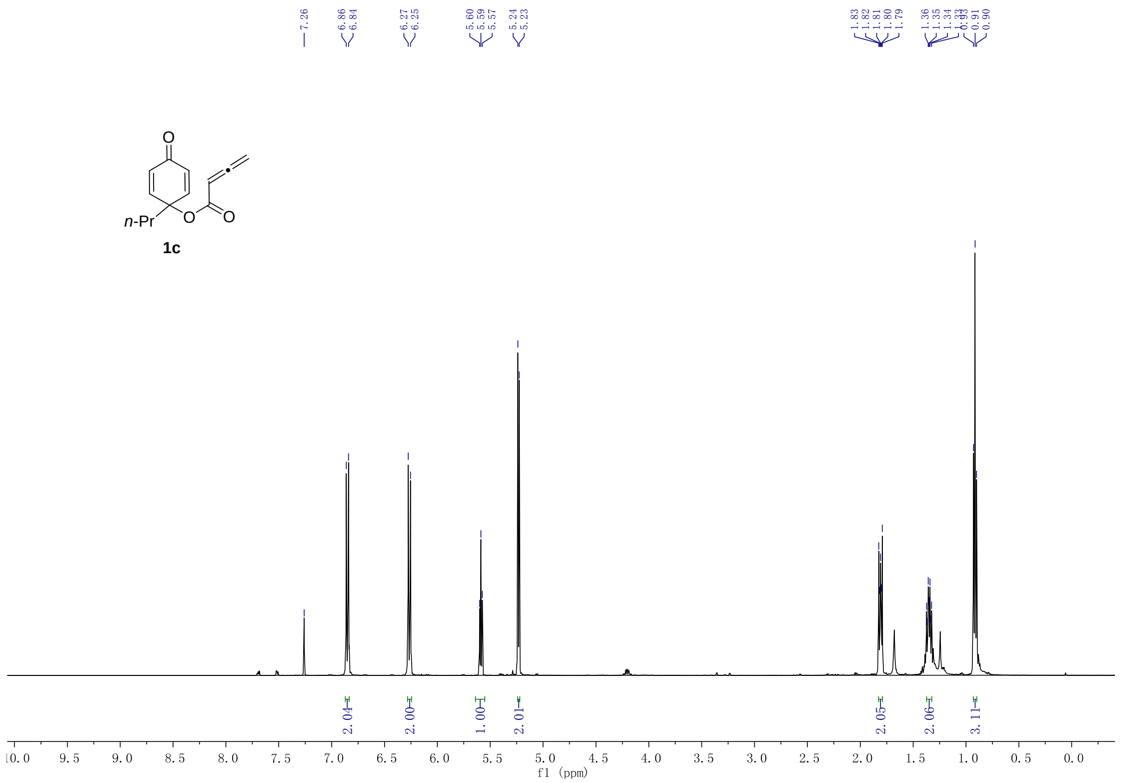

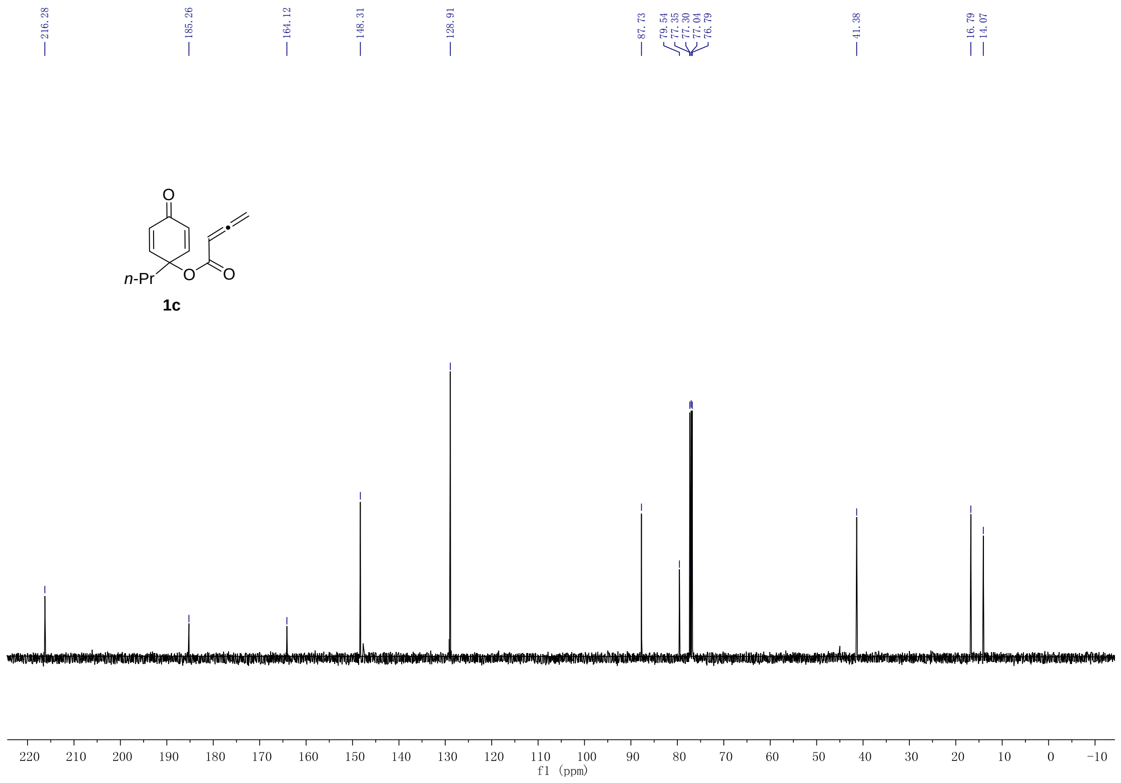

Supplementary Figure 3. ^{1}H and ^{13}C NMR spectra for compound **1c**

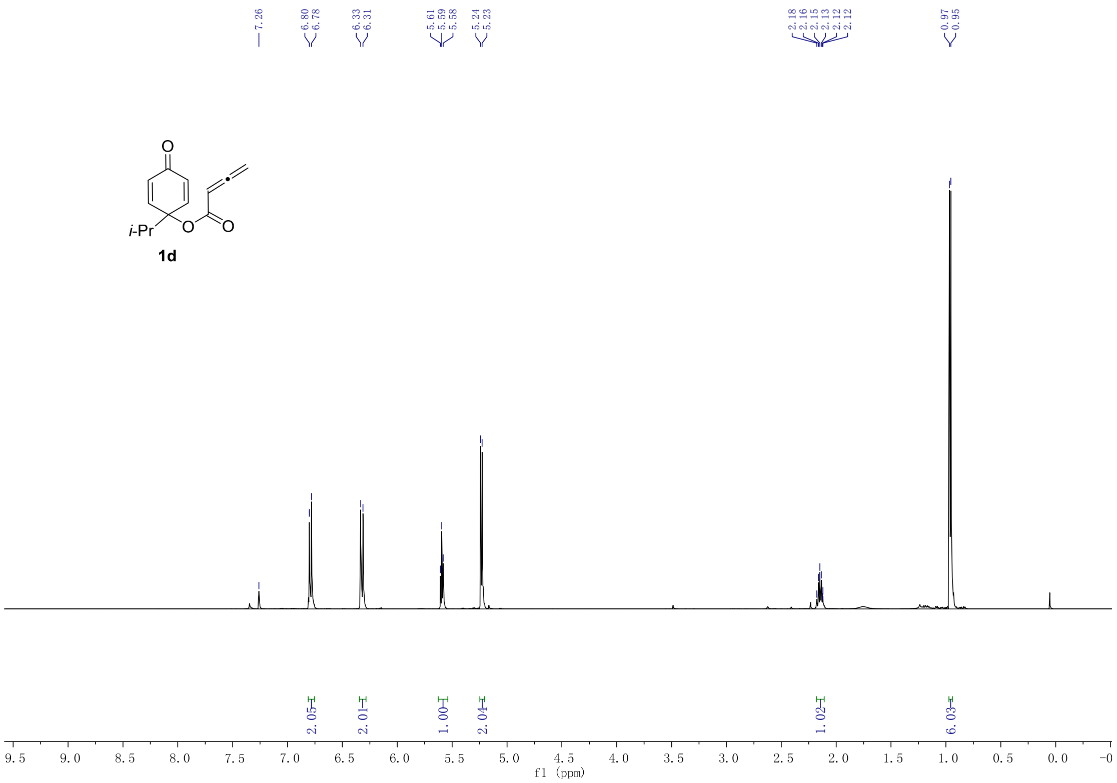

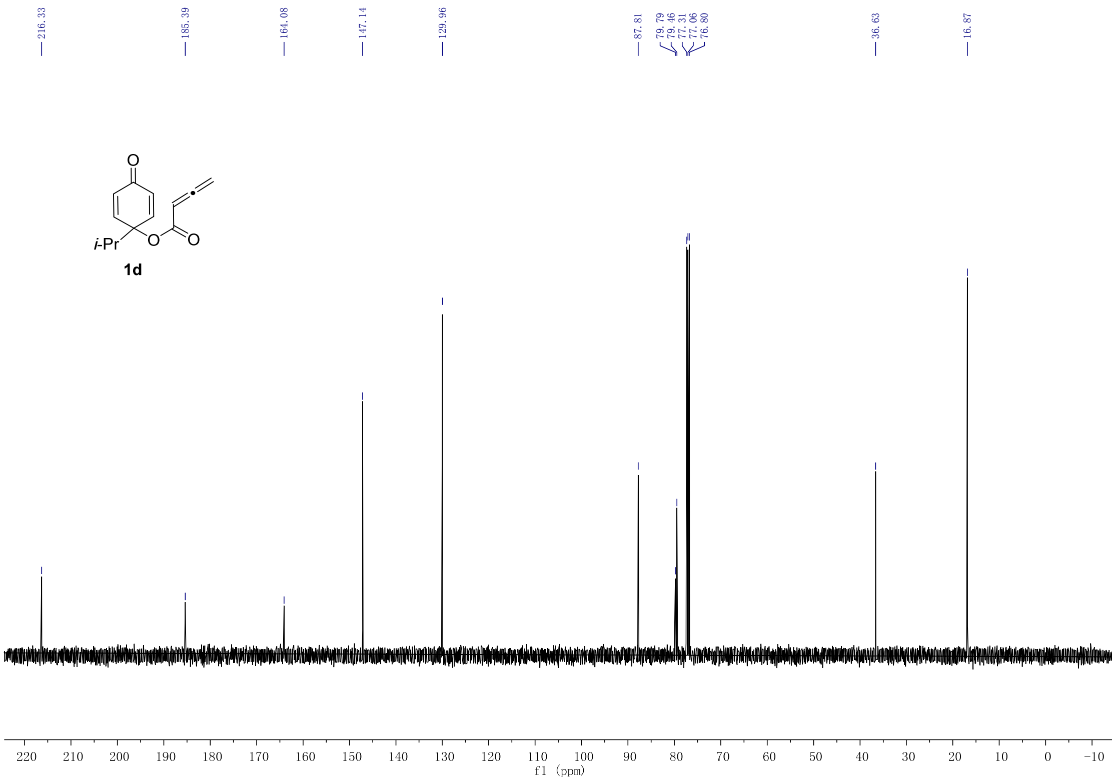

Supplementary Figure 4. ^{1}H and ^{13}C NMR spectra for compound **1d**

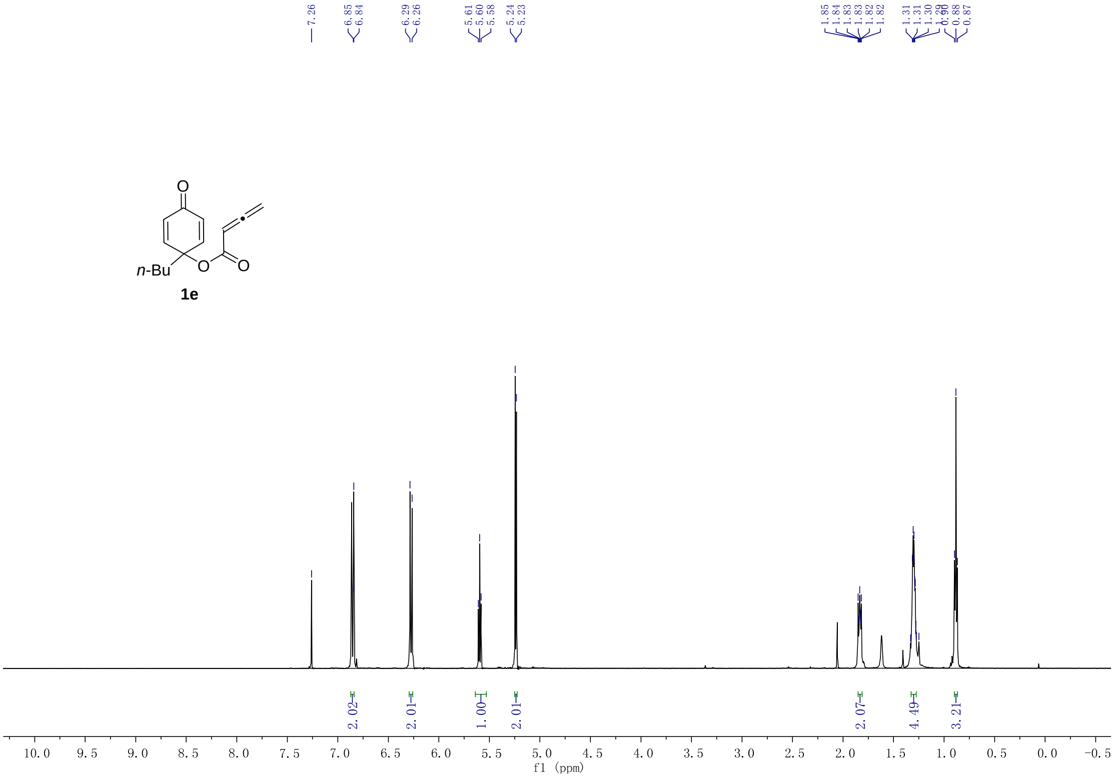

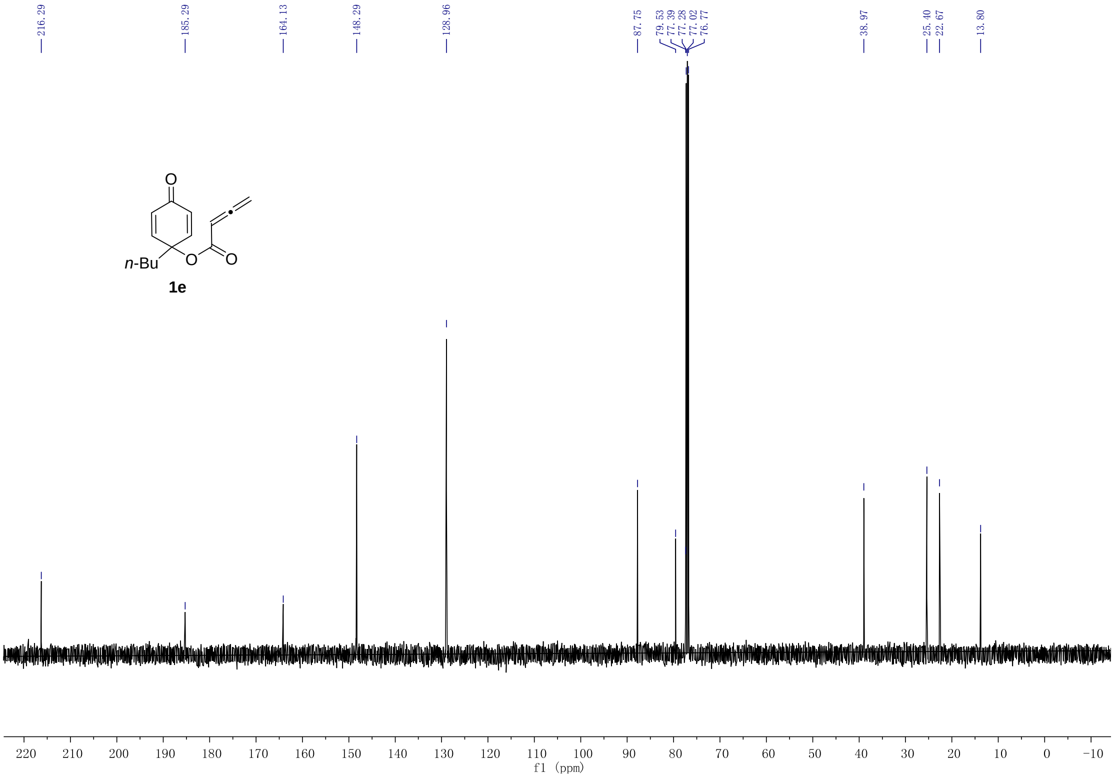

Supplementary Figure 5. ^{1}H and ^{13}C NMR spectra for compound **1e**

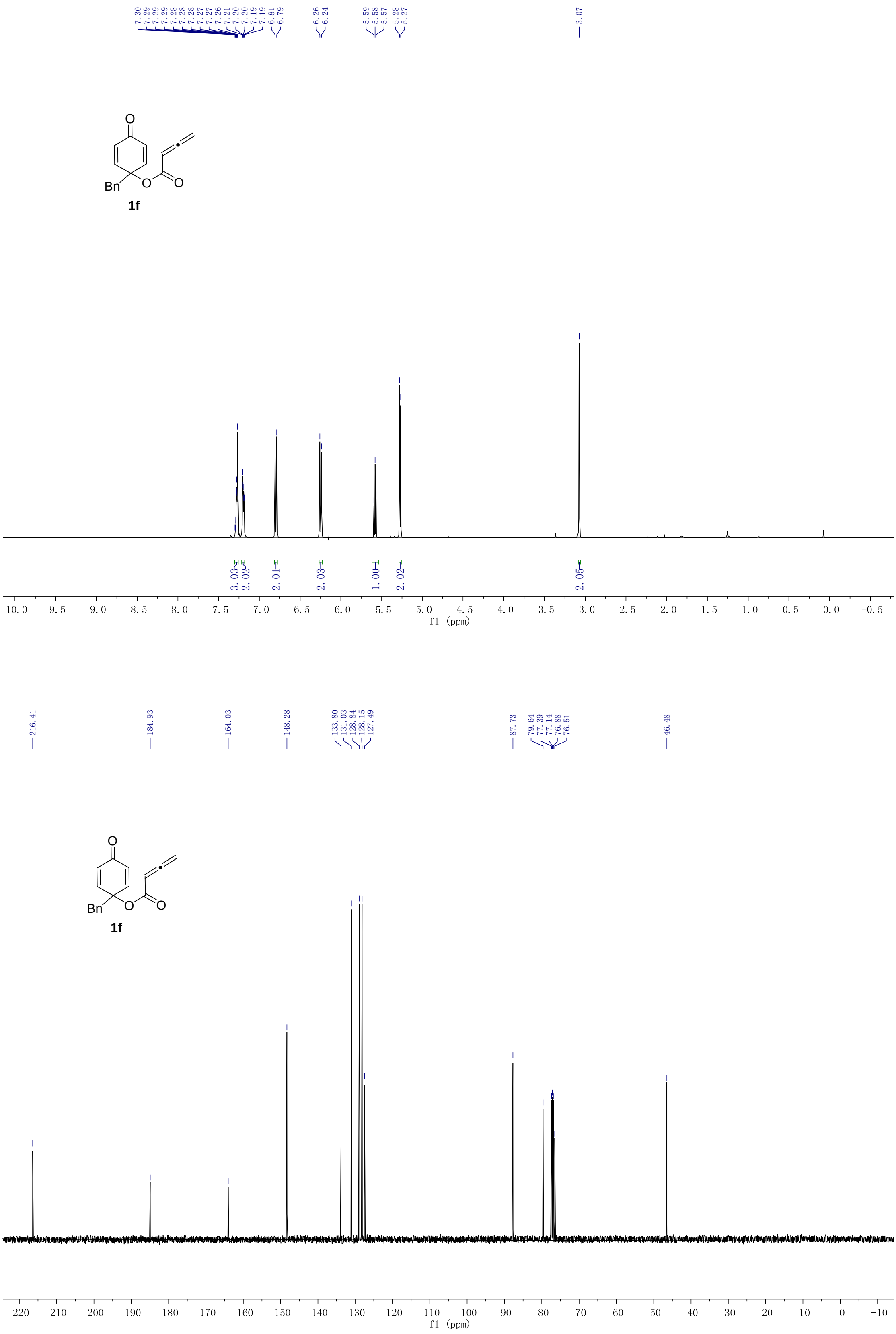

Supplementary Figure 6. ^{1}H and ^{13}C NMR spectra for compound **1f**

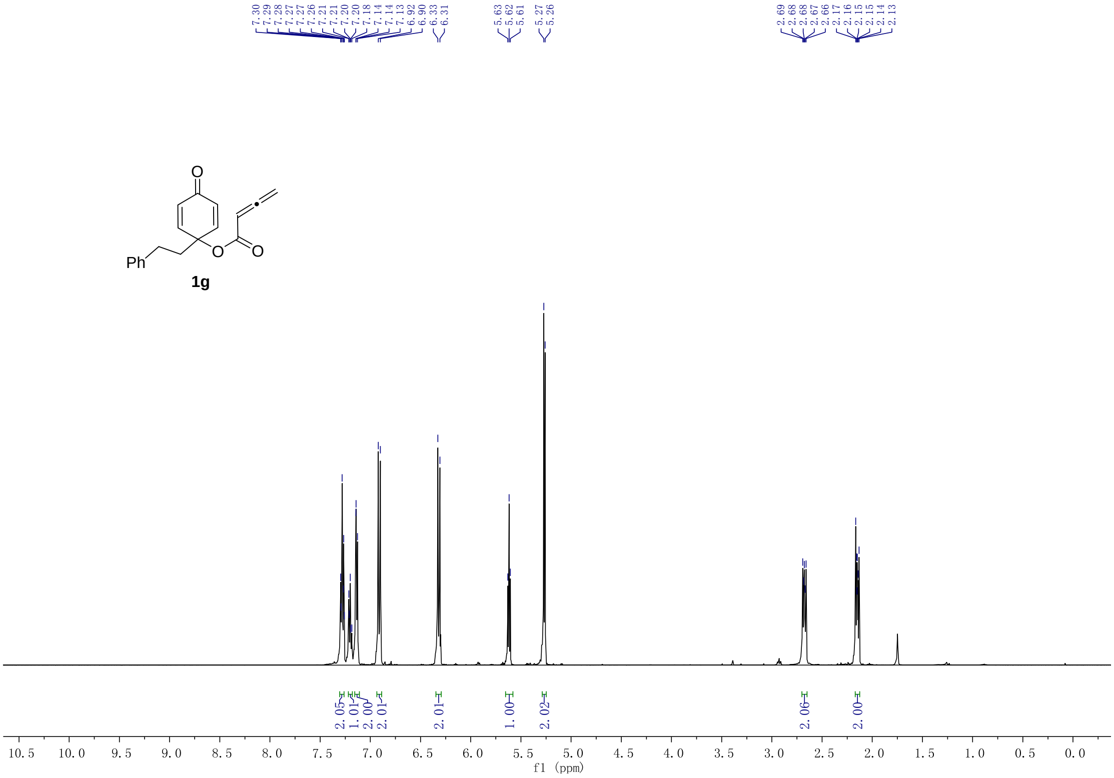

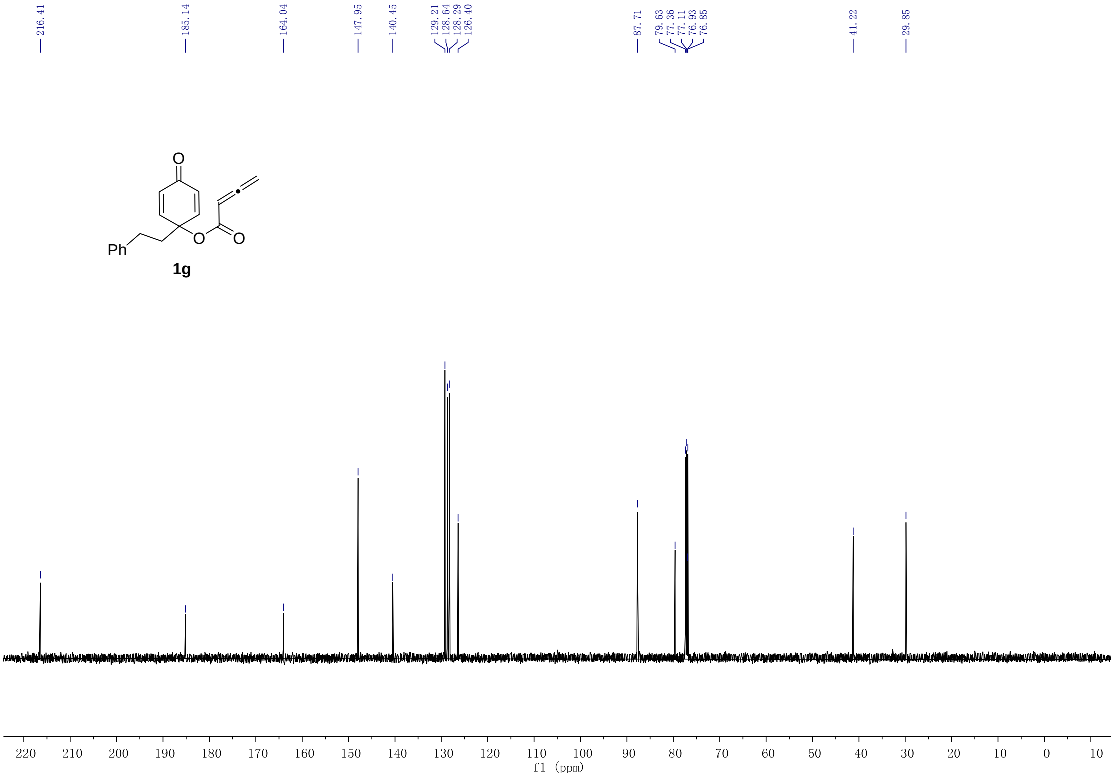

Supplementary Figure 7. ^{1}H and ^{13}C NMR spectra for compound **1g**

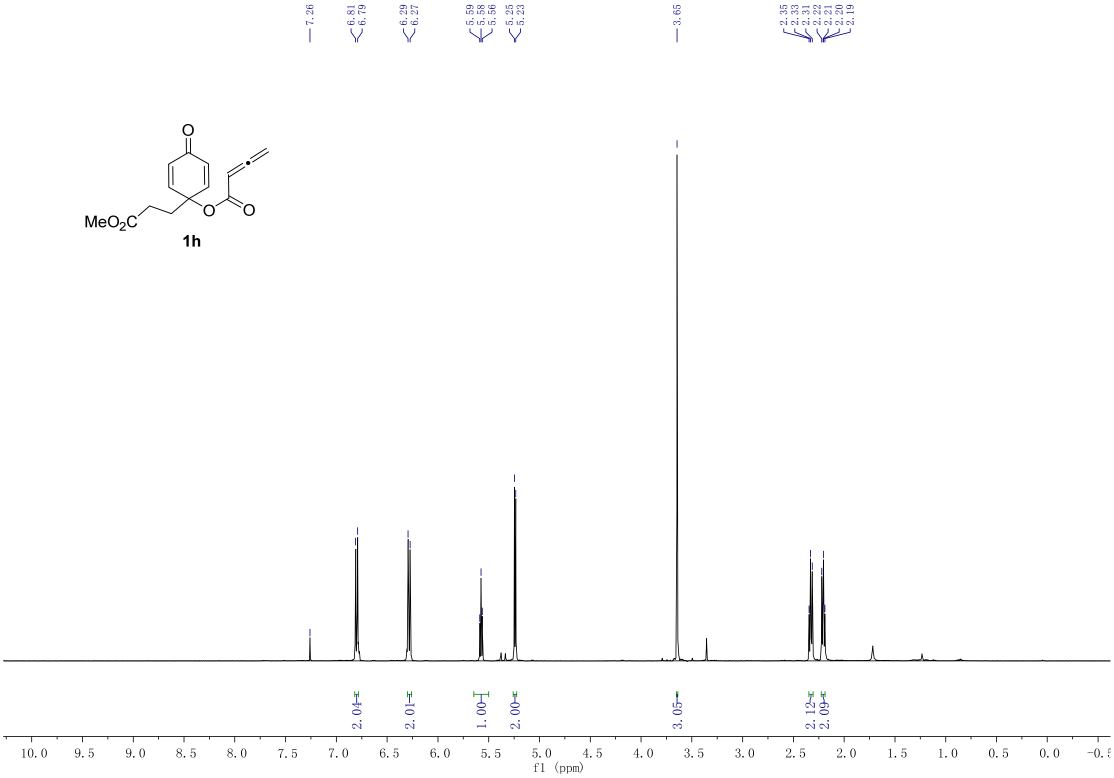

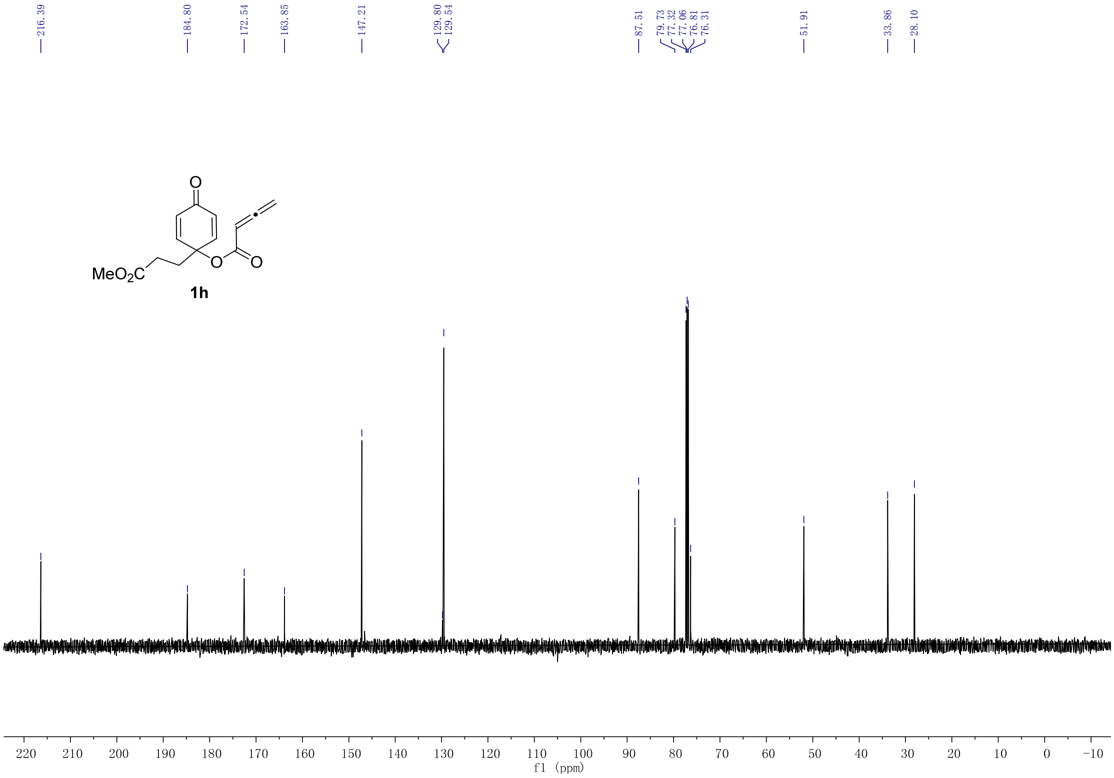

Supplementary Figure 8. ^{1}H and ^{13}C NMR spectra for compound **1h**

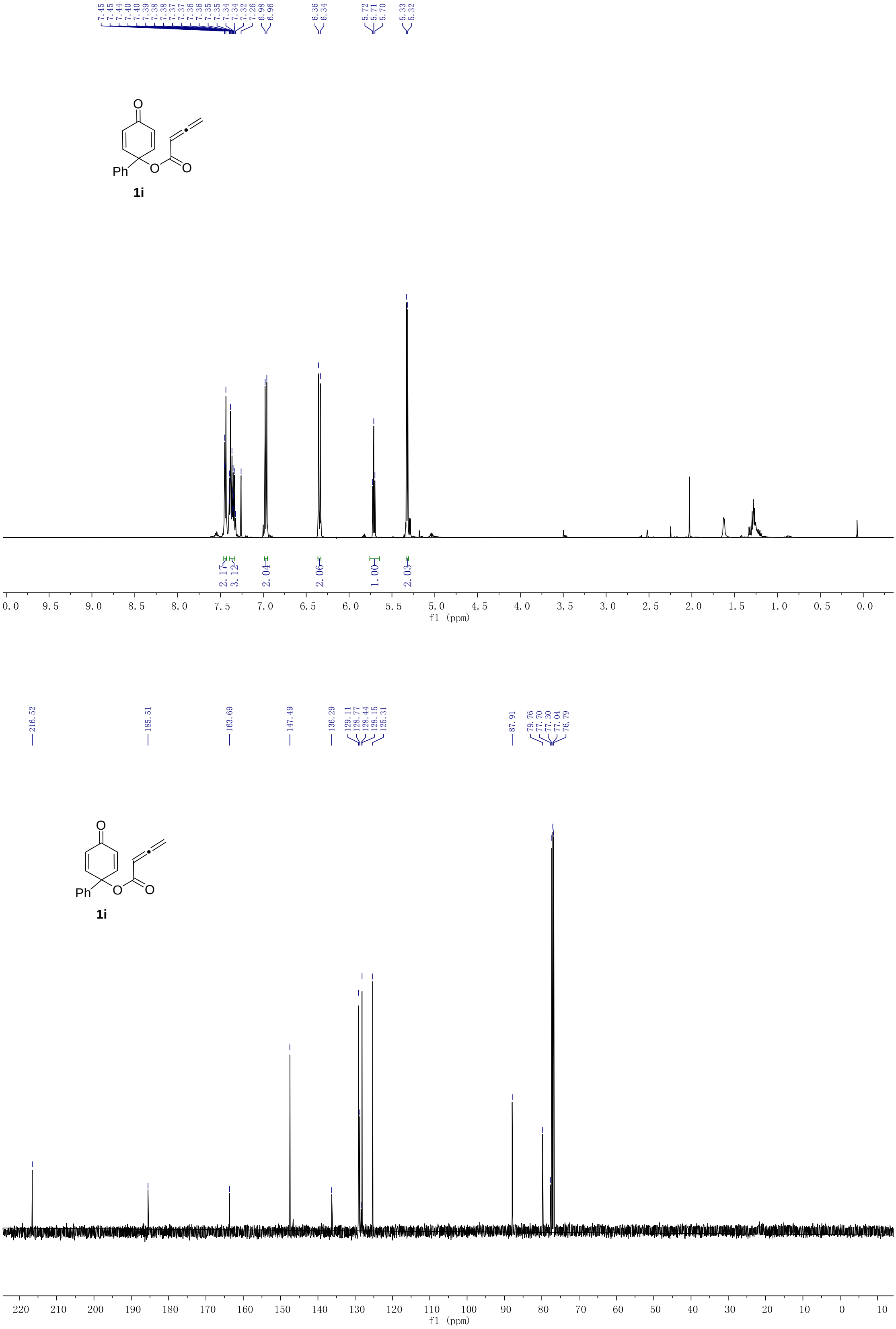

Supplementary Figure 9. ^{1}H and ^{13}C NMR spectra for compound **1i**

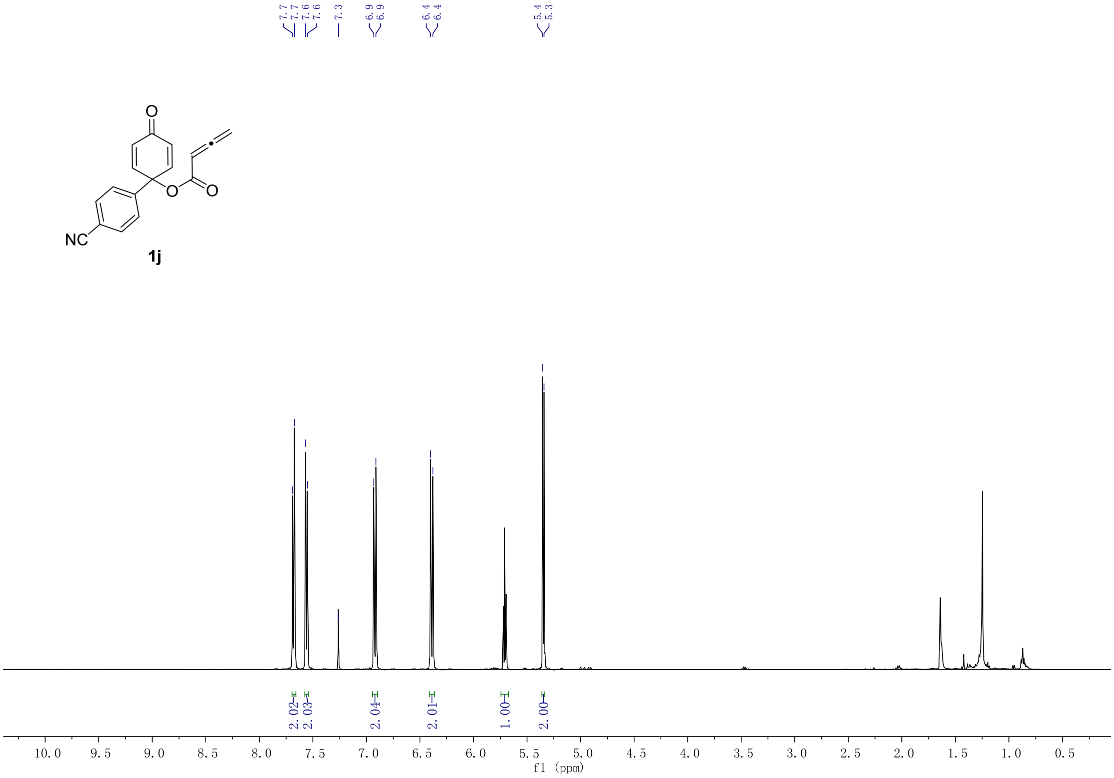

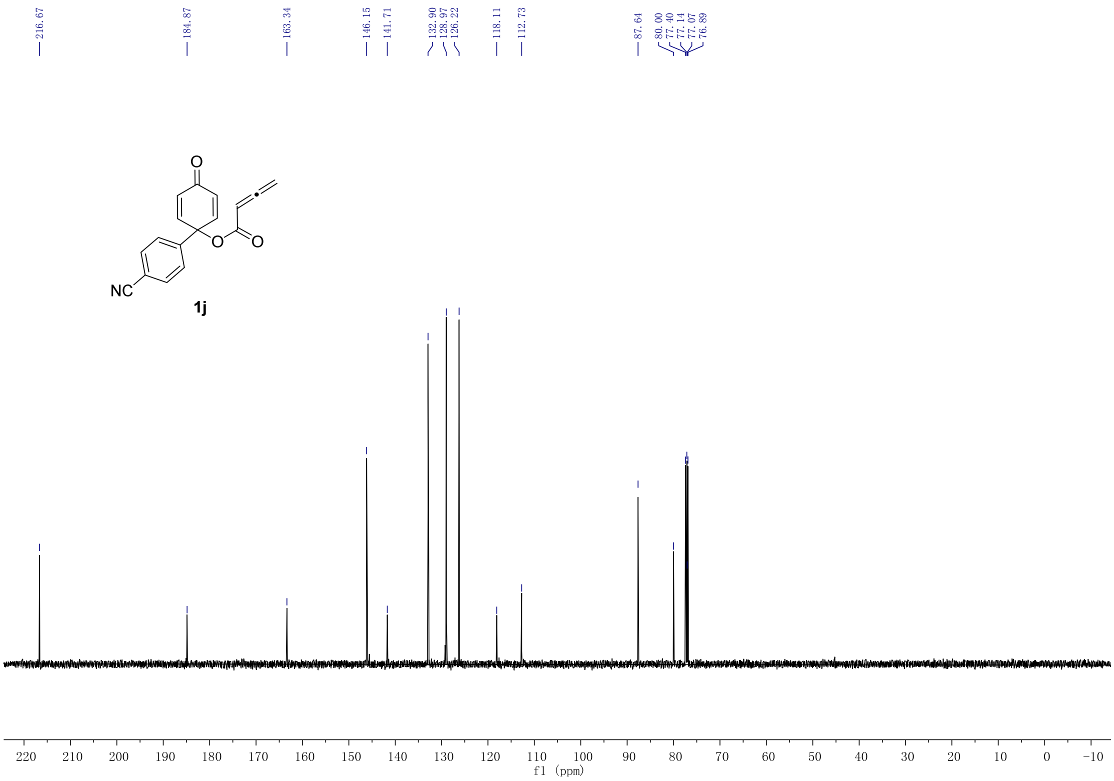

Supplementary Figure 10. ^{1}H and ^{13}C NMR spectra for compound **1j**

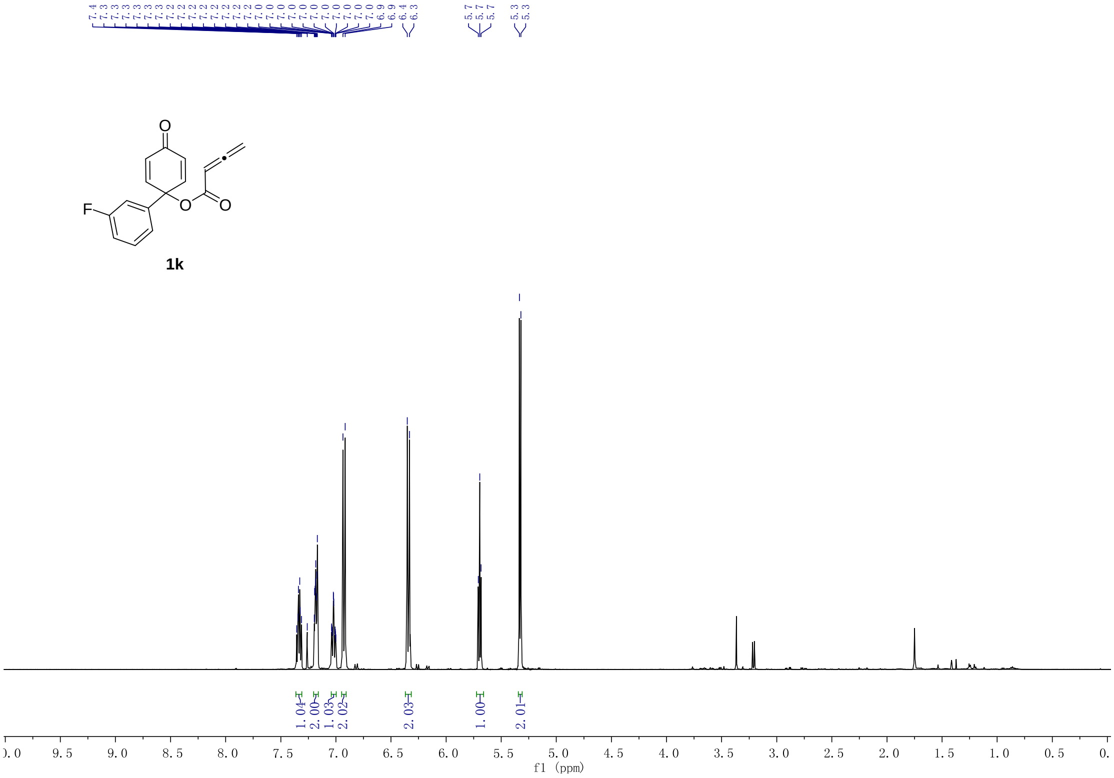

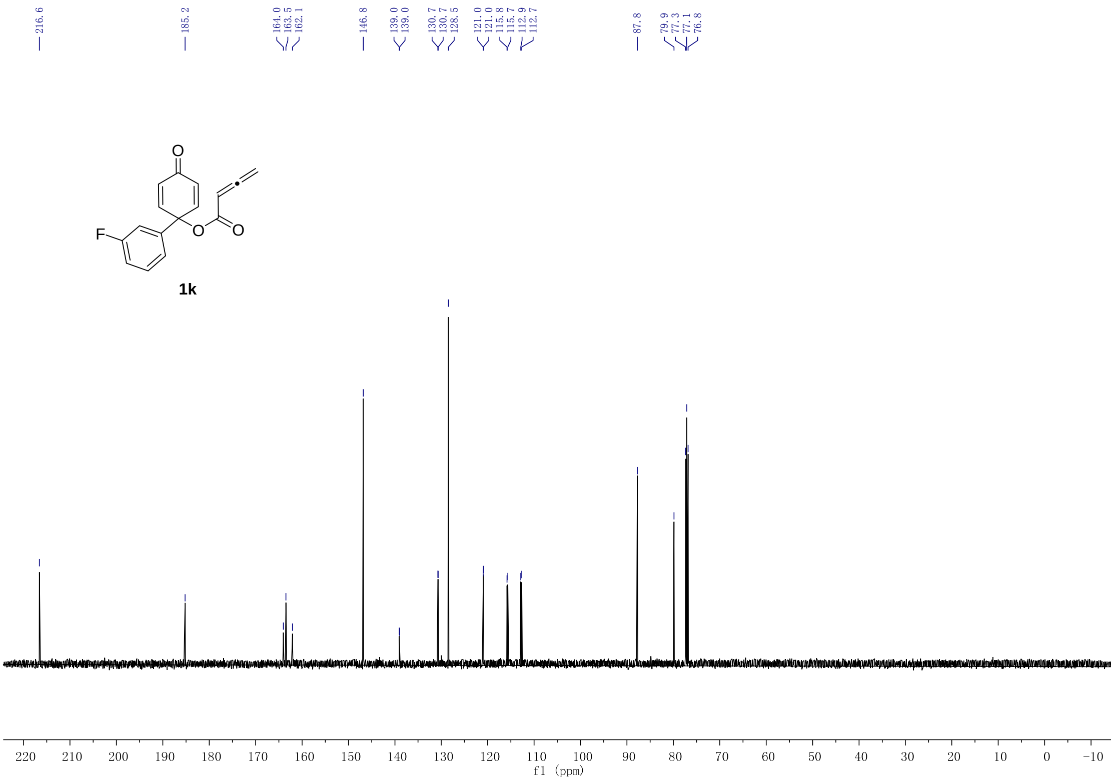

Supplementary Figure 11. ^{1}H and ^{13}C NMR spectra for compound **1k**

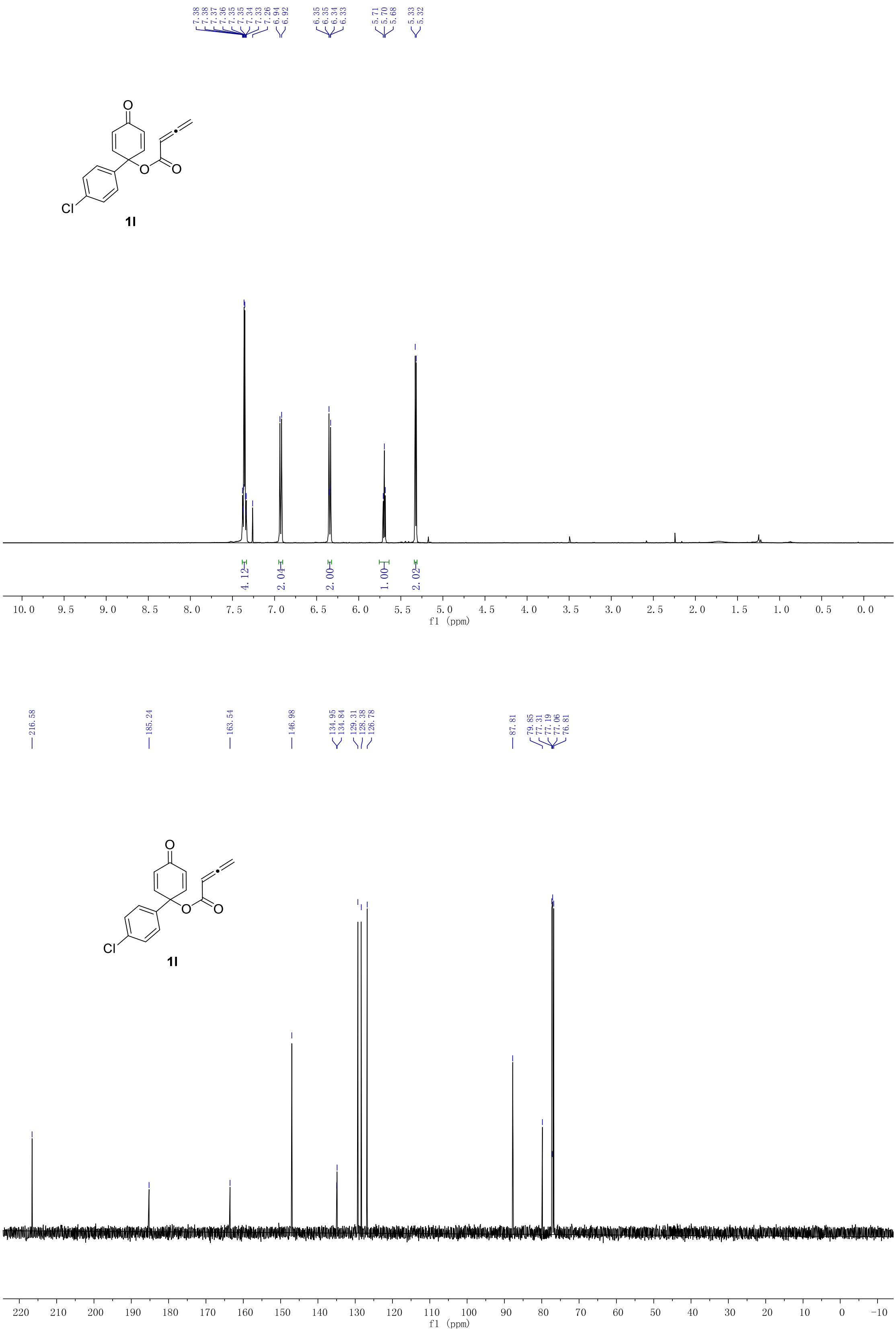

Supplementary Figure 12. ^{1}H and ^{13}C NMR spectra for compound **1l**

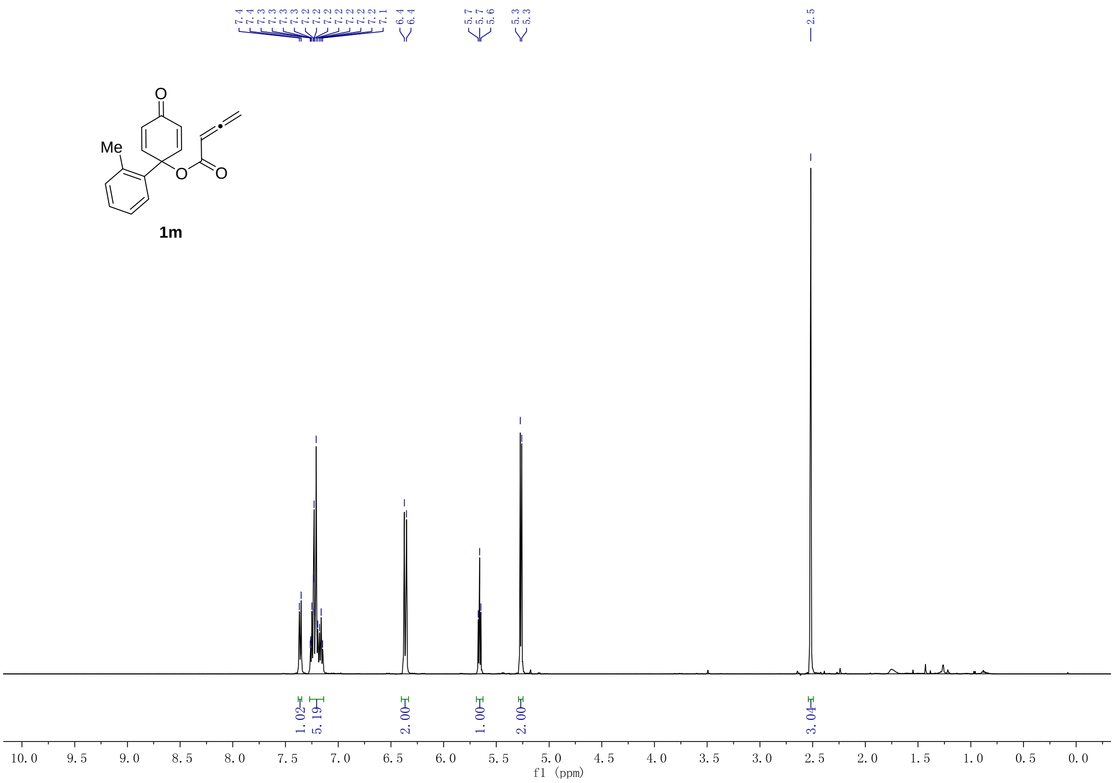

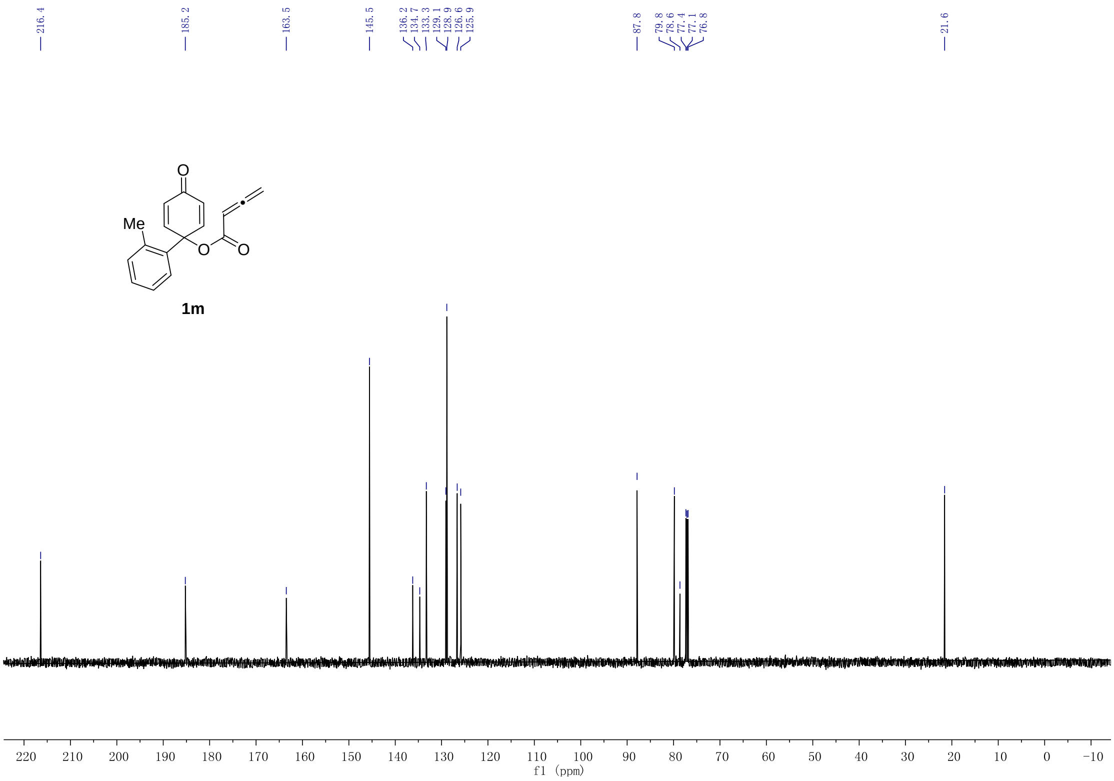

Supplementary Figure 13. ^{1}H and ^{13}C NMR spectra for compound **1m**

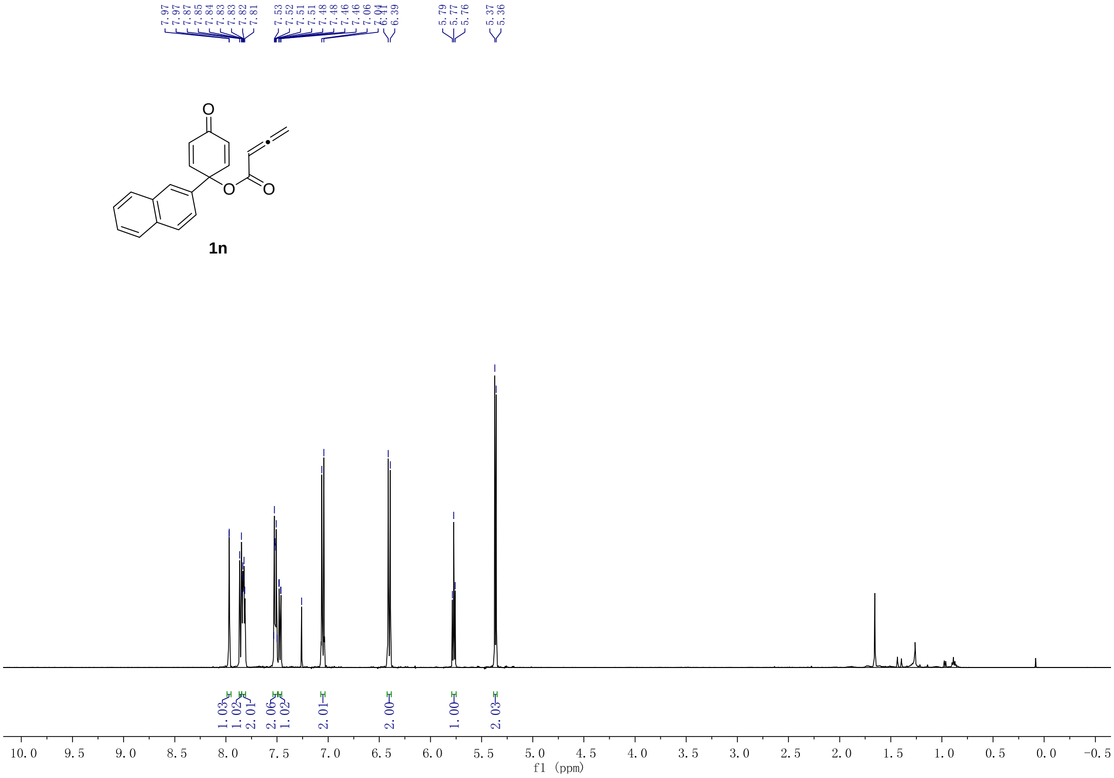

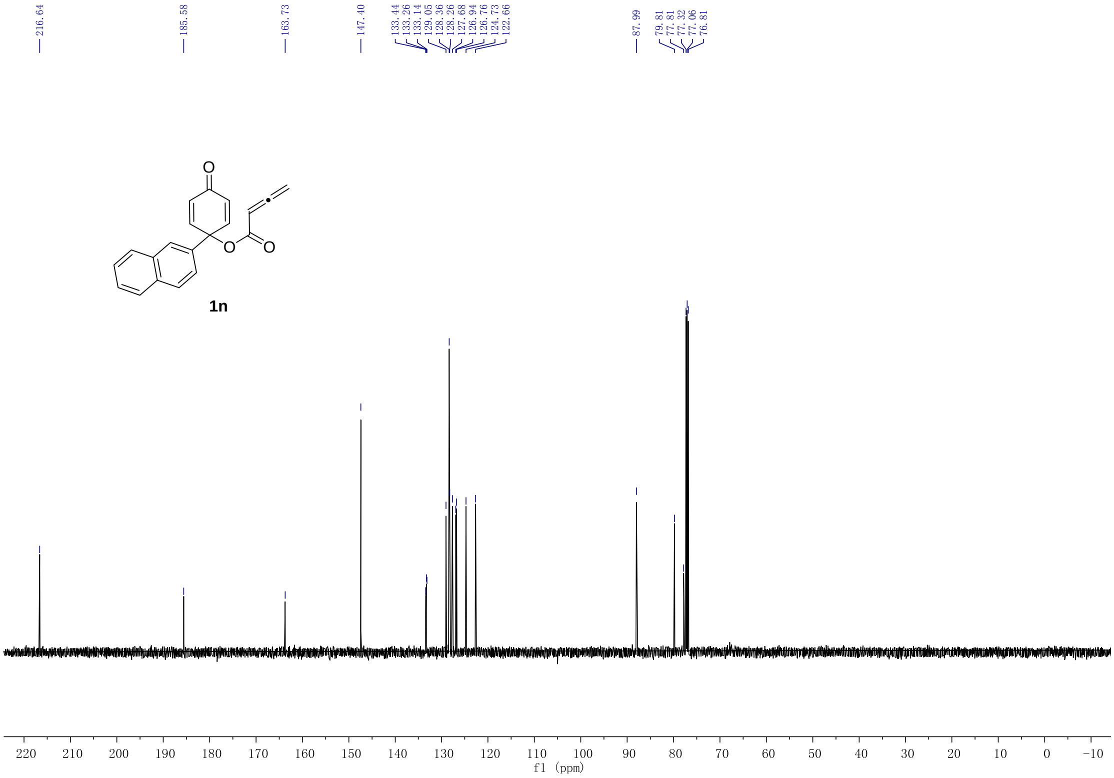

Supplementary Figure 14. ^{1}H and ^{13}C NMR spectra for compound **1n**

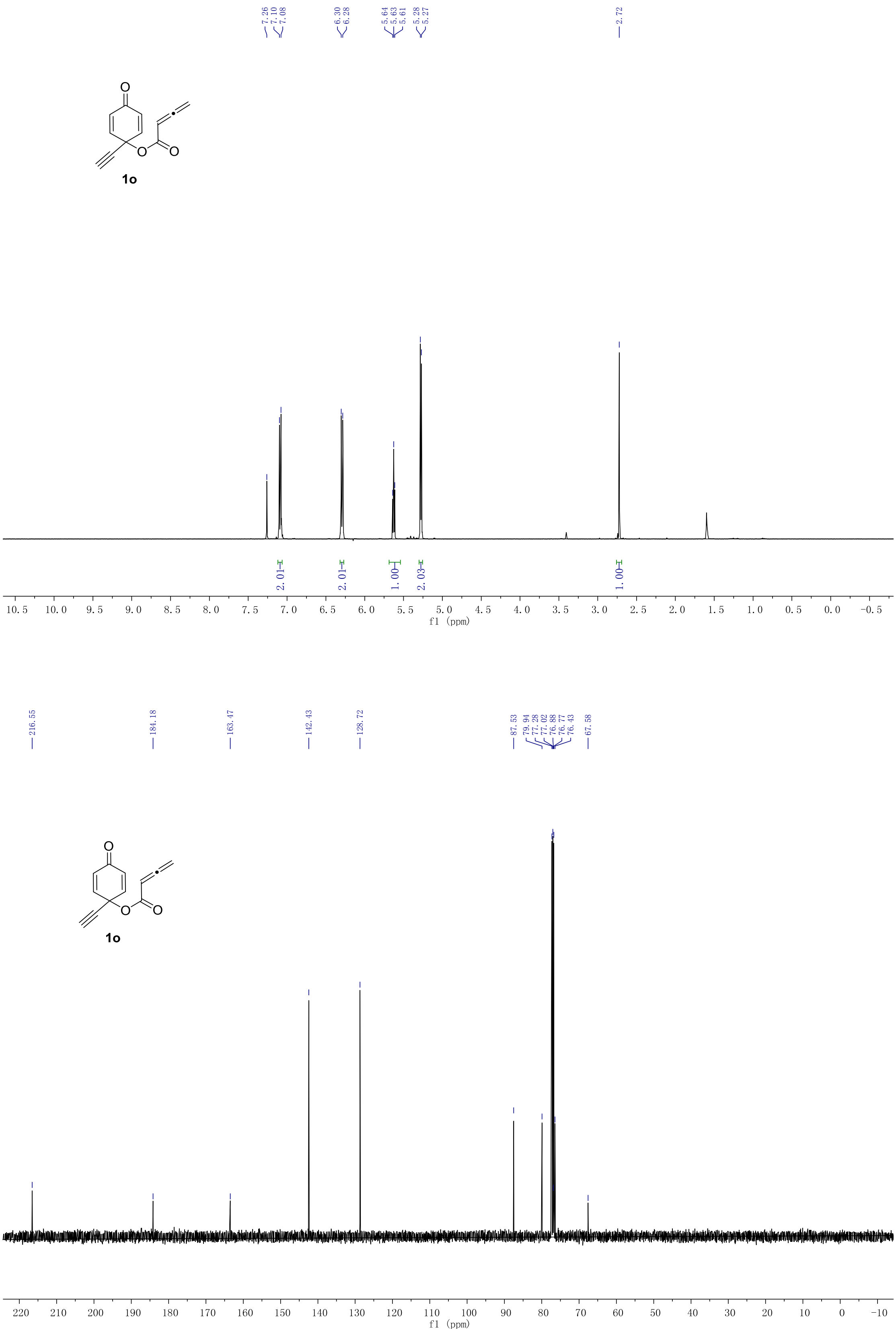

Supplementary Figure 15. ^{1}H and ^{13}C NMR spectra for compound **1o**

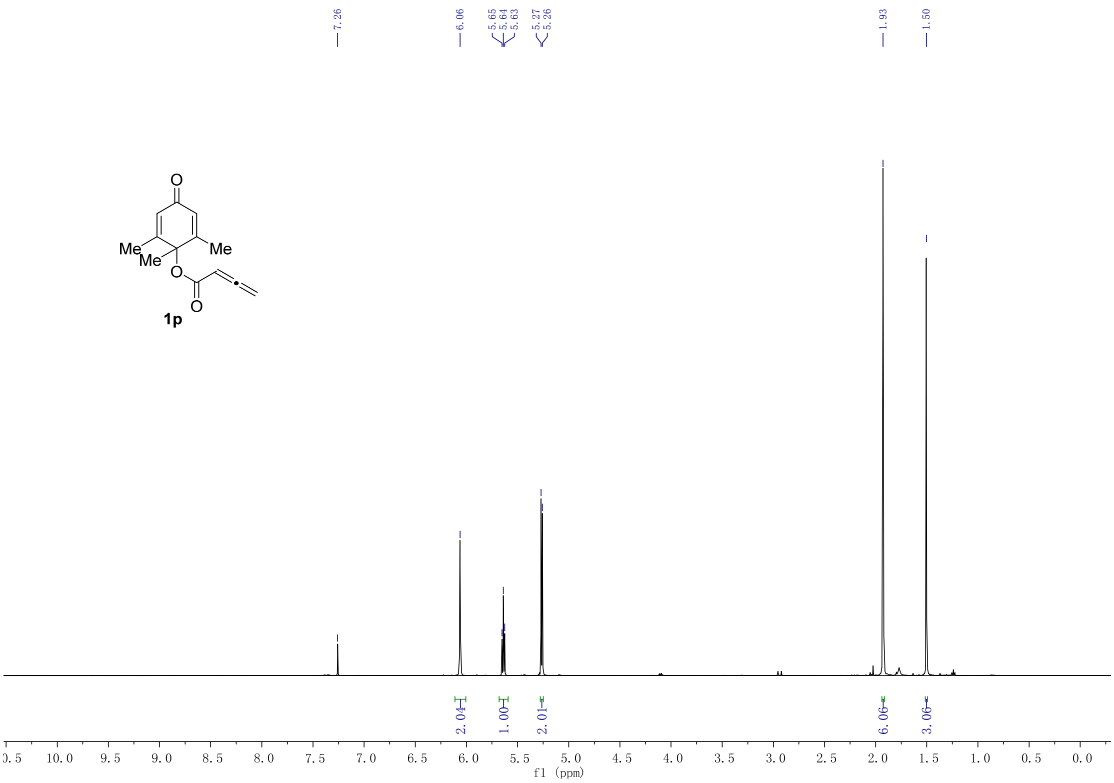

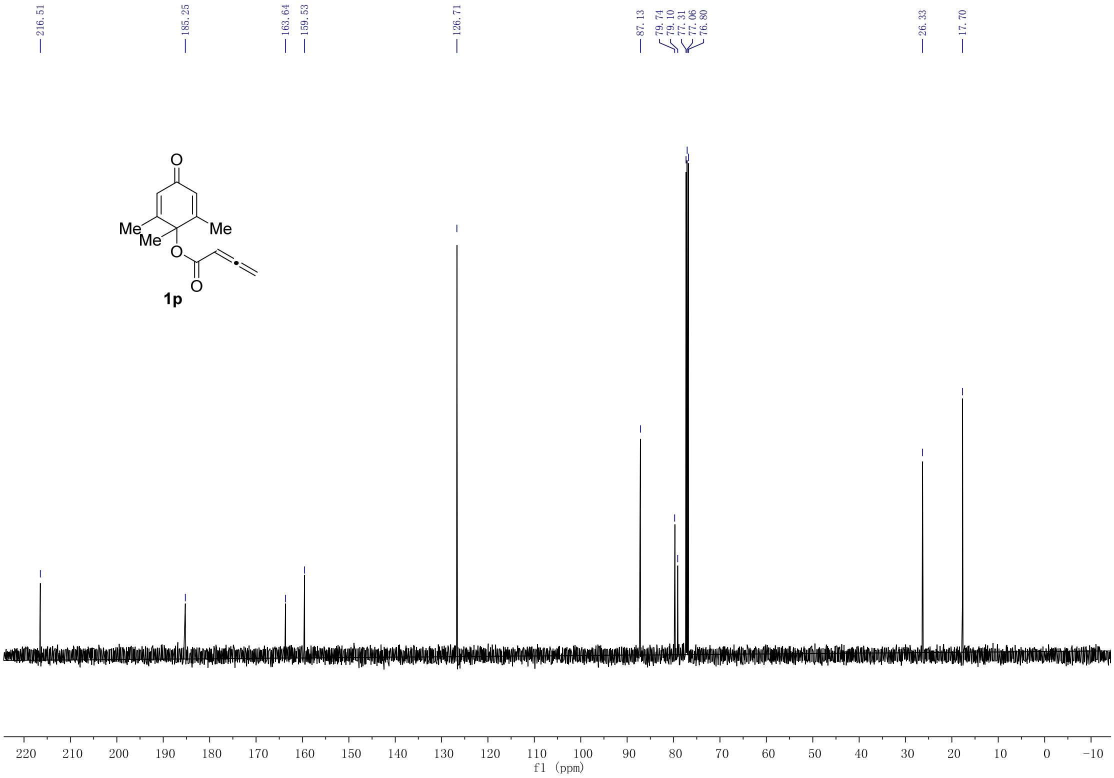

Supplementary Figure 16. ^{1}H and ^{13}C NMR spectra for compound **1p**

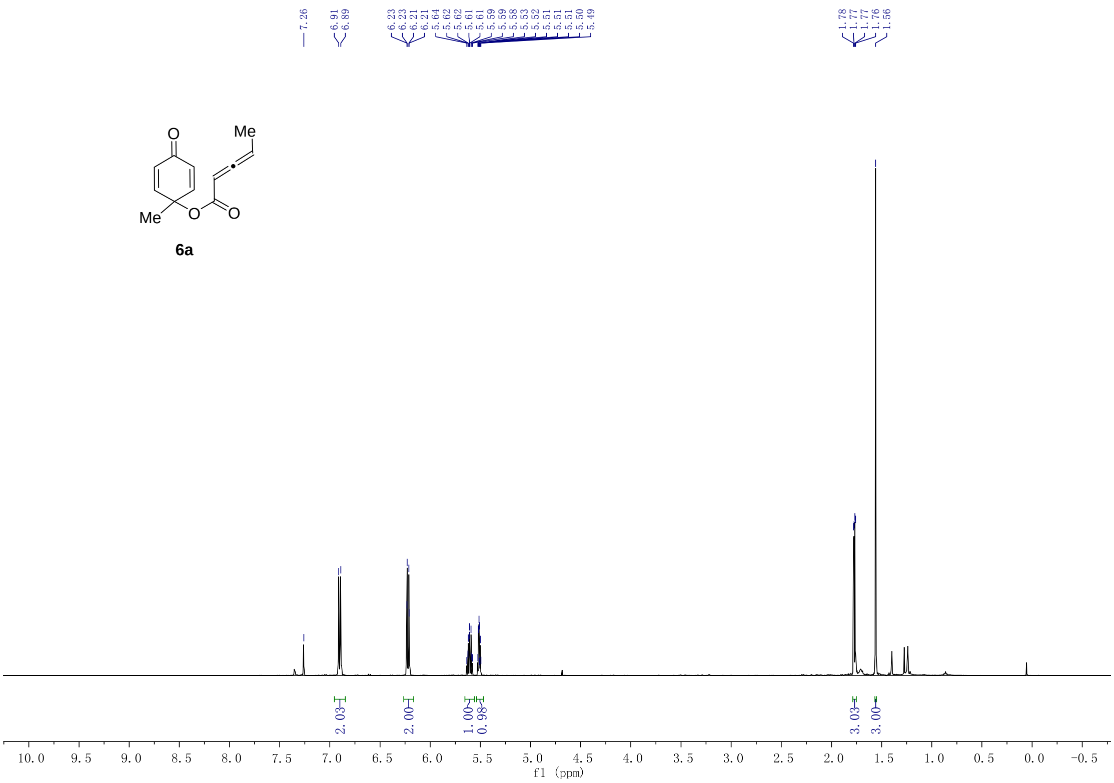

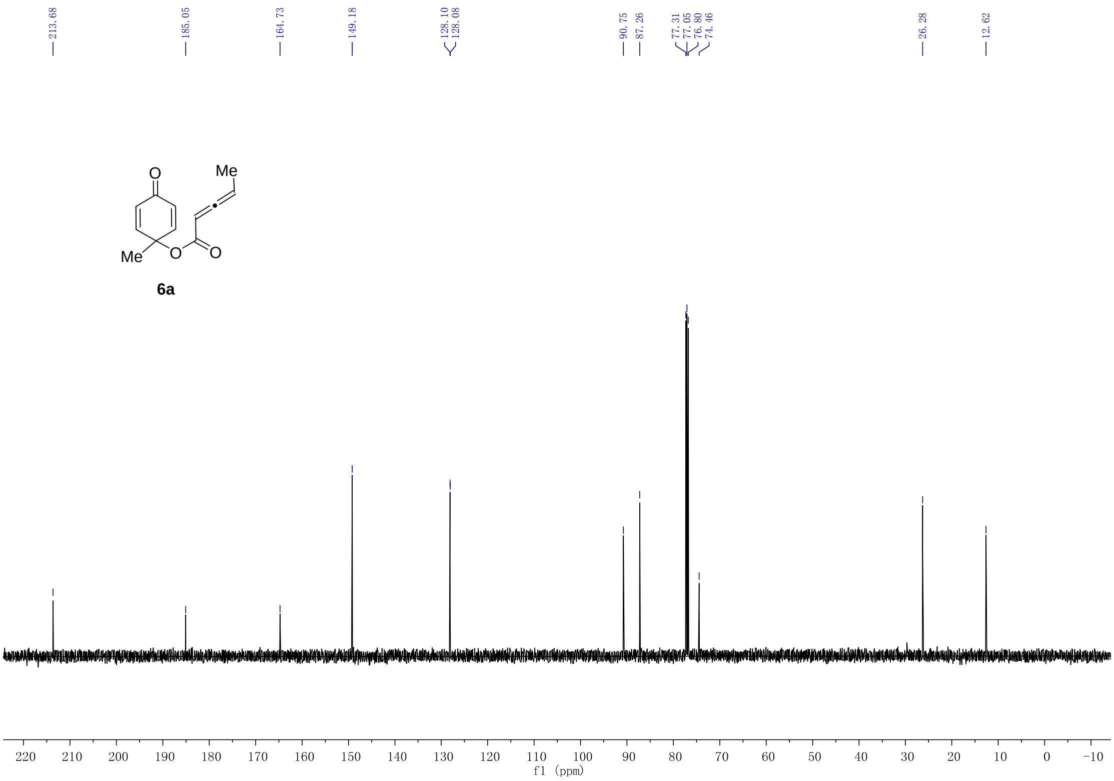

Supplementary Figure 17. ^{1}H and ^{13}C NMR spectra for compound **6a**

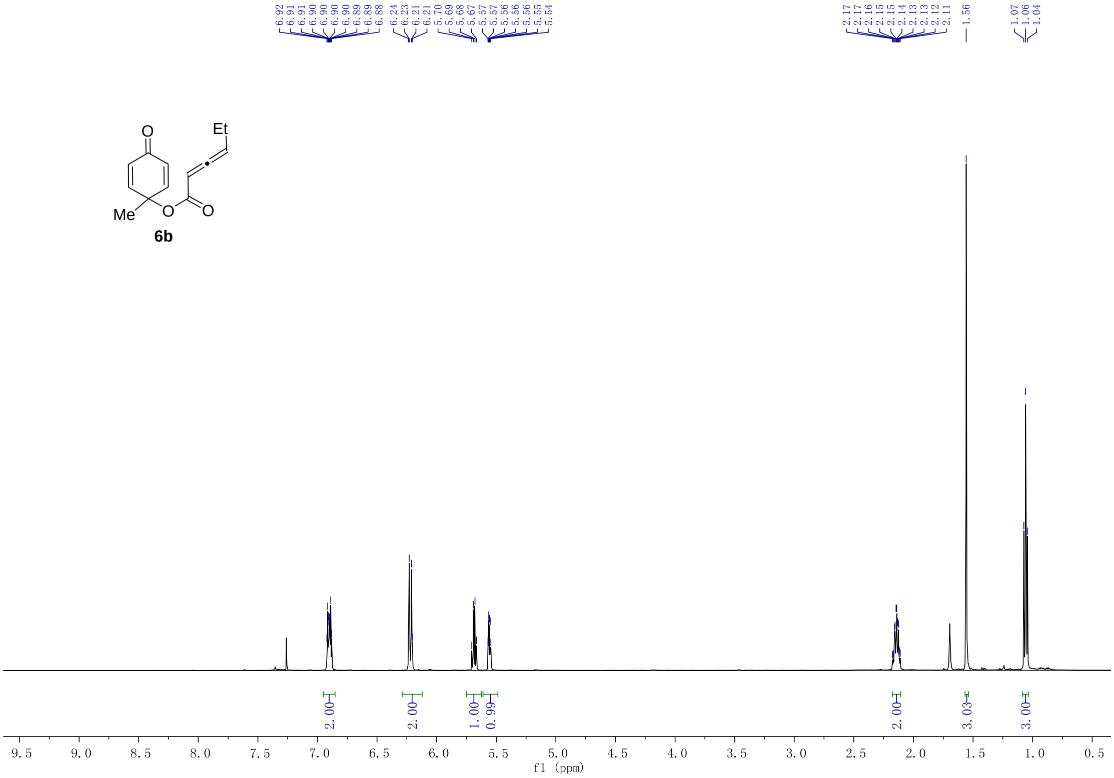

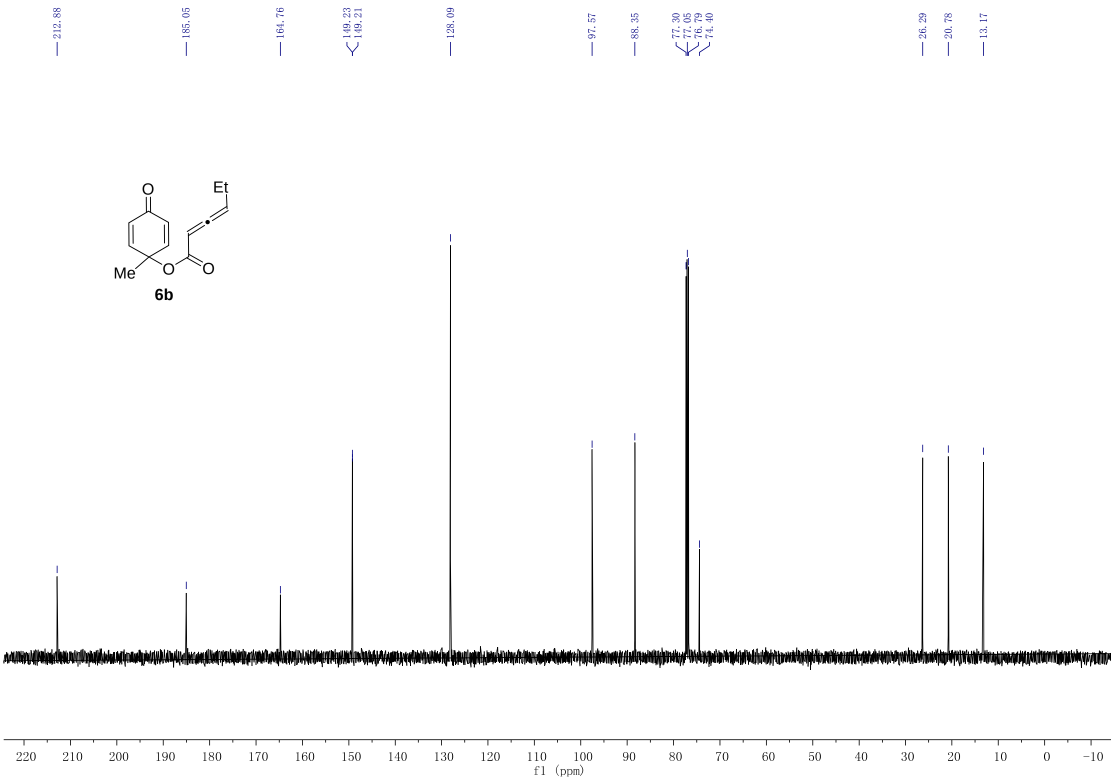

Supplementary Figure 18. ^{1}H and ^{13}C NMR spectra for compound **6b**

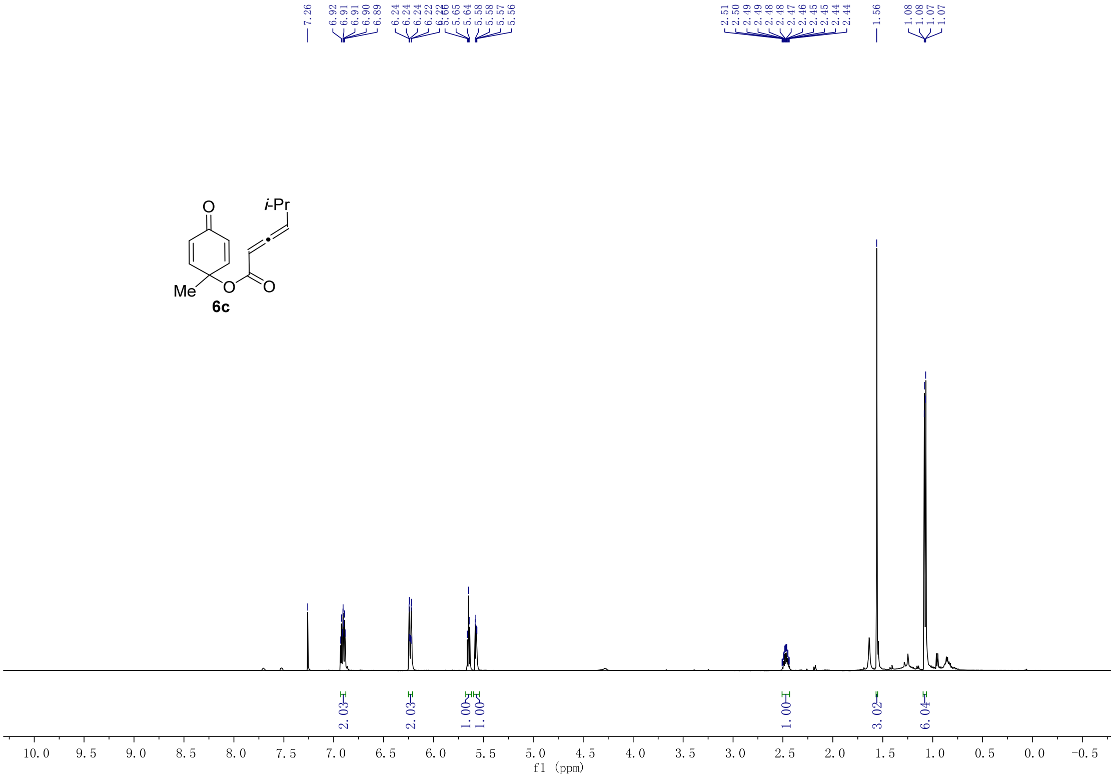

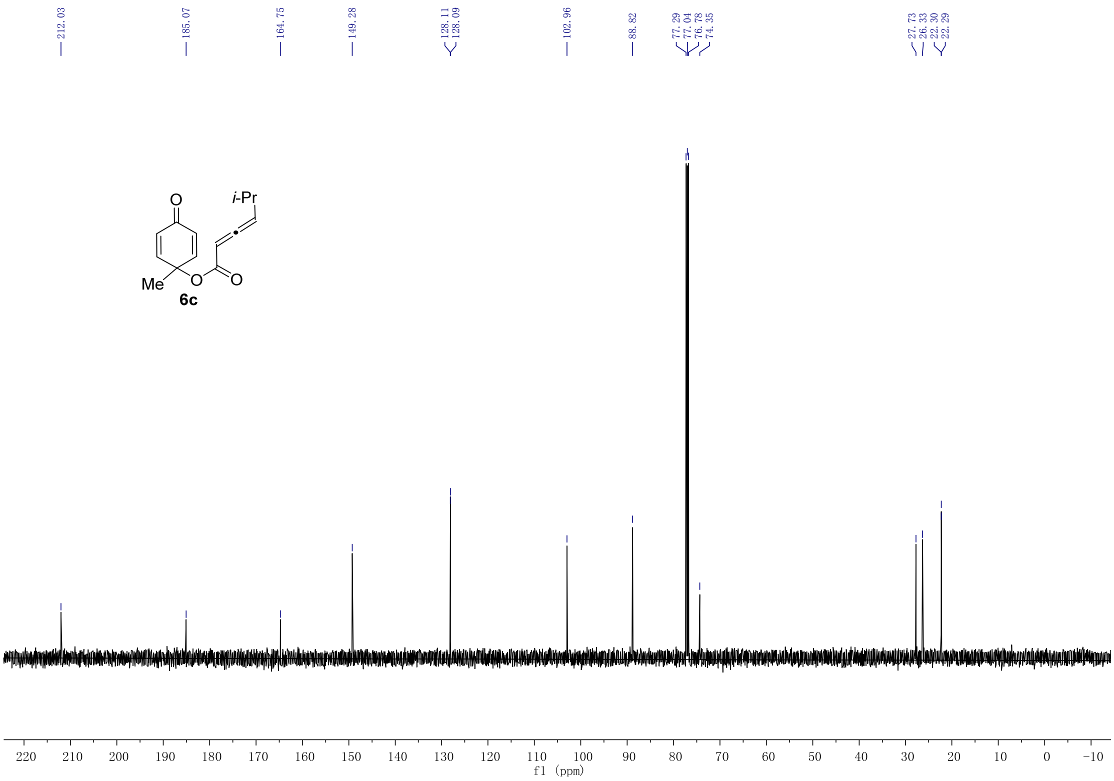

Supplementary Figure 19. ^{1}H and ^{13}C NMR spectra for compound **6c**

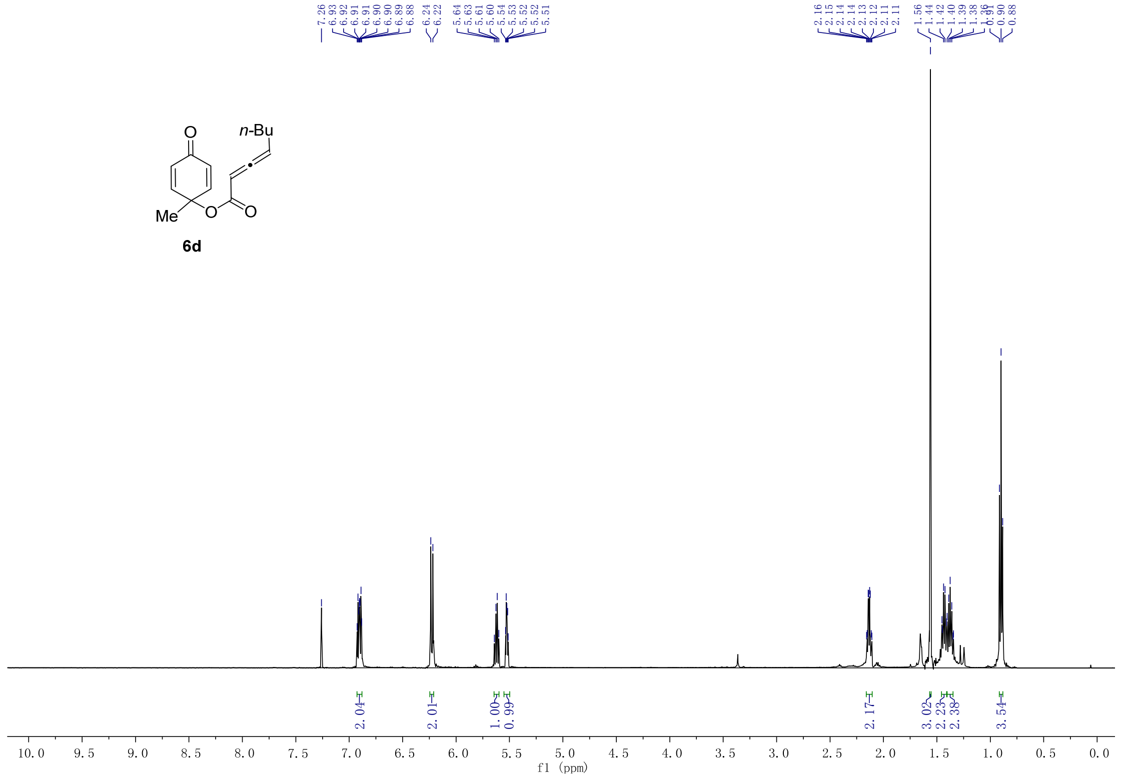

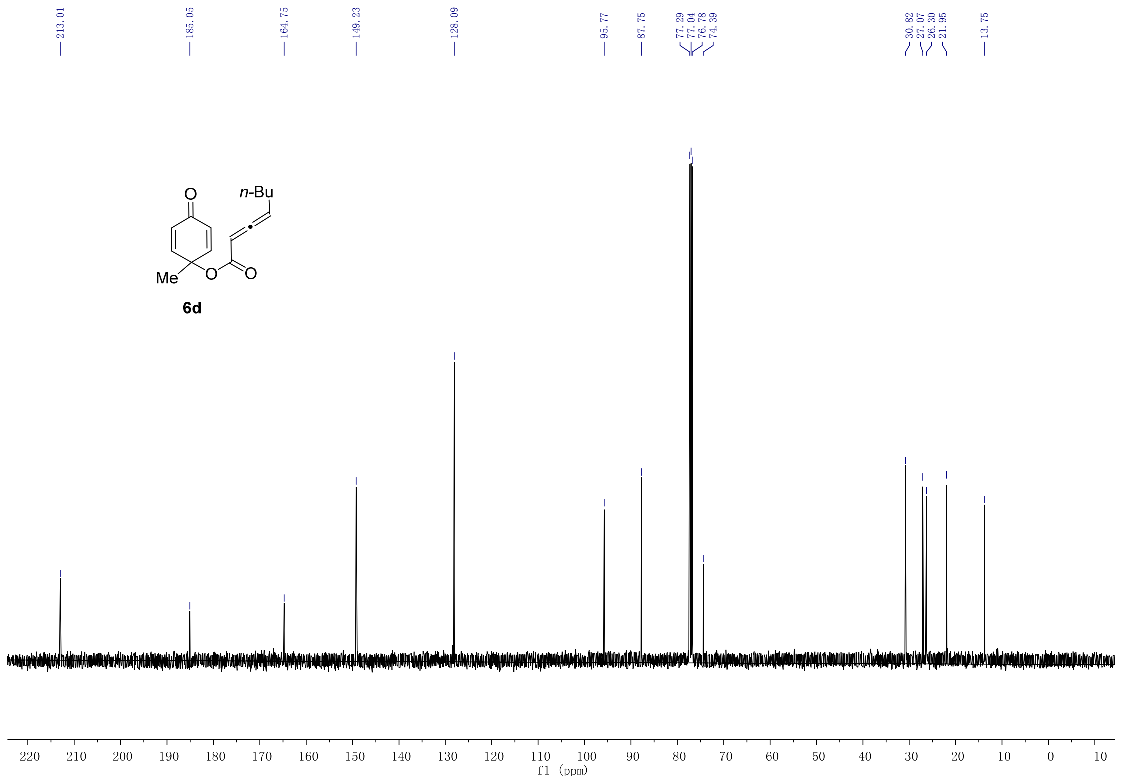

Supplementary Figure 20. ^{1}H and ^{13}C NMR spectra for compound **6d**

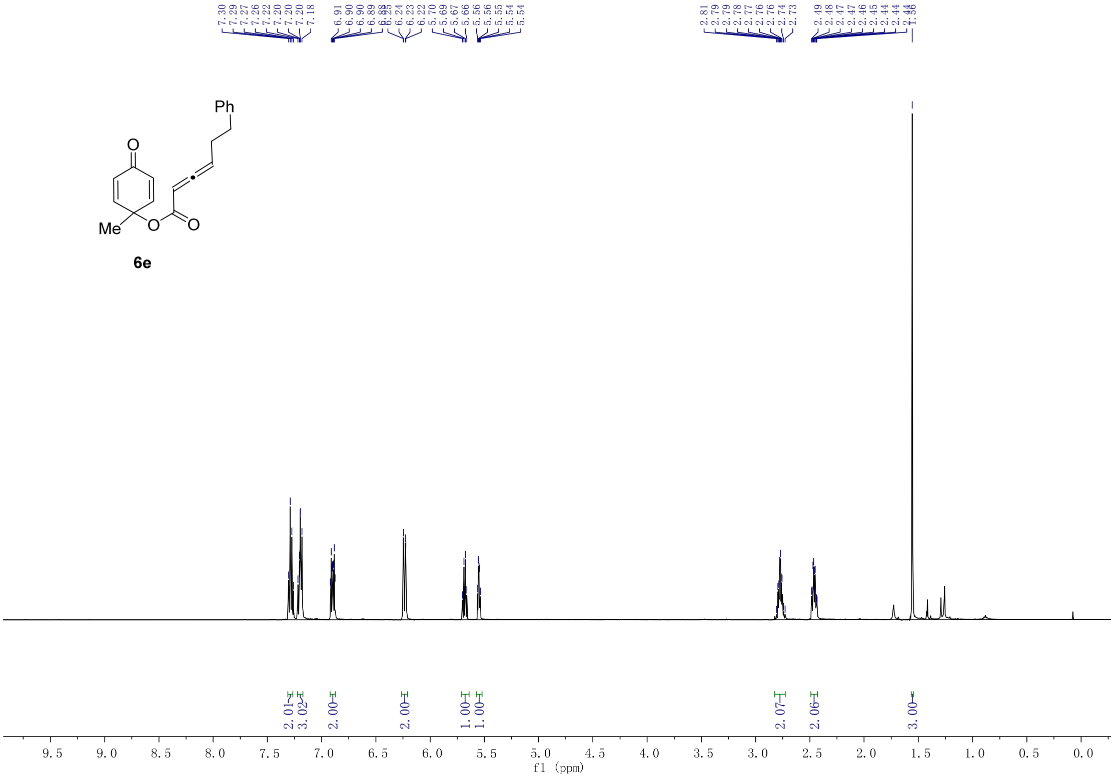

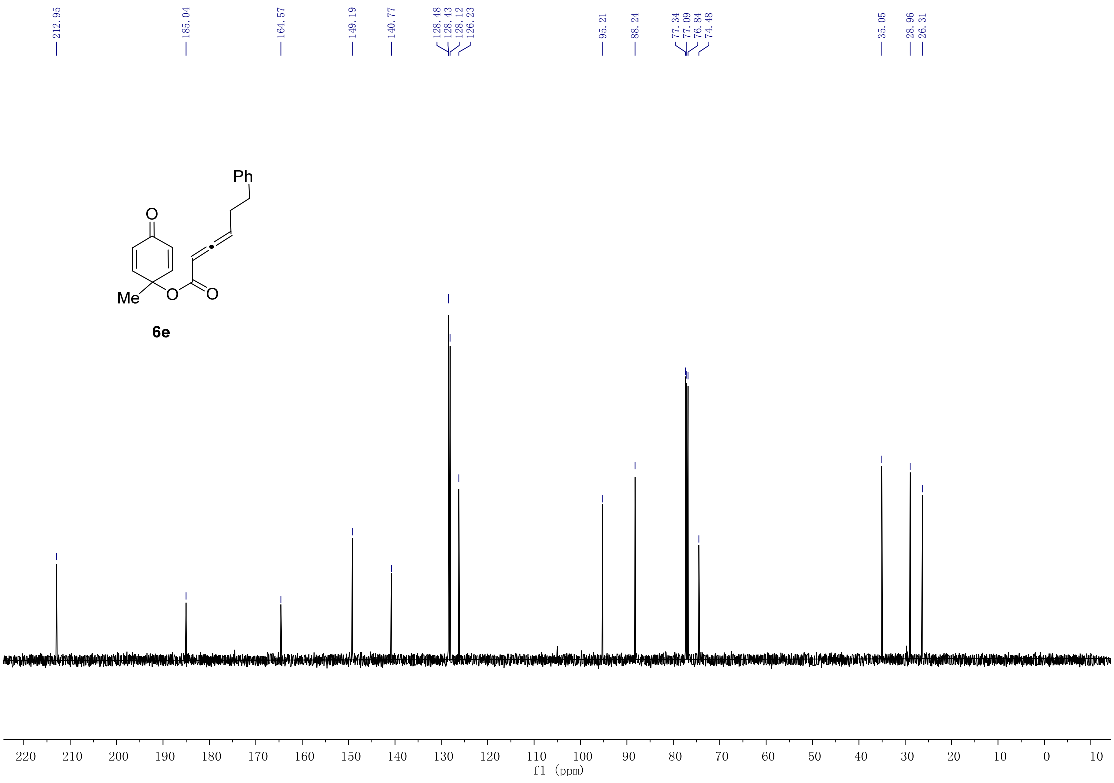

Supplementary Figure 21. ^{1}H and ^{13}C NMR spectra for compound **6e**

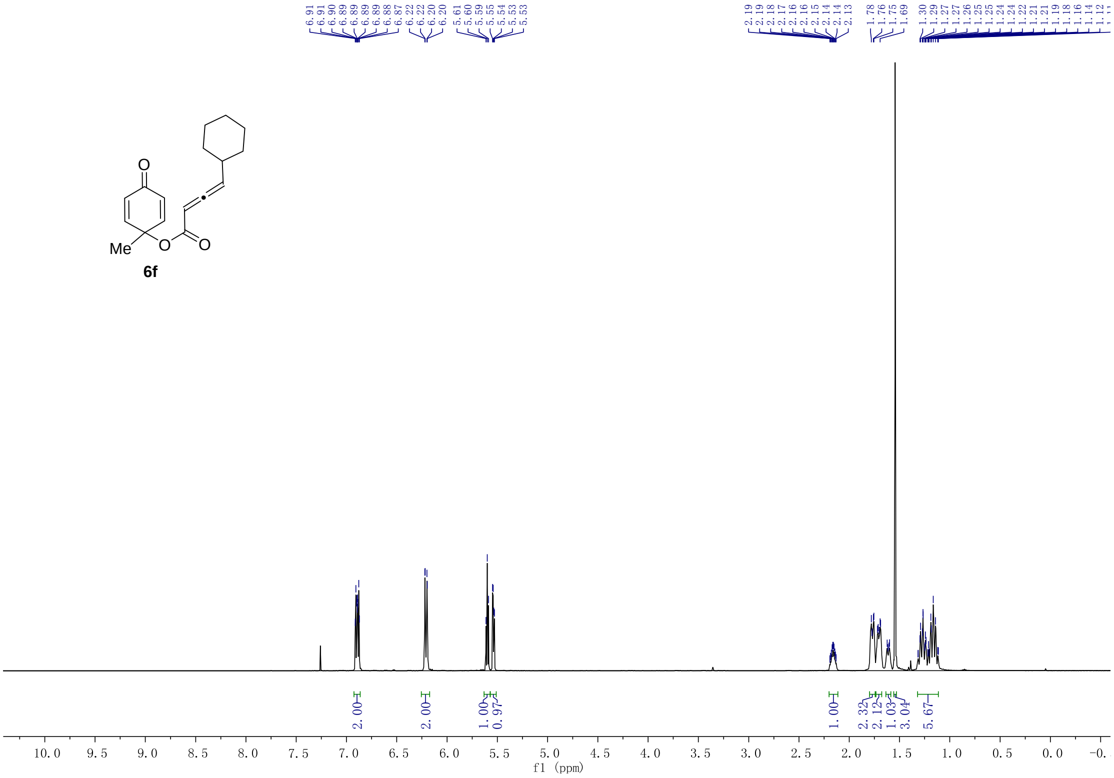

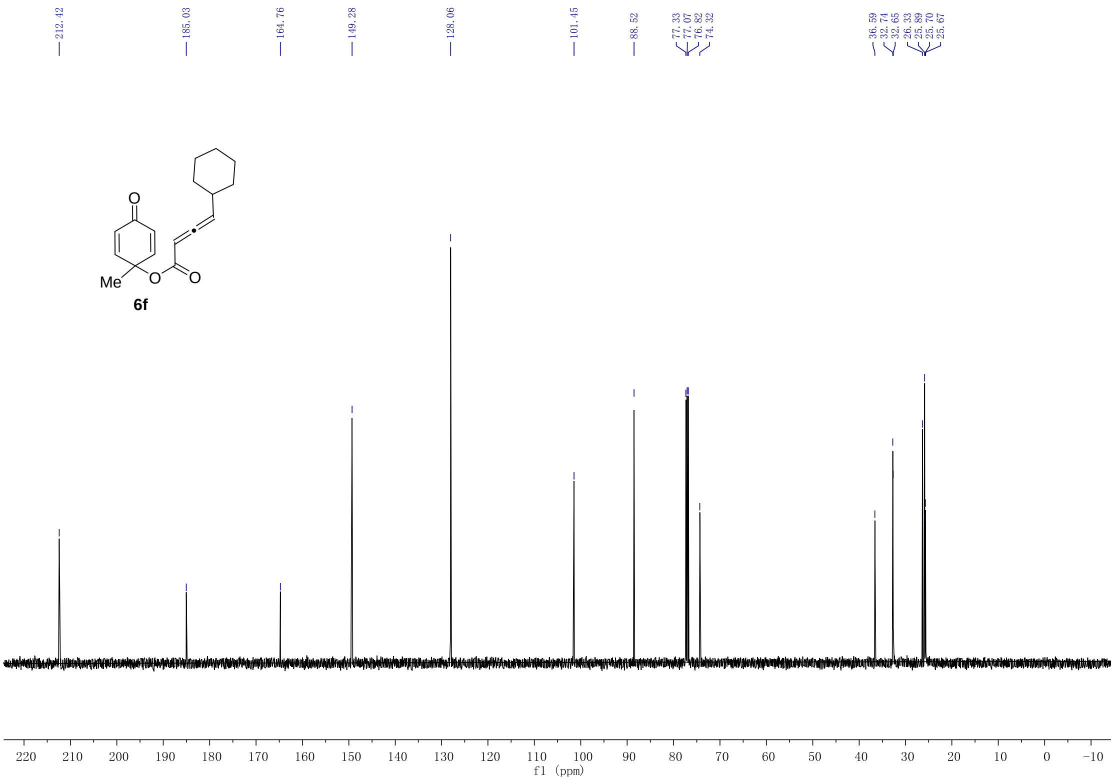

Supplementary Figure 22. ^{1}H and ^{13}C NMR spectra for compound **6f**

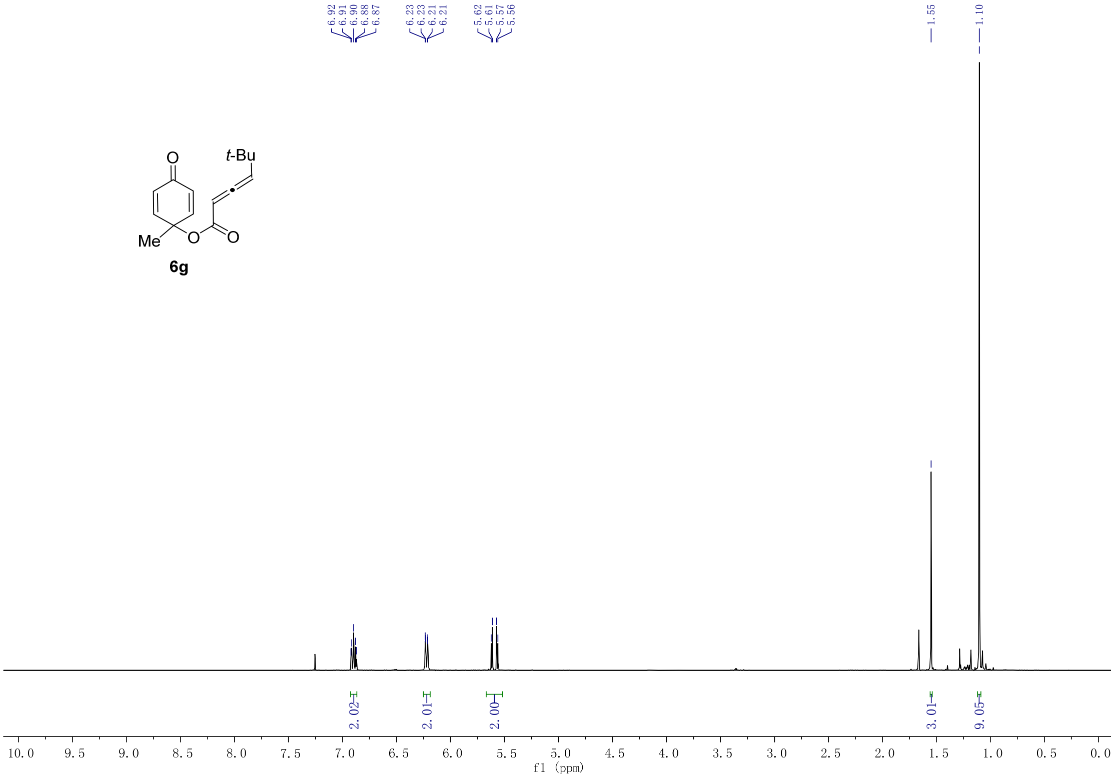

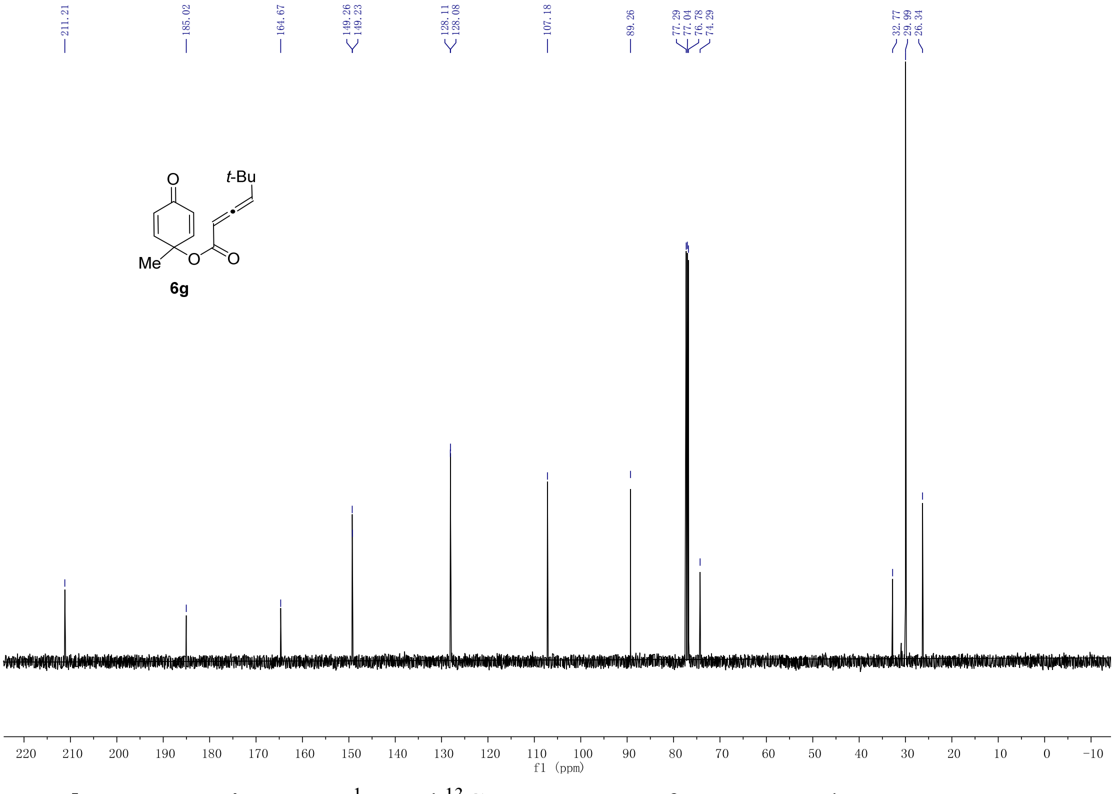

Supplementary Figure 23. ^{1}H and ^{13}C NMR spectra for compound **6g**

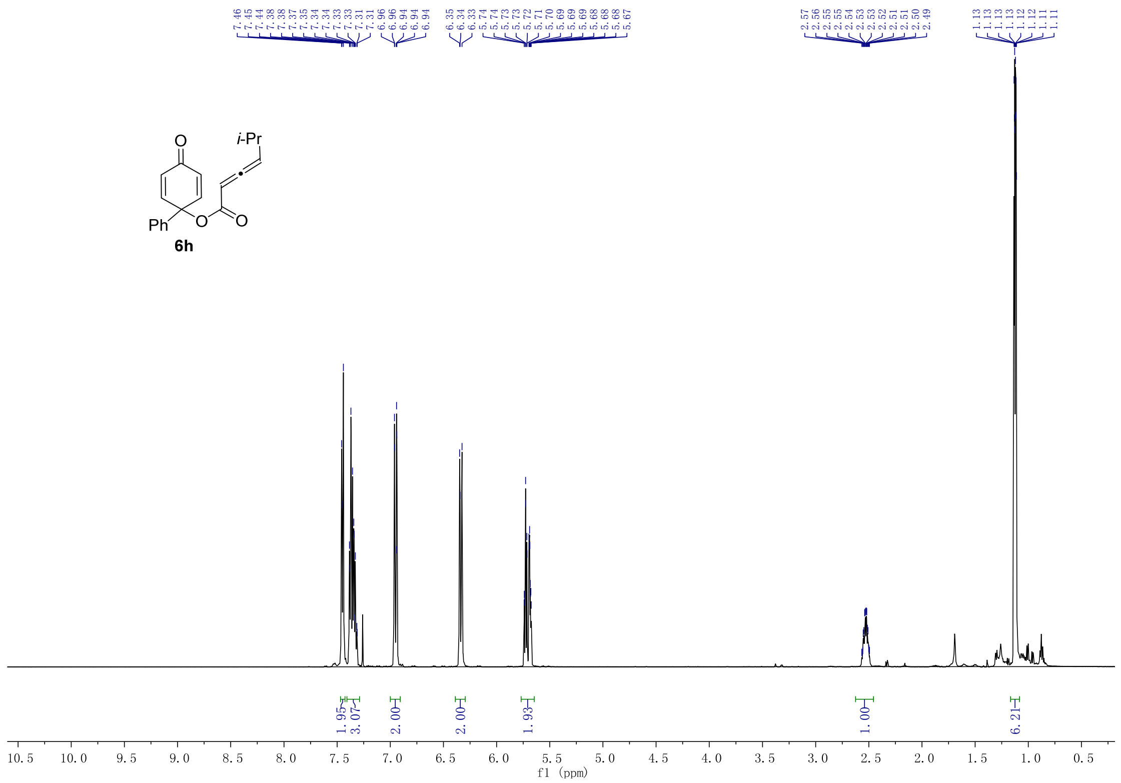

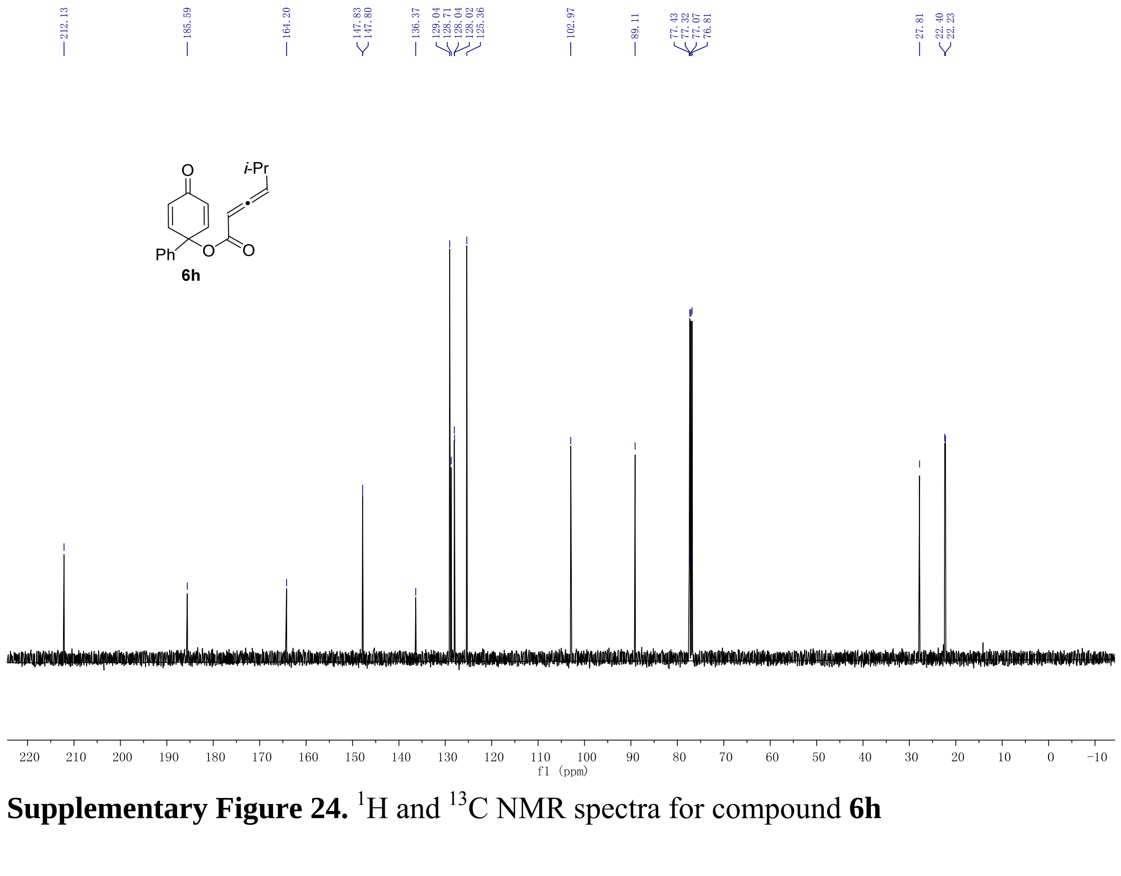

Supplementary Figure 24. ^{1}H and ^{13}C NMR spectra for compound **6h**

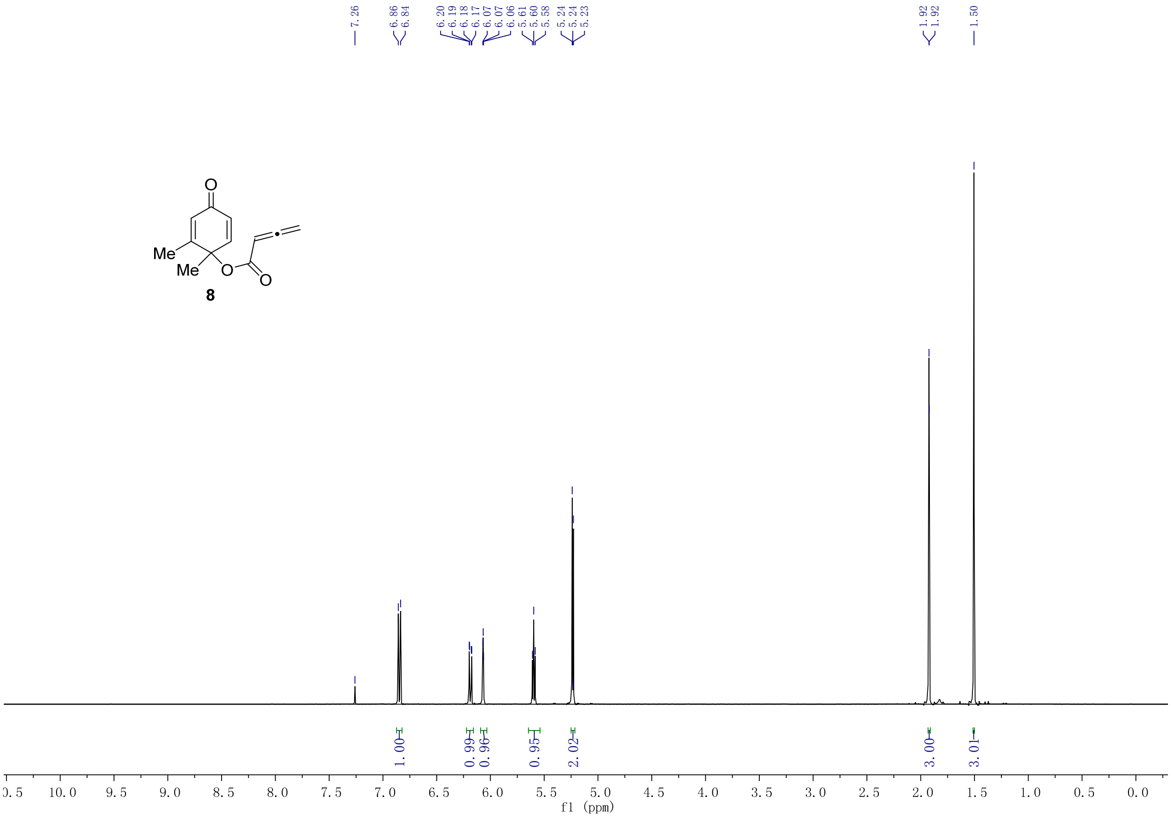

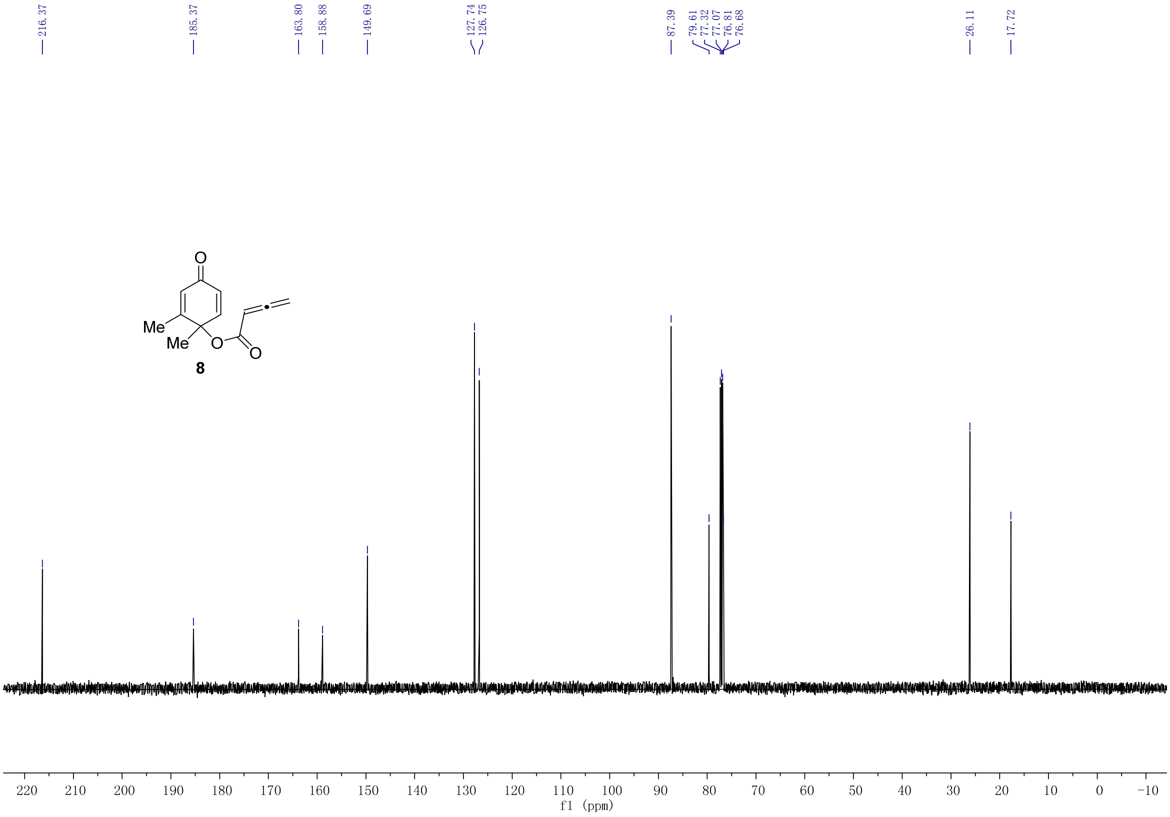

Supplementary Figure 25. ^{1}H and ^{13}C NMR spectra for compound **8**

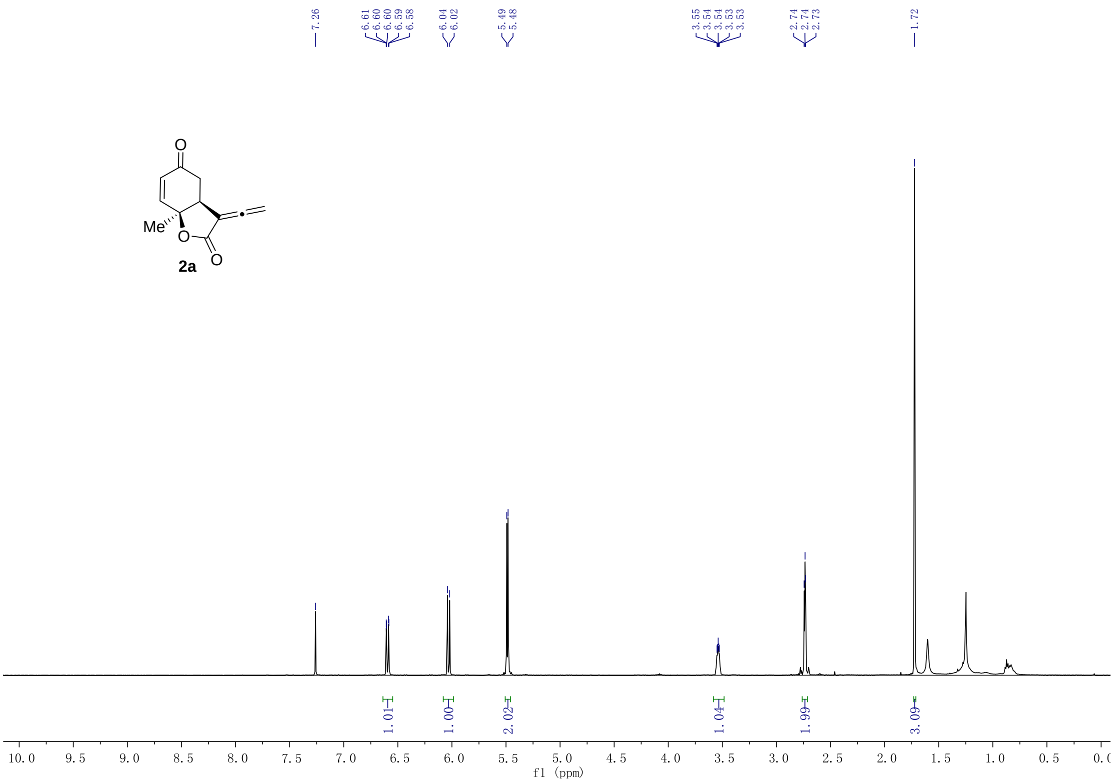

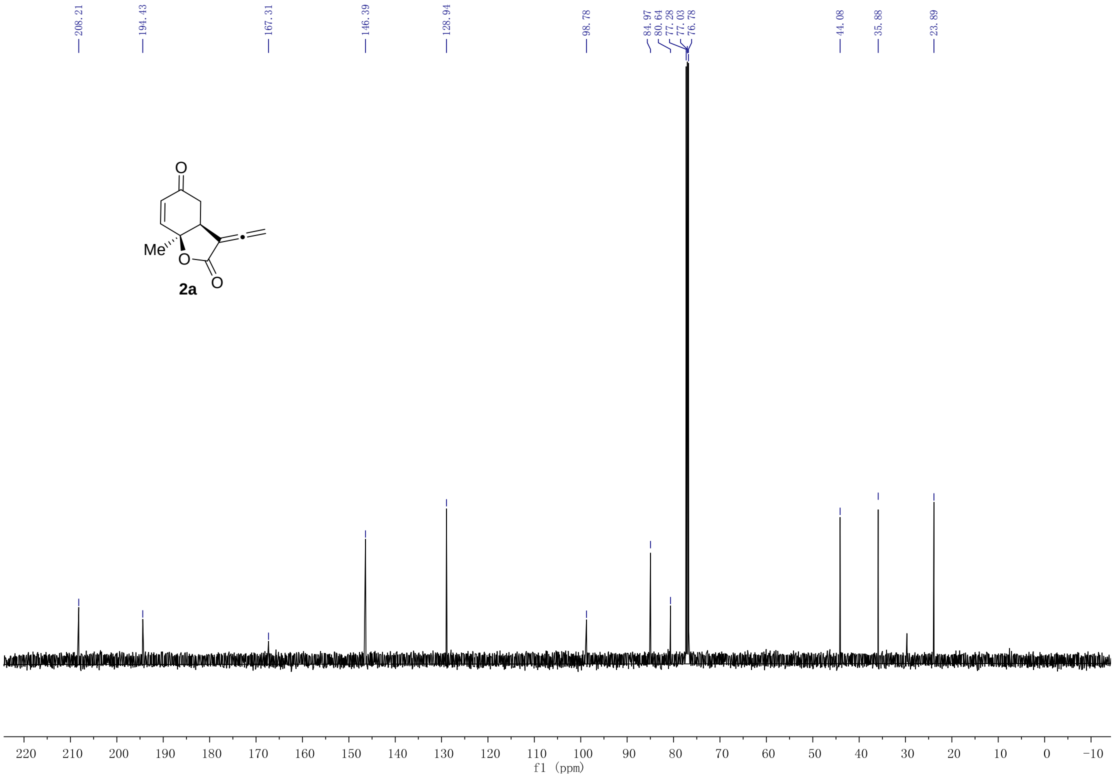

Supplementary Figure 26. ^{1}H and ^{13}C NMR spectra for compound **2a**

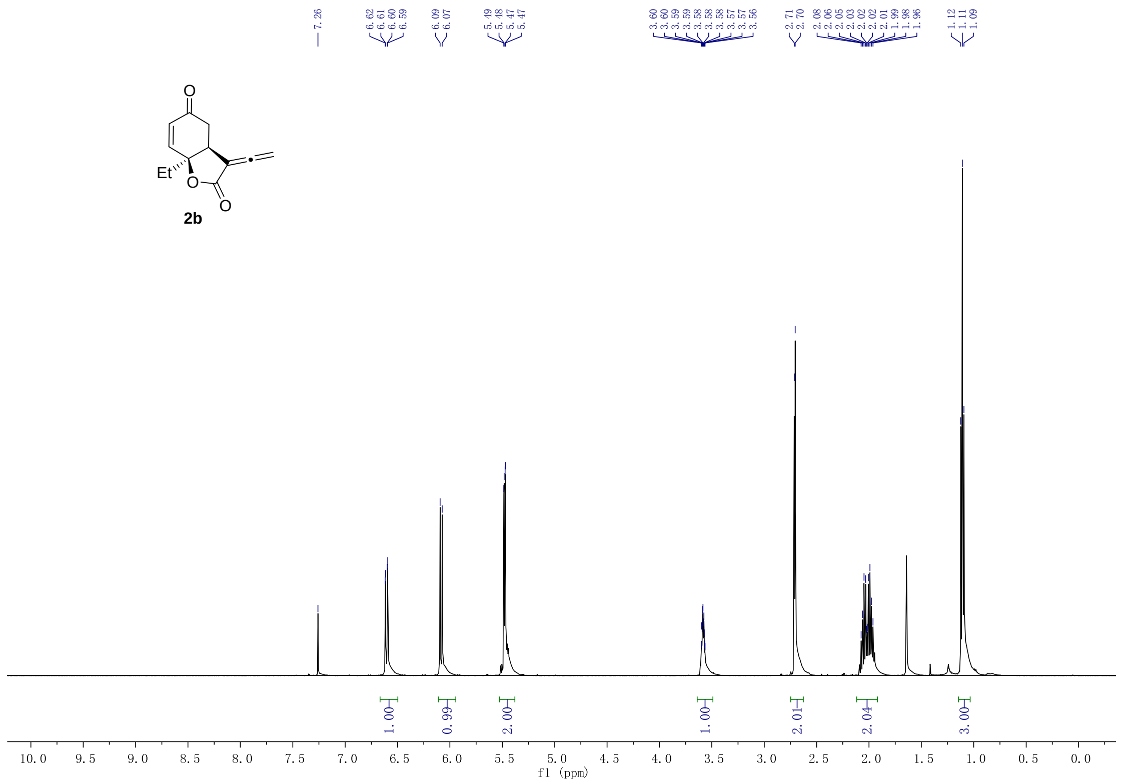

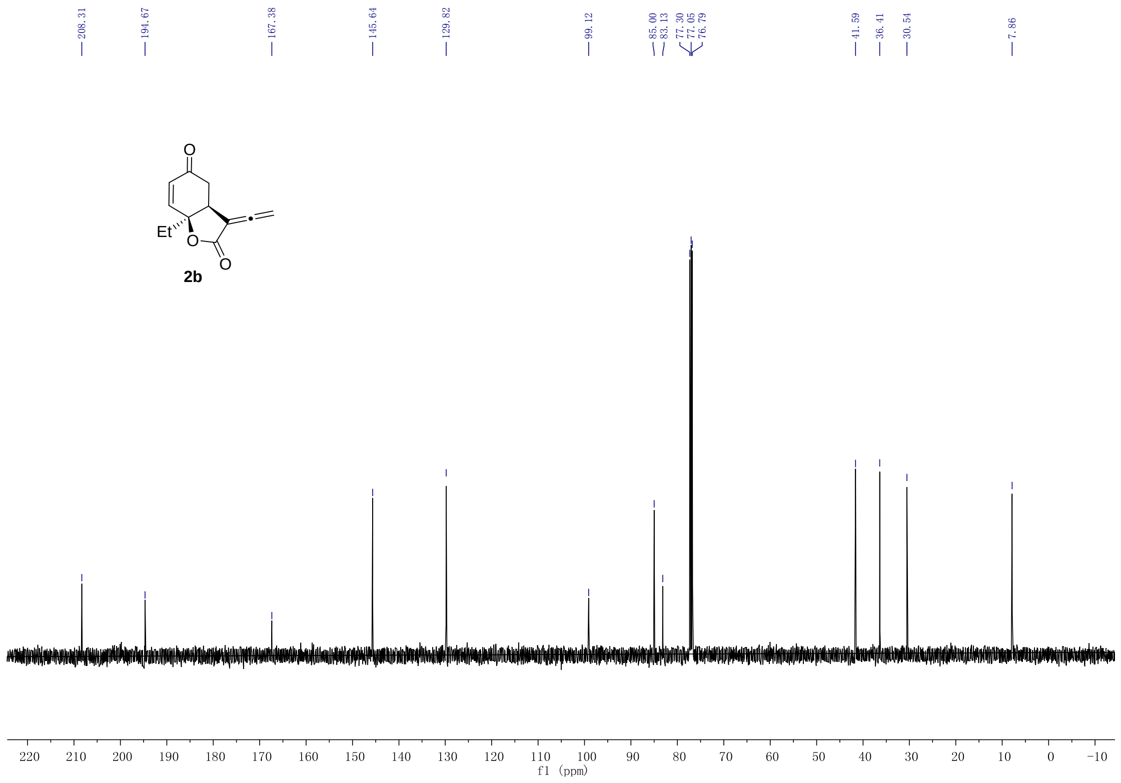

Supplementary Figure 27. ^{1}H and ^{13}C NMR spectra for compound **2b**

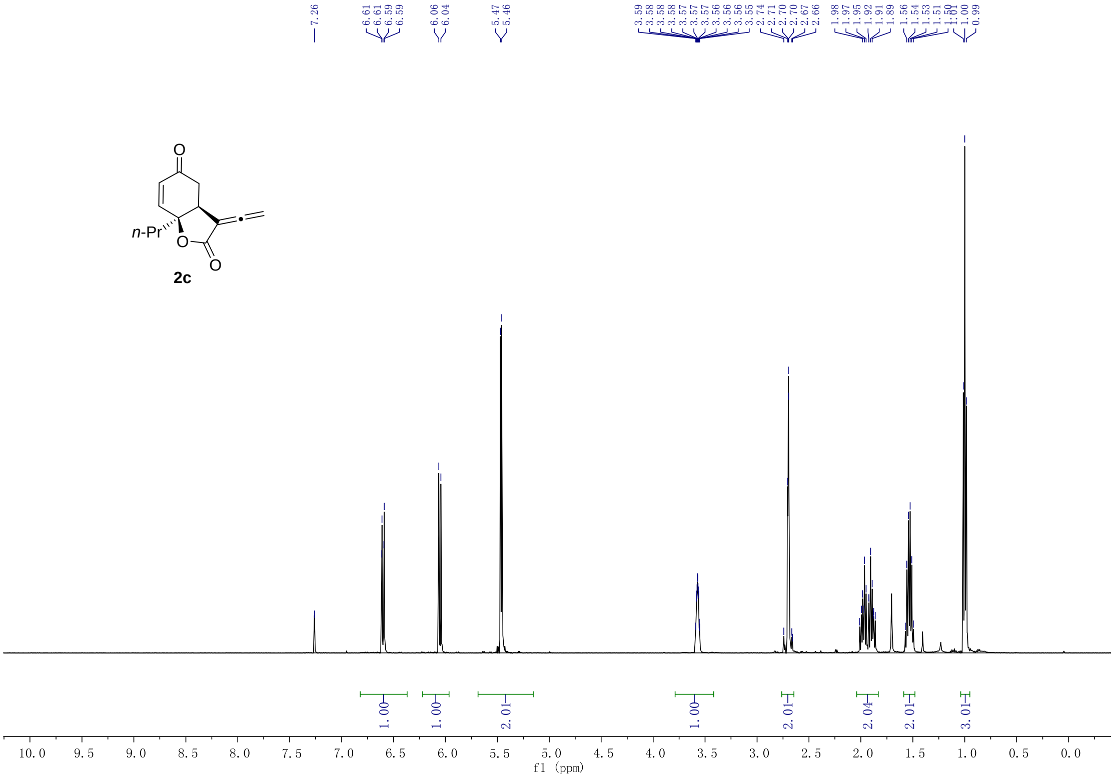

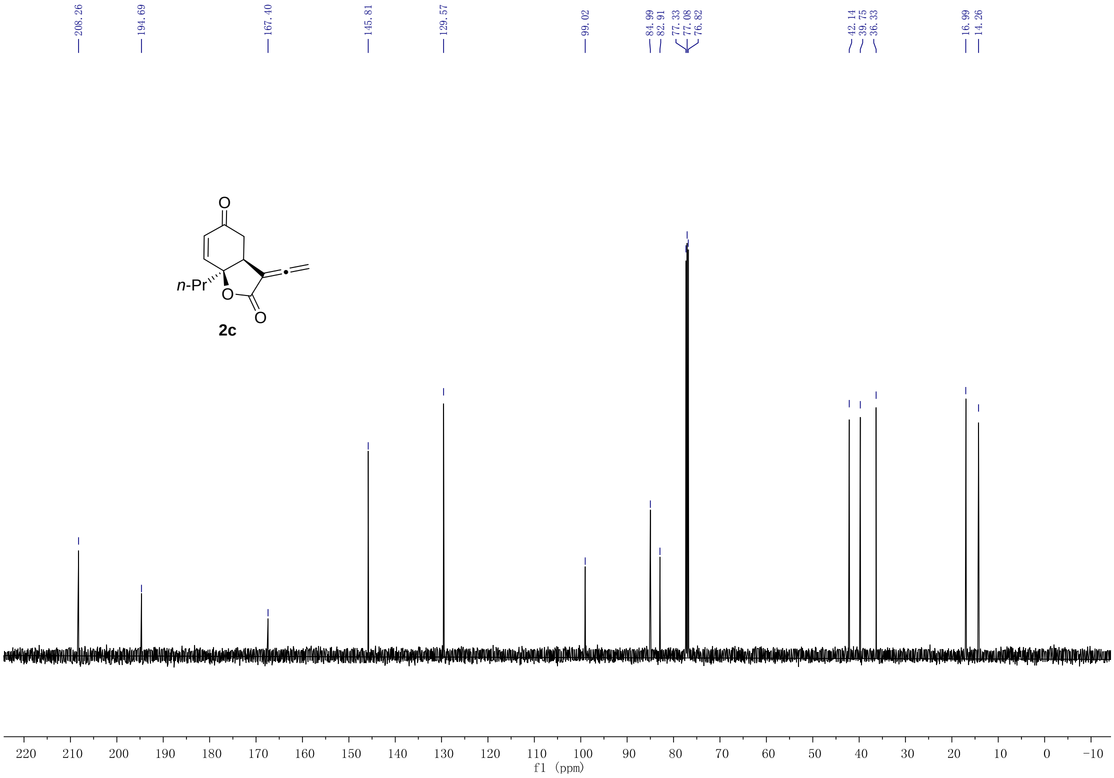

Supplementary Figure 28. ^{1}H and ^{13}C NMR spectra for compound **2c**

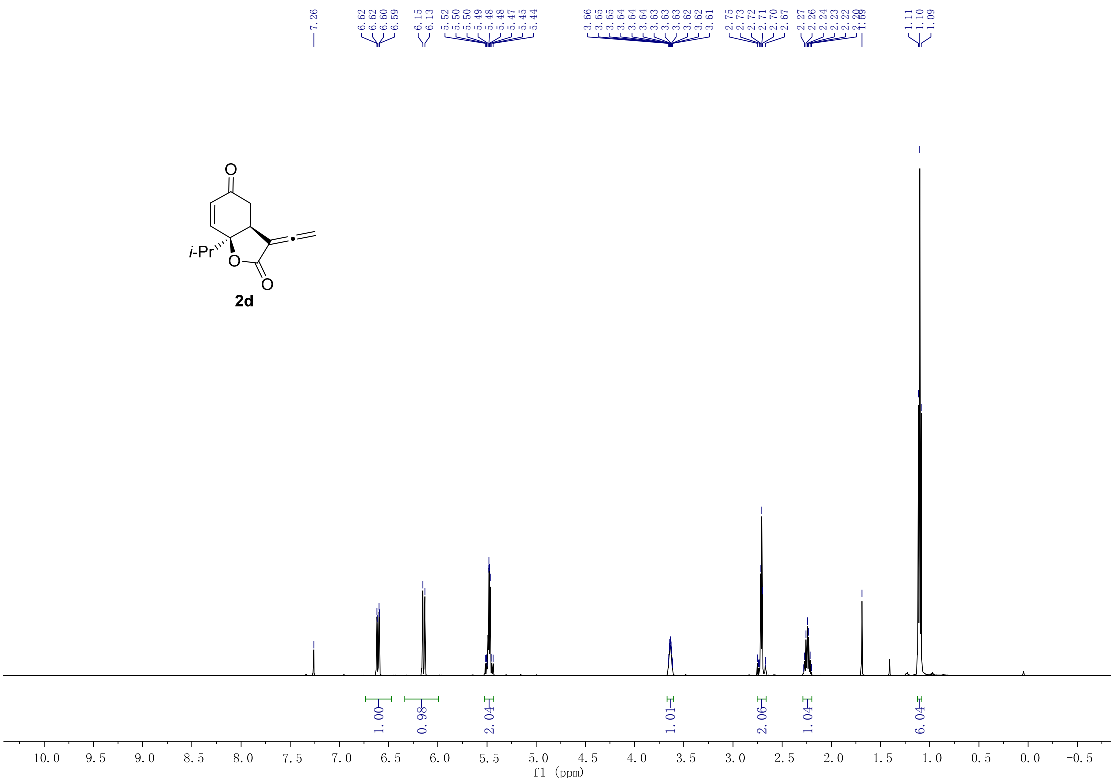

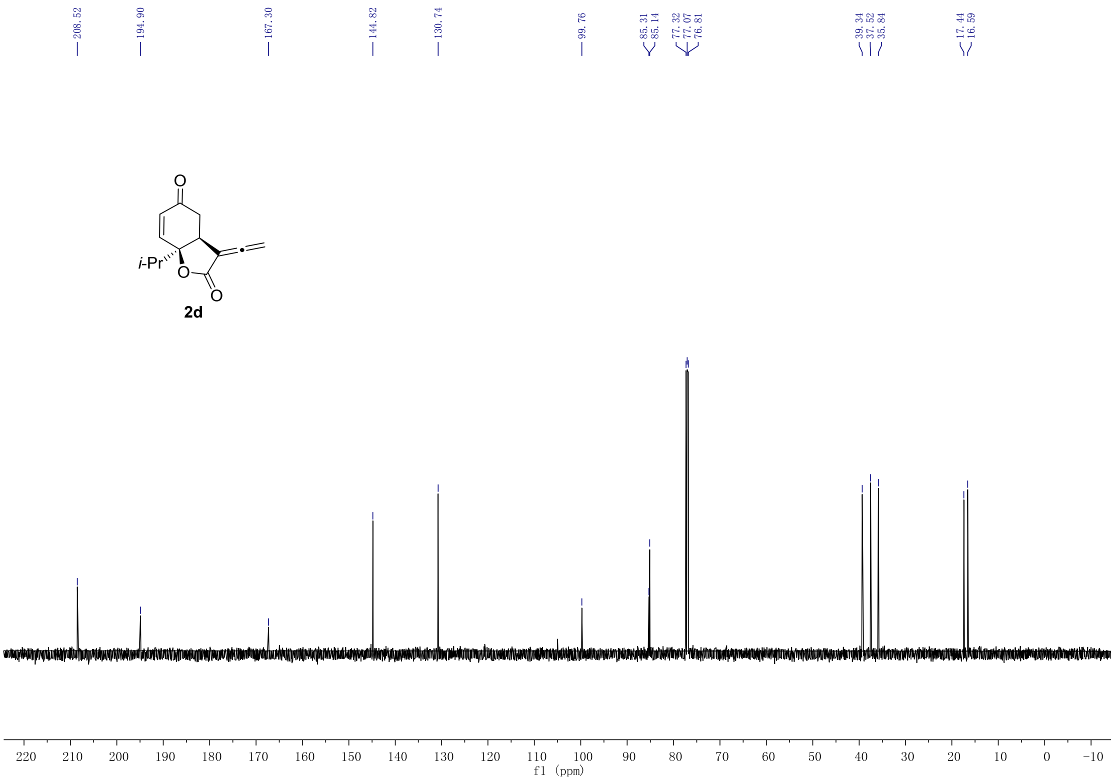

Supplementary Figure 29. ^{1}H and ^{13}C NMR spectra for compound **2d**

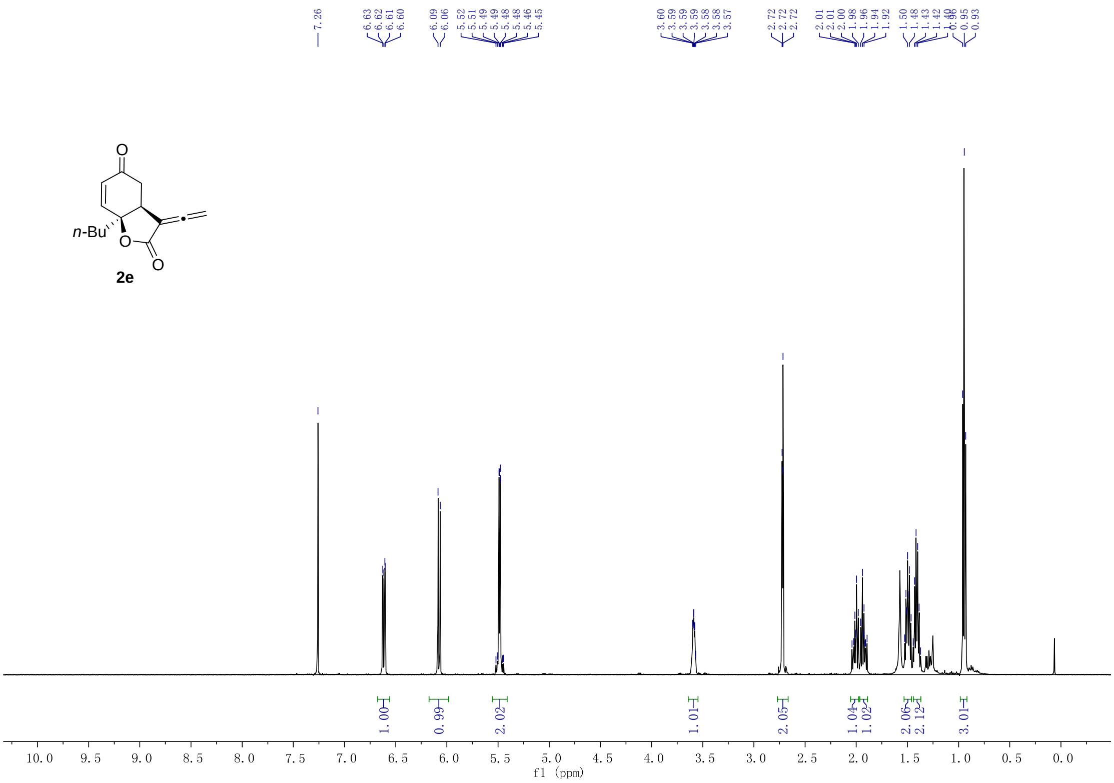

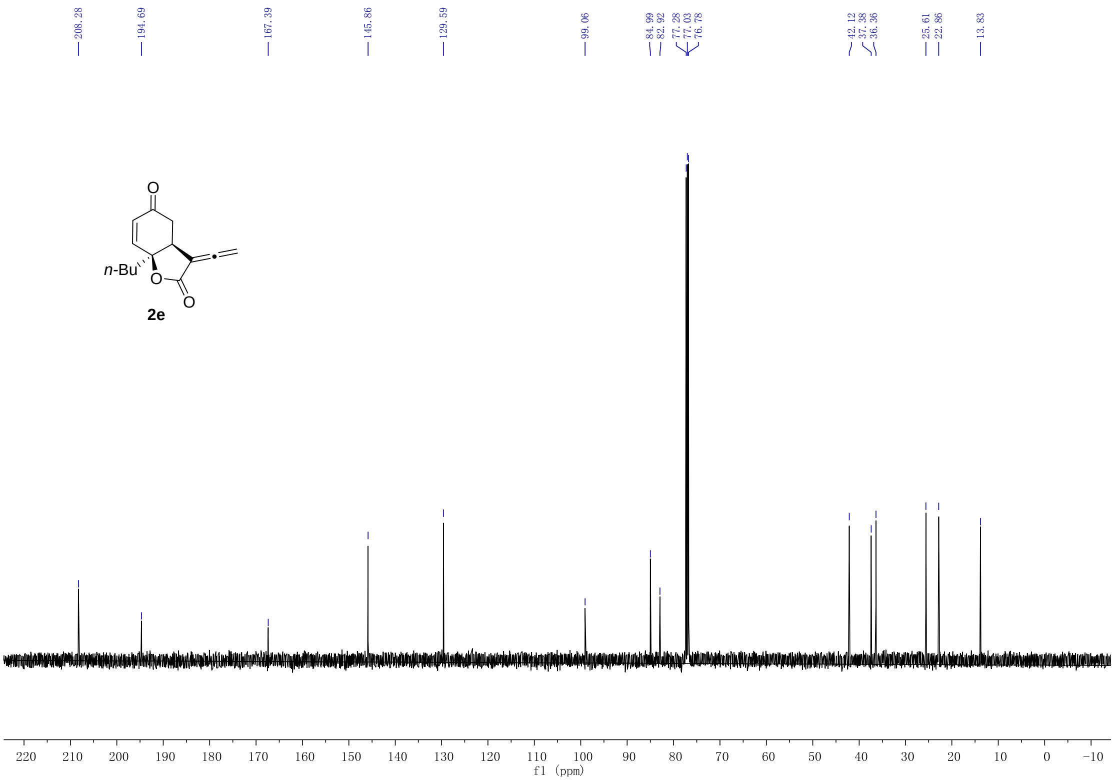

Supplementary Figure 30. ^{1}H and ^{13}C NMR spectra for compound **2e**

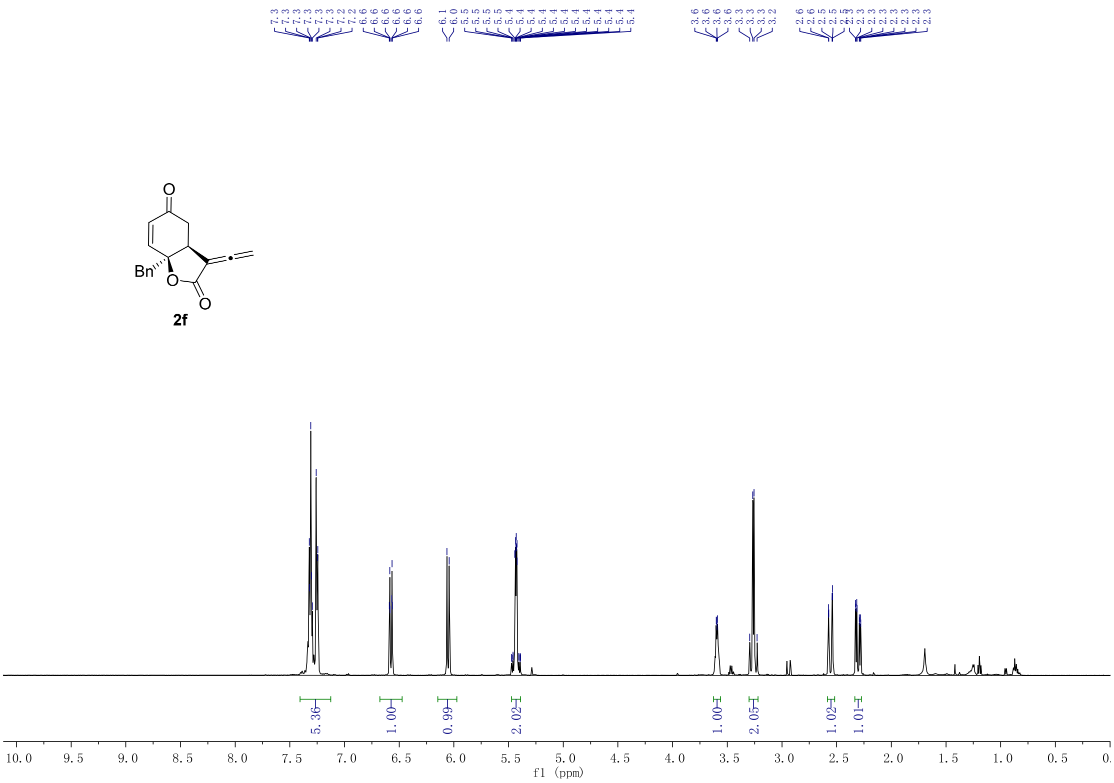

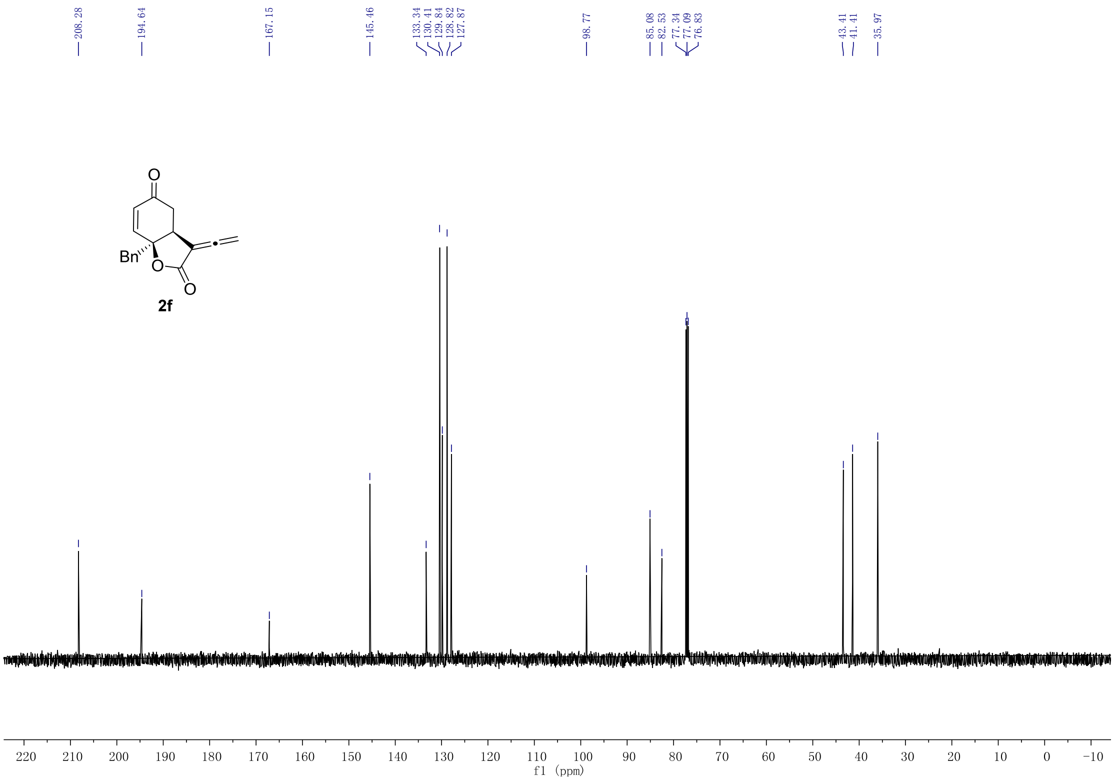

Supplementary Figure 31. ^{1}H and ^{13}C NMR spectra for compound **2f**

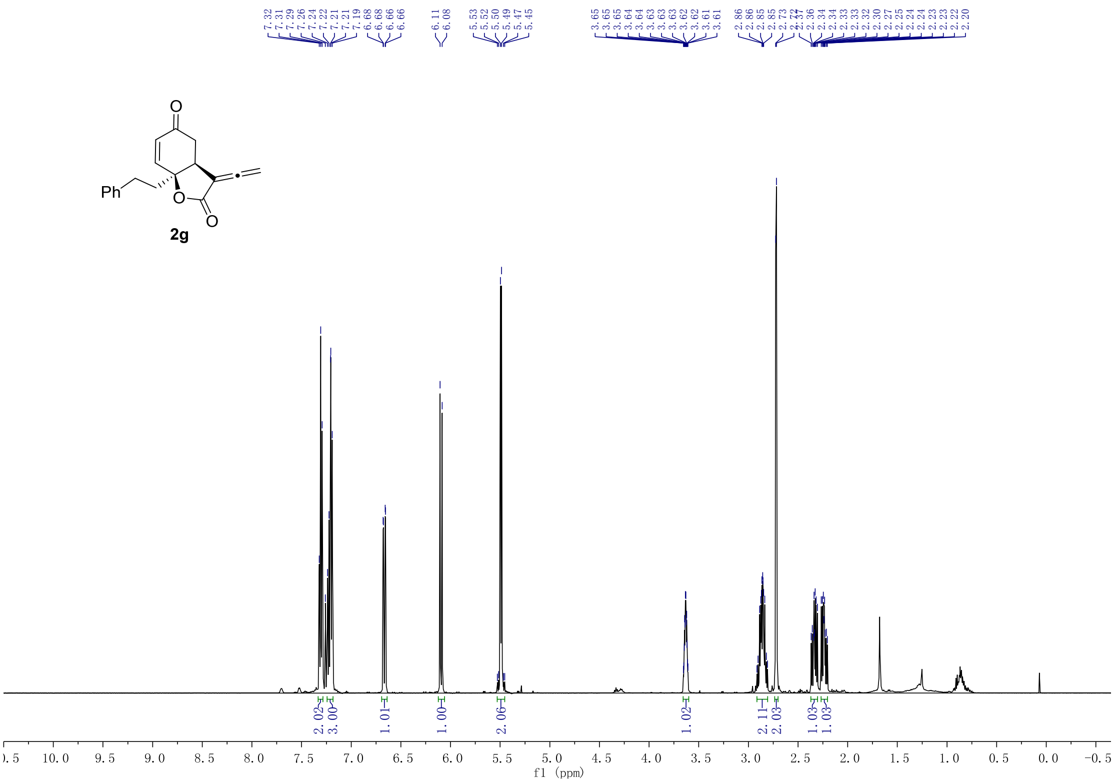

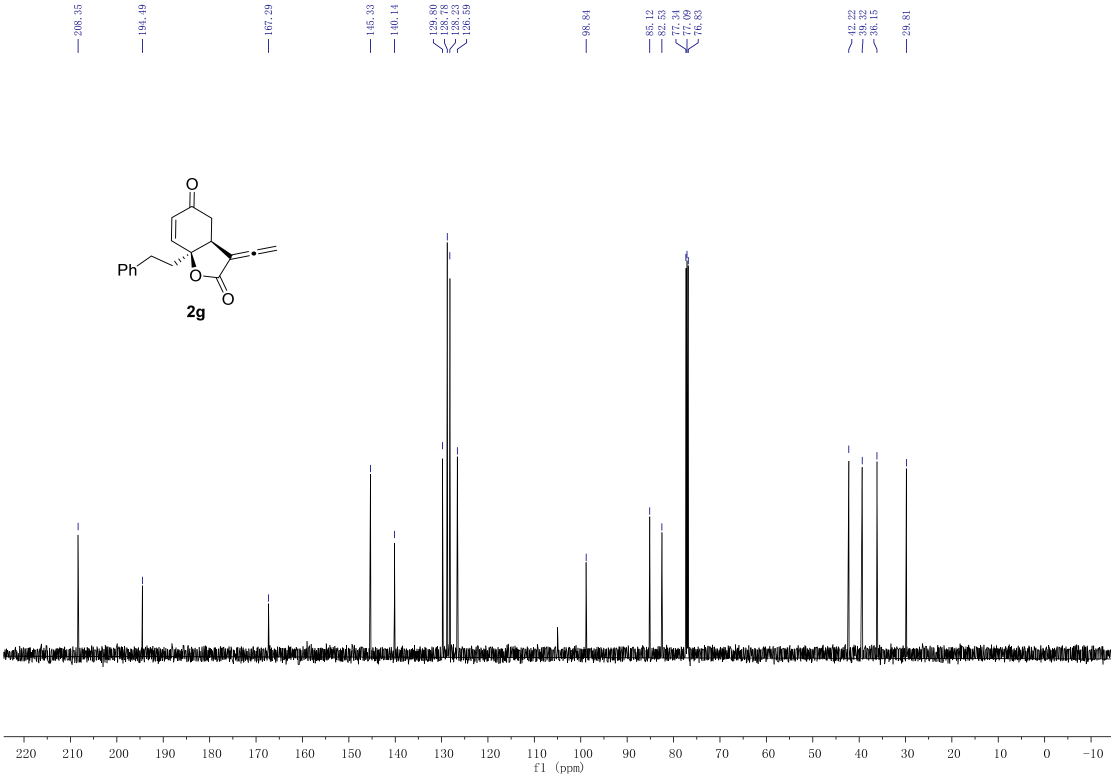

Supplementary Figure 32. ^{1}H and ^{13}C NMR spectra for compound **2g**

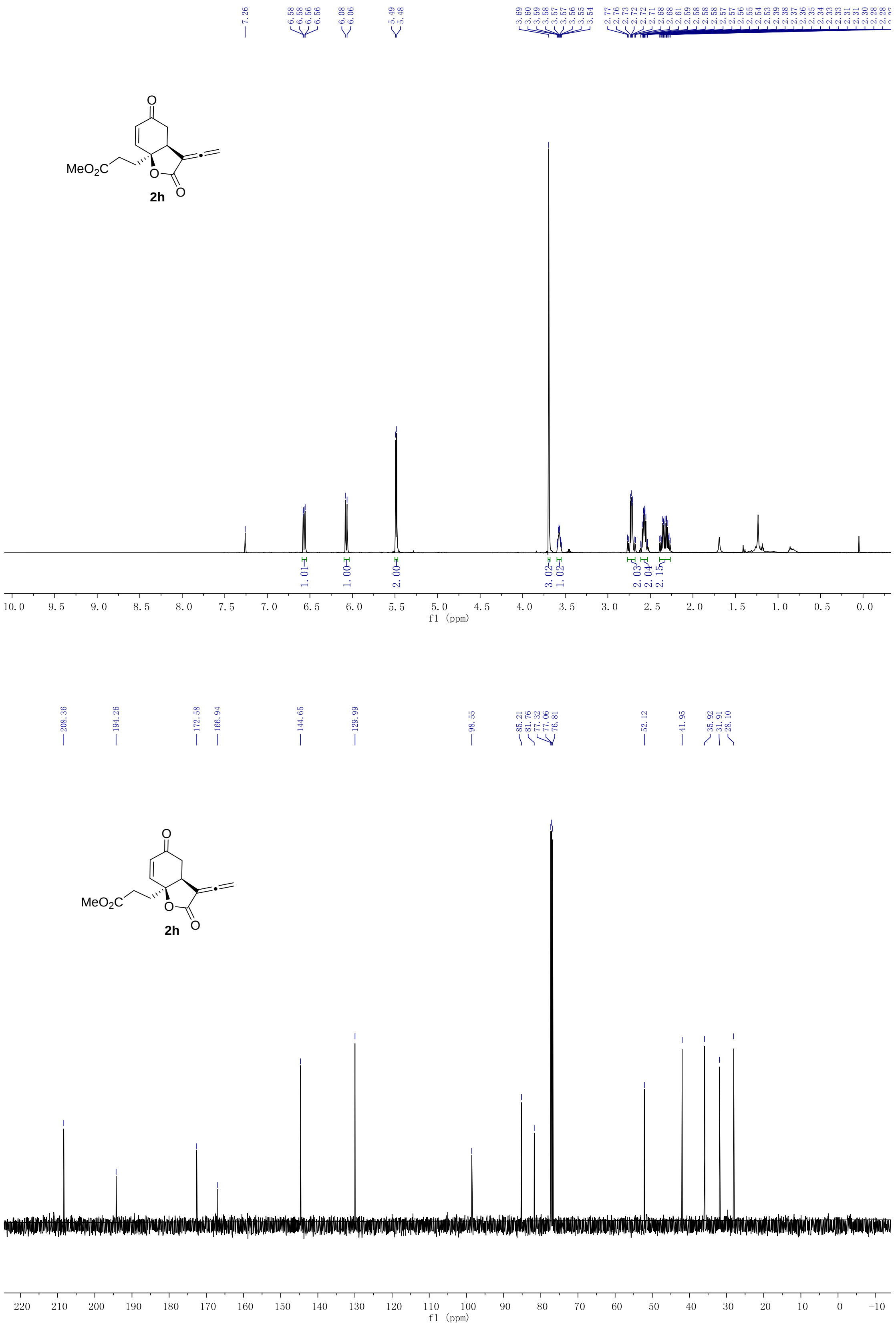

Supplementary Figure 33. ^{1}H and ^{13}C NMR spectra for compound **2h**

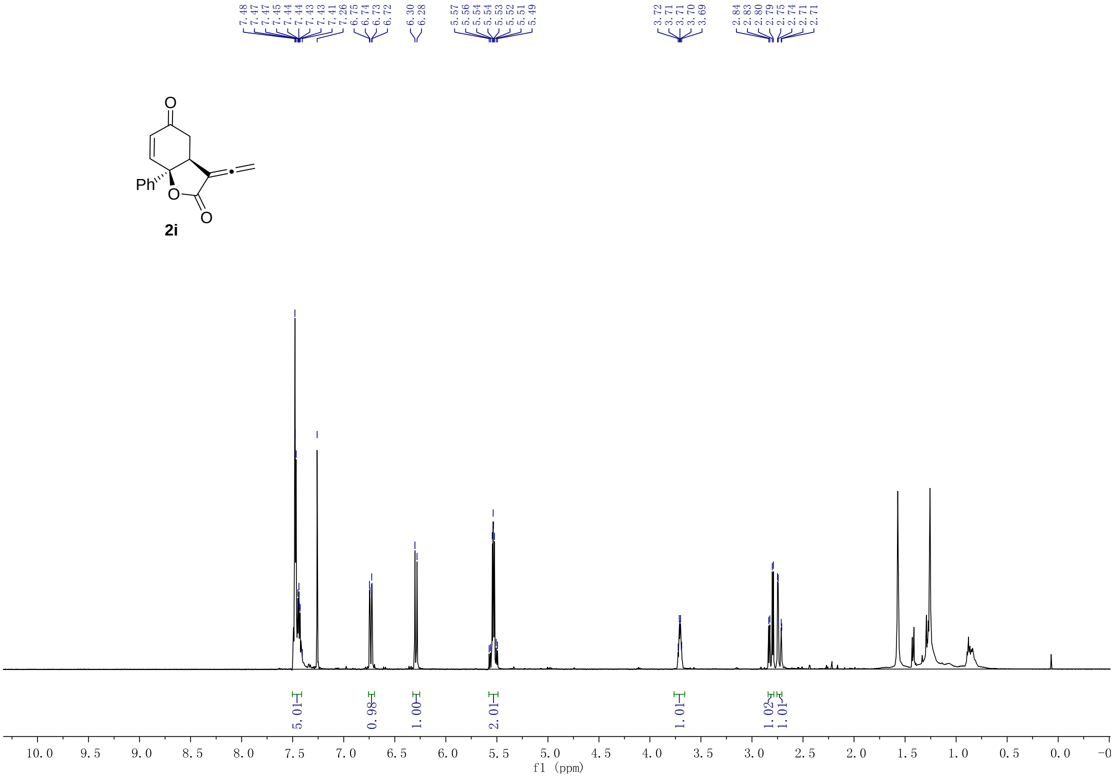

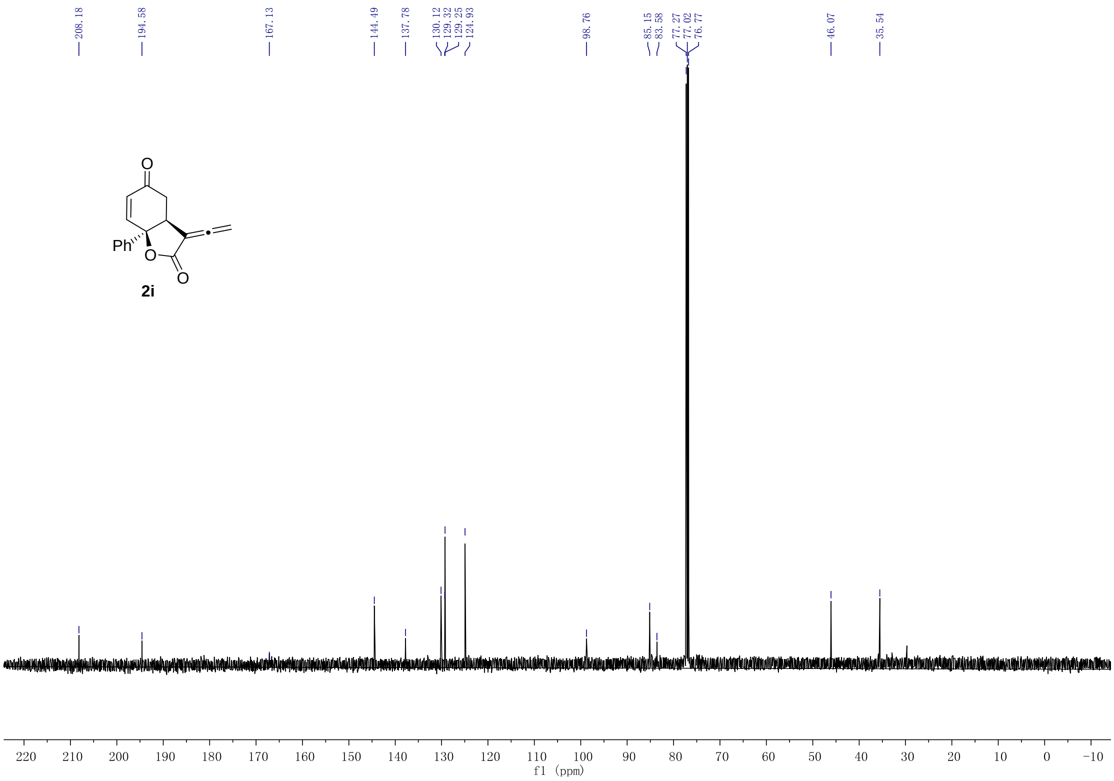

Supplementary Figure 34. ^{1}H and ^{13}C NMR spectra for compound **2i**

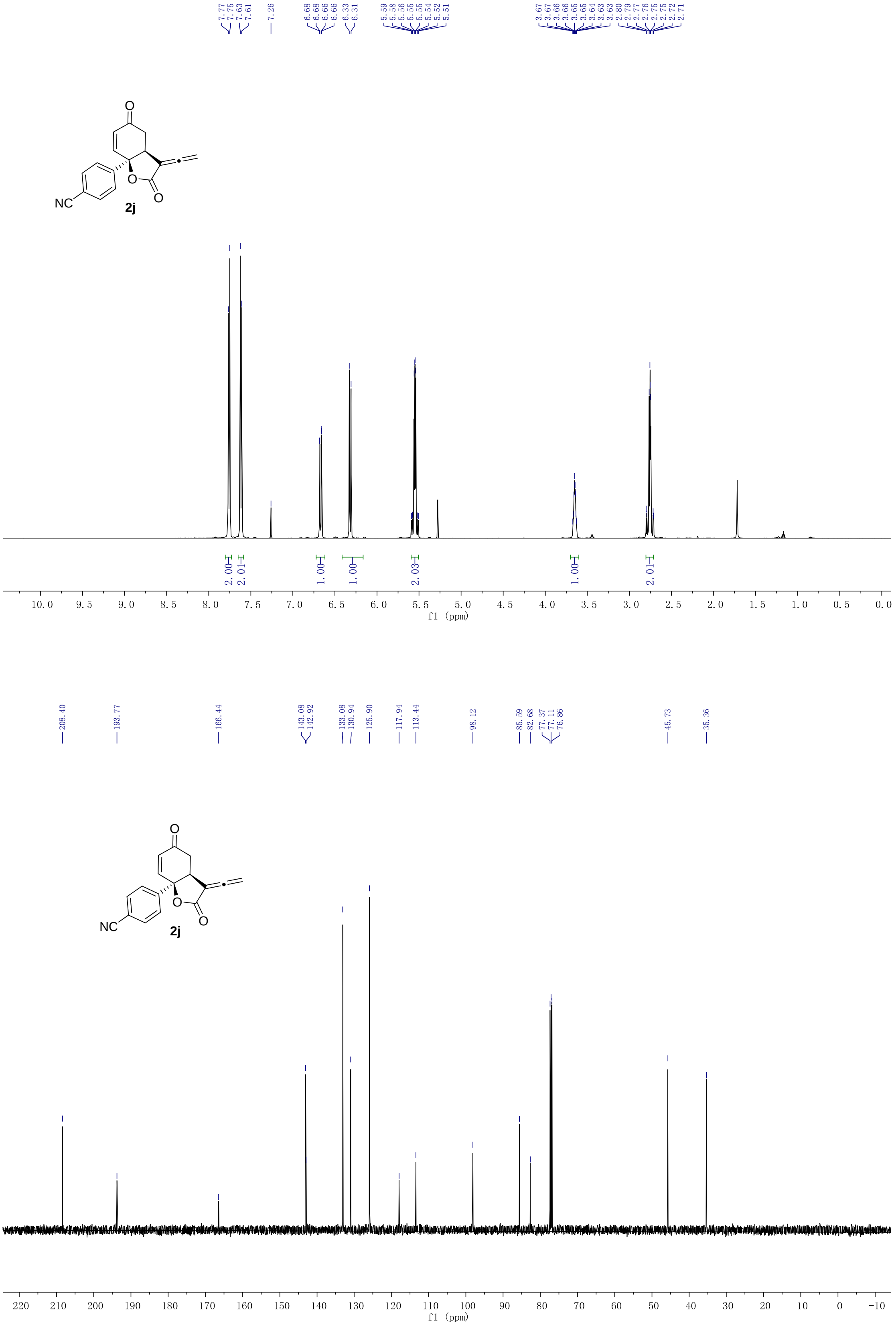

Supplementary Figure 35. ^{1}H and ^{13}C NMR spectra for compound **2j**

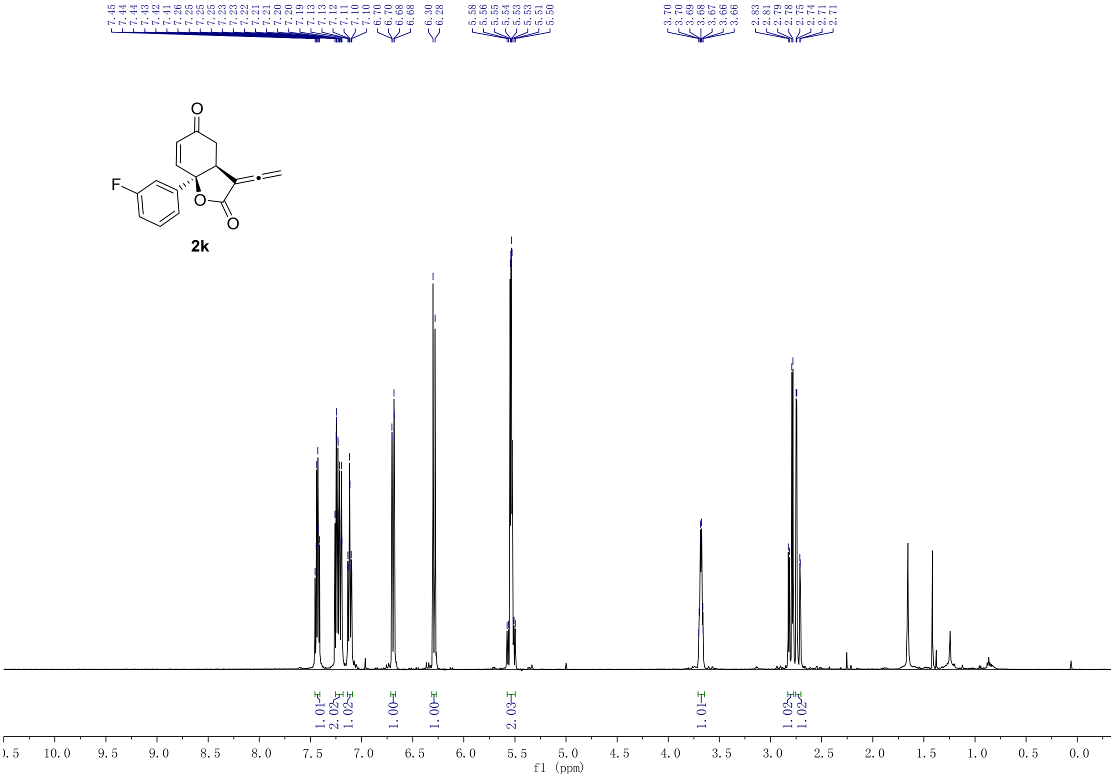

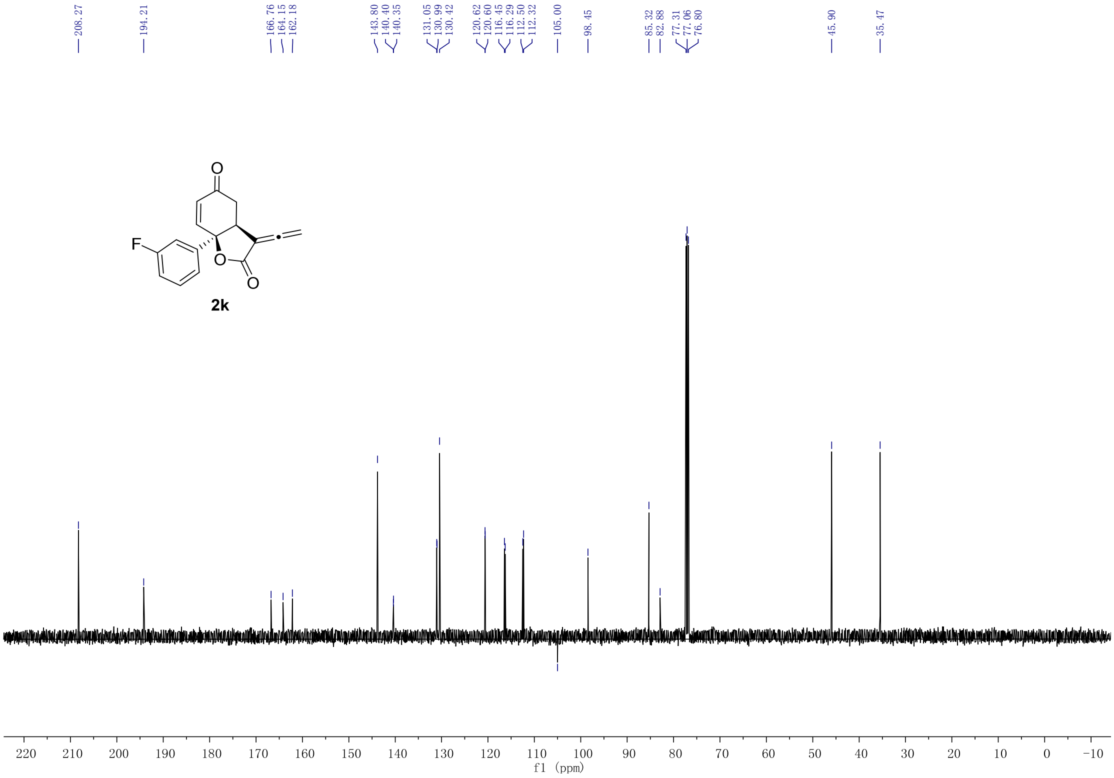

Supplementary Figure 36. ^{1}H and ^{13}C NMR spectra for compound **2k**

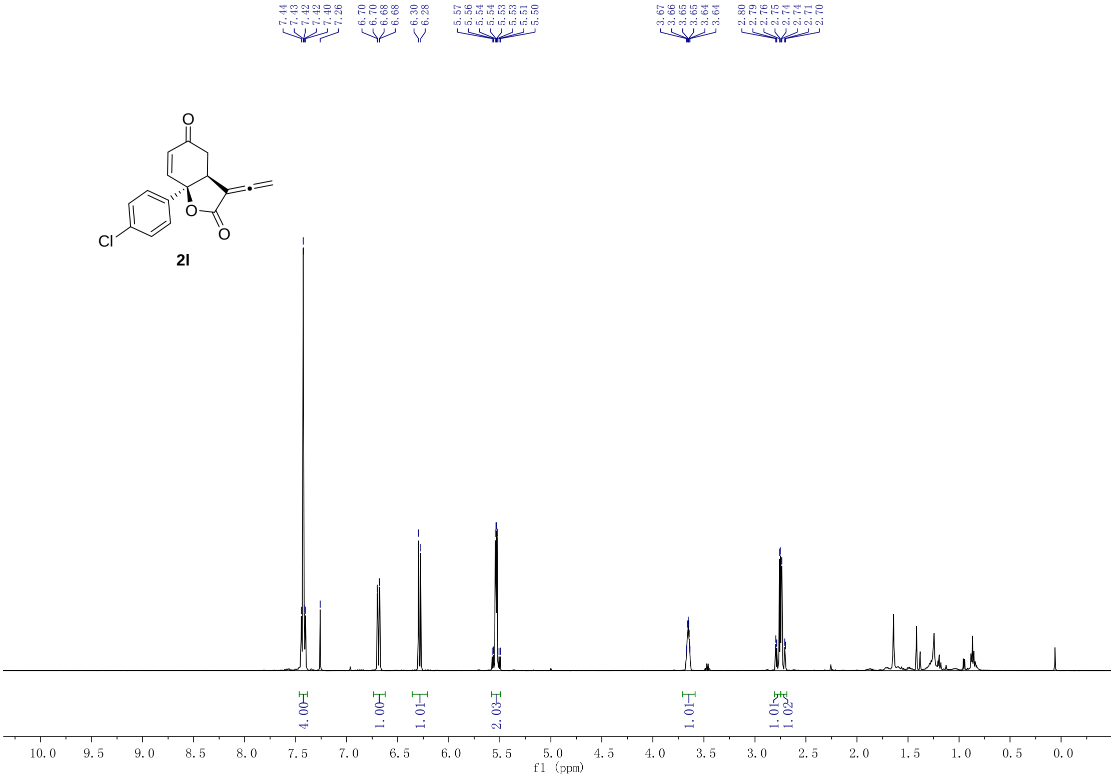

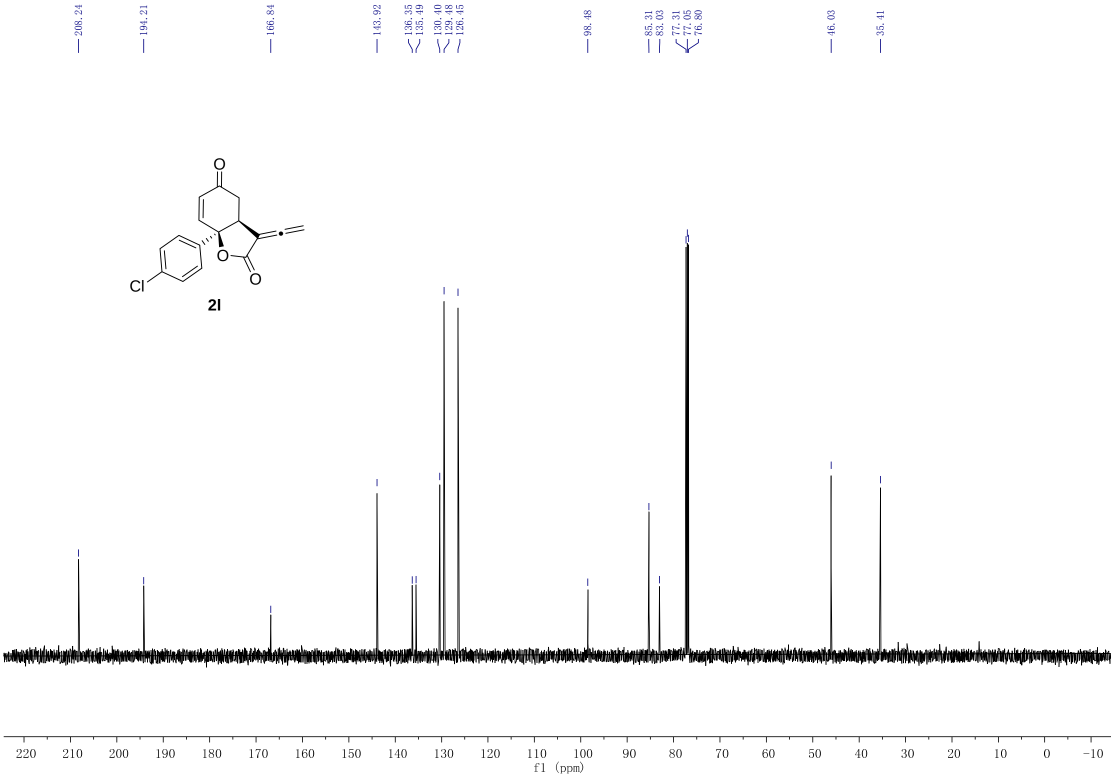

Supplementary Figure 37. ^{1}H and ^{13}C NMR spectra for compound **2l**

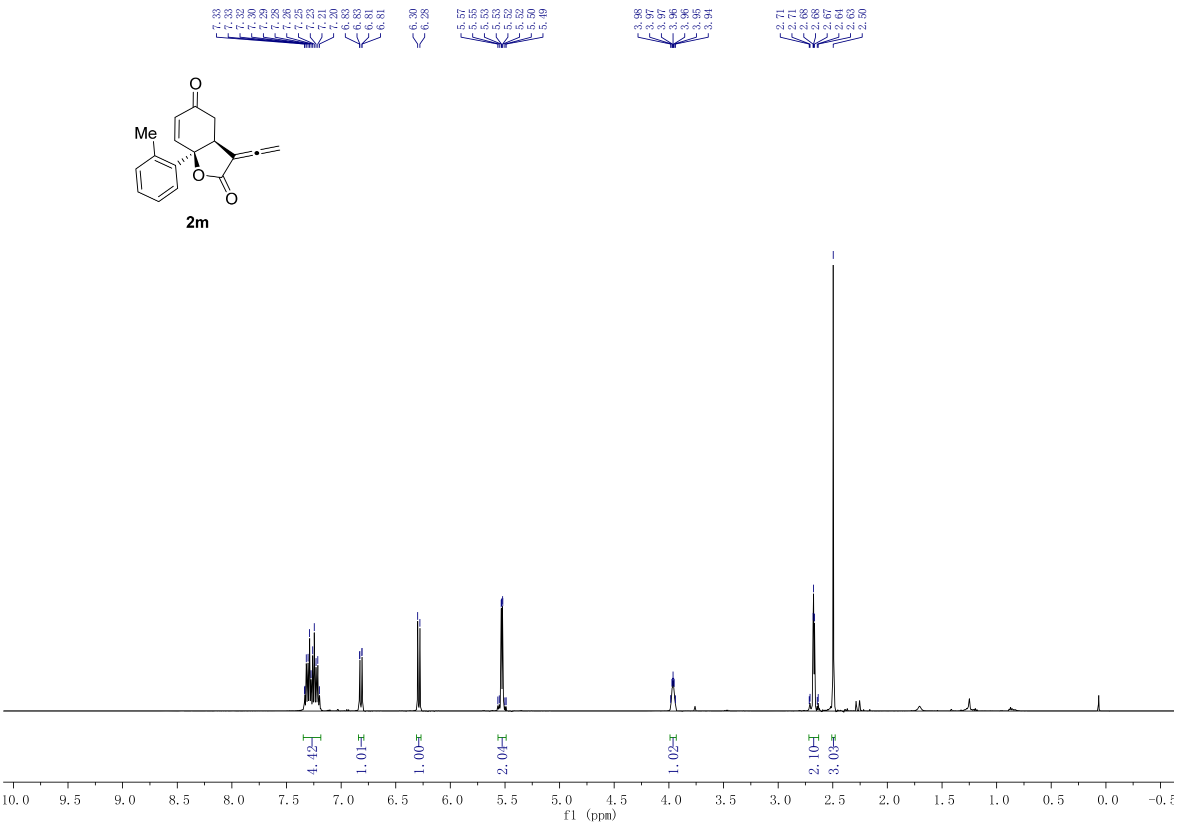

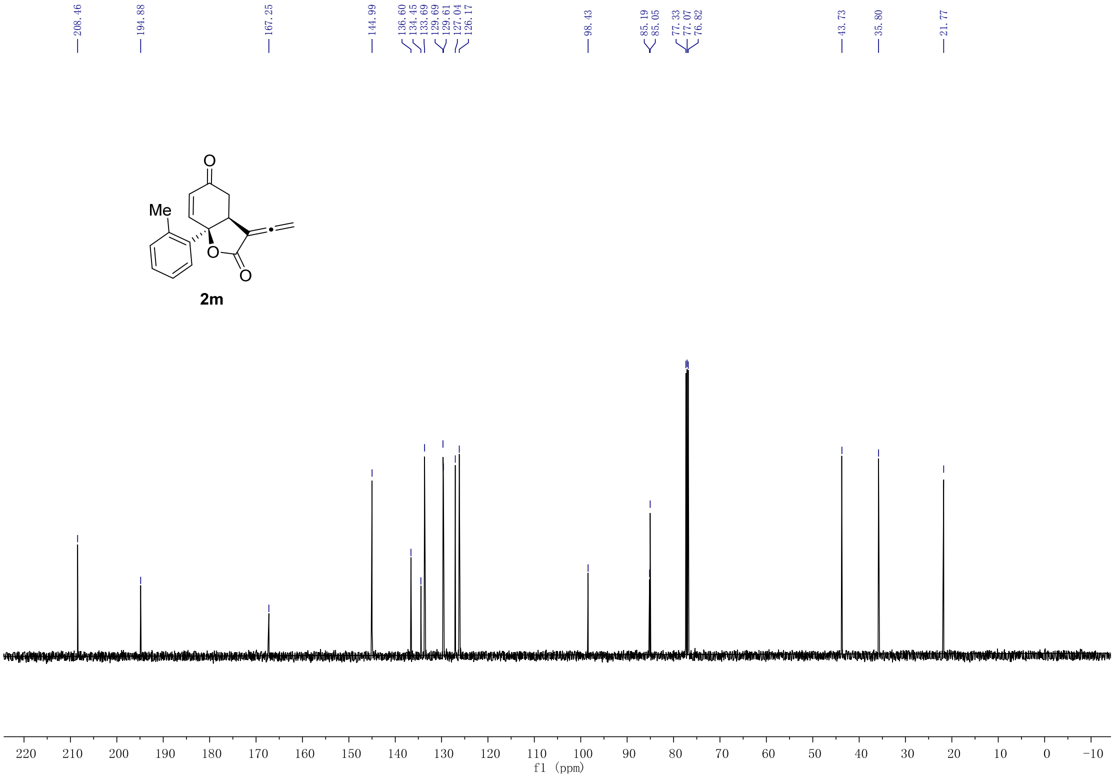

Supplementary Figure 38. ^{1}H and ^{13}C NMR spectra for compound **2m**

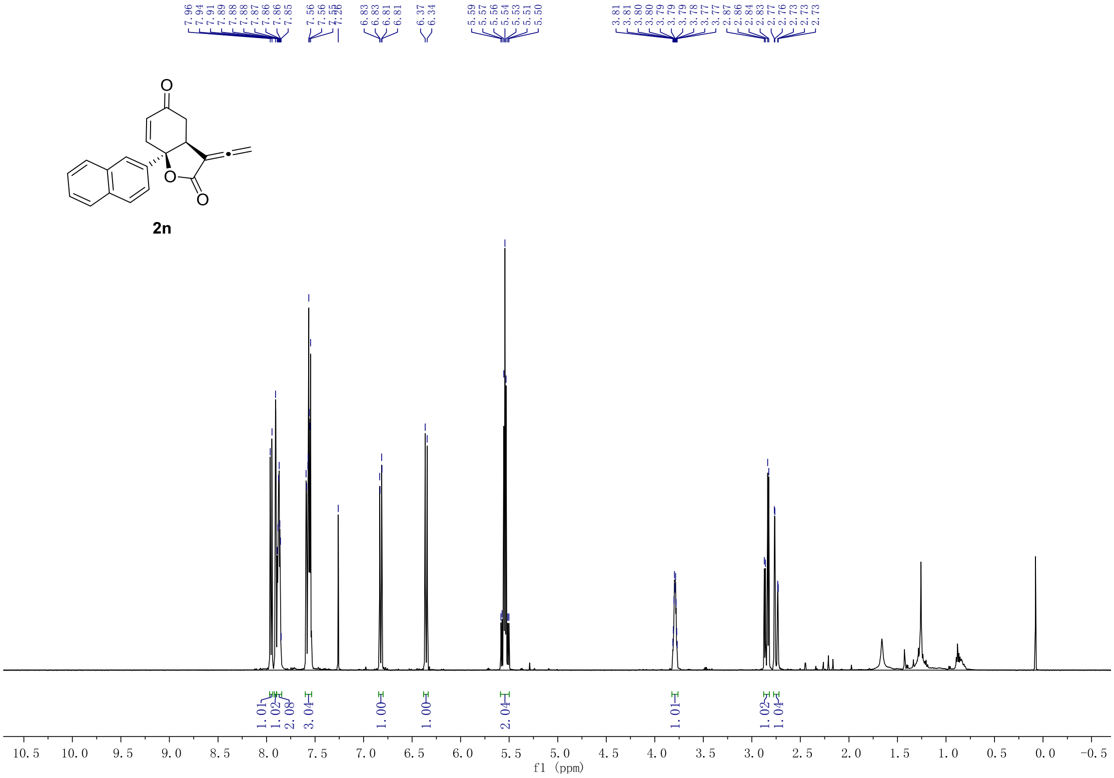

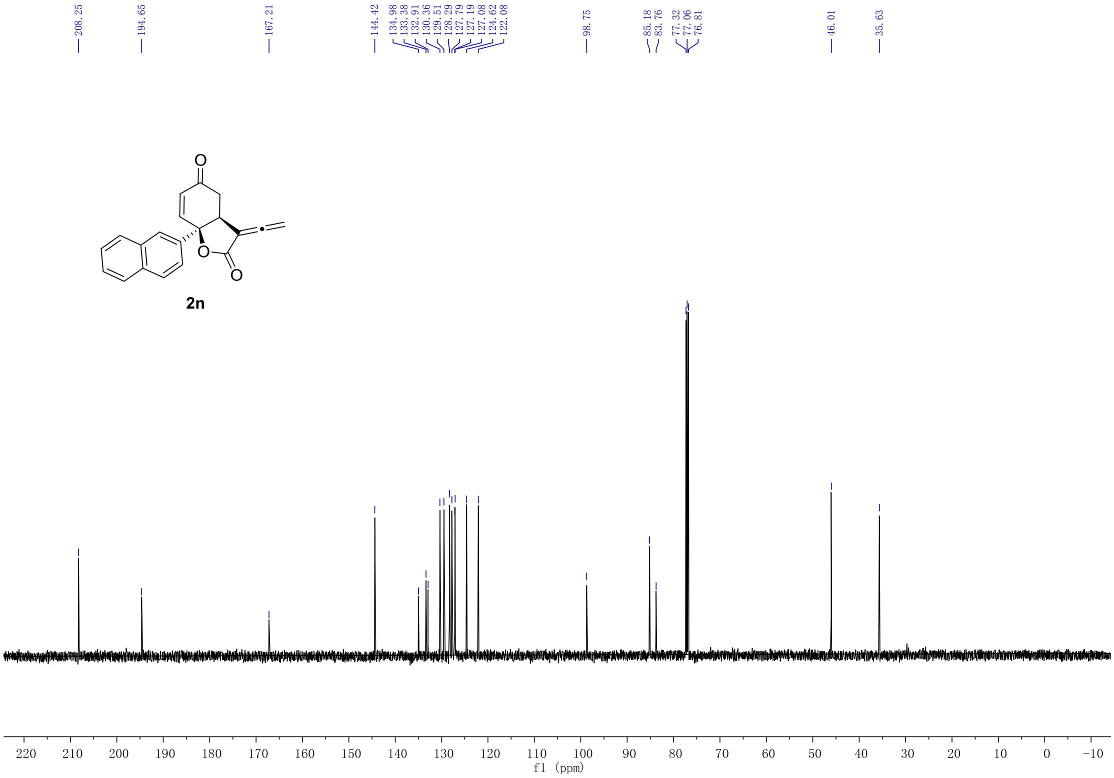

Supplementary Figure 39. ^{1}H and ^{13}C NMR spectra for compound **2n**

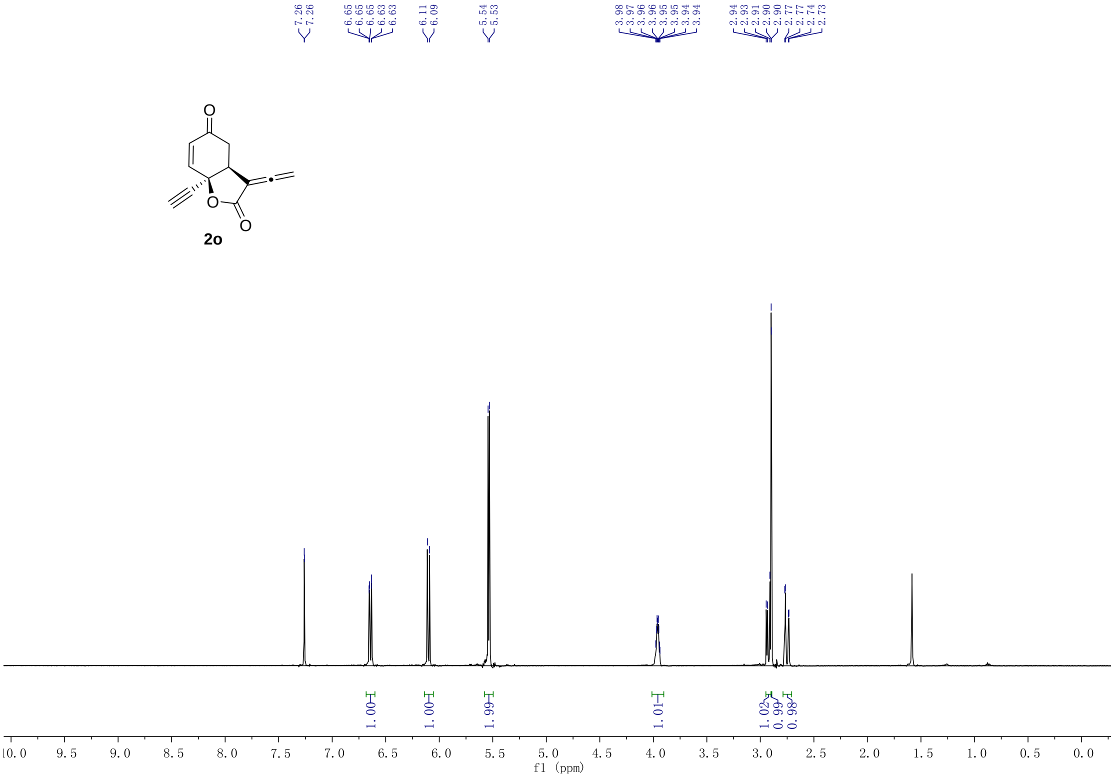

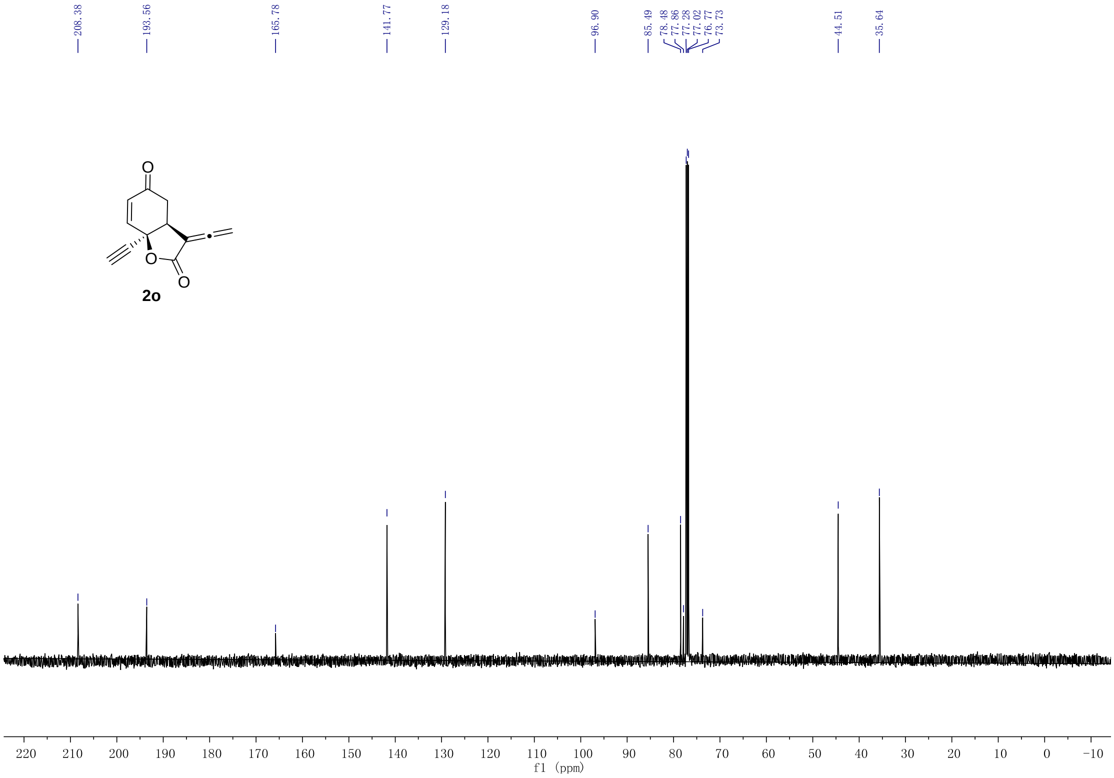

Supplementary Figure 40. ^{1}H and ^{13}C NMR spectra for compound **2o**

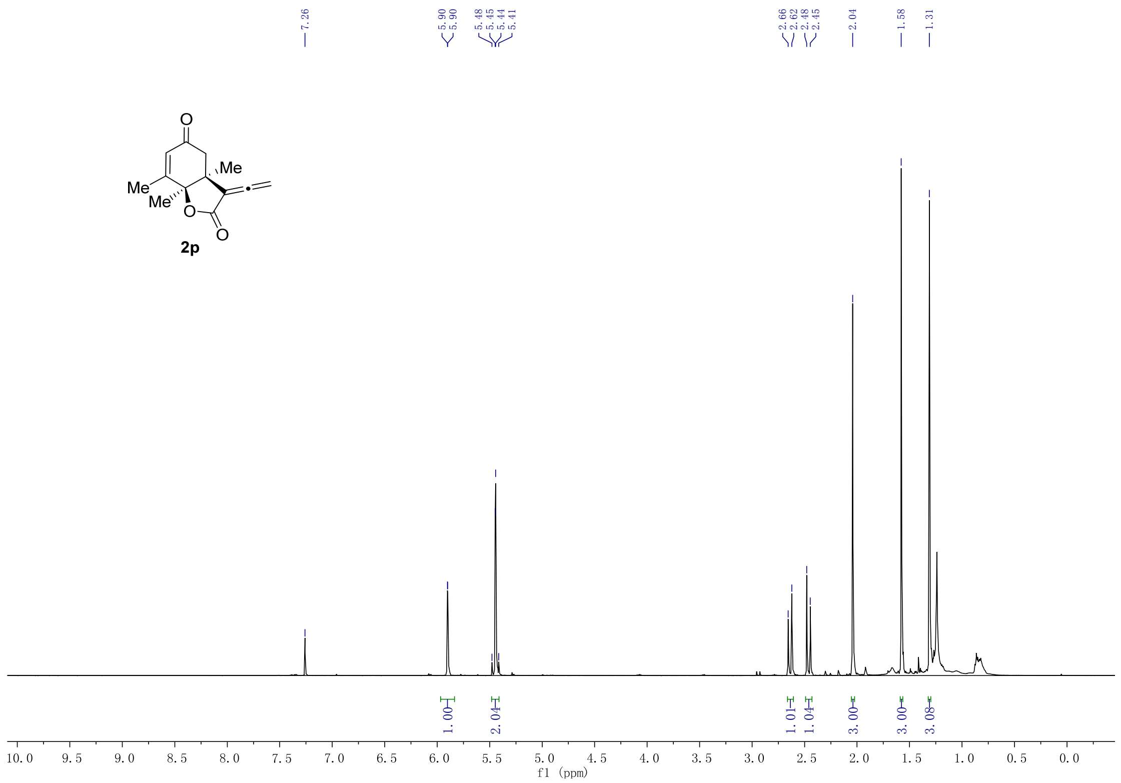

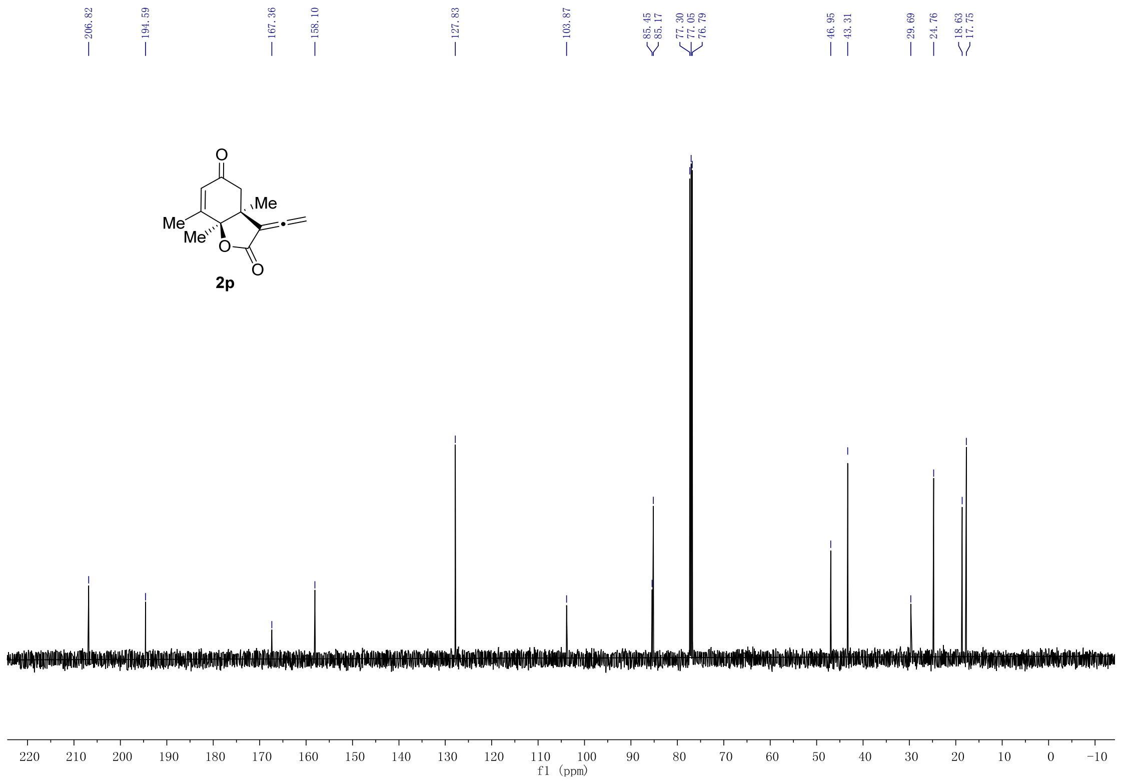

Supplementary Figure 41. ^{1}H and ^{13}C NMR spectra for compound **2p**

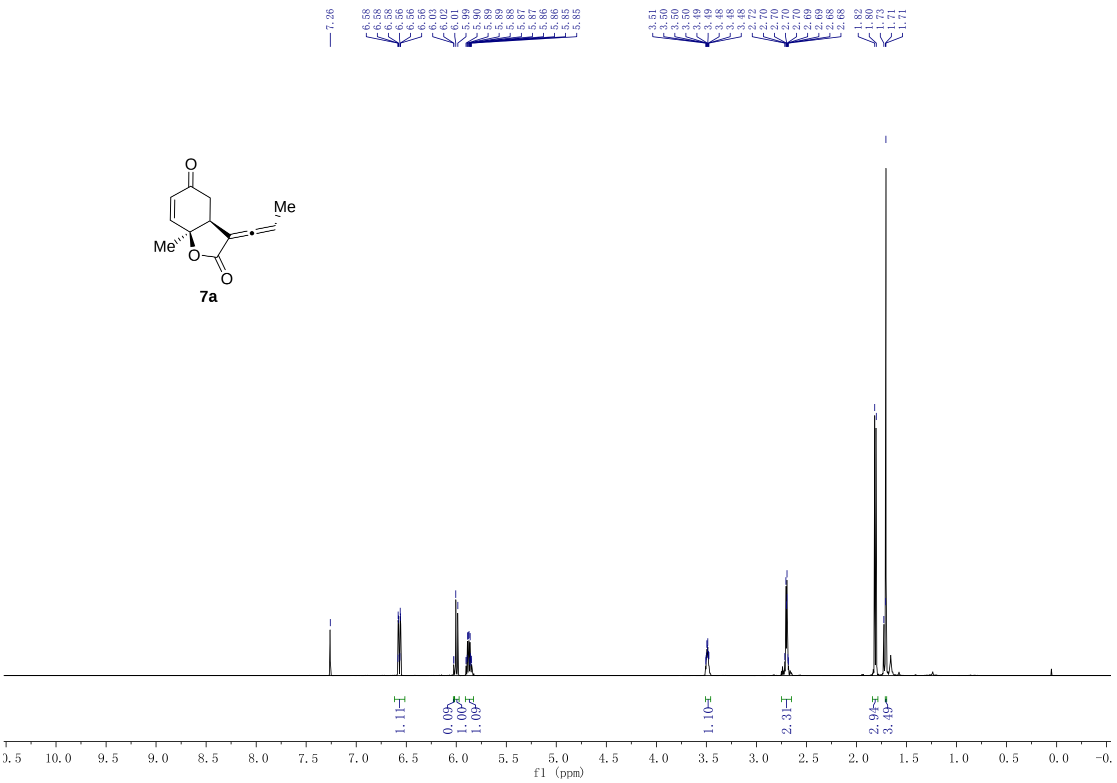

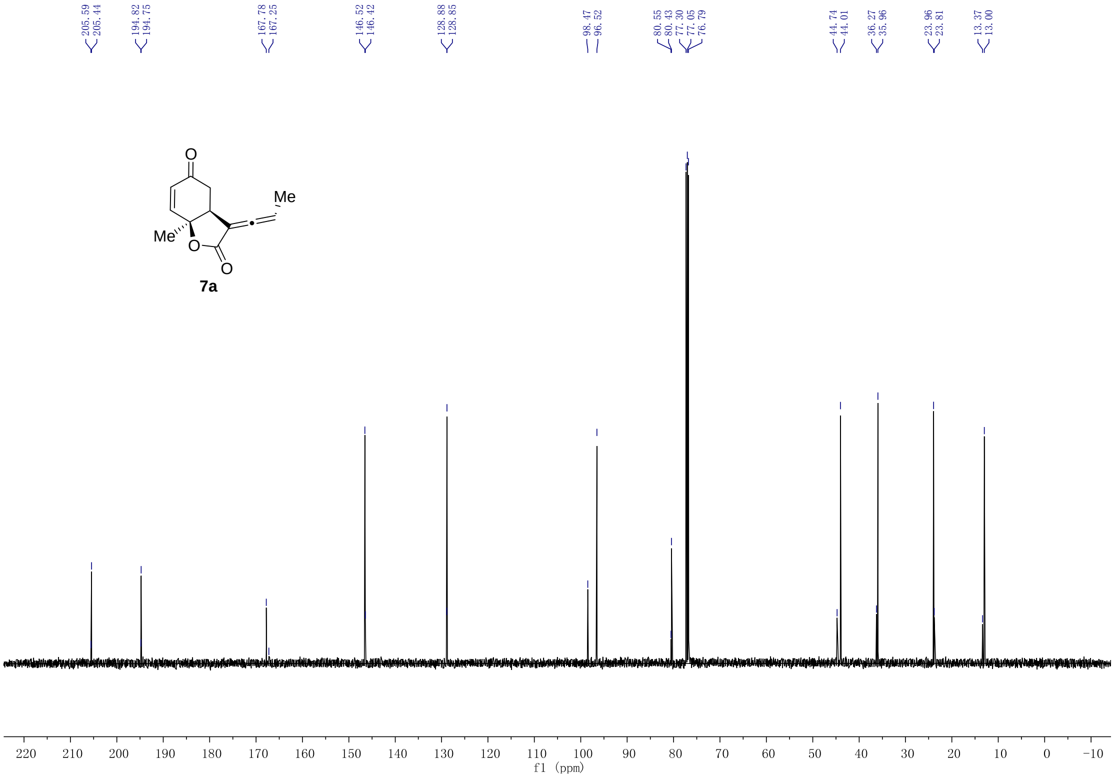

Supplementary Figure 42. ^{1}H and ^{13}C NMR spectra for compound **7a**

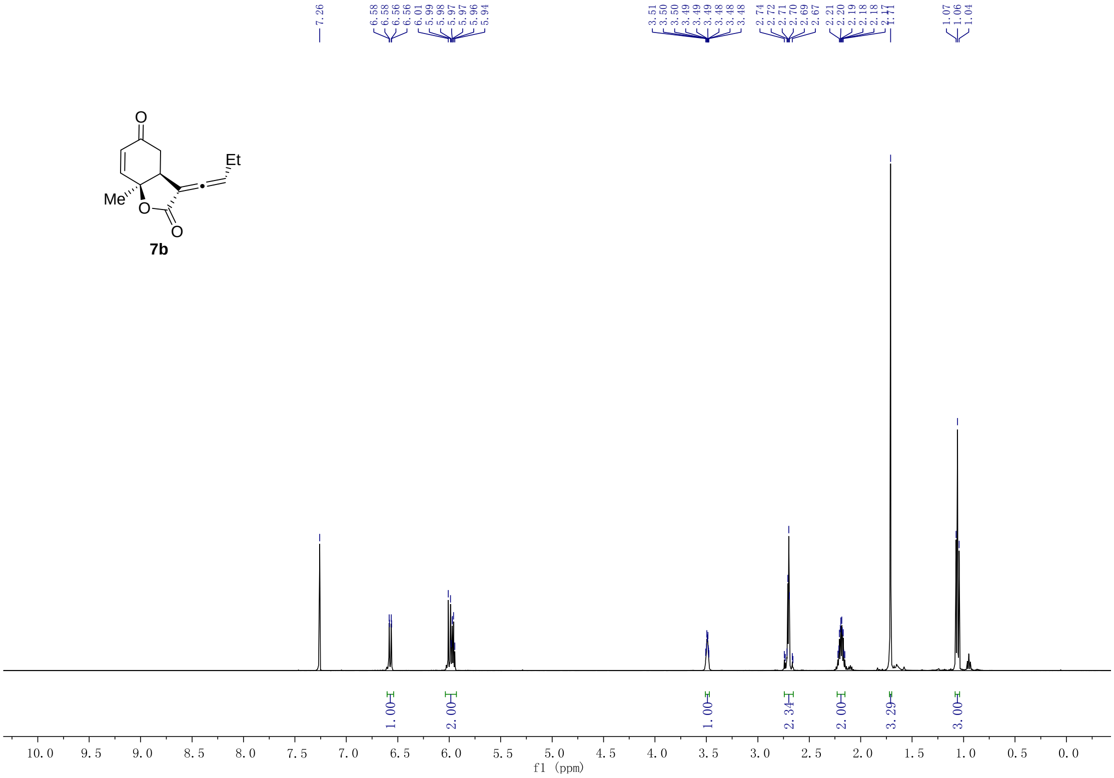

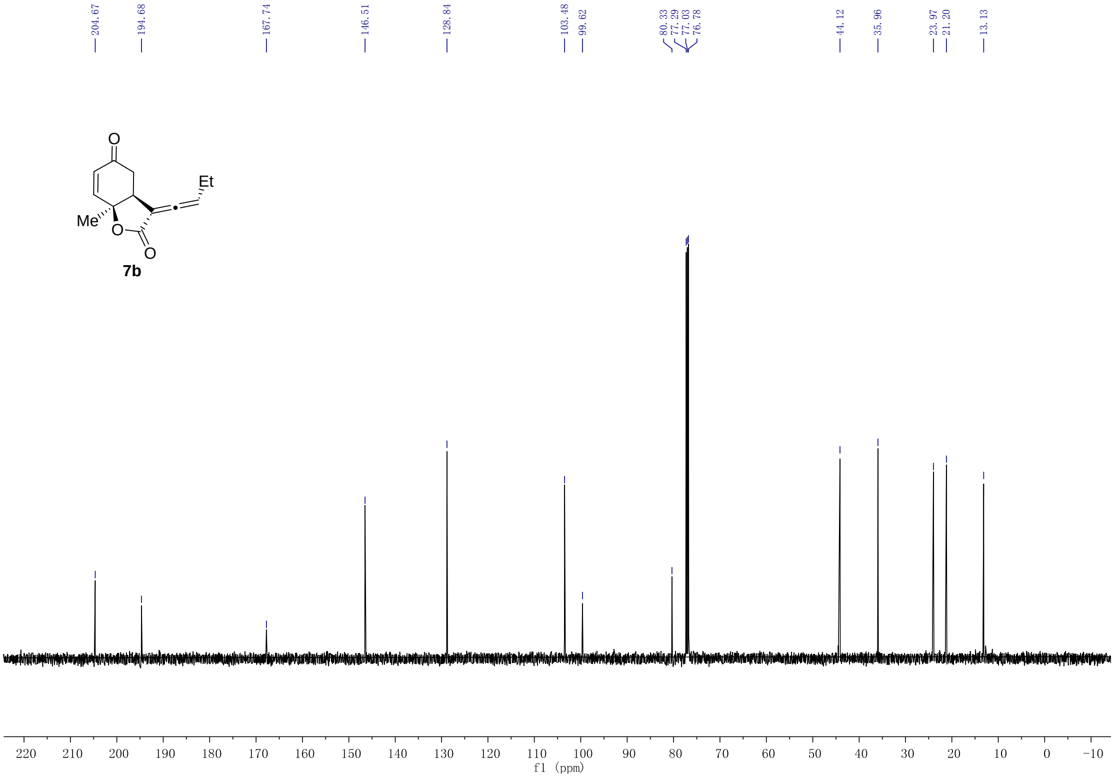

Supplementary Figure 43. ^{1}H and ^{13}C NMR spectra for compound **7b**

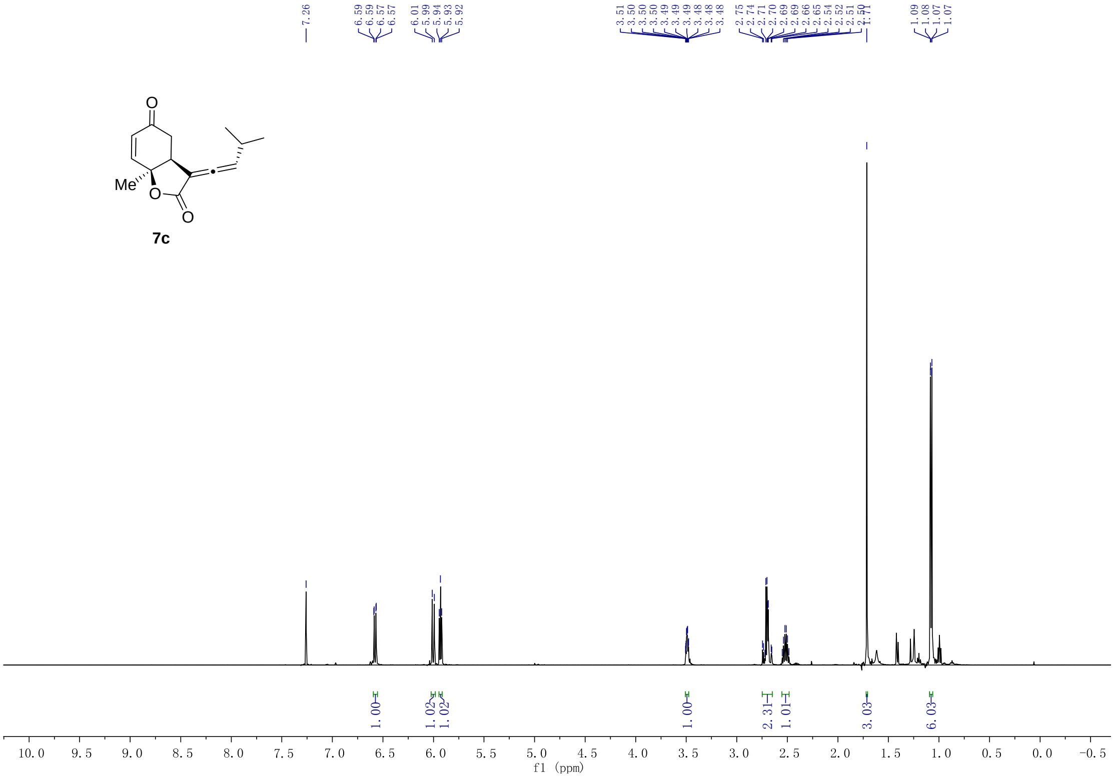

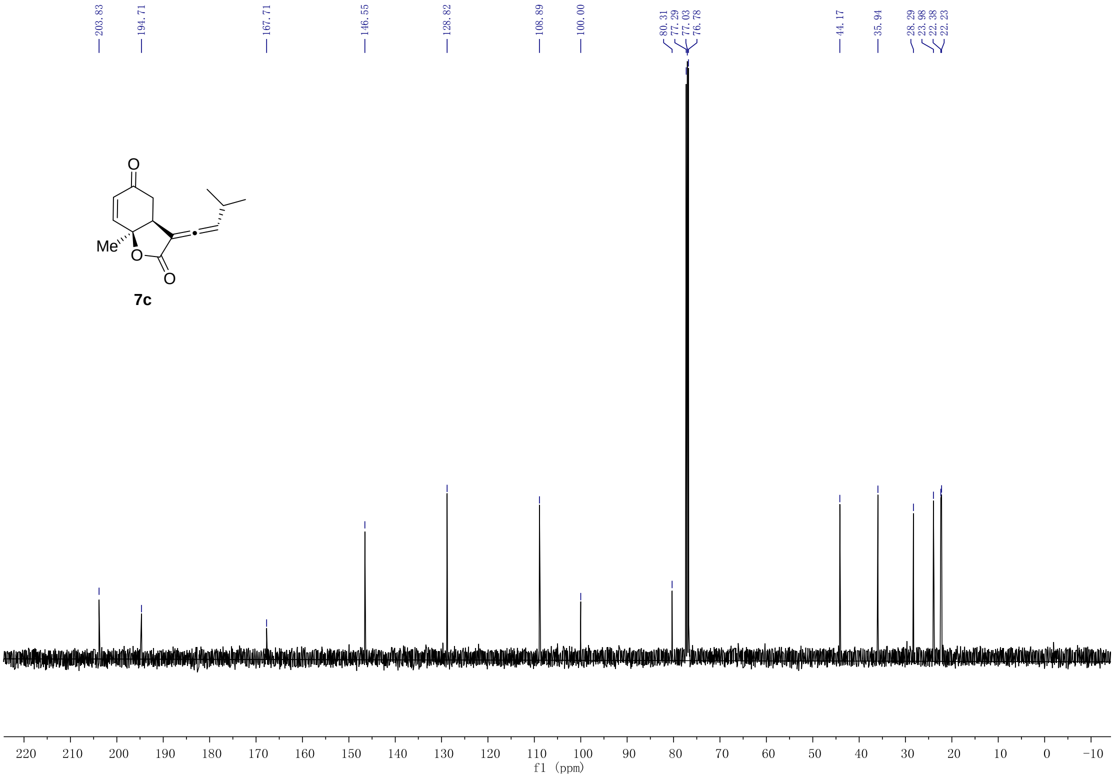

Supplementary Figure 44. ^{1}H and ^{13}C NMR spectra for compound **7c**

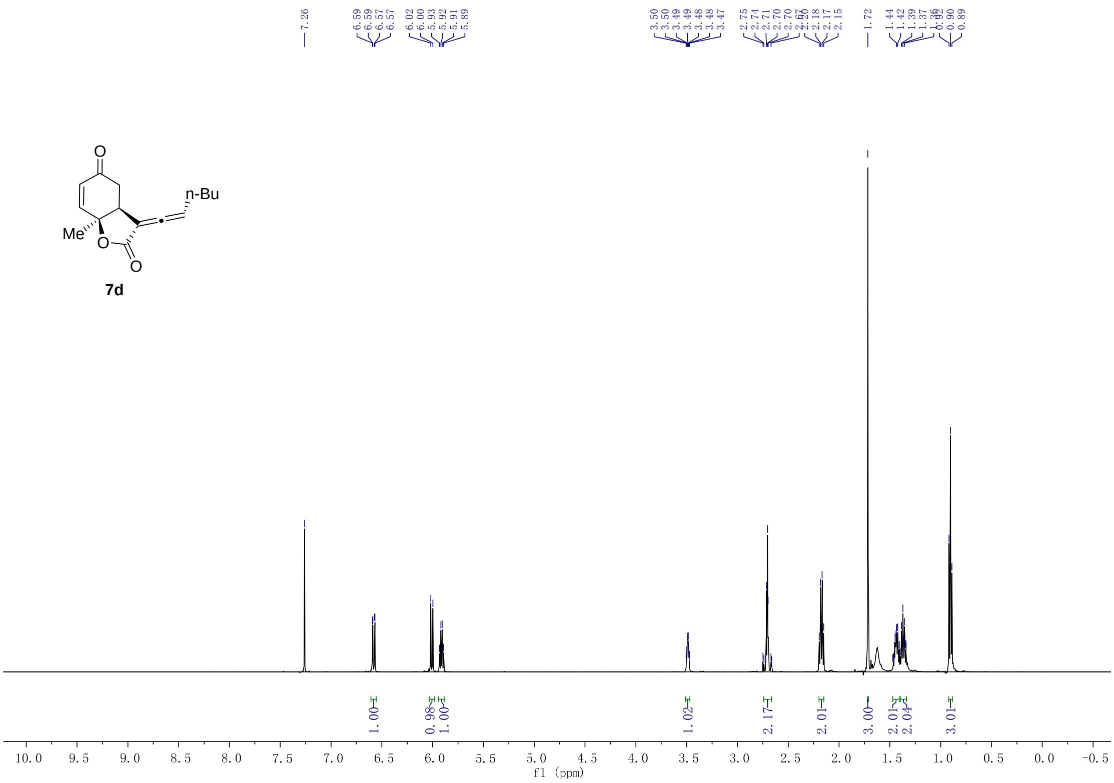

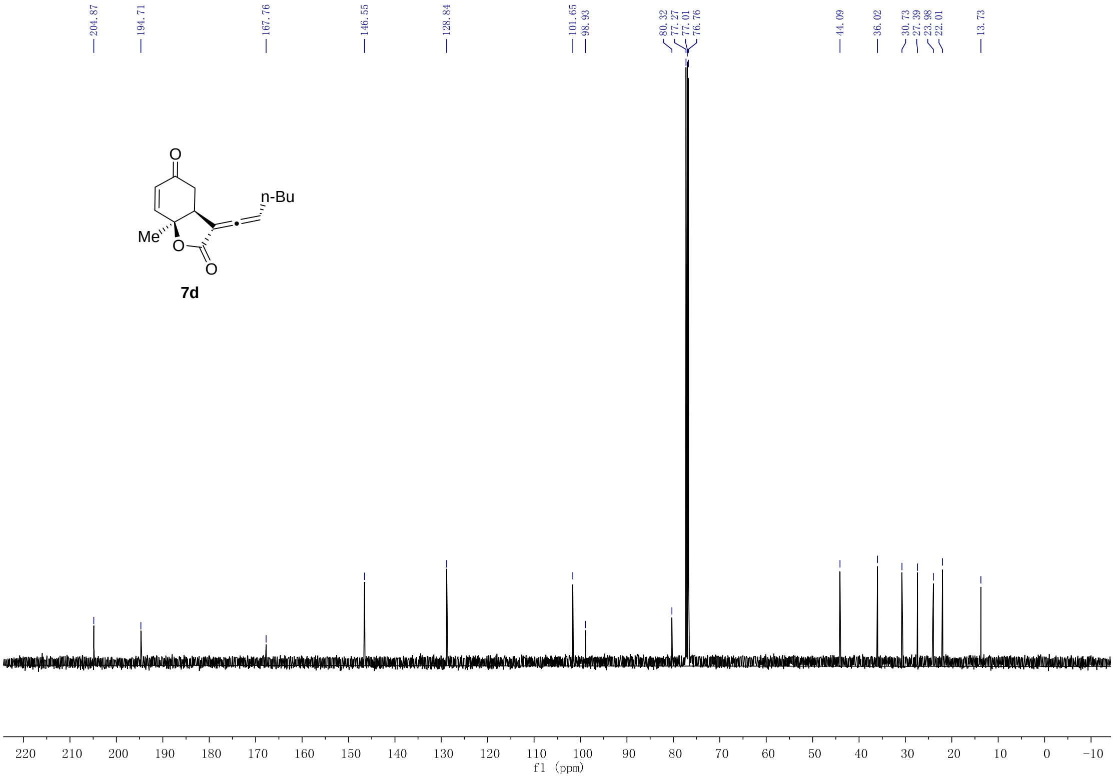

Supplementary Figure 45. ^{1}H and ^{13}C NMR spectra for compound **7d**

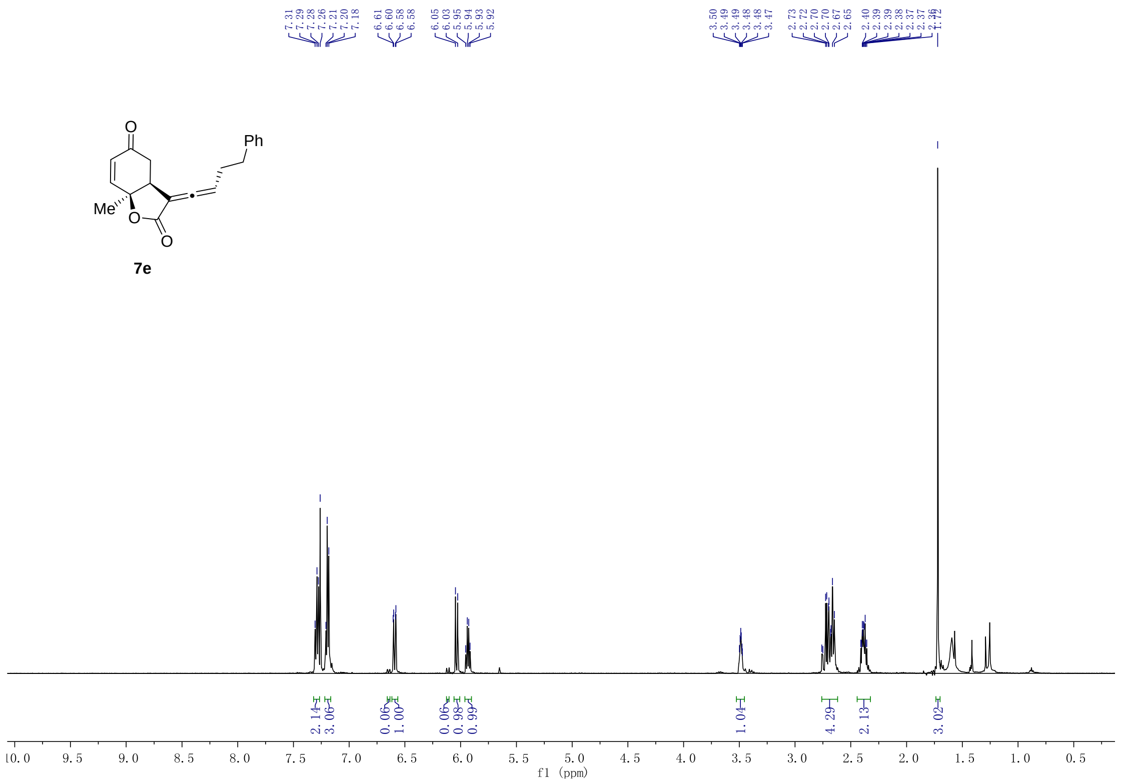

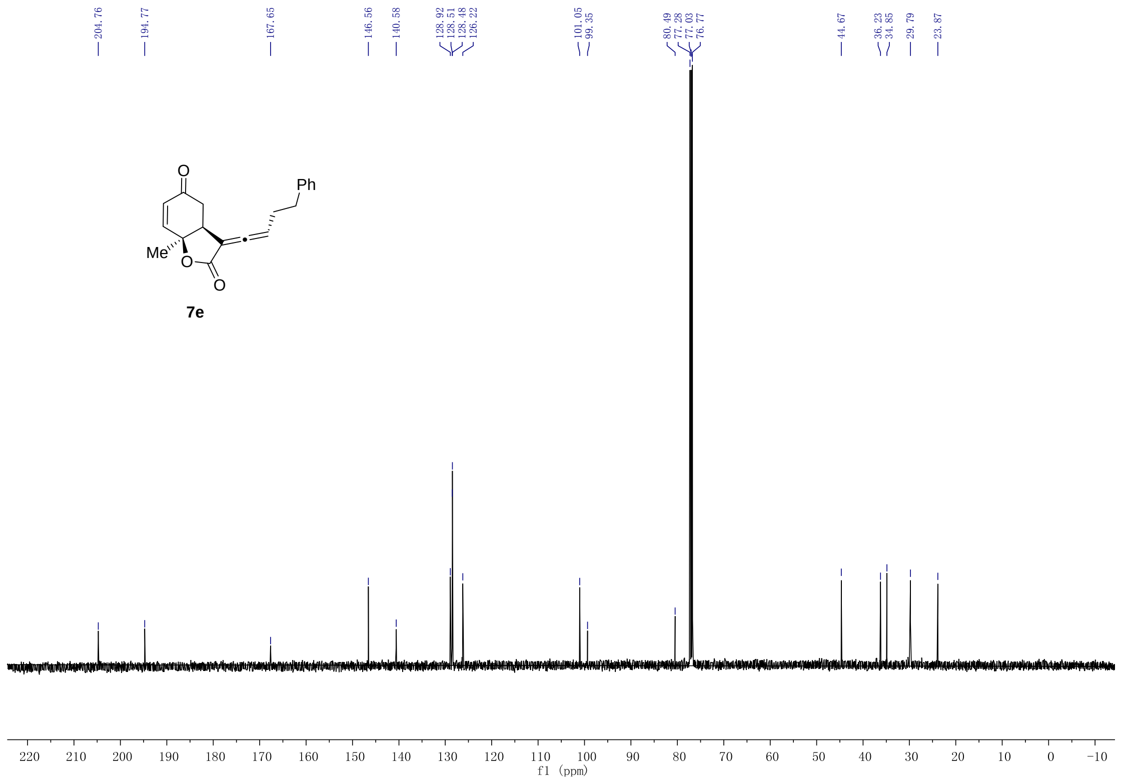

Supplementary Figure 46. ^{1}H and ^{13}C NMR spectra for compound **7e**

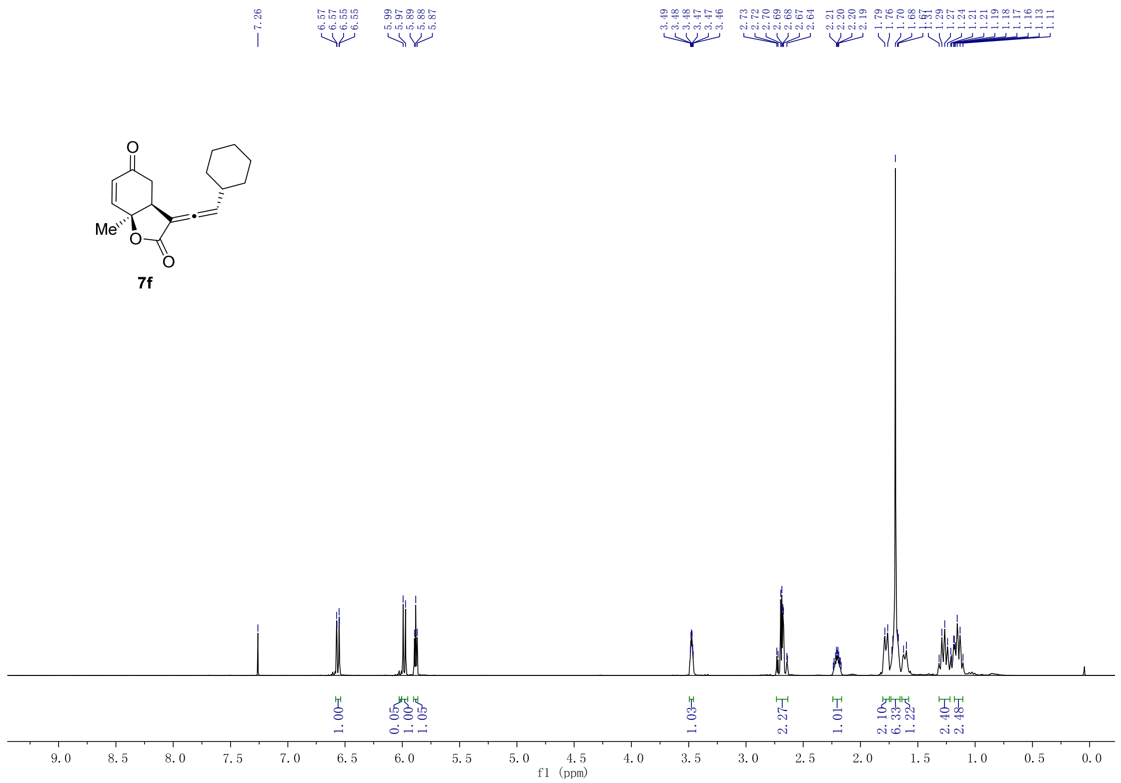

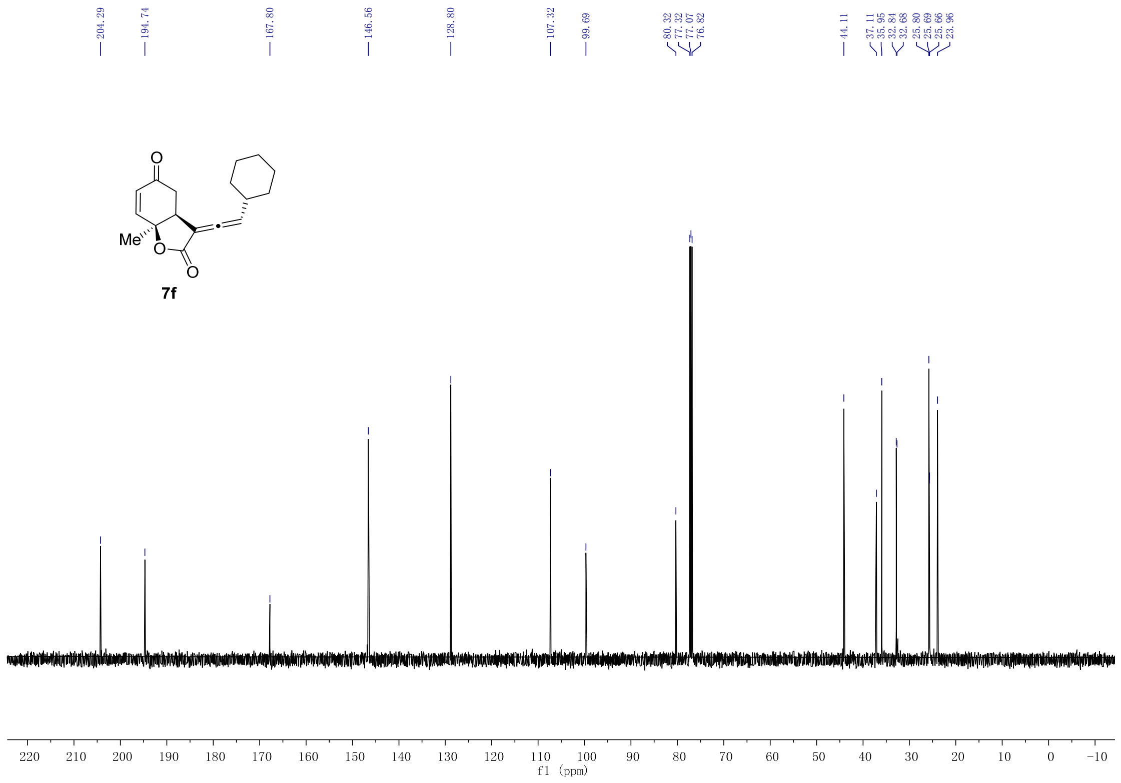

Supplementary Figure 47. ^{1}H and ^{13}C NMR spectra for compound **7f**

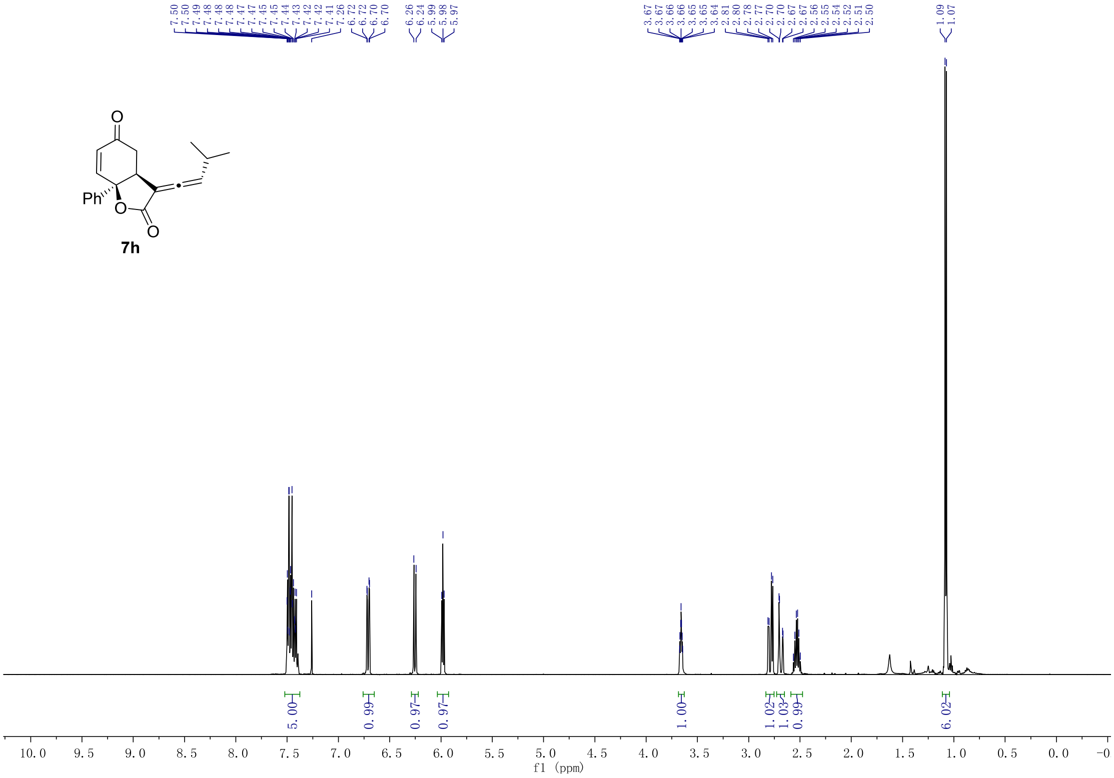

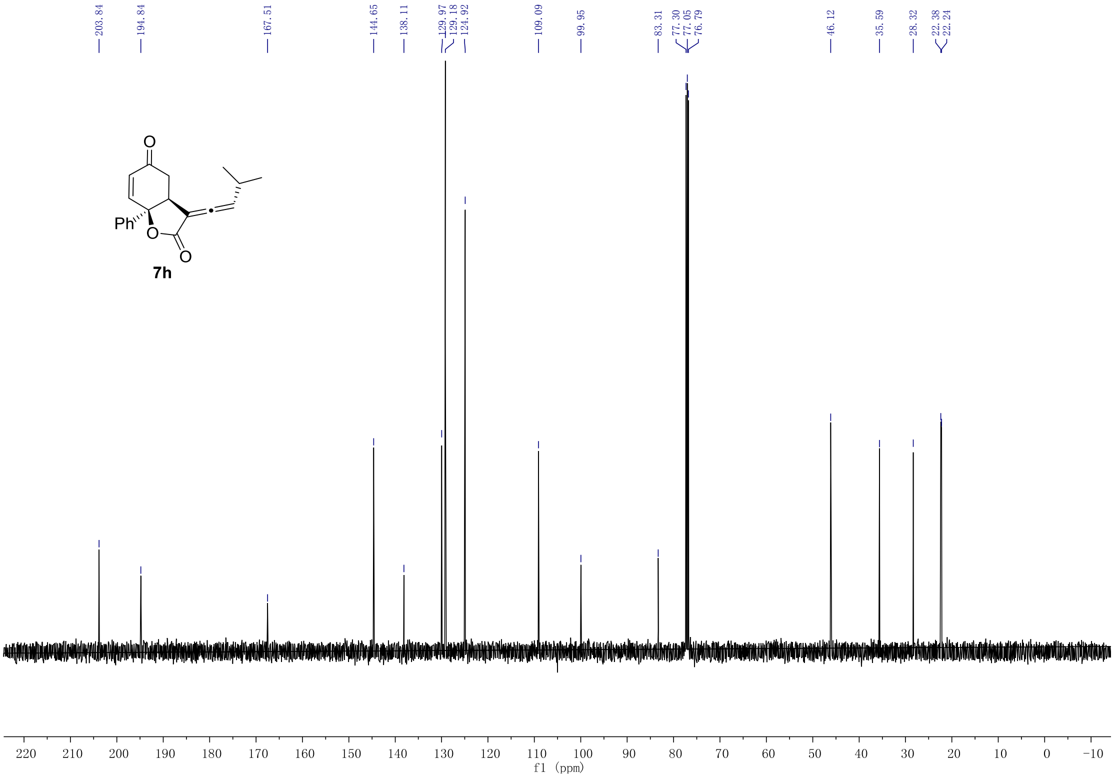

Supplementary Figure 48. ^{1}H and ^{13}C NMR spectra for compound **7h**

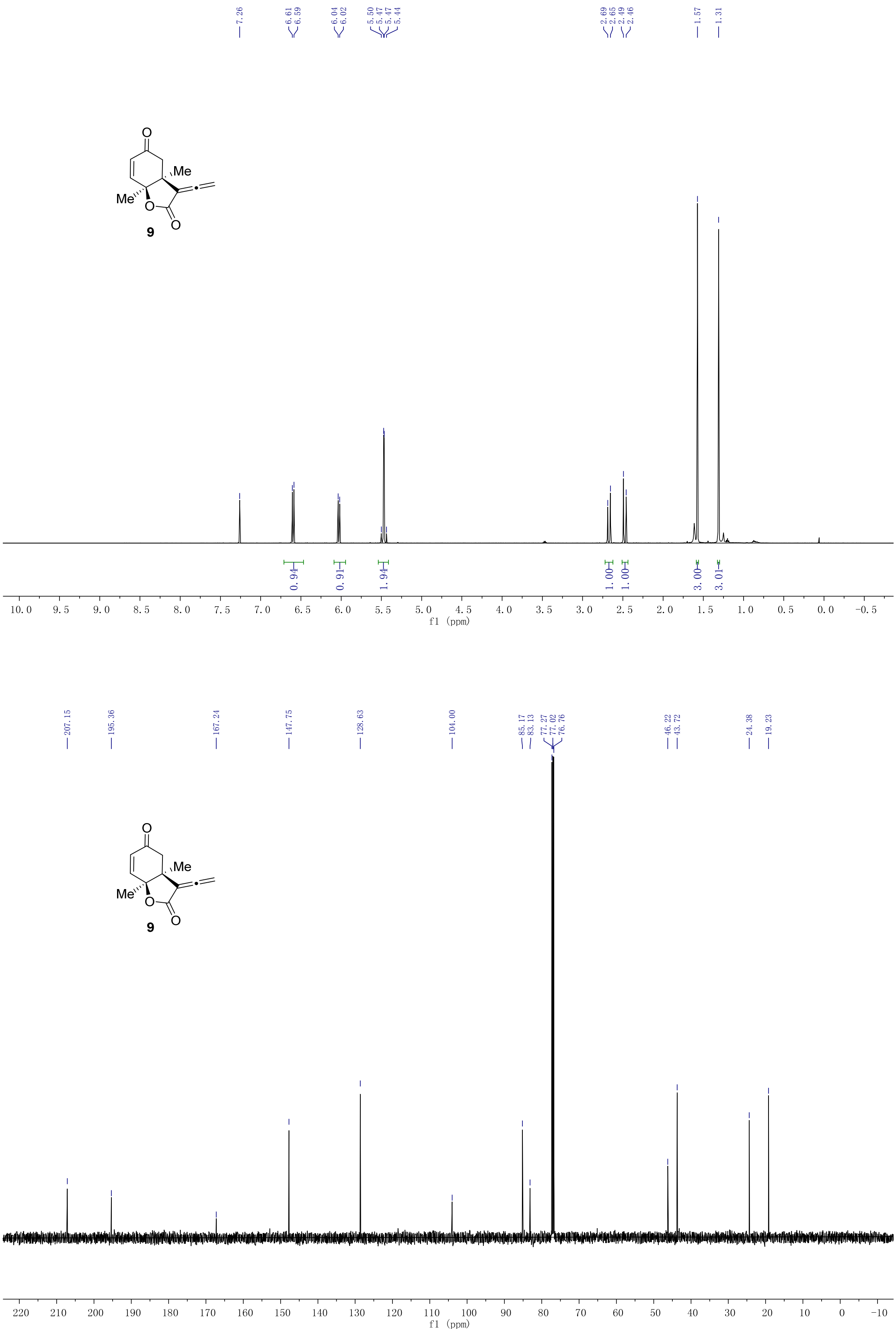

Supplementary Figure 49. ^{1}H and ^{13}C NMR spectra for compound **9**

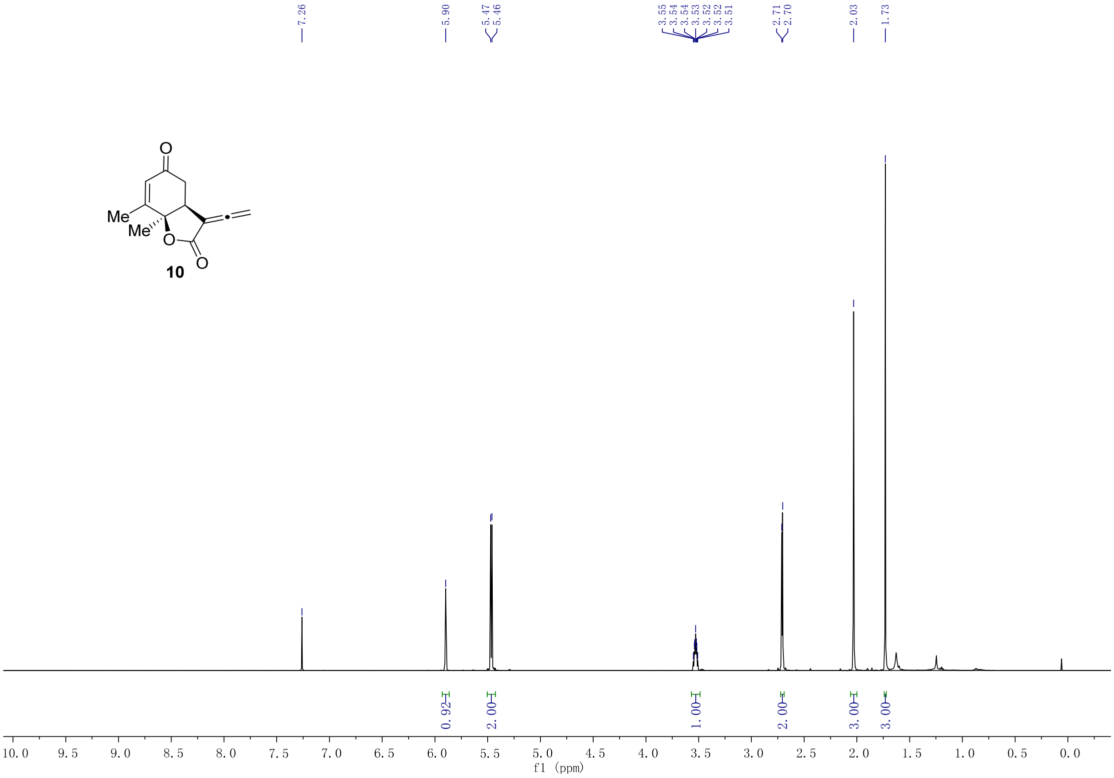

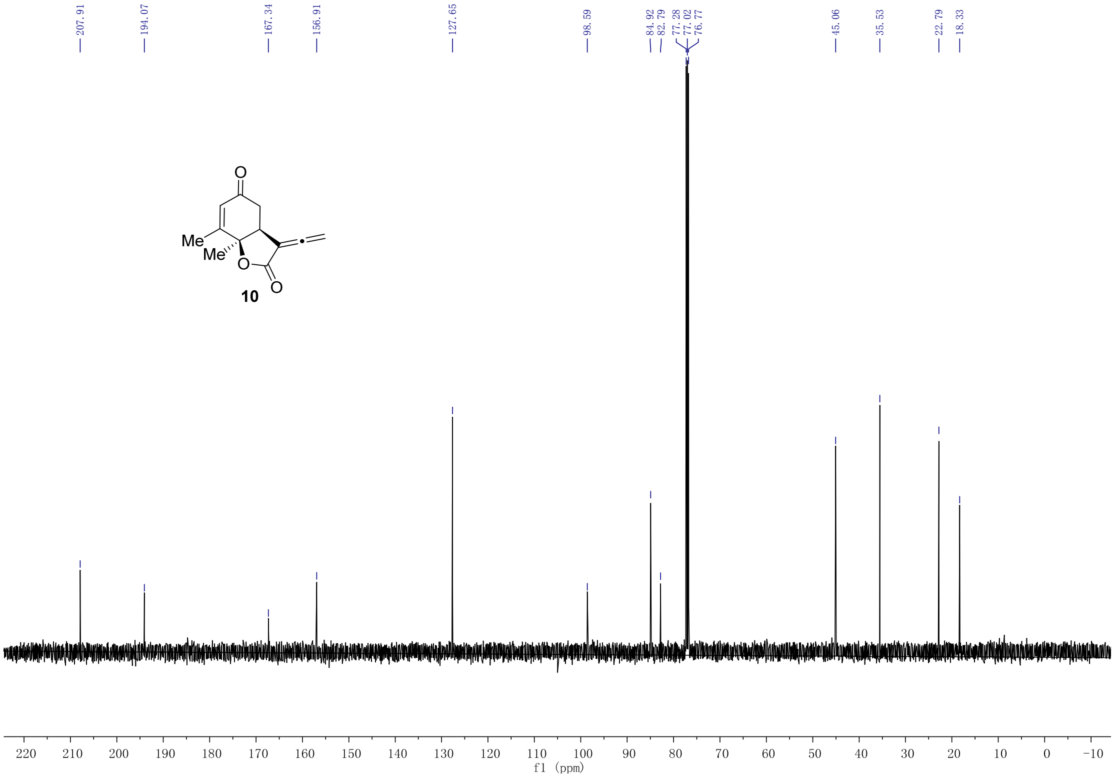

Supplementary Figure 50. ^{1}H and ^{13}C NMR spectra for compound **10**

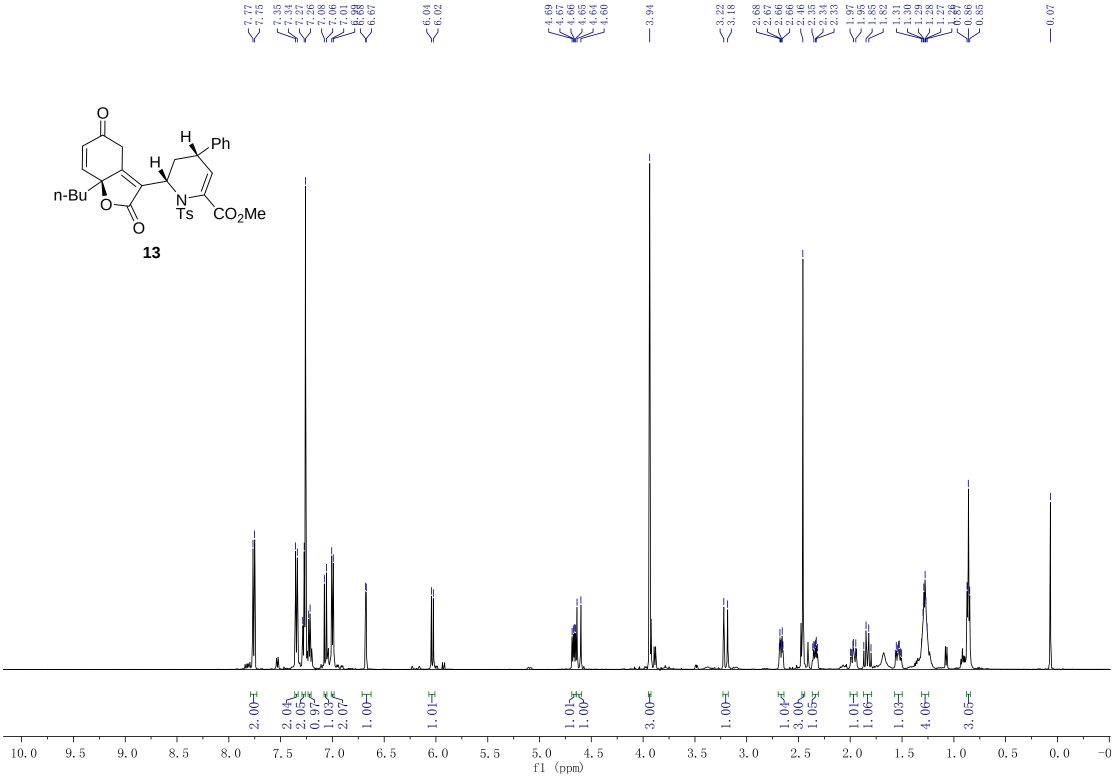

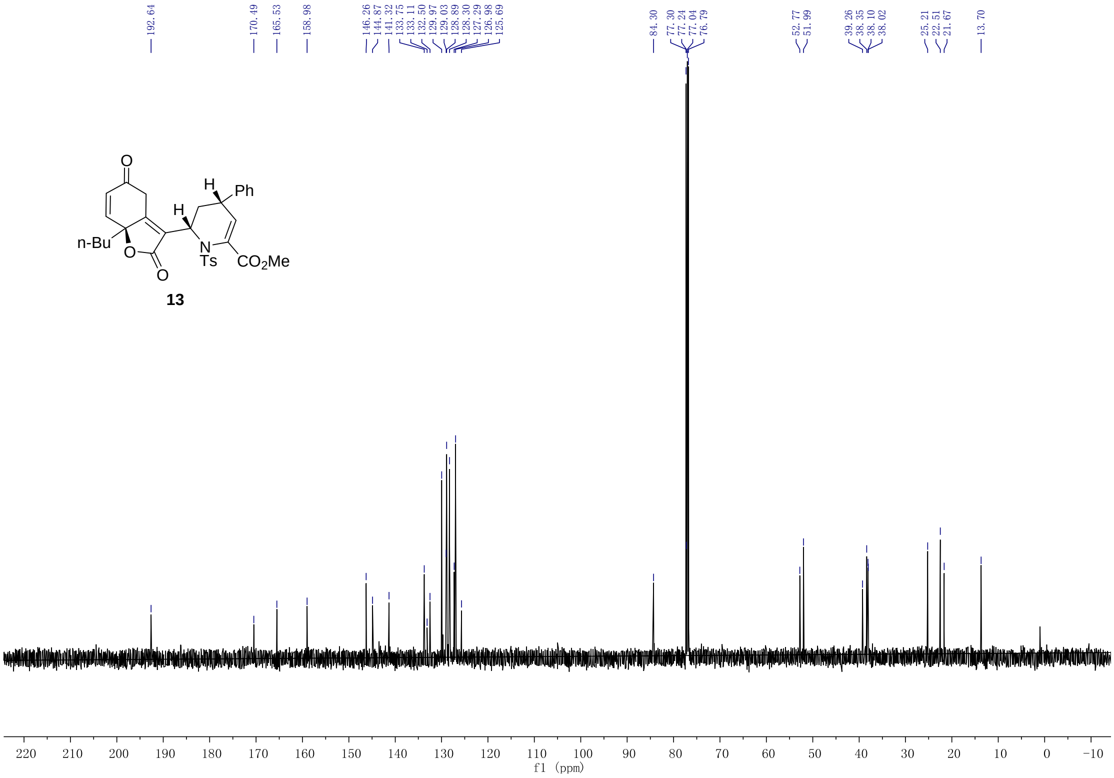

Supplementary Figure 51. ^{1}H and ^{13}C NMR spectra for compound **13**

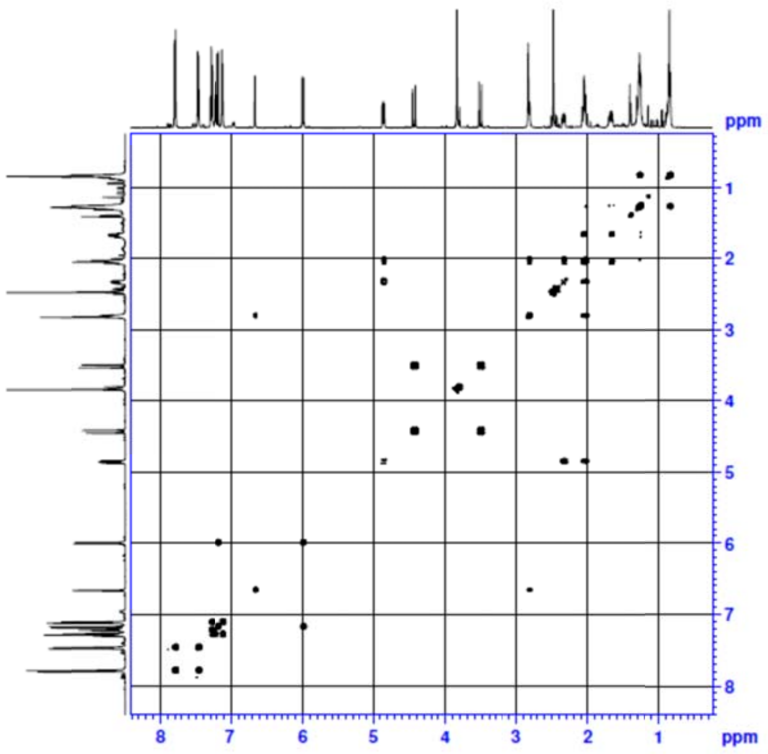

Supplementary Figure 52. COSY spectra for compound **13**

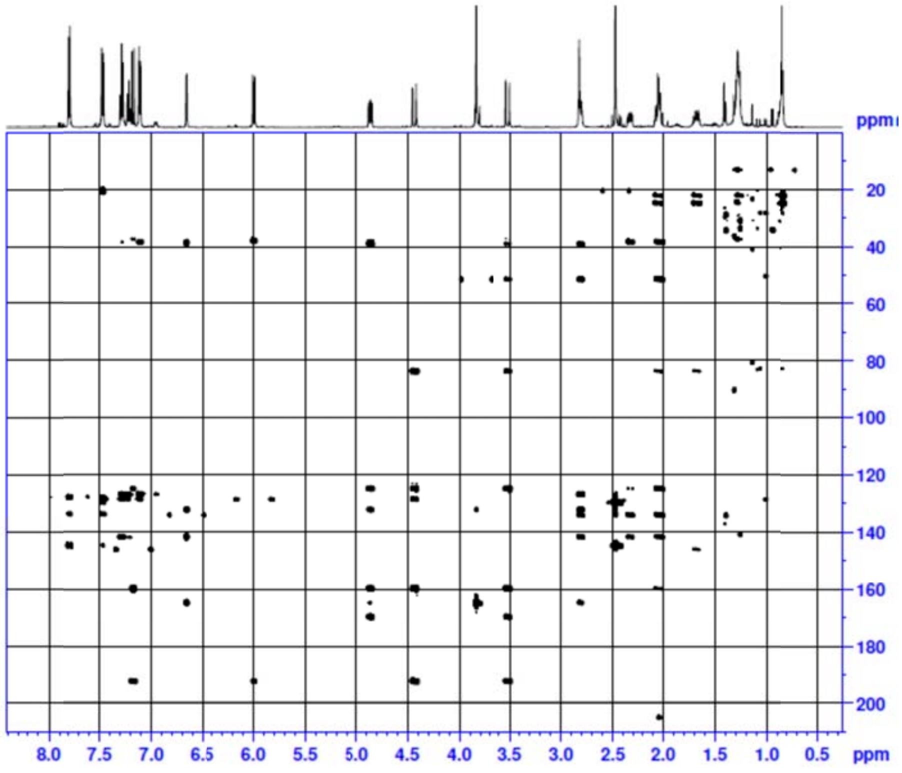

Supplementary Figure 53. HMBC spectra for compound **13**

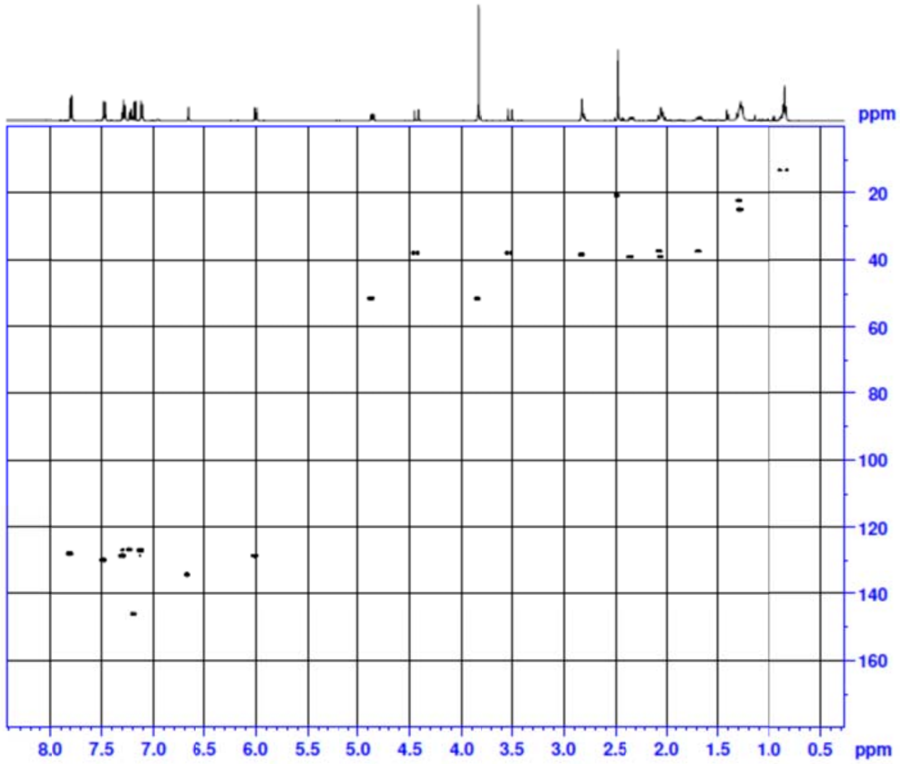

Supplementary Figure 54. HMQC spectra for compound **13**

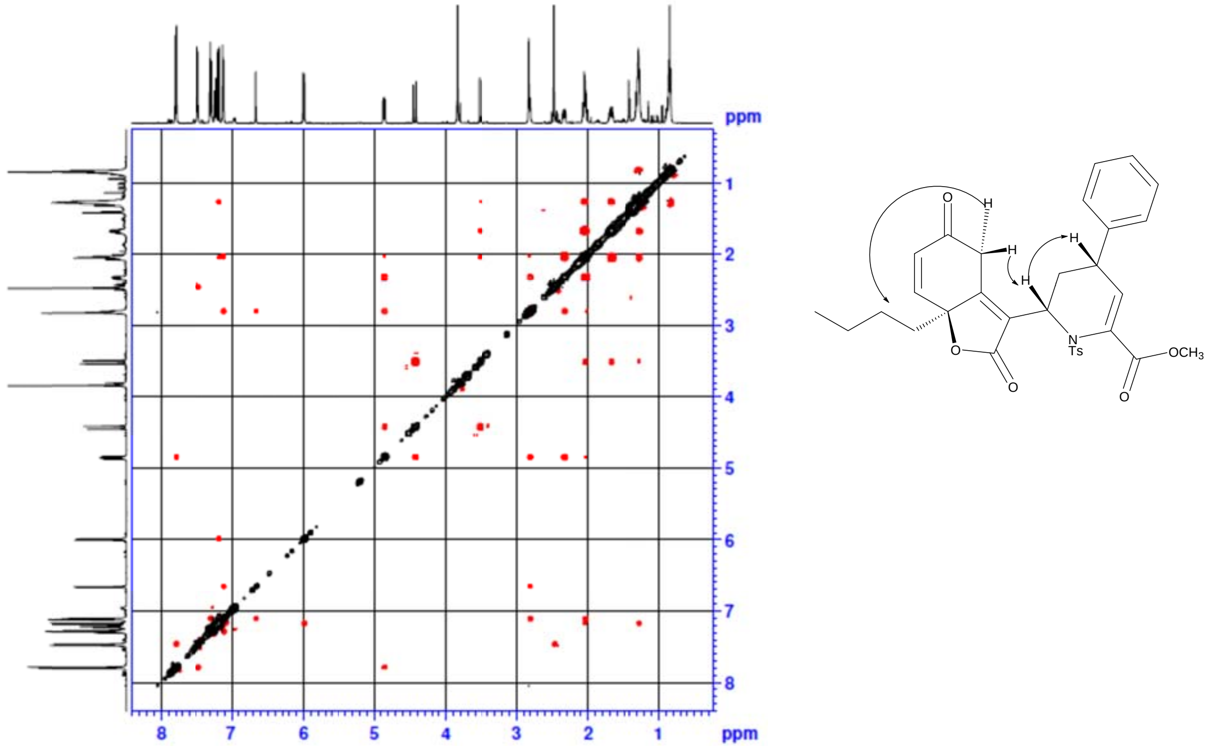

Supplementary Figure 55. NOESY spectra for compound **13**

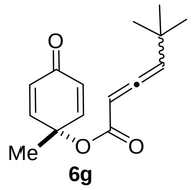

6g

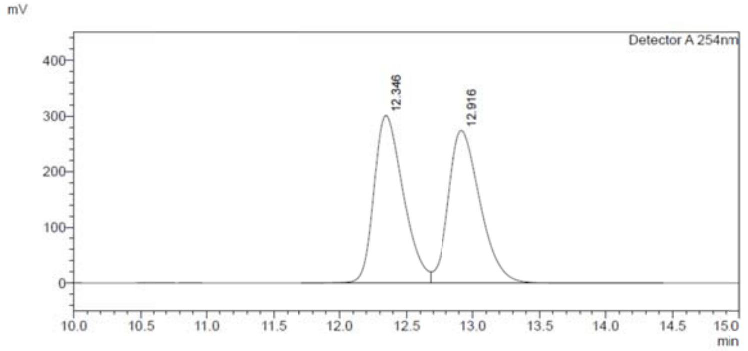

<Peak Table>

Detector A 254nm

Peak#	Ret. Time	Area	Height	Conc.	Unit	Mark	Name
1	12.346	4577714	301241	50.202			
2	12.916	4540844	274639	49.798		V	
Total		9118558	575880				

Racemic **6g**

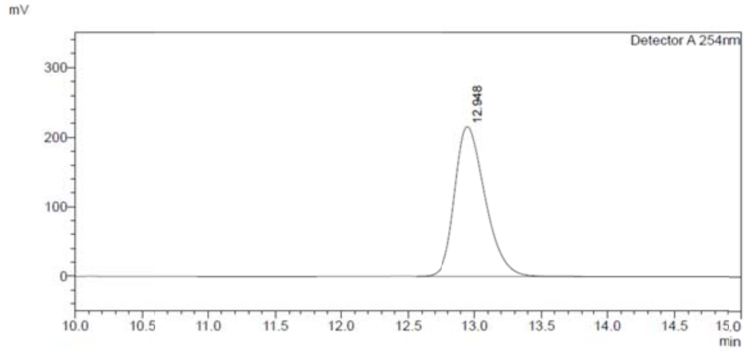

<Peak Table>

Detector A 254nm

Peak#	Ret. Time	Area	Height	Conc.	Unit	Mark	Name
1	12.948	3409588	215648	100.000			
Total		3409588	215648				

Optically pure **6g-1**

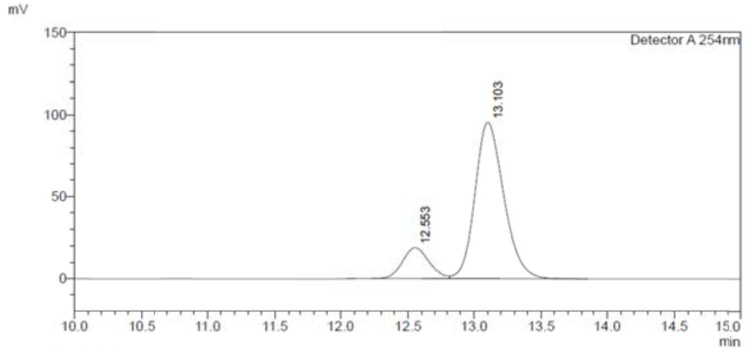

<Peak Table>

Detector A 254nm

Peak#	Ret. Time	Area	Height	Conc.	Unit	Mark	Name
1	12.553	260377	18935	15.477			
2	13.103	1421944	95420	84.523		V	
Total		1682321	114355				

Recover of **6g-1’**

Supplementary Figure 56. HPLC spectra for compound **6g** obtained via by use of a chiral stationary phase column (see Supplementary methods)

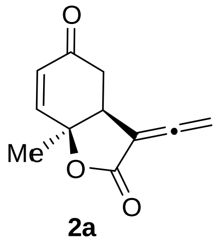

2a

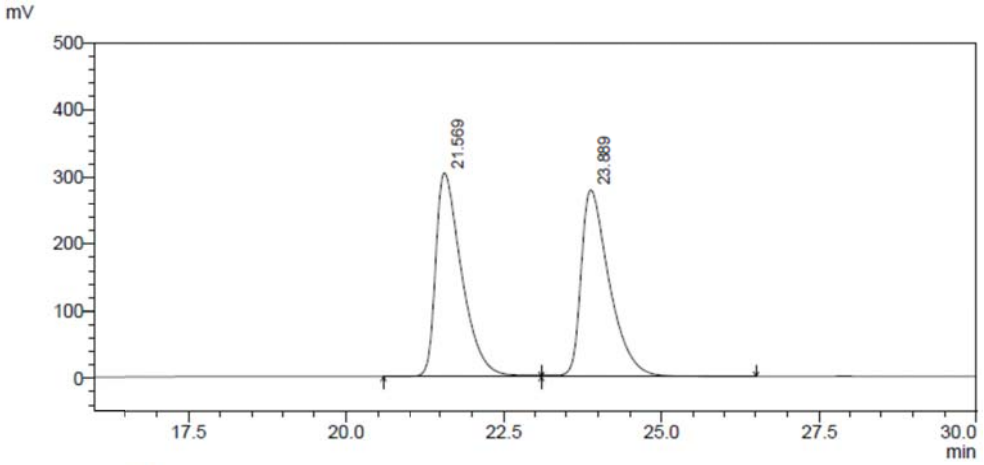

<Peak Table>

Detector A 215nm

Peak#	Ret. Time	Area	Height	Conc.	Unit	Mark	Name
1	21.569	8665120	304146	50.123		M	
2	23.889	8622716	278570	49.877		V M	
Total		17287836	582716				

Racemic **2a**

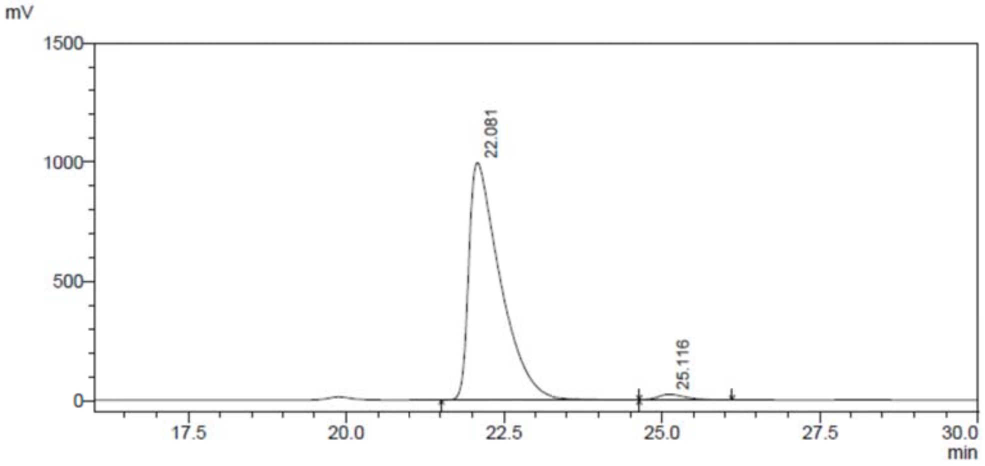

<Peak Table>

Detector A 215nm

Peak#	Ret. Time	Area	Height	Conc.	Unit	Mark	Name
1	22.081	34153292	993976	97.993		M	
2	25.116	699343	23268	2.007		V M	
Total		34852636	1017244				

Enantiomerically enriched **2a**

Supplementary Figure 57. HPLC spectra for compound **2a**

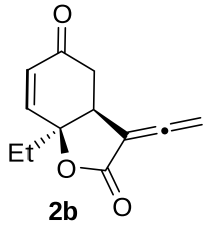

2b

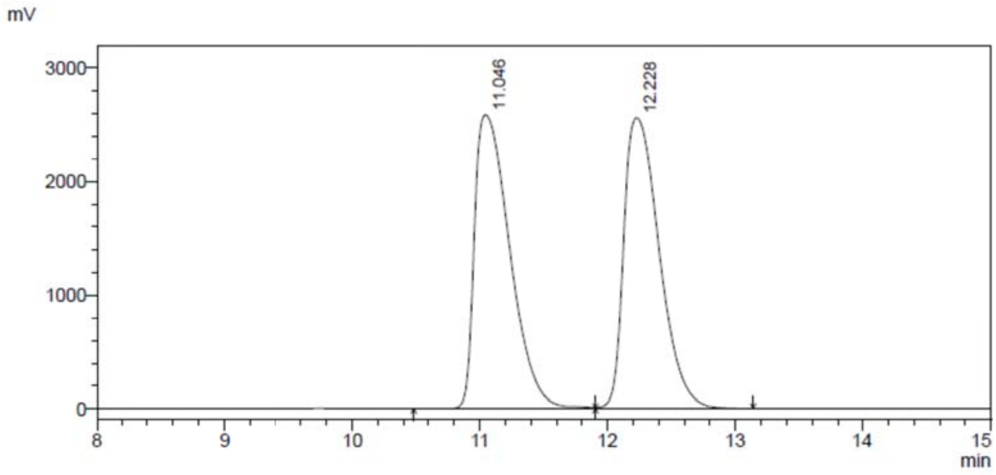

<Peak Table>

Detector A 215nm

Peak#	Ret. Time	Area	Height	Conc.	Unit	Mark	Name
1	11.046	49251633	2590163	50.161			
2	12.228	48935368	2562109	49.839		V	
Total		98187001	5152272				

Racemic **2b**

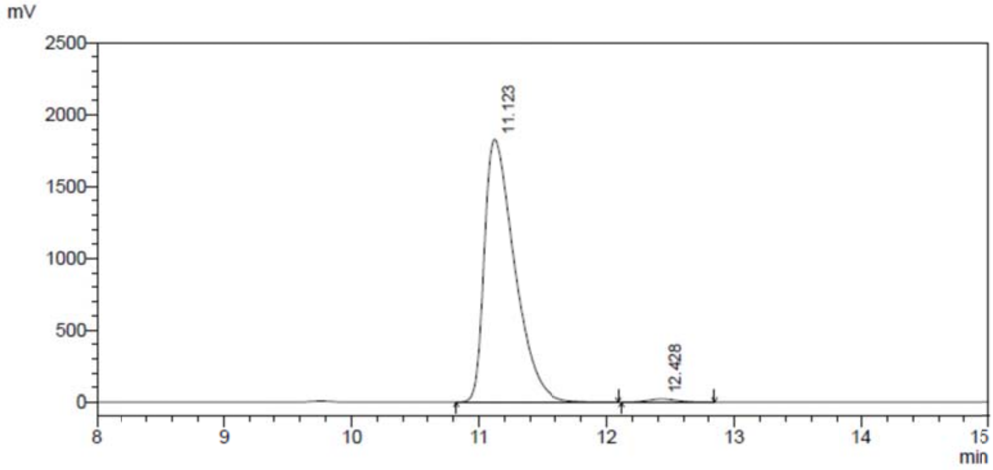

<Peak Table>

Detector A 215nm

Peak#	Ret. Time	Area	Height	Conc.	Unit	Mark	Name
1	11.123	29789315	1829287	98.876		M	
2	12.428	338761	22303	1.124		M	
Total		30128076	1851590				

Enantiomerically enriched **2b**

Supplementary Figure 58. HPLC spectra for compound **2b**

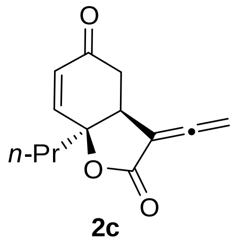

2c

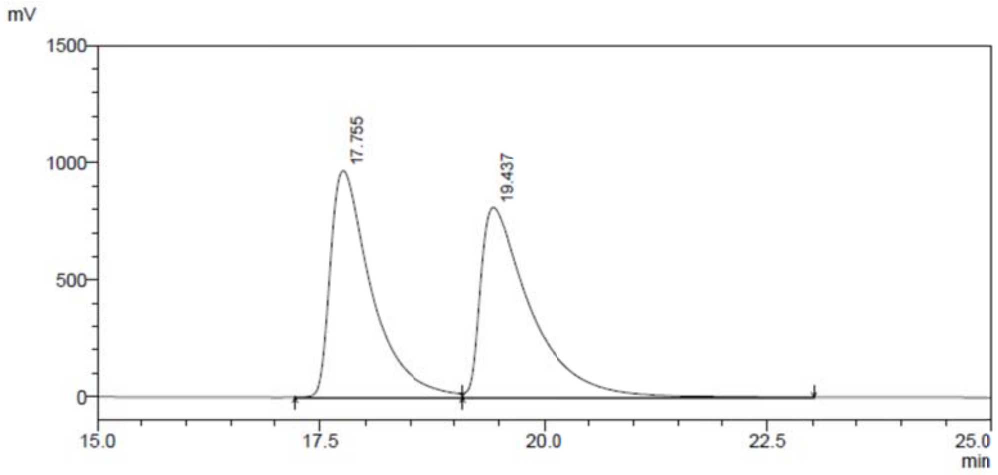

<Peak Table>

Detector A 215nm

Peak#	Ret. Time	Area	Height	Conc.	Unit	Mark	Name
1	17.755	30675174	968598	49.405		M	
2	19.437	31413446	810058	50.595		V M	
Total		62088620	1778657				

Racemic **2c**

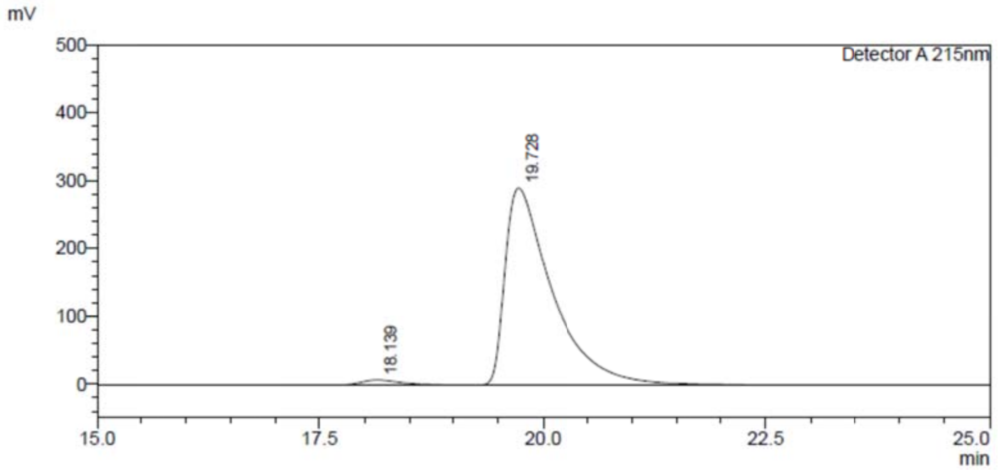

<Peak Table>

Detector A 215nm

Peak#	Ret. Time	Area	Height	Conc.	Unit	Mark	Name
1	18.139	240772	7715	2.184			
2	19.728	10783419	290723	97.816			
Total		11024191	298438				

Enantiomerically enriched **2c**

Supplementary Figure 59. HPLC spectra for compound **2c**

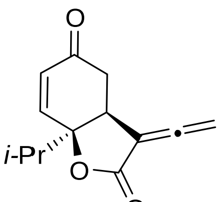

2d

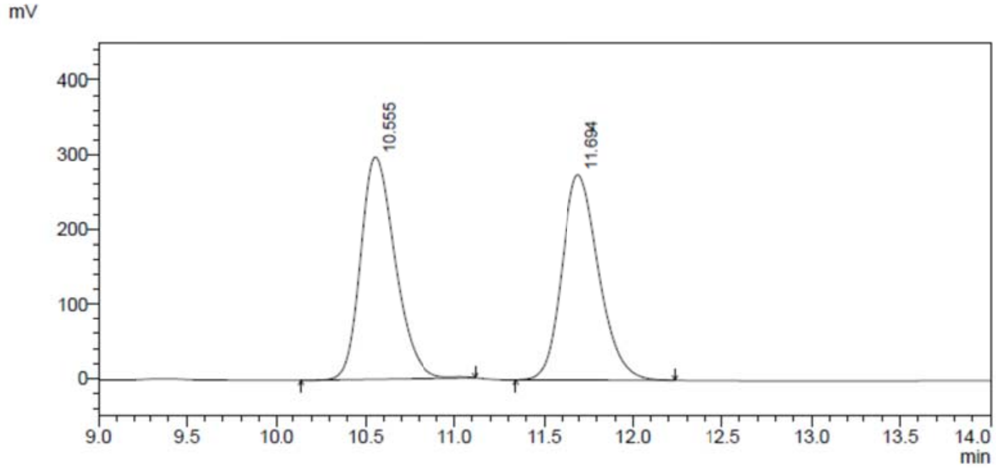

<Peak Table>

Detector A 215nm

Peak#	Ret. Time	Area	Height	Conc.	Unit	Mark	Name
1	10.555	3988167	298379	49.963		M	
2	11.694	3994067	275133	50.037		M	
Total		7982234	573512				

Racemic **2d**

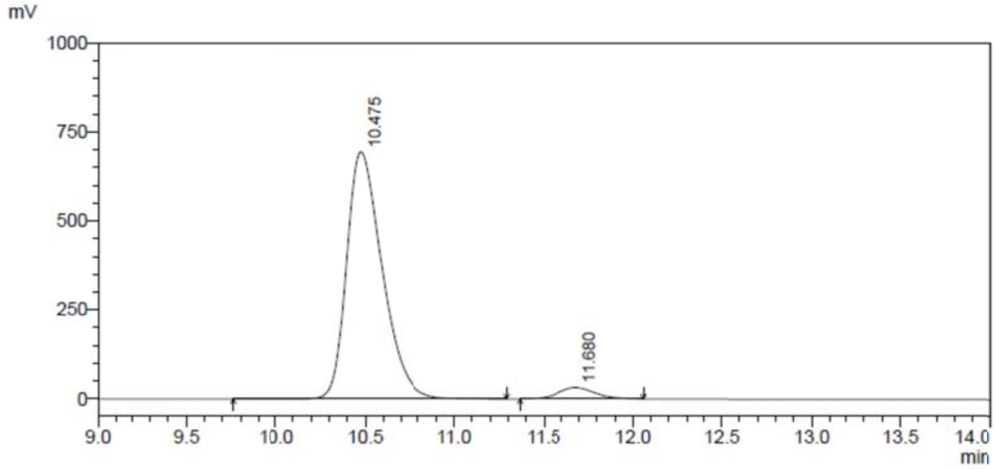

<Peak Table>

Detector A 215nm

Peak#	Ret. Time	Area	Height	Conc.	Unit	Mark	Name
1	10.475	9555851	694969	95.508		M	
2	11.680	449468	31775	4.492		M	
Total		10005319	726745				

Enantiomerically enriched **2d**

Supplementary Figure 60. HPLC spectra for compound **2d**

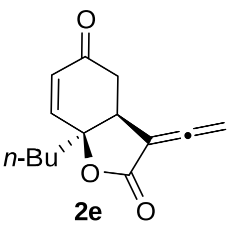

2e

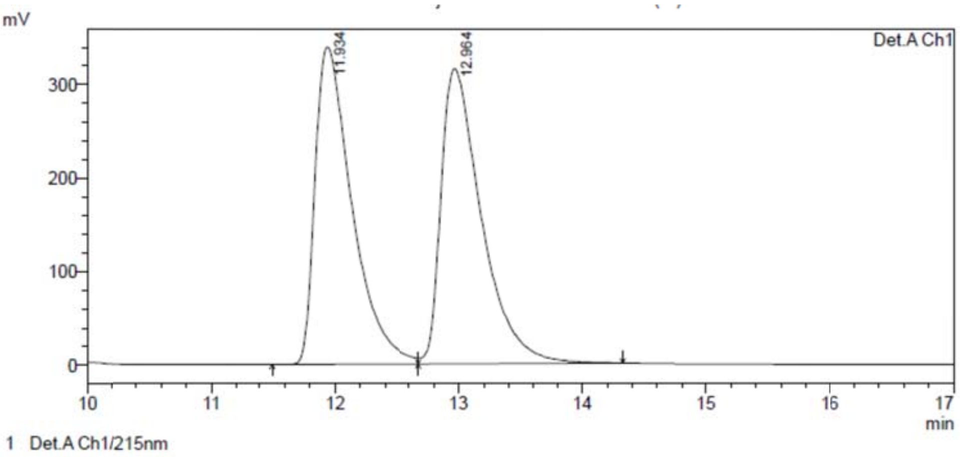

PeakTable

Detector A Ch1 215nm

Peak#	Ret. Time	Area	Height	Area %	Height %
1	11.934	6964224	339654	49.478	51.781
2	12.964	7111237	316291	50.522	48.219
Total		14075462	655945	100.000	100.000

Racemic **2e**

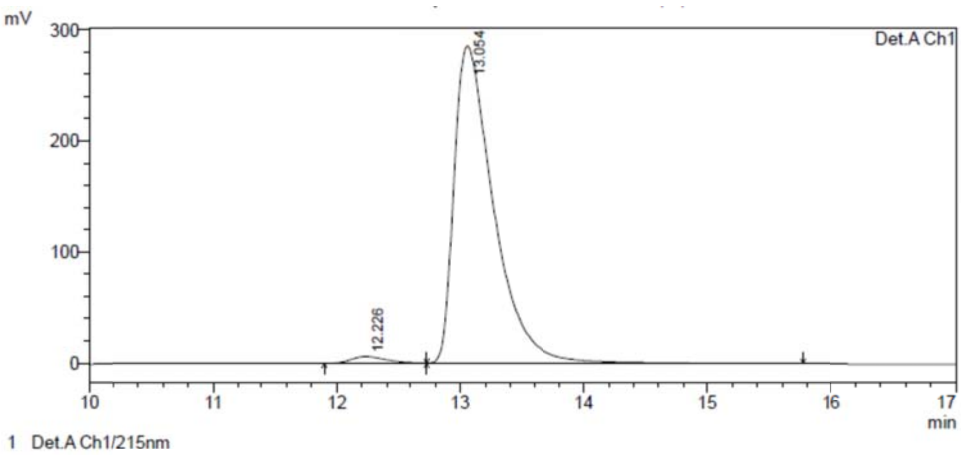

PeakTable

Detector A Ch1 215nm

Peak#	Ret. Time	Area	Height	Area %	Height %
1	12.226	129926	6306	2.003	2.154
2	13.054	6357942	286383	97.997	97.846
Total		6487868	292689	100.000	100.000

Enantiomerically enriched **2e**

Supplementary Figure 61. HPLC spectra for compound **2e**

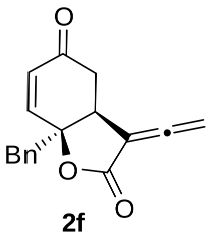

2f

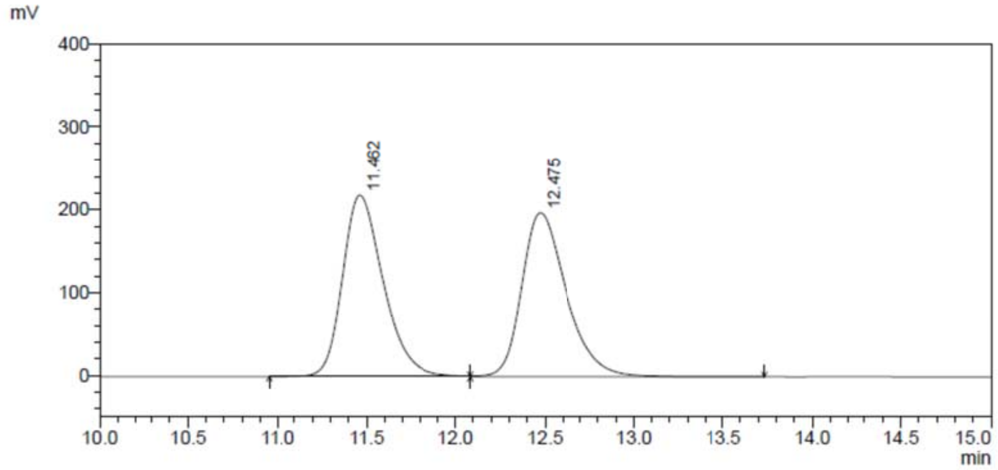

<Peak Table>

Detector A 215nm

Peak#	Ret. Time	Area	Height	Conc.	Unit	Mark	Name
1	11.462	3442081	218370	49.964			
2	12.475	3446993	197626	50.036		V	
Total		6889073	415996				

Racemic **2f**

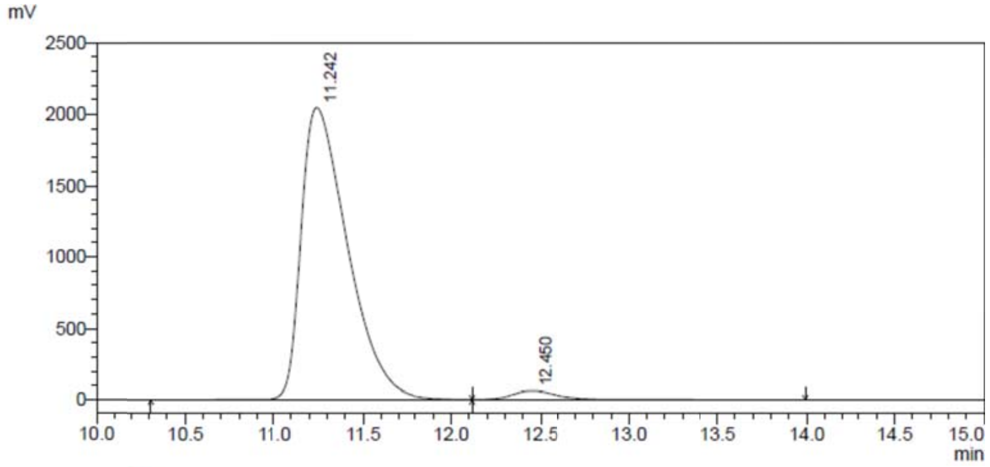

<Peak Table>

Detector A 215nm

Peak#	Ret. Time	Area	Height	Conc.	Unit	Mark	Name
1	11.242	36746773	2048919	97.067			
2	12.450	1110262	61915	2.933		V	
Total		37857035	2110834				

Enantiomerically enriched **2f**

Supplementary Figure 62. HPLC spectra for compound **2f**

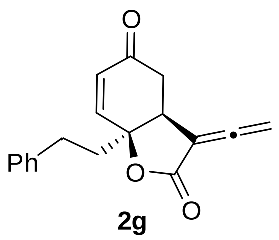

2g

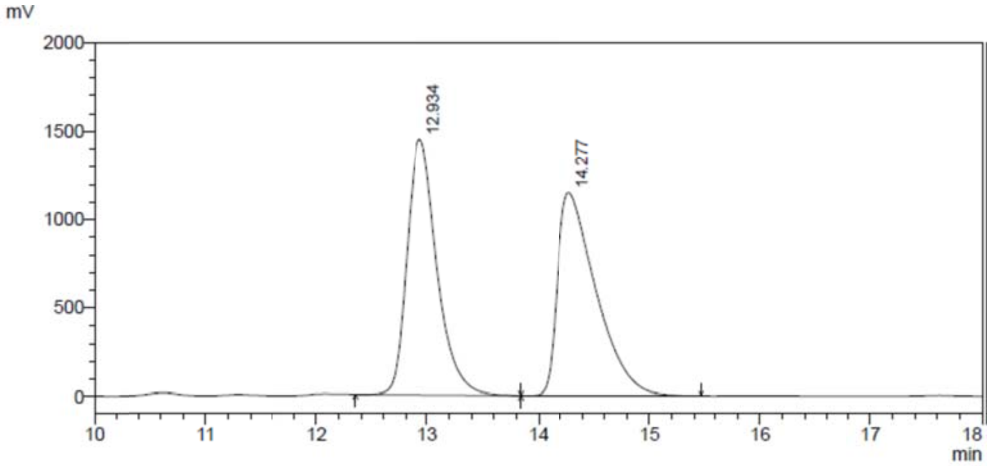

<Peak Table>

Detector A 215nm

Peak#	Ret. Time	Area	Height	Conc.	Unit	Mark	Name
1	12.934	27754100	1446305	49.843		M	
2	14.277	27928621	1147739	50.157		M	
Total		55682722	2594044				

Racemic **2g**

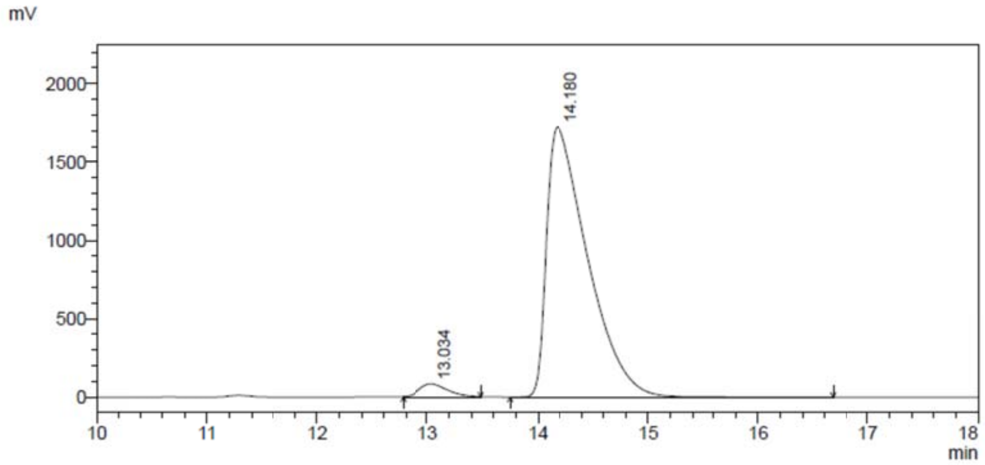

<Peak Table>

Detector A 215nm

Peak#	Ret. Time	Area	Height	Conc.	Unit	Mark	Name
1	13.034	1489827	83322	3.191		M	
2	14.180	45192274	1723767	96.809		M	
Total		46682101	1807089				

Enantiomerically enriched **2g**

Supplementary Figure 63. HPLC spectra for compound **2g**

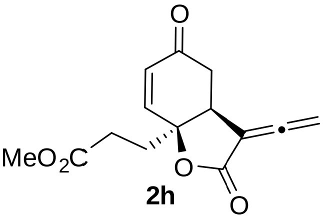

2h

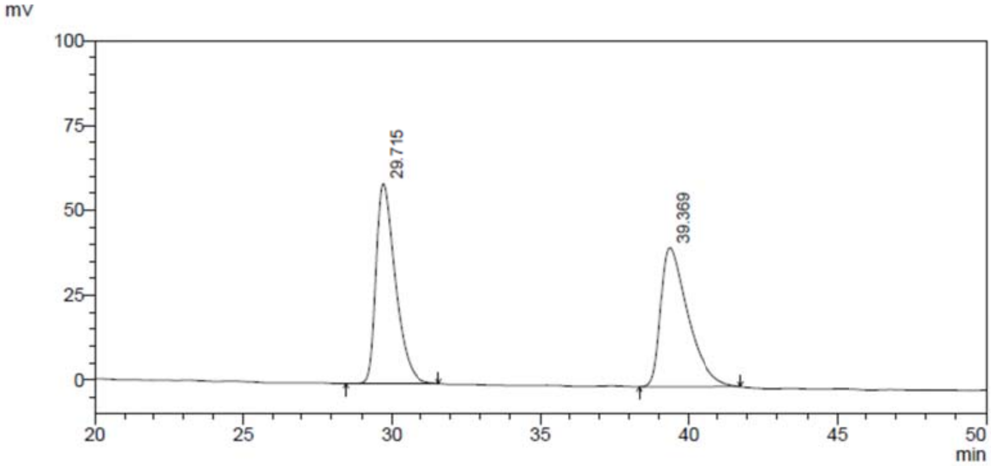

<Peak Table>

Detector A 215nm

Peak#	Ret. Time	Area	Height	Conc.	Unit	Mark	Name
1	29.715	2645202	58830	50.031		M	
2	39.369	2641886	41170	49.969		M	
Total		5287088	100001				

Racemic **2h**

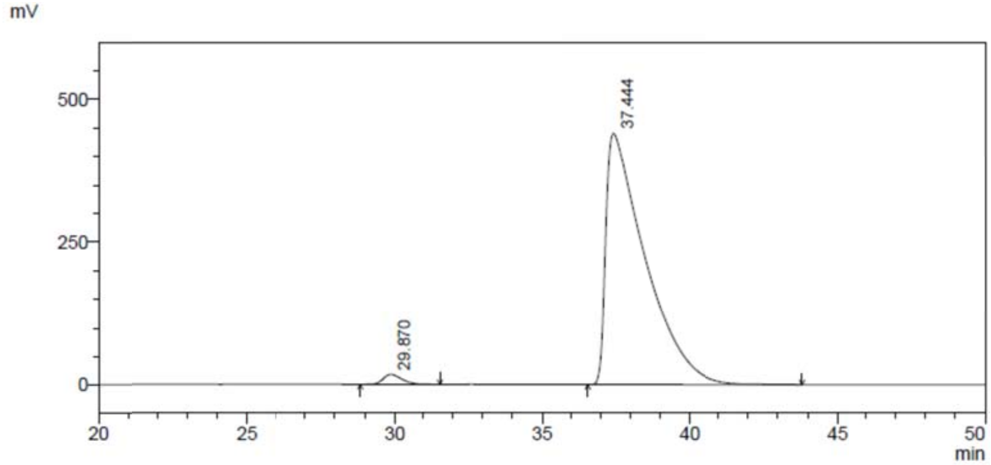

<Peak Table>

Detector A 215nm

Peak#	Ret. Time	Area	Height	Conc.	Unit	Mark	Name
1	29.870	769897	17531	1.870			
2	37.444	40406771	439845	98.130			
Total		41176668	457377				

Enantiomerically enriched **2h**

Supplementary Figure 64. HPLC spectra for compound **2h**

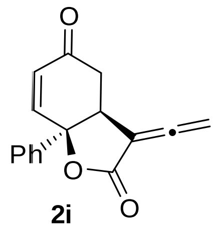

2i

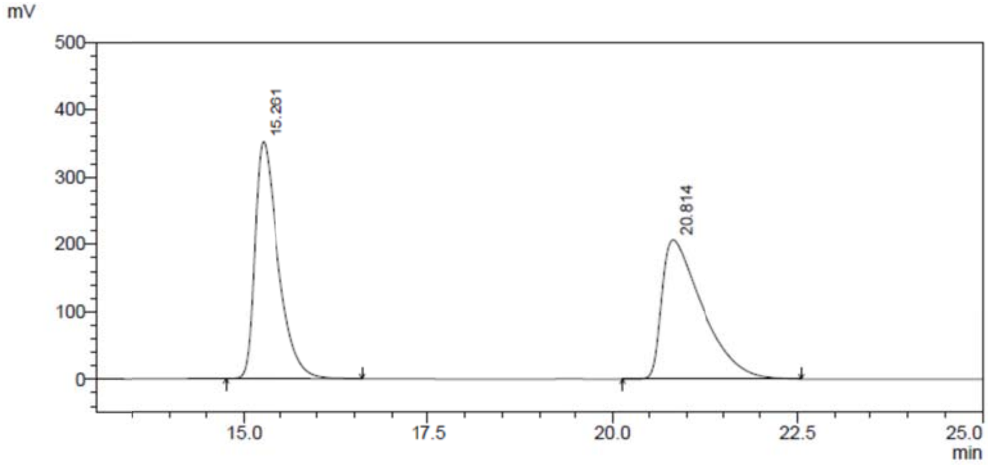

<Peak Table>

Detector A 215nm

Peak#	Ret. Time	Area	Height	Conc.	Unit	Mark	Name
1	15.261	7846790	351900	49.785		M	
2	20.814	7914462	206405	50.215		M	
Total		15761252	558305				

Racemic **2i**

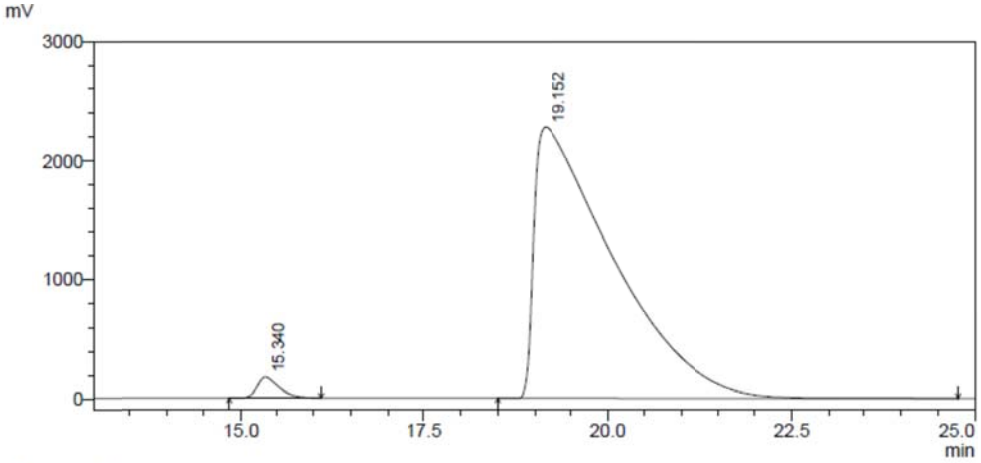

<Peak Table>

Detector A 215nm

Peak#	Ret. Time	Area	Height	Conc.	Unit	Mark	Name
1	15.340	3879207	178012	2.219		M	
2	19.152	170949225	2281270	97.781		M	
Total		174828432	2459282				

Enantiomerically enriched **2i**

Supplementary Figure 65. HPLC spectra for compound **2i**

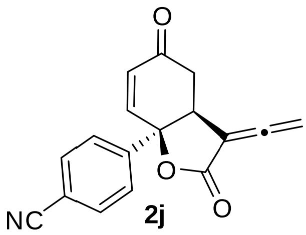

2j

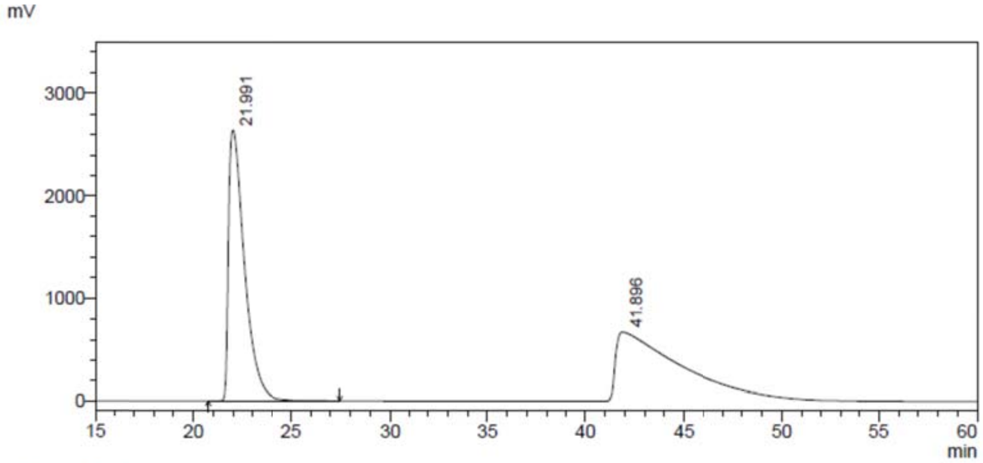

<Peak Table>

Detector A 215nm

Peak#	Ret. Time	Area	Height	Conc.	Unit	Mark	Name
1	21.991	147663105	2642503	48.166			
2	41.896	158906280	671527	51.834		M	
Total		306569384	3314030				

Racemic **2j**

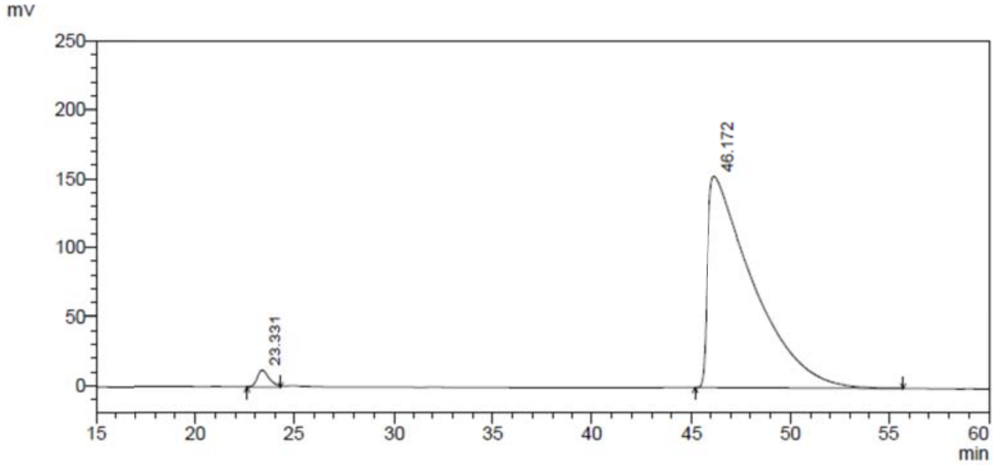

<Peak Table>

Detector A 215nm

Peak#	Ret. Time	Area	Height	Conc.	Unit	Mark	Name
1	23.331	517569	12283	2.066		M	
2	46.172	24528243	153658	97.934			
Total		25045812	165941				

Enantiomerically enriched **2j**

Supplementary Figure 66. HPLC spectra for compound **2j**

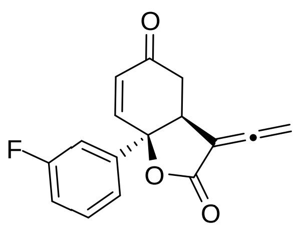

2k

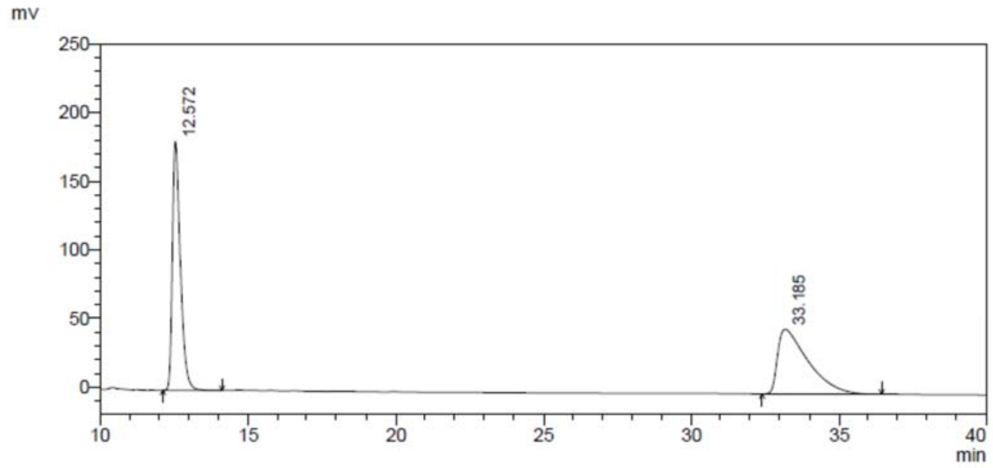

<Peak Table>

Detector A 215nm

Peak#	Ret. Time	Area	Height	Conc.	Unit	Mark	Name
1	12.572	3347369	181171	50.183			
2	33.185	3322933	46958	49.817			
Total		6670301	228128				

Racemic **2k**

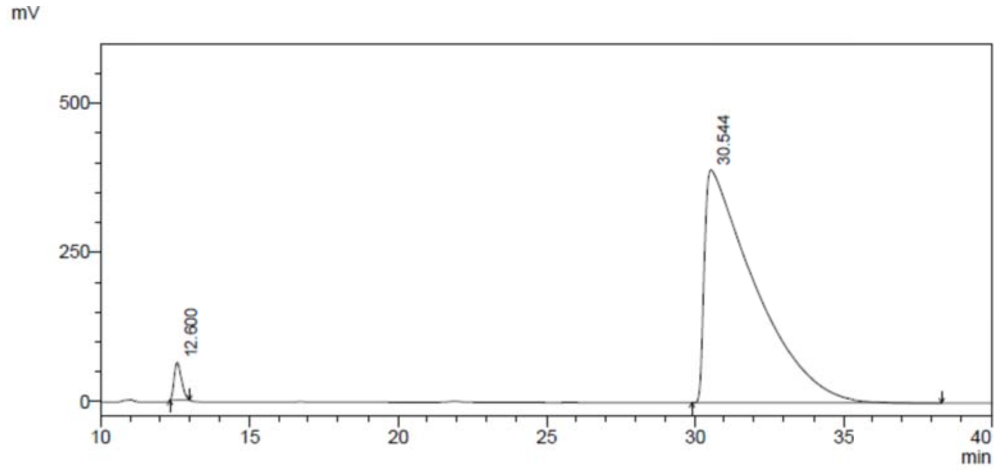

<Peak Table>

Detector A 215nm

Peak#	Ret. Time	Area	Height	Conc.	Unit	Mark	Name
1	12.600	1060087	63107	2.222		M	
2	30.544	46656273	390226	97.778			
Total		47716361	453333				

Enantiomerically enriched **2k**

Supplementary Figure 67. HPLC spectra for compound **2k**

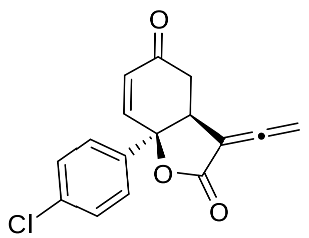

2l

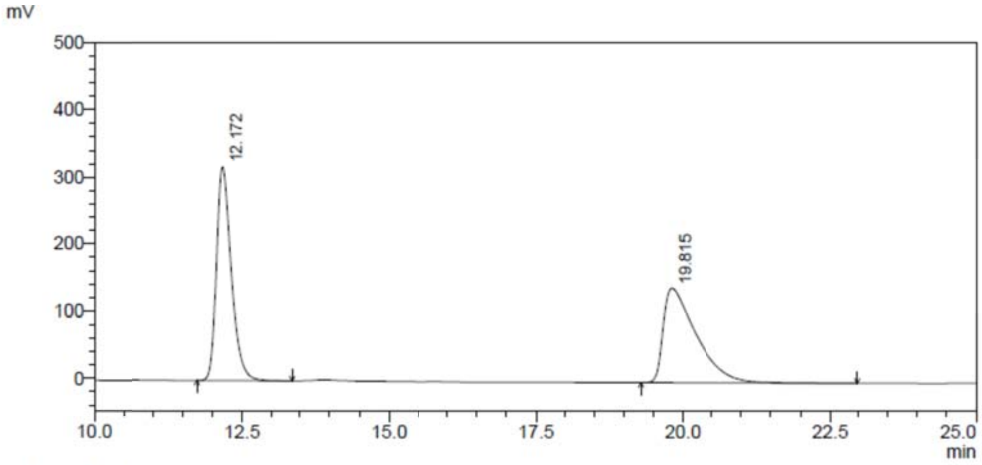

<Peak Table>

Detector A 215nm

Peak#	Ret. Time	Area	Height	Conc.	Unit	Mark	Name
1	12.172	5537961	318592	49.939			
2	19.815	5551382	141621	50.061			
Total		11089343	460213				

Racemic **2l**

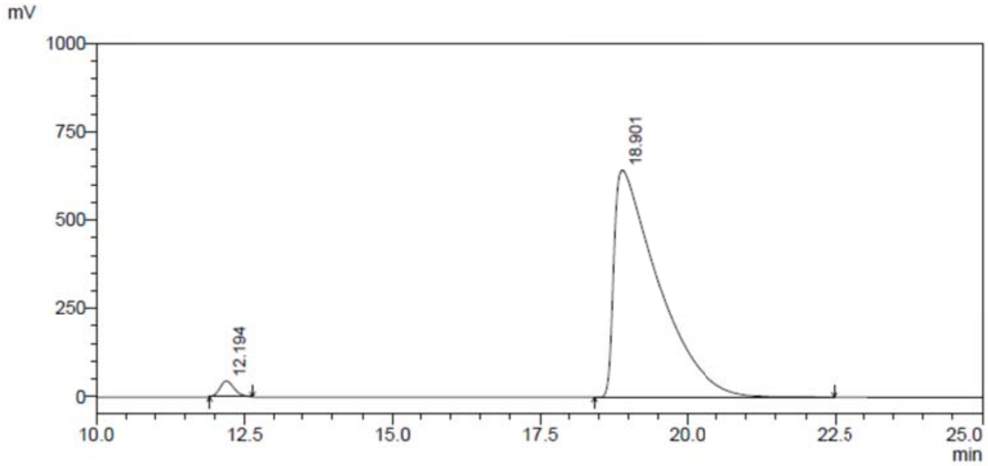

<Peak Table>

Detector A 215nm

Peak#	Ret. Time	Area	Height	Conc.	Unit	Mark	Name
1	12.194	735356	44586	2.159		M	
2	18.901	33321128	642183	97.841		M	
Total		34056483	686769				

Enantiomerically enriched **2l**

Supplementary Figure 68. HPLC spectra for compound **2l**

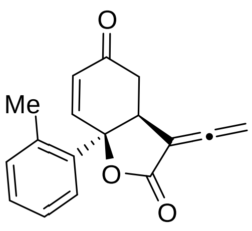

2m

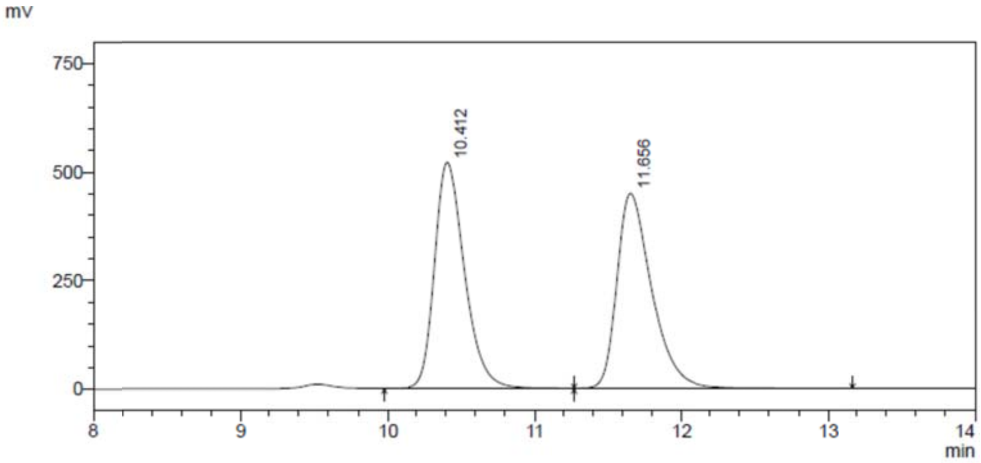

<Peak Table>

Detector A 215nm

Peak#	Ret. Time	Area	Height	Conc.	Unit	Mark	Name
1	10.412	7343168	521483	49.901			
2	11.656	7372381	449522	50.099		V	
Total		14715549	971005				

Racemic **2m**

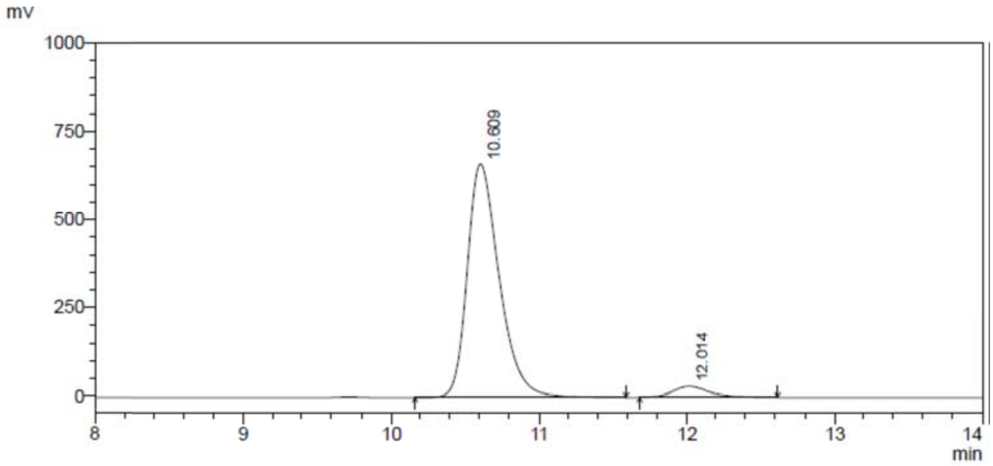

<Peak Table>

Detector A 215nm

Peak#	Ret. Time	Area	Height	Conc.	Unit	Mark	Name
1	10.609	9844480	661067	94.927		M	
2	12.014	526084	30424	5.073		M	
Total		10370564	691491				

Enantiomerically enriched **2m**

Supplementary Figure 69. HPLC spectra for compound **2m**

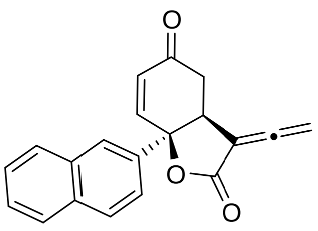

2n

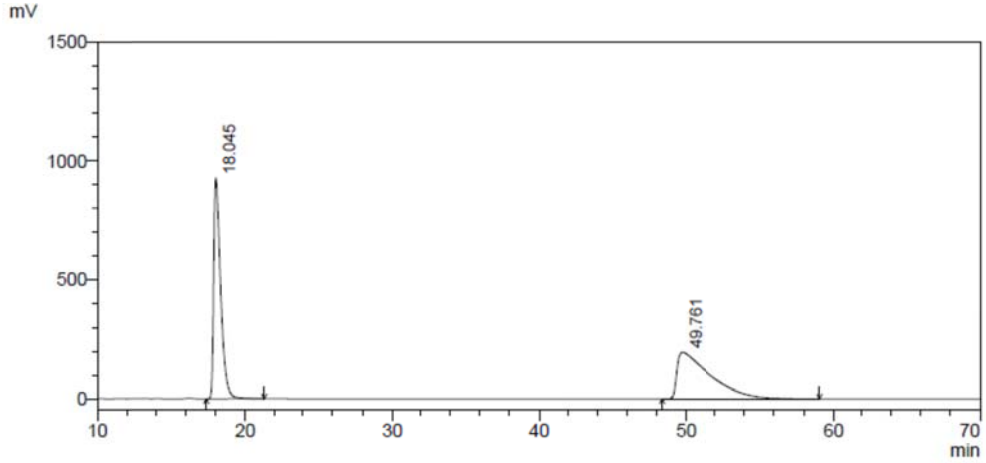

<Peak Table>

Detector A 215nm

Peak#	Ret. Time	Area	Height	Conc.	Unit	Mark	Name
1	18.045	29874577	926295	49.159			
2	49.761	30897216	194998	50.841			
Total		60771793	1121294				

Racemic **2n**

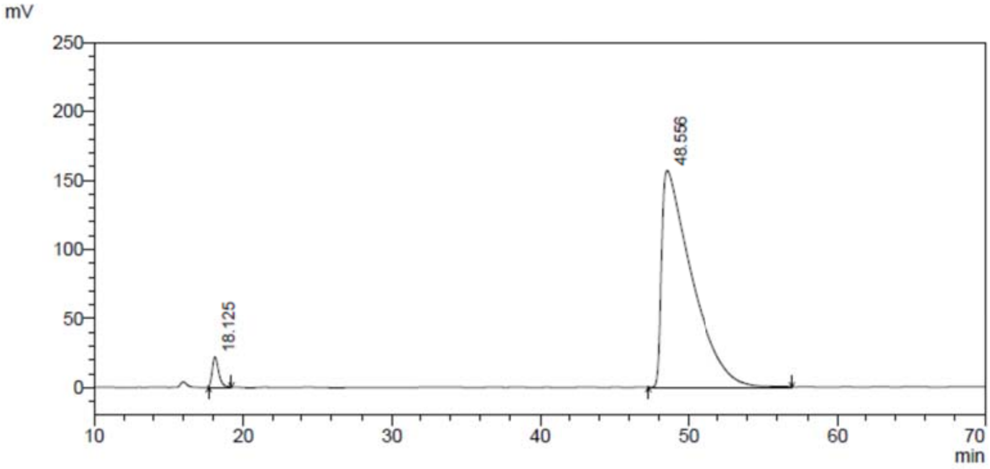

<Peak Table>

Detector A 215nm

Peak#	Ret. Time	Area	Height	Conc.	Unit	Mark	Name
1	18.125	654345	21919	2.861		M	
2	48.556	22218043	156738	97.139		M	
Total		22872388	178657				

Enantiomerically enriched **2n**

Supplementary Figure 70. HPLC spectra for compound **2n**

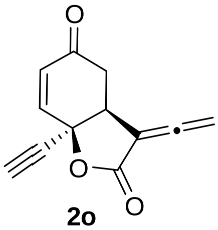

2o

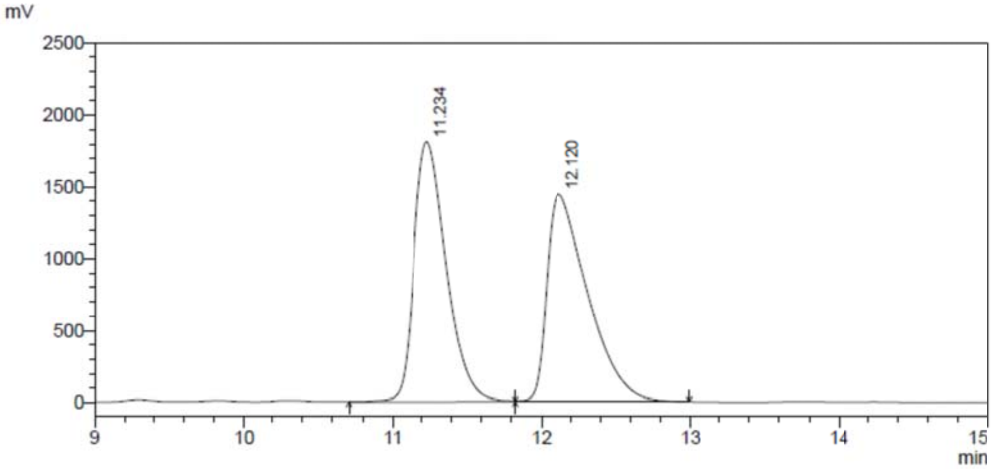

<Peak Table>

Detector A 215nm

Peak#	Ret. Time	Area	Height	Conc.	Unit	Mark	Name
1	11.234	27601657	1811794	49.744		M	
2	12.120	27885394	1442801	50.256		V M	
Total		55487051	3254595				

Racemic **2o**

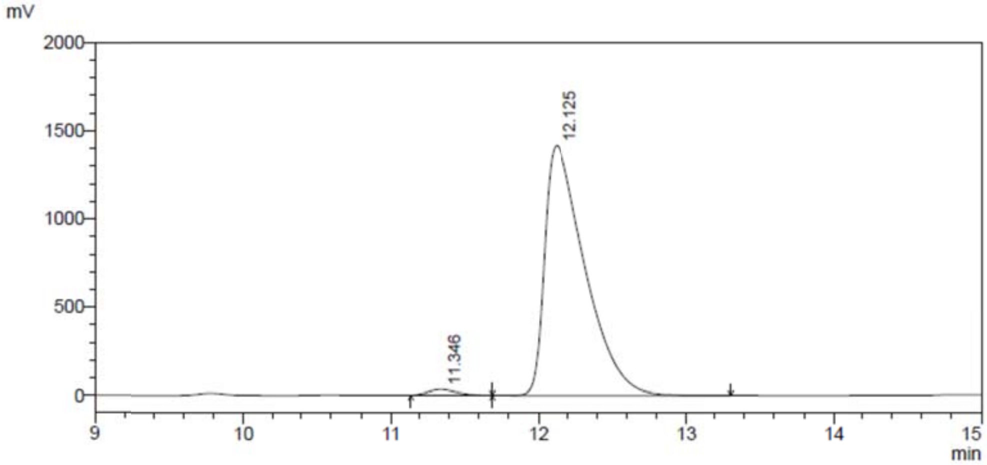

<Peak Table>

Detector A 215nm

Peak#	Ret. Time	Area	Height	Conc.	Unit	Mark	Name
1	11.346	467873	33760	1.700		M	
2	12.125	27047471	1417201	98.300		V M	
Total		27515344	1450960				

Enantiomerically enriched **2o**

Supplementary Figure 71. HPLC spectra for compound **2o**

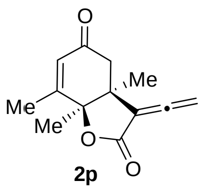

2p

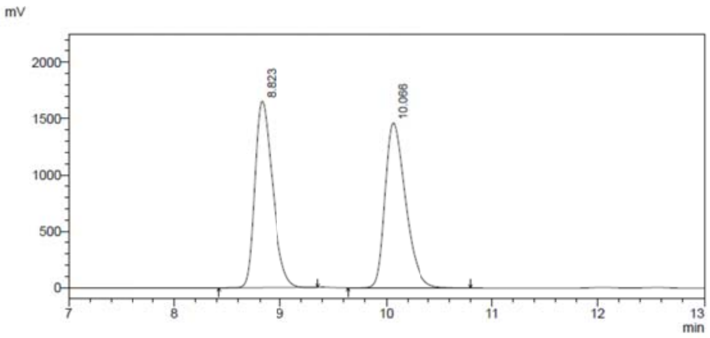

<Peak Table>

Detector A 215nm

Peak#	Ret. Time	Area	Height	Conc.	Unit	Mark	Name
1	8.823	19449482	1653487	49.674		M	
2	10.066	19705018	1463008	50.326		M	
Total		39154500	3116495				

Racemic **2p**

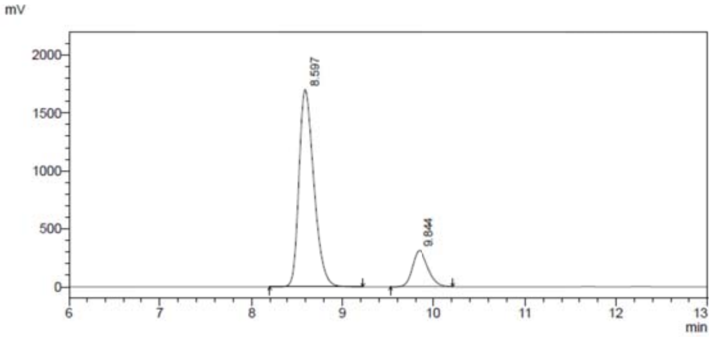

<Peak Table>

Detector A 215nm

Peak#	Ret. Time	Area	Height	Conc.	Unit	Mark	Name
1	8.597	19190323	1699143	83.356		M	
2	9.844	3831913	313301	16.644		M	
Total		23022236	2012444				

Chiral **2p** before recrystallization

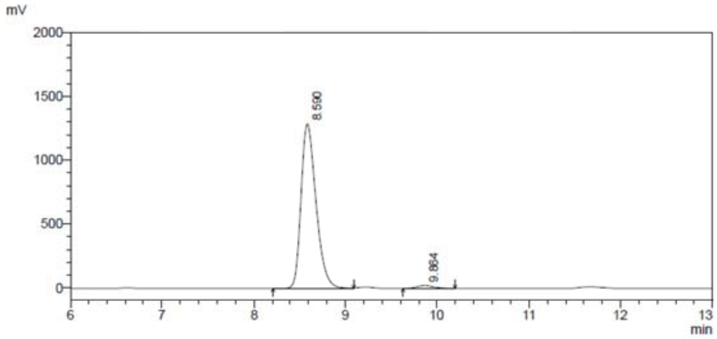

<Peak Table>

Detector A 215nm

Peak#	Ret. Time	Area	Height	Conc.	Unit	Mark	Name
1	8.590	14470678	1288187	97.941		M	
2	9.864	304286	23334	2.059		M	
Total		14774964	1311521				

Enantiomerically enriched **2p** after recrystallization

Supplementary Figure 72. HPLC spectra for compound **2p**

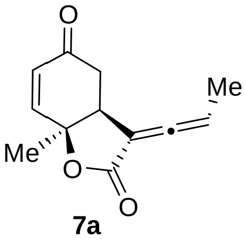

7a

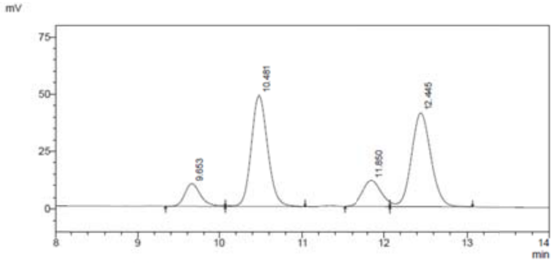

Detector A 215nm

Peak#	Ret. Time	Area	Height	Conc.	Unit
1	9.653	131964	9901	8.048	
2	10.481	664740	48477	40.539	
3	11.850	177172	11299	10.805	
4	12.445	665862	40990	40.608	
Total		1639738	110666		

Racemic **7a**

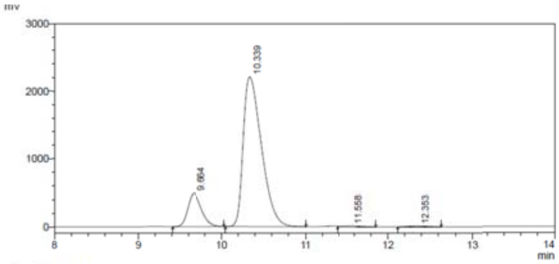

<Peak Table>

Detector A 215nm

Peak#	Ret. Time	Area	Height	Conc.	Unit	Mark	Name
1	9.664	5823623	488504	14.692		M	
2	10.339	33393299	2214243	84.247		M	
3	11.558	259603	13731	0.655		M	
4	12.353	161059	11053	0.406		M	
Total		39637584	2727531				

Enantiomerically enriched **7a**

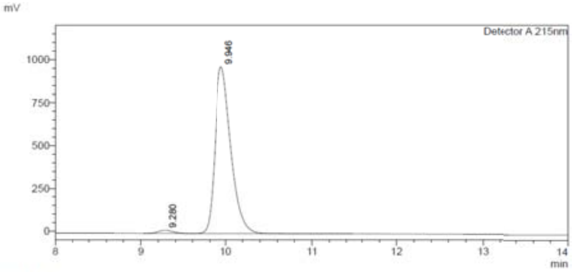

<Peak Table>

Detector A 215nm

Peak#	Ret. Time	Area	Height	Conc.	Unit	Mark	Name
1	9.280	214240	18373	1.611			
2	9.946	13084321	971519	98.389		M	
Total		13298561	989892				

Optically pure **7a** after Recrystallization

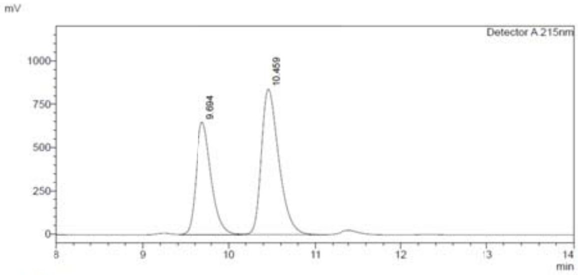

<Peak Table>

Detector A 215nm

Peak#	Ret. Time	Area	Height	Conc.	Unit	Mark	Name
1	9.694	8088972	650224	40.849			
2	10.459	11713048	840155	59.151		V	
Total		19802020	1490379				

7a’

Supplementary Figure 73. HPLC spectra for compound **7a**

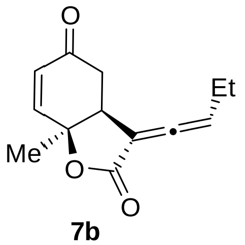

7b

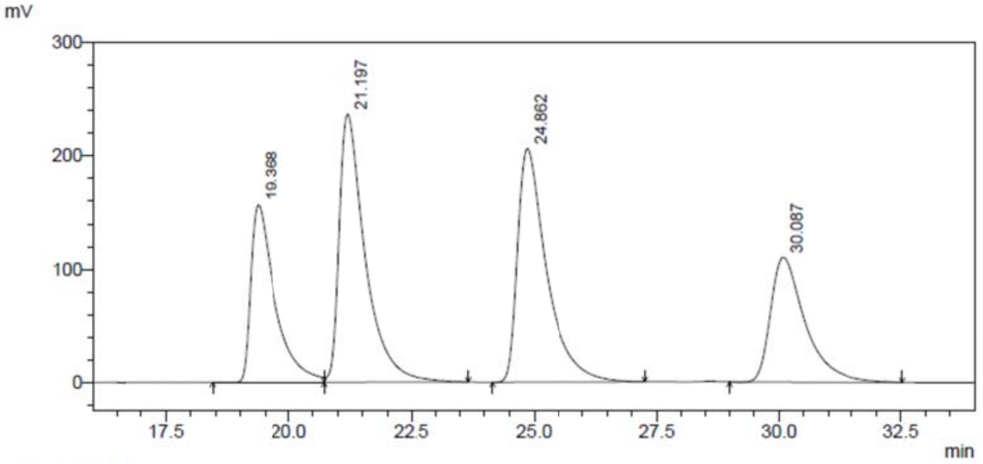

<Peak Table>

Detector A 215nm

Peak#	Ret. Time	Area	Height	Conc.	Unit	Mark	Name
1	19.368	5437606	156197	19.485		M	
2	21.197	8600578	235831	30.819		V M	
3	24.862	8495401	205605	30.442		M	
4	30.087	5373447	109875	19.255		M	
Total		27907033	707509				

Racemic **7b**

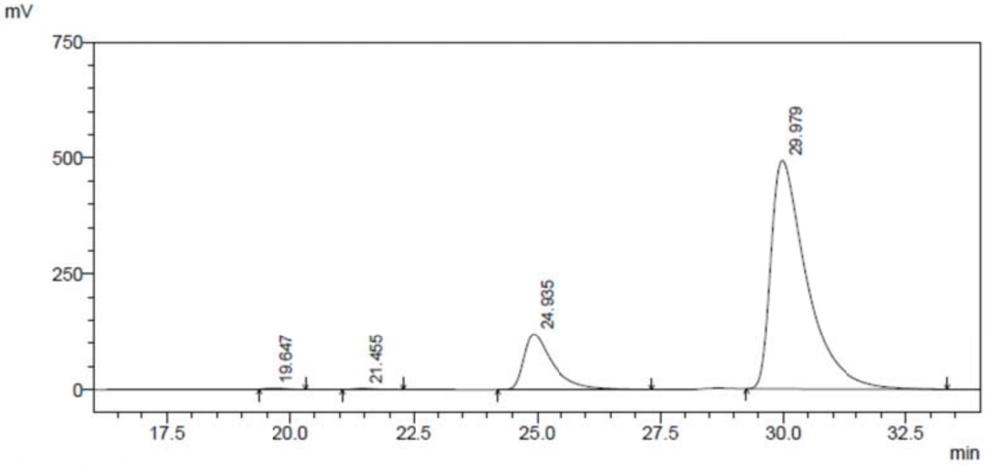

<Peak Table>

Detector A 215nm

Peak#	Ret. Time	Area	Height	Conc.	Unit	Mark	Name
1	19.647	61331	2184	0.205		M	
2	21.455	63143	2020	0.211		M	
3	24.935	4997722	120099	16.666		M	
4	29.979	24866002	493125	82.919		M	
Total		29988198	617428				

Enantiomerically enriched **7b**

Supplementary Figure 74. HPLC spectra for compound **7b**

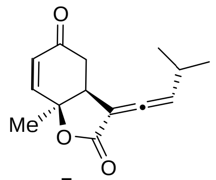

7c

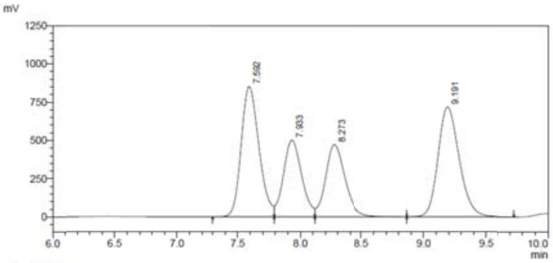

<Peak Table>

Detector A 215nm

Peak#	Ret. Time	Area	Heigh:	Conc.	Unit	Mark	Name
1	7.592	8408881	853464	31.066			
2	7.933	5058259	501210	18.688		V	
3	8.273	5147812	471718	19.018		V	
4	9.191	8452632	719313	31.228		V	
Total		27067583	2545705				

Racemic **7c**

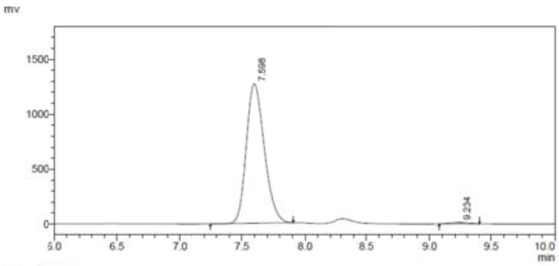

<Peak Table>

Detector A 215nm

Peak#	Ret. Time	Area	Height	Conc.	Unit	Mark	Name
1	7.598	12615838	1271908	99.039		M	
2	9.234	122363	12162	0.961		M	
Total		12738200	1284070				

Enantiomerically enriched **7c**

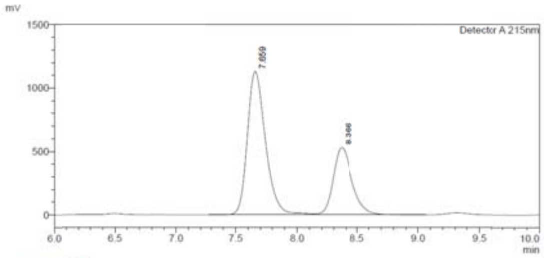

<Peak Table>

Detector A 215nm

Peak#	Ret. Time	Area	Height	Conc.	Unit	Mark	Name
1	7.659	11675889	1131462	66.189			
2	8.366	5964332	535159	33.811		V	
Total		17640221	1666621				

7c’

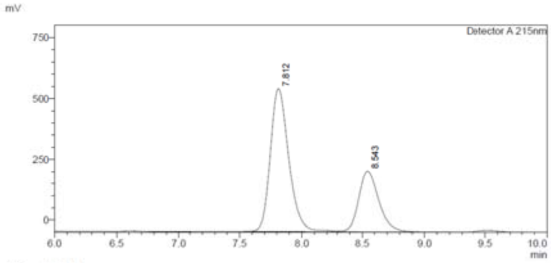

<Peak Table>

Detector A 215nm

Peak#	Ret. Time	Area	Height	Conc.	Unit	Mark	Name
1	7.812	6088175	588724	68.400			
2	8.543	2812678	251035	31.600		V	
Total		8900853	839760				

7c”

Supplementary Figure 75. HPLC spectra for compound **7c**

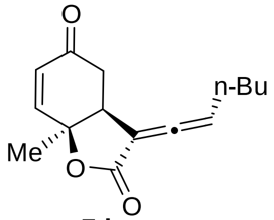

7d

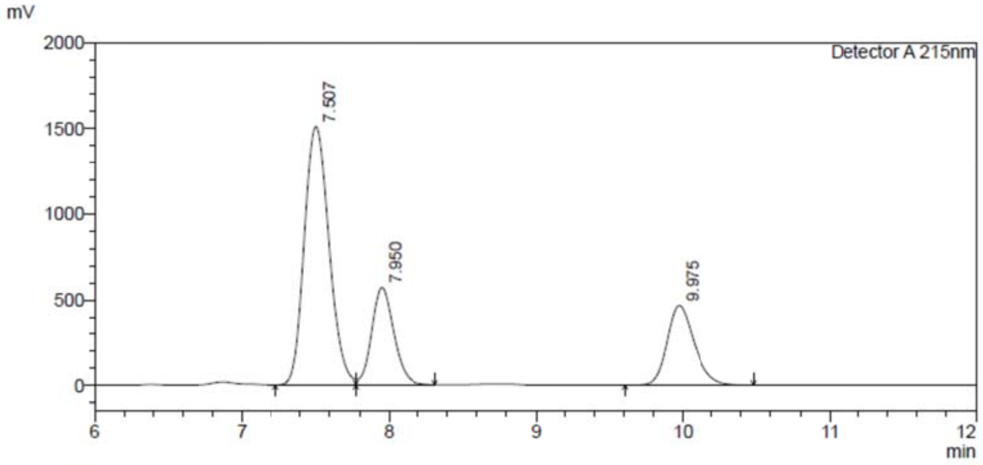

<Peak Table>

Detector A 215nm

Peak#	Ret. Time	Area	Height	Conc.	Unit	Mark	Name
1	7.507	17509646	1510597	59.444		M	
2	7.950	5931022	571776	20.135		V M	
3	9.975	6015000	466810	20.421		M	
Total		29455668	2549183				

Racemic **7d**

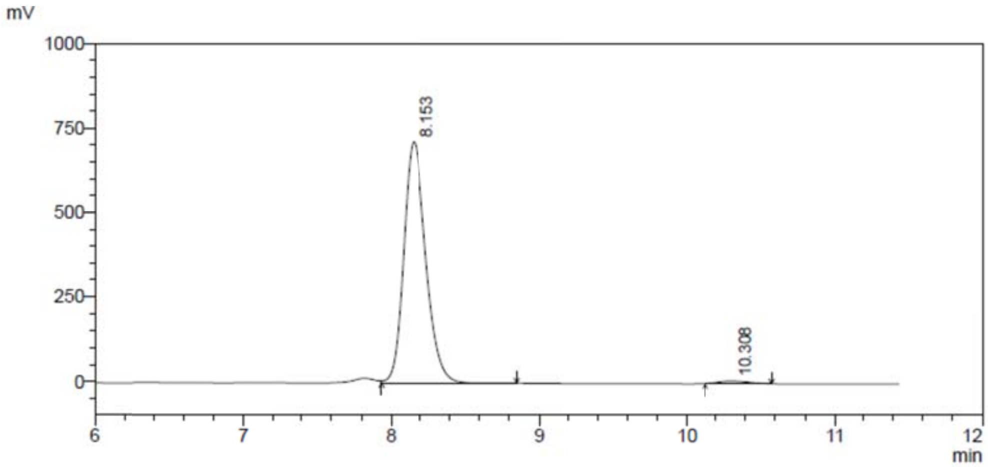

<Peak Table>

Detector A 215nm

Peak#	Ret. Time	Area	Height	Conc.	Unit	Mark	Name
1	8.153	7631473	715242	98.653		M	
2	10.308	104208	8174	1.347		M	
Total		7735681	723416				

Enantiomerically enriched **7d**

Supplementary Figure 76. HPLC spectra for compound **7d**

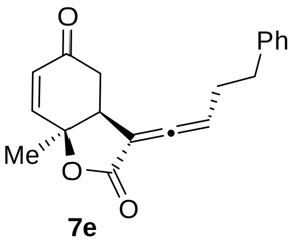

7e

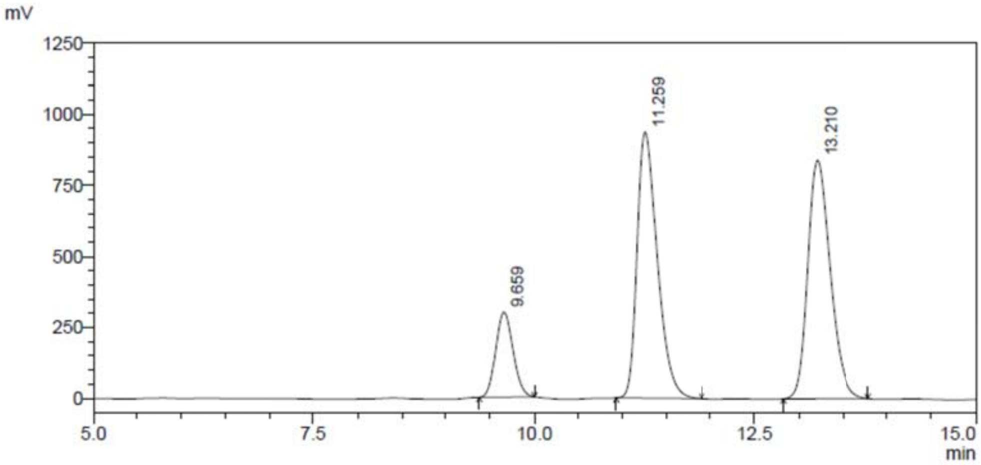

<Peak Table>

Detector A 215nm

Peak#	Ret. Time	Area	Height	Conc.	Unit	Mark	Name
1	9.659	4082776	300622	12.095		M	
2	11.259	14821145	937219	43.909		M	
3	13.210	14850677	839072	43.996		M	
Total		33754599	2076913				

Racemic **7e**

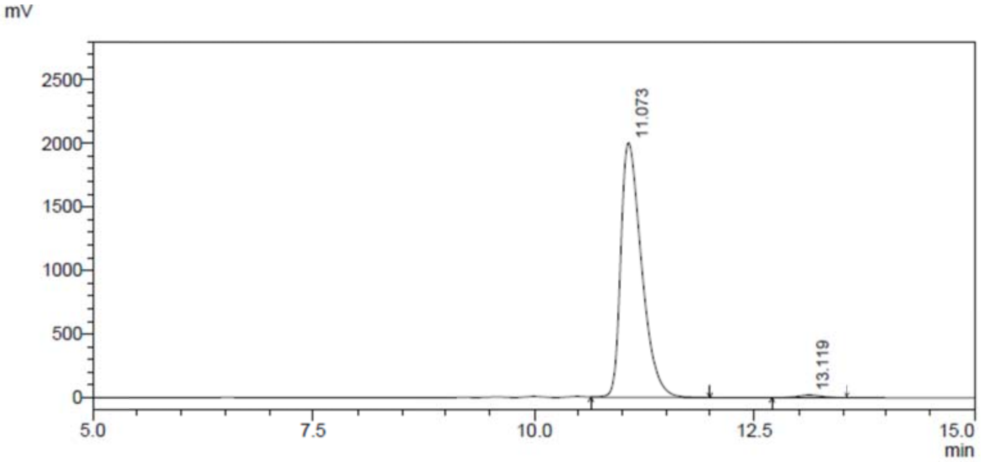

<Peak Table>

Detector A 215nm

Peak#	Ret. Time	Area	Height	Conc.	Unit	Mark	Name
1	11.073	32597243	2003819	98.935		M	
2	13.119	350913	20802	1.065		M	
Total		32948155	2024621				

Enantiomerically enriched **7e**

Supplementary Figure 77. HPLC spectra for compound **7e**

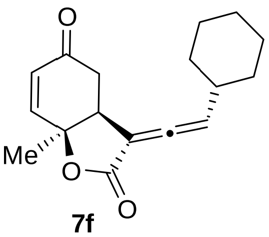

7f

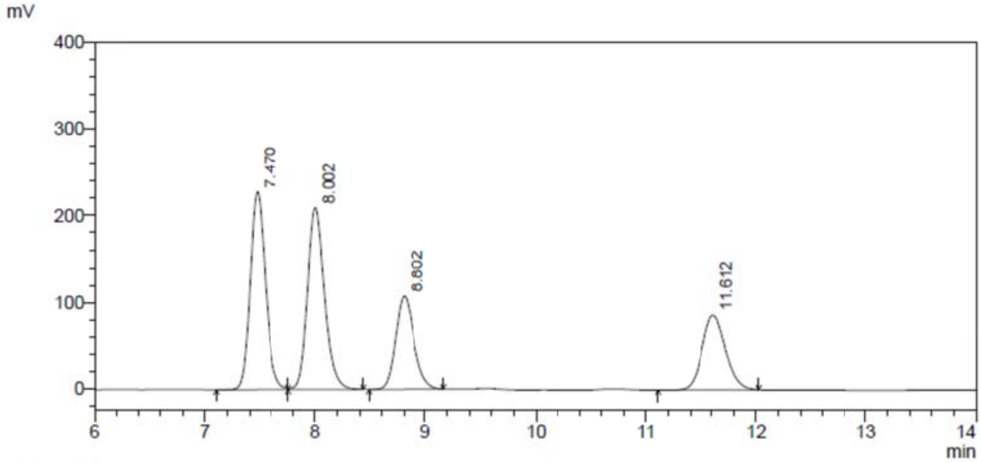

<Peak Table>

Detector A 215nm

Peak#	Ret. Time	Area	Height	Conc.	Unit	Mark	Name
1	7.470	2219144	228176	31.692		M	
2	8.002	2218776	209414	31.687		V M	
3	8.802	1277627	107777	18.246		M	
4	11.612	1286588	86579	18.374		M	
Total		7002135	631947				

Racemic **7f**

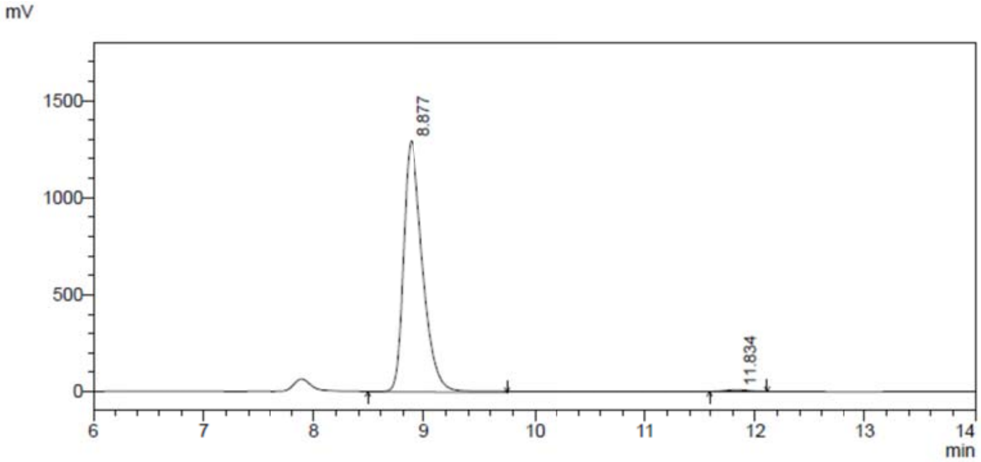

<Peak Table>

Detector A 215nm

Peak#	Ret. Time	Area	Height	Conc.	Unit	Mark	Name
1	8.877	16307037	1293874	99.090		M	
2	11.834	149684	9613	0.910		M	
Total		16456721	1303486				

Enantiomerically enriched **7f**

Supplementary Figure 78. HPLC spectra for compound **7f**

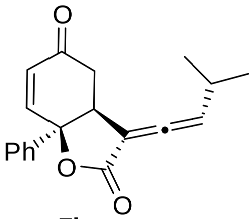

7h

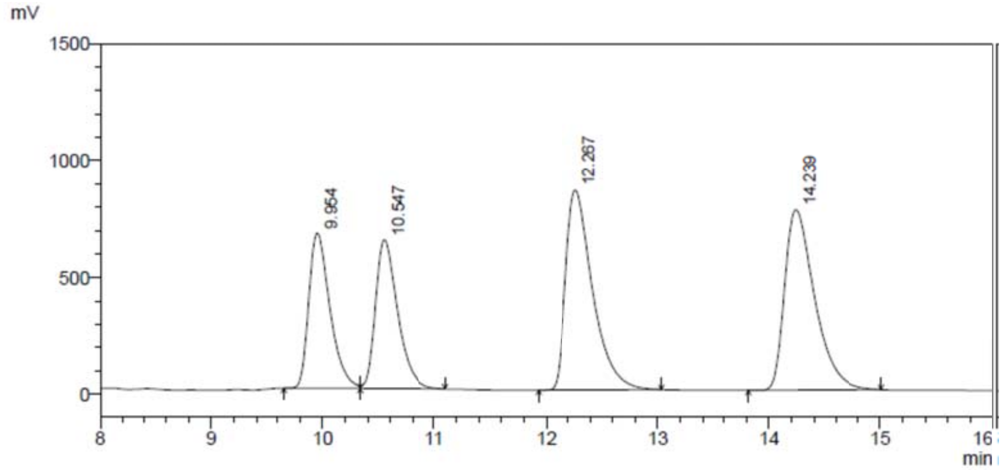

<Peak Table>

Detector A 215nm

Peak#	Ret. Time	Area	Height	Conc.	Unit	Mark	Name
1	9.954	8667369	663069	18.754		M	
2	10.547	8775069	636003	18.987		V M	
3	12.267	14383693	855225	31.123		M	
4	14.239	14390216	771214	31.137		M	
Total		46216347	2925511				

Racemic **7h**

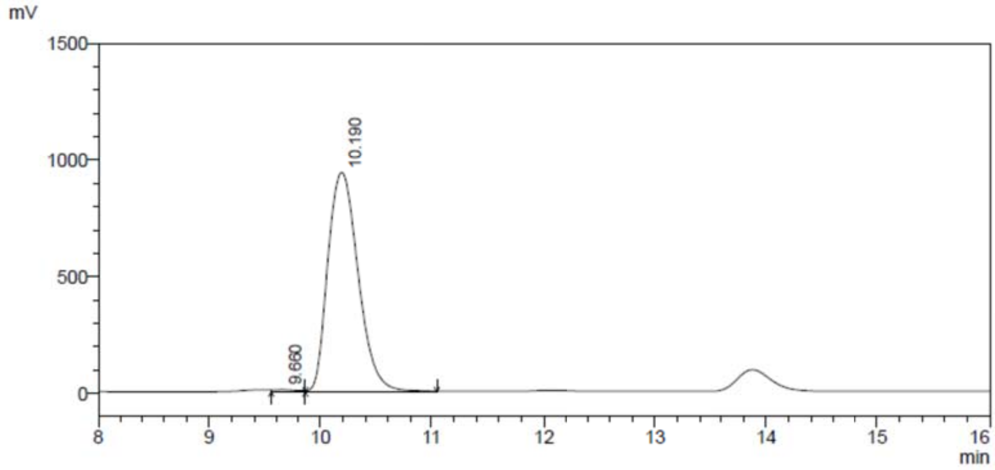

<Peak Table>

Detector A 215nm

Peak#	Ret. Time	Area	Height	Conc.	Unit	Mark	Name
1	9.660	138896	8822	0.781		M	
2	10.190	17649867	940053	99.219		V M	
Total		17788763	948875				

Enantiomerically enriched **7h**

Supplementary Figure 79. HPLC spectra for compound **7h**

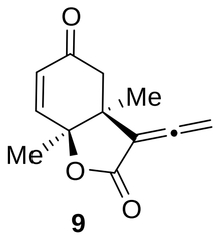

9

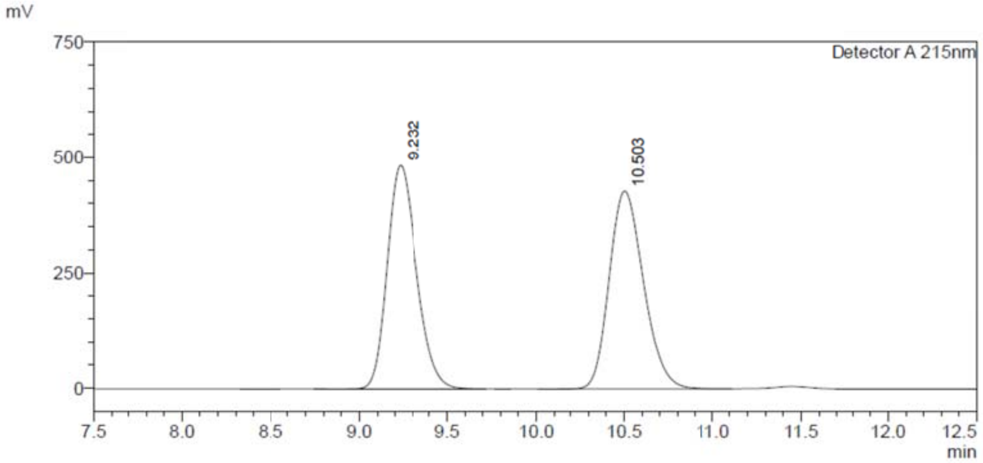

<Peak Table>

Detector A 215nm

Peak#	Ret. Time	Area	Height	Conc.	Unit	Mark	Name
1	9.232	5684877	484800	49.771		M	
2	10.503	5737298	428372	50.229		M	
Total		11422175	913173				

Racemic **9**

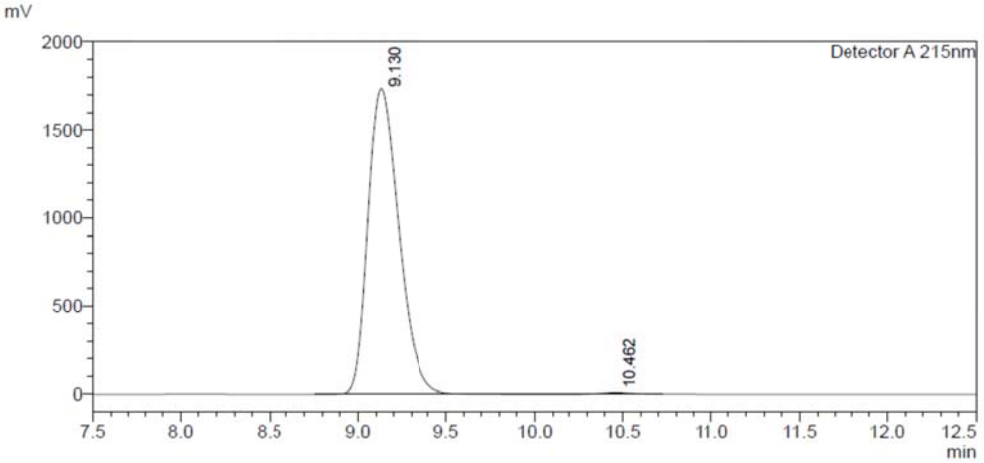

<Peak Table>

Detector A 215nm

Peak#	Ret. Time	Area	Height	Conc.	Unit	Mark	Name
1	9.130	21066981	1736499	99.468		M	
2	10.462	112607	8362	0.532		V M	
Total		21179587	1744861				

Enantiomerically enriched **9**

Supplementary Figure 80. HPLC spectra for compound **9**

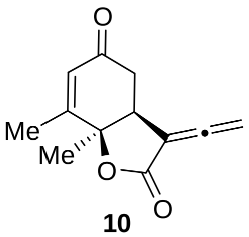

10

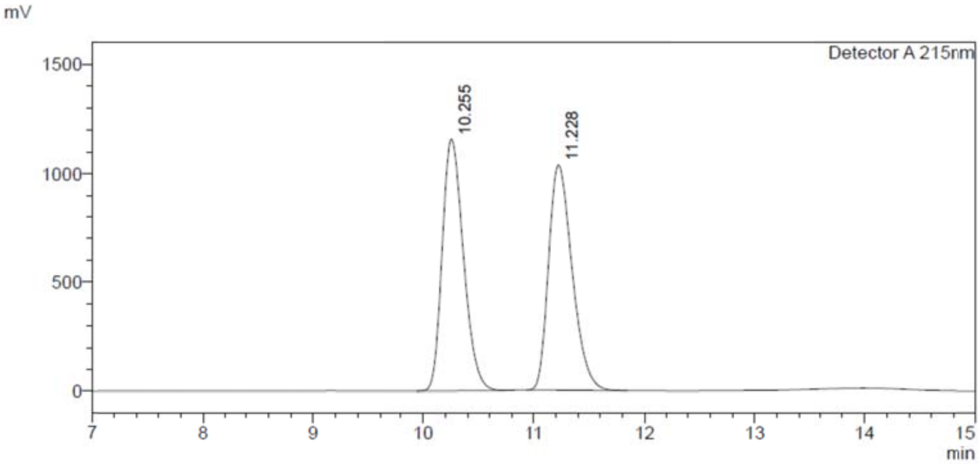

<Peak Table>

Detector A 215nm

Peak#	Ret. Time	Area	Height	Conc.	Unit	Mark	Name
1	10.255	15352731	1159757	50.107			
2	11.228	15287415	1036931	49.893			
Total		30640146	2196687				

Racemic **10**

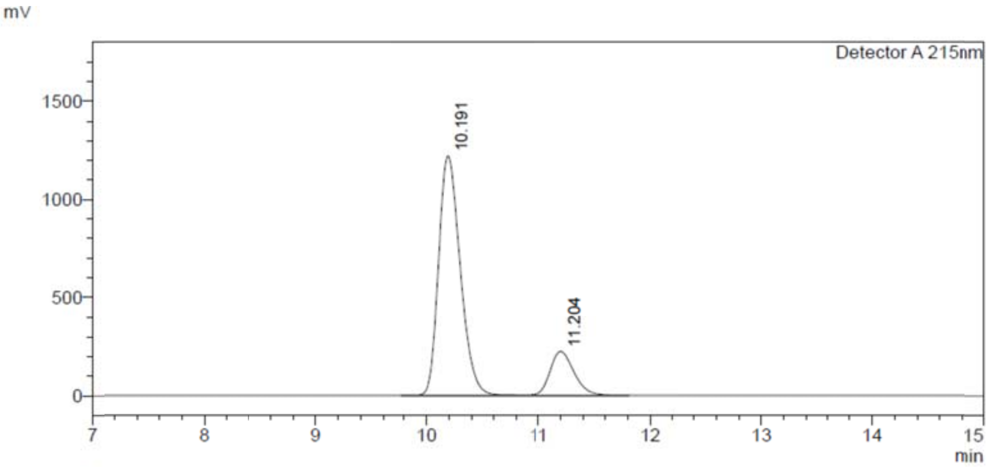

<Peak Table>

Detector A 215nm

Peak#	Ret. Time	Area	Height	Conc.	Unit	Mark	Name
1	10.191	16312402	1222884	83.336		M	
2	11.204	3261890	223795	16.664		V M	
Total		19574292	1446680				

Enantiomerically enriched **10**

Supplementary Figure 81. HPLC spectra for compound **10**

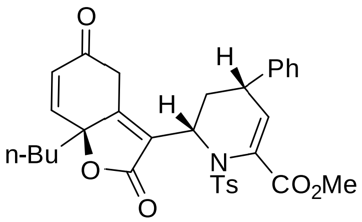

13

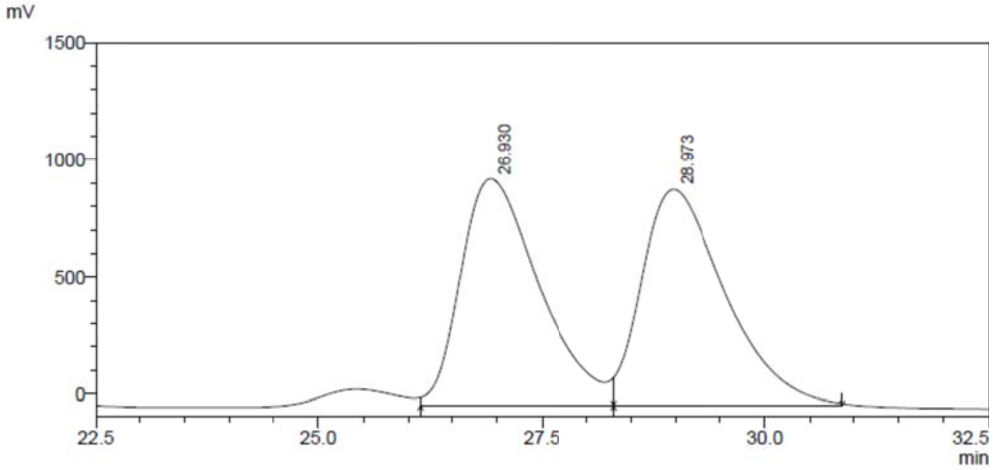

<Peak Table>

Detector A 215nm

Peak#	Ret. Time	Area	Height	Conc.	Unit	Mark	Name
1	26.930	59162101	968422	49.672		M	
2	28.973	59944015	922949	50.328		V M	
Total		119106117	1891371				

Racemic **13**

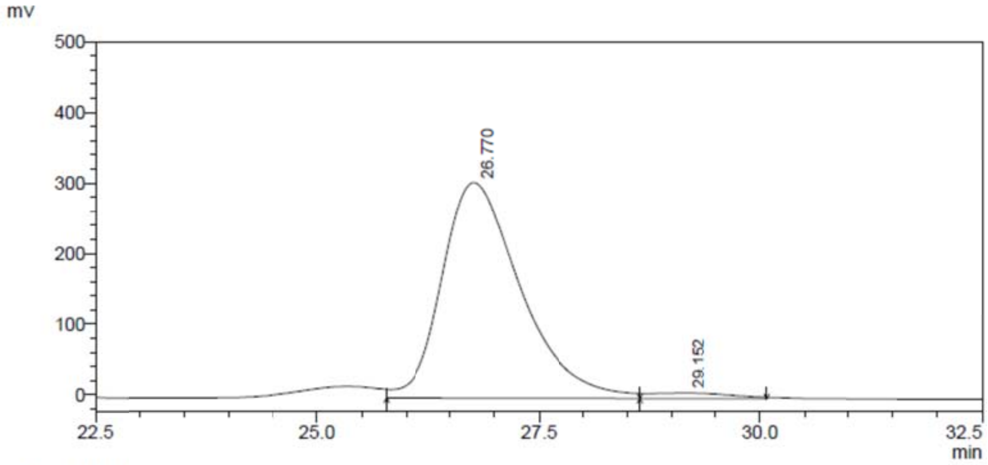

<Peak Table>

Detector A 215nm

Peak#	Ret. Time	Area	Height	Conc.	Unit	Mark	Name
1	26.770	18859589	305328	97.335		M	
2	29.152	516344	8023	2.665		V M	
Total		19375934	313352				

Enantiomerically enriched **13**

Supplementary Figure 82. HPLC spectra for compound **13**

Supplementary Table 1. Crystal data and structure refinement for **2a**

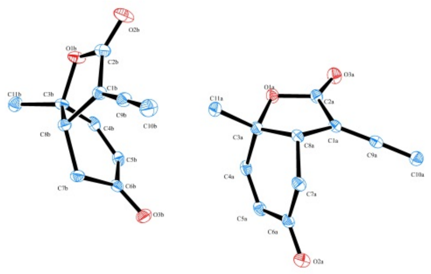

Empirical formula	C_{11} H_{10} O_3	
Formula weight	190.19	
Temperature	100(2) K	
Wavelength	1.54178 Å	
Crystal system	Triclinic	
Space group	P1	
Unit cell dimensions	a = 7.1339(4) Å	a= 82.4350(10)°.
	b = 7.3602(4) Å	b= 72.1990(10)°.
	c = 9.4972(6) Å	g = 81.3930(10)°.
Volume	467.49(5) $Å^3$	
Z	2	
Density (calculated)	1.351 Mg/m^3	
Absorption coefficient	0.816 mm^{-1}	
F(000)	200	
Crystal size	0.154 x 0.142 x 0.104 mm^3	
Theta range for data collection	4.911 to 72.411°.	
Index ranges	-8<=h<=8, -9<=k<=8, -11<=l<=11	
Reflections collected	12888	
Independent reflections	3494 [R(int) = 0.0208]	
Completeness to theta = 67.679°	98.9 %	
Absorption correction	Semi-empirical from equivalents	
Max. and min. transmission	0.920 and 0.885	
Refinement method	Full-matrix least-squares on F^2	
Data / restraints / parameters	3494 / 6 / 268	
Goodness-of-fit on F^2	1.073	
Final R indices [I>2sigma(I)]	R1 = 0.0246, wR2 = 0.0631	
R indices (all data)	R1 = 0.0246, wR2 = 0.0631	
Absolute structure parameter	0.07(2)	
Extinction coefficient	0.050(4)	
Largest diff. peak and hole	0.170 and -0.166 e.$Å^{-3}$	

Supplementary Table 2. Crystal data and structure refinement for **2n**

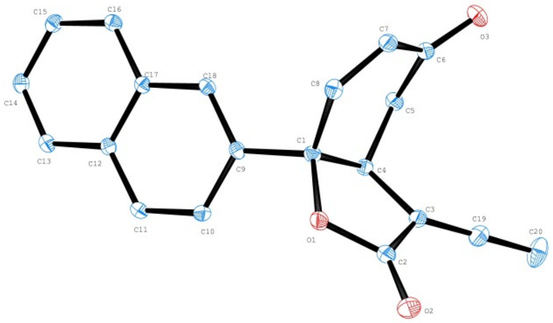

Empirical formula	C_{20} $H_{14}O_3$	
Formula weight	302.31	
Temperature	100(2) K	
Wavelength	1.54178 Å	
Crystal system	Orthorhombic	
Space group	$P2_12_12_1$	
Unit cell dimensions	a = 7.5440(3) Å	a= 90°.
	b = 9.7264(4) Å	b= 90°.
	c = 20.4603(10) Å	g = 90°.
Volume	1501.29(11) $Å^3$	
Z	4	
Density (calculated)	1.338 Mg/m^3	
Absorption coefficient	0.724 mm^{-1}	
F(000)	632	
Crystal size	0.277 x 0.230 x 0.128 mm^3	
Theta range for data collection	4.322 to 68.245°.	
Index ranges	-9<=h<=8, -11<=k<=11, -20<=l<=24	
Reflections collected	9213	
Independent reflections	2748 [R(int) = 0.0196]	
Completeness to theta = 67.679°	99.8 %	
Absorption correction	Semi-empirical from equivalents	
Max. and min. transmission	0.913 and 0.825	
Refinement method	Full-matrix least-squares on F^2	
Data / restraints / parameters	2748 / 1 / 215	
Goodness-of-fit on F^2	1.064	
Final R indices [I>2sigma(I)]	R1 = 0.0272, wR2 = 0.0702	
R indices (all data)	R1 = 0.0280, wR2 = 0.0709	
Absolute structure parameter	-0.06(6)	
Extinction coefficient	n/a	
Largest diff. peak and hole	0.203 and -0.180 e.$Å^{-3}$	

Supplementary Table 3. Crystal data and structure refinement for **7b**

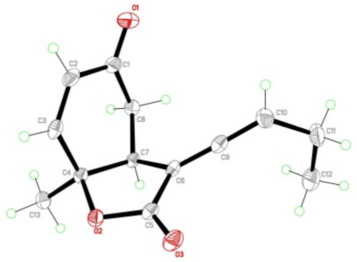

Empirical formula	$C_{14} H_{16}O_3$	
Formula weight	232.27	
Temperature	100(2) K	
Wavelength	1.54178 Å	
Crystal system	Orthorhombic	
Space group	$P2_12_12_1$	
Unit cell dimensions	a = 6.4002(4) Å	a= 90°.
	b = 7.3555(4) Å	b= 90°.
	c = 26.1963(15) Å	g = 90°.
Volume	1233.23(12) $Å^3$	
Z	4	
Density (calculated)	1.251 Mg/m^3	
Absorption coefficient	0.707 mm^{-1}	
F(000)	496	
Crystal size	0.471 x 0.372 x 0.210 mm^3	
Theta range for data collection	6.249 to 72.378°.	
Index ranges	-7<=h<=7, -9<=k<=6, -32<=l<=31	
Reflections collected	10988	
Independent reflections	2416 [R(int) = 0.0427]	
Completeness to theta = 67.679°	99.9 %	
Absorption correction	Semi-empirical from equivalents	
Max. and min. transmission	0.866 and 0.732	
Refinement method	Full-matrix least-squares on F^2	
Data / restraints / parameters	2416 / 0 / 173	
Goodness-of-fit on F^2	1.098	
Final R indices [I>2sigma(I)]	R1 = 0.0362, wR2 = 0.0905	
R indices (all data)	R1 = 0.0364, wR2 = 0.0909	
Absolute structure parameter	0.11(5)	
Extinction coefficient	n/a	
Largest diff. peak and hole	0.322 and -0.273 e.$Å^{-3}$	

Supplementary Note 1

Crystal data of all these crystals CCDC No. 1444859 (**2a**), 144860 (**2n**) and 1444861 (**7b**) were collected on a Bruker AXS D8 Venture equipped with a Photon 100 CMOS active pixel sensor detector using graphite-monochromated Cu-Kα radiation (λ = 1.54178 Å) using a sealed tube. Absorption corrections were made with the program SADABS[1], and the crystallographic package SHELXTL[2,3] was used for all calculations.

2a at 100(2) K: $C_{11}H_{10}O_3$, FW = 190.19, triclinic, $P1$; a = 7.1339(4) Å, b = 7.3602(4) Å, c = 9.4972(6) Å, α = 82.4350(10)°, β = 72.1990(10)°, γ = 81.3930(10)°, V = 467.49(5) Å^3, Z = 2, ρ_{calc} = 1.351 g·cm^{-3}, μ = 0.82 mm^{-1}, GOF = 1.073, final R_1 = 0.0246, wR_2 = 0.0631 [for 3492 data $I > 2\sigma(I)$]. Flack parameter, 0.09(14) and Parson, 0.066(23). There are two formula units in the asymmetric unit. The coordinates of the hydrogen atoms for C10A and C10B were refined with SADI option as the AFIX 93 did not work probably for the allylic group. However a riding model was used for the thermal parameters of the H atoms. The absolute structure parameter and chirality could not be determined reliably as the compound does not have heavier elements.

2n at 100(2) K: $C_{20}H_{14}O_3$, FW = 302.31, orthorhombic, $P2_12_12_1$; a = 7.5440(3) Å, b = 9.7264(4) Å, c = 20.4603(10) Å, V = 1501.29(11) Å^3, Z = 2, ρ_{calc} = 1.338 g·cm^{-3}, μ = 0.724 mm^{-1}, GOF = 1.064, final R_1 = 0.0280, wR_2 = 0.0709 [for 2689 data $I > 2\sigma(I)$]. Flack parameter, -0.12(24) and Parson, 0.058(57). The coordinates of the hydrogen atoms for C20 were refined with SADI option as the AFIX 93 did not work probably for the allylic group. However a riding model was used for the thermal parameters of the H atoms. The absolute structure parameter and chirality could not be determined reliably as the compound does not have heavier elements.

7b at 100(2) K: $C_{14}H_{16}O_3$, FW = 232.27, orthorhombic, $P2_12_12_1$; a = 6.4002(4) Å, b = 7.3555(4) Å, c = 20.4603(10) Å, V = 1233.23(12) Å^3, Z = 4, ρ_{calc} = 1.251 g·cm^{-3}, μ = 0.707 mm^{-1}, GOF = 1.098, final R_1 = 0.0364, wR_2 = 0.0909 [for 2399 data $I > 2\sigma(I)$]. Flack parameter, 0.11(5) and Parson, 0.048(27). The absolute structure parameter and chirality could not be determined reliably as the compound does not have heavier elements.

Supplementary Methods
General Information

Unless otherwise specified, all reactions were carried out under a nitrogen atmosphere, with dry, freshly distilled solvents in anhydrous conditions. THF, ether and toluene were distilled from sodium; while CH_2Cl_2 and MeCN were distilled from CaH_2 and ethyl acetate (Ea) and $CHCl_3$ were used without further purification. All chemicals were used without further purification as commercially available unless otherwise noted. Thin-layer chromatography (TLC) was performed on silica gel plates (60F-254) using UV-light (254 and 365 nm). Flash chromatography was conducted on silica gel (300–400 mesh). ^1H and ^{13}C NMR spectra were recorded on a Bruker AMX500 (500 MHz) spectrometer. Chemical shifts were reported in parts per million (ppm). All high resolution mass spectra were obtained on a Finnigan/MAT 95XL-T spectrometer. Optical rotations were measured using a Jasco DIP - 1000 polarimeter. Enantiomeric excesses were determined by HPLC analysis on a chiral stationary phase. The racemic sample was prepared by DABCO catalysis.

Representative procedure for the synthesis of allenoates 1, 6 and 8

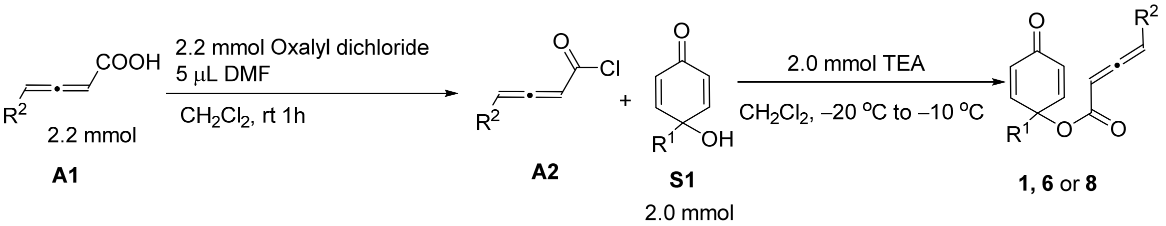

Allenoates **1, 6** and **8** were synthesized by reacting in-situ prepared allenic acid chloride **A2** with the corresponding *tert*-alcohol **S1**[4–8], which were easily accessible from the corresponding phenols. To a stirred solution of allenic acid **A1**[9] (2.2 mmol) in anhydrous CH_2Cl_2 (10 mL) under N_2 atmosphere at room temperature was added oxalyl chloride (2.2 mmol, 189 μL), followed by DMF (5 μL). The resulting mixture was stirred further 1 h and was used directly in the next step. To a flame dried round bottle flask with a magnetic stirring bar under N_2 were added *tert*-alcohol **S1** (2.0 mmol) and Et_3N (2.0 mmol, 278 μL), followed by anhydrous CH_2Cl_2 (10 mL). The resulting mixture was cooled to – 20 °C, and the above **A2** solution was added dropwise over 15 min under N_2 atmosphere. The reaction mixture was kept at – 20 °C for 1 h and then at – 10 °C for another 2 h. The reaction was then quenched with iced water, and extracted with CH_2Cl_2 (2× 30 mL). The combined organic extracts were washed by brine (50 mL) and concentrated. The residue was dissolved in EtOAc (40 mL) and washed by 0.1 N NaOH (20 mL), Brine (20 mL) successively. The organic phase was dried over Na_2SO_4, filtered and concentrated. The residue was purified directly by flash column chromatography to afford **1, 6** or **8** and recovered **S1**.

Analytical data of allenoates 1, 6 and 8

1-Methyl-4-oxocyclohexa-2,5-dien-1-yl buta-2,3-dienoate (**1a**)

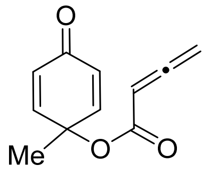

32% yield, white solid; ^{1}H NMR (500 MHz, $CDCl_3$) δ 6.91 (d, J = 10.1 Hz, 2H), 6.24 (d, J = 10.1 Hz, 2H), 5.60 (t, J = 6.5 Hz, 1H), 5.24 (d, J = 6.5 Hz, 2H), 1.58 (s, 3H); ^{13}C NMR (125 MHz, $CDCl_3$) δ 216.3, 185.0, 164.2, 149.0, 128.2, 87.7, 79.7, 74.7, 26.2. HRMS (ESI) m/z calcd for $C_{11}H_{10}NaO_3$ $[M+Na]^+$ = 213.0522, found = 213.0529.

2-Ethyl-4-oxocyclohexa-2,5-dien-1-yl buta-2,3-dienoate (**1b**)

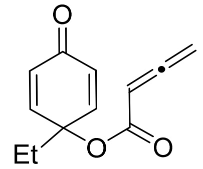

29% yield, white solid; ^{1}H NMR (500 MHz, $CDCl_3$) δ 6.78 (d, J = 10.2 Hz, 2H), 6.22 (d, J = 10.2 Hz, 2H), 5.54 (t, J = 6.5 Hz, 1H), 5.18 (d, J = 6.5 Hz, 2H), 1.83 (q, J = 7.5 Hz, 2H), 0.85 (t, J = 7.5 Hz, 3H); ^{13}C NMR (125 MHz, $CDCl_3$) δ 216.2, 185.2, 164.1, 148.1, 129.1, 87.7, 79.5, 77.6, 32.3, 7.6. HRMS (ESI) m/z calcd for $C_{12}H_{12}NaO_3$ $[M+Na]^+$ = 227.0679, found = 227.0685.

4-Oxo-1-propylcyclohexa-2,5-dien-1-yl buta-2,3-dienoate (**1c**)

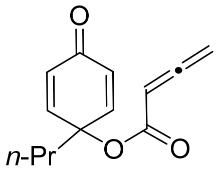

27% yield, Colorless oil; ^{1}H NMR (500 MHz, $CDCl_3$) δ 6.85 (d, J = 10.2 Hz, 2H), 6.26 (d, J = 10.2 Hz, 2H), 5.59 (t, J = 6.5 Hz, 1H), 5.23 (d, J = 6.6 Hz, 2H), 1.96 – 1.72 (m, 2H), 1.45 – 1.23 (m, 2H), 0.91 (t, J = 7.4 Hz, 3H); ^{13}C NMR (125 MHz, $CDCl_3$) δ 216.3, 185.3, 164.1, 148.3, 128.9, 87.7, 79.5, 77.4, 41.4, 16.8, 14.1. HRMS (ESI) m/z calcd for $C_{12}H_{12}NaO_3$ $[M+Na]^+$ = 241.0835, found = 241. 0827.

1-Isopropyl-4-oxocyclohexa-2,5-dien-1-yl buta-2,3-dienoate (**1d**)

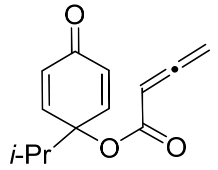

17% yield, Pale yellow oil; ^{1}H NMR (500 MHz, $CDCl_3$) δ 6.79 (d, J = 10.3 Hz, 2H), 6.32 (d, J = 10.3 Hz, 2H), 5.59 (t, J = 6.5 Hz, 1H), 5.23 (d, J = 6.5 Hz, 2H), 2.53 – 1.85 (m, 1H), 0.96 (d, J = 6.9 Hz, 6H); ^{13}C NMR (125 MHz, $CDCl_3$) δ 216.3, 185.4, 164.1, 147.1, 130.0, 87.8, 79.8, 79.5, 36.6, 16.9. HRMS (ESI) m/z calcd for $C_{13}H_{14}NaO_3$ $[M+Na]^+$ = 241.0835, found = 241.0829.

1-Butyl-4-oxocyclohexa-2,5-dien-1-yl buta-2,3-dienoate (**1e**)

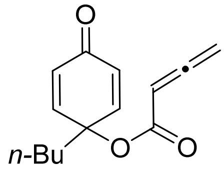

31% yield, pale yellow oil; ^{1}H NMR (500 MHz, $CDCl_3$) δ 6.84 (d, J = 1.8 Hz, 2H), 6.27 (d, J = 10.2 Hz, 2H), 5.60 (t, J = 6.5 Hz, 1H), 5.24 (d, J = 6.5 Hz, 2H), 1.93 – 1.74 (m, 2H), 1.37 – 1.21 (m, 4H), 0.88 (t, J = 7.0 Hz, 3H); ^{13}C NMR (125 MHz, $CDCl_3$) δ 216.3, 185.3, 164.1, 148.3, 129.0, 87.8, 79.5, 77.4, 39.0, 25.4, 22. 7, 13.8; HRMS (ESI) m/z calcd for $C_{14}H_{16}NaO_3$ $[M+Na]^+$ = 255.0992, found = 255.1003.

1-Benzyl-4-oxocyclohexa-2,5-dien-1-yl buta-2,3-dienoate (**1f**)

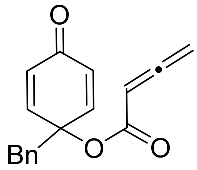

23% yield, white solid; ^{1}H NMR (500 MHz, $CDCl_3$) δ 7.30 – 7.26 (m, 3H), 7.22 – 7.17 (m, 2H), 6.80 (d, J = 10.2 Hz, 2H), 6.25 (d, J = 10.2 Hz, 2H), 5.58 (t, J = 6.5 Hz, 1H), 5.27 (d, J = 6.5 Hz, 2H), 3.07 (s, 2H); ^{13}C NMR (125 MHz, $CDCl_3$) δ 216.4, 184.9, 164.0, 148.3, 133.8, 131.0, 128.8, 128.2, 127.5, 87.7, 79.6, 76.5, 46.5. HRMS (ESI) m/z calcd for $C_{17}H_{14}NaO_3$ $[M+Na]^+$ = 289.0835, found = 289.0827.

4-Oxo-1-phenethylcyclohexa-2,5-dien-1-yl buta-2,3-dienoate (**1g**)

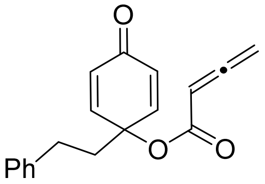

22% yield, pale yellow oil; ^{1}H NMR (500 MHz, $CDCl_3$) δ 7.31 – 7.25 (m, 2H), 7.23 – 7.18 (m, 1H), 7.16 – 7.10 (m, 2H), 6.91 (d, J = 10.2 Hz, 2H), 6.32 (d, J = 10.2 Hz, 2H), 5.62 (t, J = 6.5 Hz, 1H), 5.26 (d, J = 6.5 Hz, 2H), 2.73 – 2.62 (m, 2H), 2.19 – 2.09 (m, 2H); ^{13}C NMR (125 MHz, $CDCl_3$) δ 216.4, 185.1, 164.0, 148.0, 140.5, 129.2, 128.6, 128.3, 126.4, 87.7, 79.6, 76.9, 41.2, 29.9. HRMS (ESI) m/z calcd for $C_{18}H_{16}NaO_3$ $[M+Na]^+$ = 303.0992, found =303.0988.

1-(3-Methoxy-3-oxopropyl)-4-oxocyclohexa-2,5-dien-1-yl buta-2,3-dienoate (**1h**)

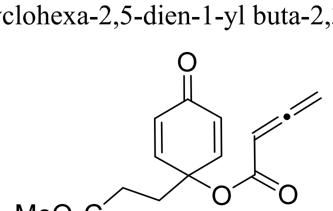

16% yield, pale yellow oil; ^{1}H NMR (500 MHz, $CDCl_3$) δ 6.80 (d, J = 10.0 Hz, 2H), 6.28 (d, J = 10.1 Hz, 2H), 5.58 (t, J = 6.5 Hz, 1H), 5.24 (d, J = 6.5 Hz, 2H), 3.65 (s, 3H), 2.33 (t, J = 7.8 Hz, 2H), 2.21 (dd, J = 8.7, 6.9 Hz, 2H); ^{13}C NMR (125 MHz, $CDCl_3$) δ 216.4, 184.8, 172.5, 163.9, 147.2, 129.8, 129.5, 87.5, 79.7, 76.3, 51.9, 33.9, 28.1; HRMS (ESI) m/z calcd for $C_{14}H_{14}NaO_5$$[M+Na]^+$ = 285.0733, found = 285.0742.

4-Oxo-[1,1'-biphenyl]-1(4H)-yl buta-2,3-dienoate (**1i**)

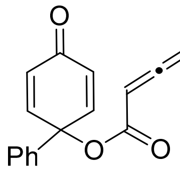

18% yield, pale yellow solid; ^{1}H NMR (500 MHz, CDCl$_3$) δ 7.48 – 7.43 (m, 2H), 7.41 – 7.31 (m, 3H), 6.97 (d, *J* = 10.1 Hz, 2H), 6.35 (d, *J* = 10.1 Hz, 2H), 5.71 (t, *J* = 6.5 Hz, 1H), 5.32 (d, *J* = 6.5 Hz, 2H); ^{13}C NMR (125 MHz, CDCl$_3$) δ 216.5, 185.5, 163.7, 147.5, 136.3, 129.1, 128.8, 128.4, 128.2, 125.3, 87.9, 79.8, 77.7; HRMS (ESI) m/z calcd for $C_{16}H_{11}O_3$ [M-H]$^-$ = 251.0714, found = 251.0704.

4'-Cyano-4-oxo-[1,1'-biphenyl]-1(4H)-yl buta-2,3-dienoate (**1j**)

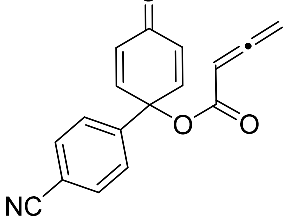

18% yield, pale yellow solid; ^{1}H NMR (500 MHz, CDCl$_3$) δ 7.68 (d, *J* = 8.3 Hz, 2H), 7.56 (d, *J* = 8.6 Hz, 2H), 6.92 (d, *J* = 10.1 Hz, 2H), 6.39 (d, *J* = 9.9 Hz, 2H), 5.71 (t, *J* = 6.5 Hz, 1H), 5.35 (d, *J* = 6.5 Hz, 2H); ^{13}C NMR (125 MHz, CDCl$_3$) δ 216.7, 184.8, 163.3, 146.1, 141.8, 132.9, 129.0, 126.2, 118.1, 112.8, 105.0, 87.7, 80.0, 77.1; HRMS (ESI) m/z calcd for $C_{17}H_{10}NO_3$ [M-H]$^-$ = 276.0666, found = 276.0674.

3'-Fluoro-4-oxo-[1,1'-biphenyl]-1(4H)-yl buta-2,3-dienoate (**1k**)

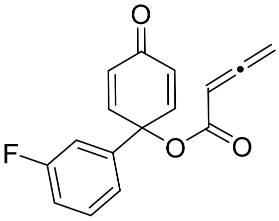

16% yield, pale yellow solid; ^{1}H NMR (500 MHz, CDCl$_3$) δ 7.33 (td, *J* = 8.2, 5.9 Hz, 1H), 7.18 (ddd, *J* = 7.2, 4.2, 2.0 Hz, 2H), 7.05 – 6.99 (m, 1H), 6.93 (d, *J* = 10.1 Hz, 2H), 6.34 (d, *J* = 10.1 Hz, 2H), 5.70 (t, *J* = 6.5 Hz, 1H), 5.33 (d, *J* = 6.5 Hz, 2H); ^{13}C NMR (125 MHz, CDCl$_3$) δ 216.6, 185.2, 163.1 (d, J = 247.4 Hz), 163.5, 146.8, 139.0 (d, *J* = 7.3 Hz), 130.7 (d, *J* = 8.2 Hz), 128.5, 121.0 (d, *J* = 3.0 Hz), 115.8 (d, *J* = 21.1 Hz), 112.8 (d, *J* = 24.1 Hz), 87.8, 79.9; HRMS (ESI) m/z calcd for $C_{16}H_{10}FO_3$ [M-H]$^-$ = 269.0619, found = 269.0610.

4'-Chloro-4-oxo-[1,1'-biphenyl]-1(4H)-yl buta-2,3-dienoate (**1l**)

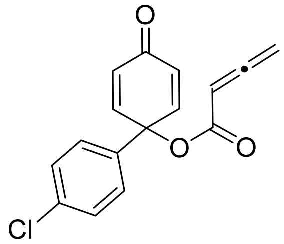

21% yield, pale yellow solid; ^{1}H NMR (500 MHz, CDCl$_3$) δ 7.52 – 7.32m, 4), 6.93 (d, *J* = 10.1 Hz, 2), 6.52 – 6.14 (m, 2), 5.70 (t, *J* = 6.5 Hz, 1H), 5.32 (d, *J* = 6.5 Hz, 2); ^{13}C NMR (125MHz, CDCl$_3$) δ 216.6 185.2, 163.5, 147.0, 135.0, 134.8, 129.3, 128.4, 126. 8, 87.8, 79.9, 77.2; HRMS (ESI) m/z calcd for $C_{16}H_{11}ClNaO_3$ $[M+Na]^+$ = 309.0289, found = 309.0301.

2'-Methyl-4-oxo-[1,1'-biphenyl]-1(4H)-yl buta-2,3-dienoate (**1m**)

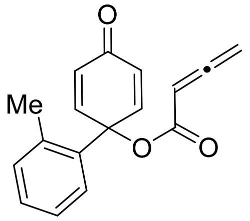

14% yield, pale yellow oil; ^{1}H NMR (500 MHz, CDCl$_3$) δ 7.36 (d, *J* = 7.8 Hz, 1H), 7.28 – 7.13 (m, 5H), 6.36 (d, *J* = 10.2 Hz, 2H), 5.66 (t, *J* = 6.5 Hz, 1H), 5.27 (d, *J* = 6.5 Hz, 2H), 2.52 (s, 3H); ^{13}C NMR (125 MHz, CDCl$_3$) δ 216.4, 185.2, 163.5, 145.5, 136.2, 134.7, 133.3, 129.1, 128.9, 126.6, 125.9, 87.9, 79.8, 78.6, 21.6; HRMS (ESI) m/z calcd for $C_{17}H_{14}NaO_3$ $[M+Na]^+$ = 289.0835, found = 289.0844.

1-(Naphthalen-2-yl)-4-oxocyclohexa-2,5-dien-1-yl buta-2,3-dienoate (**1n**)

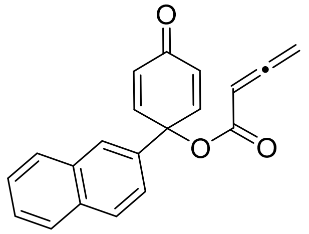

15% yield, pale yellow solid; ^{1}H NMR (500 MHz, CDCl$_3$) δ 7.97 (d, *J* = 1.2 Hz, 1H), 7.86 (d, *J* = 8.8 Hz, 1H), 7.82 (dd, *J* = 6.1, 3.4 Hz, 2H), 7.55 – 7.49 (m, 2H), 7.47 (dd, *J* = 8.7, 1.9 Hz, 1H), 7.05 (d, *J* = 10.1 Hz, 2H), 6.40 (d, *J* = 10.1 Hz, 2H), 5.77 (t, *J* = 6.5 Hz, 1H), 5.36 (d, *J* = 6.5 Hz, 2H); ^{13}C NMR (125 MHz, CDCl$_3$) δ 216.6, 185.6, 163.7, 147.4, 133.4, 133.3, 133.1, 129.1, 128.4, 128.3, 127.7, 126.9, 126.8, 124.7, 122.7, 88.0, 79.8, 77.8; HRMS (ESI) m/z calcd for $C_{20}H_{14}NaO_3$ $[M+Na]^+$ =325.0835, found = 325.0843.

1-Ethynyl-4-oxocyclohexa-2,5-dien-1-yl buta-2,3-dienoate (**1o**)

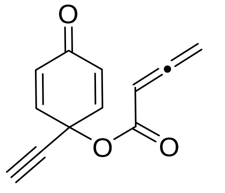

28% yield, white solid; ^{1}H NMR (500 MHz, CDCl$_3$) δ 7.09 (d, *J* = 10.1 Hz, 2H), 6.29 (d, *J* = 10.1 Hz, 2H), 5.63 (t, *J* = 6.5 Hz, 1H), 5.28 (d, *J* = 6.5 Hz, 2H), 2.72 (s, 1H); ^{13}C NMR (125 MHz, CDCl$_3$) δ 216.6, 184.2, 163.5, 142.4, 128.7, 87.5, 79.9, 76.9, 76.4, 67.6; HRMS (ESI) m/z calcd for $C_{12}H_7O_3$ $[M-H]^-$ = 199.0401, found = 199.0390.

1,2,6-Trimethyl-4-oxocyclohexa-2,5-dien-1-yl buta-2,3-dienoate (**1p**)

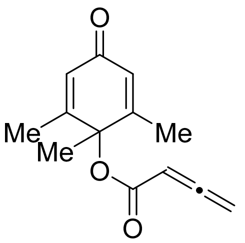

31% yield, white solid; ^{1}H NMR (500 MHz, $CDCl_3$) δ 6.06 (s, 2H), 5.64 (t, *J* = 6.5 Hz, 1H), 5.26 (d, *J* = 6.5 Hz, 2H), 1.93 (s, 6H), 1.50 (s, 3H); ^{13}C NMR (125 MHz, $CDCl_3$) δ 216.5, 185.3, 163.6, 159.5, 126.7, 87.1, 79.7, 79.1, 26.3, 17.7; HRMS (ESI) m/z calcd for $C_{13}H_{14}NaO_3$ $[M+Na]^+$ =241.0835, found = 241.0833.

1-Methyl-4-oxocyclohexa-2,5-dien-1-yl penta-2,3-dienoate (**6a**)

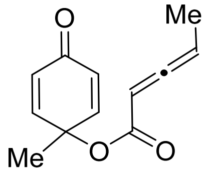

21% yield, white solid; ^{1}H NMR (500 MHz, $CDCl_3$) δ 6.90 (d, *J* = 10.2 Hz, 2H), 6.22 (dd, *J* = 10.0, 0.9 Hz, 2H), 5.61 (qd, *J* = 7.4, 6.3 Hz, 1H), 5.56 – 5.45 (m, 1H), 1.77 (dd, *J* = 7.4, 3.2 Hz, 3H), 1.56 (s, 3H); ^{13}C NMR (125 MHz, $CDCl_3$) δ 213.7, 185.1, 164.7, 149.2, 128.1, 128.1, 90.8, 87.3, 74.5, 26.3, 12.6; HRMS (ESI) m/z calcd for $C_{12}H_{12}NaO_3$ $[M+Na]^+$ =227.0679, found = 227.0674.

1-Methyl-4-oxocyclohexa-2,5-dien-1-yl hexa-2,3-dienoate (**6b**)

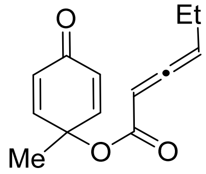

19% yield, white solid; ^{1}H NMR (500 MHz, $CDCl_3$) δ 6.99 – 6.73 (m, 2H), 6.22 (q, *J* = 2.8 Hz, 2H), 5.69 (q, *J* = 6.4 Hz, 1H), 5.59 – 5.45 (m, 1H), 2.14 (pd, *J* = 7.3, 3.2 Hz, 2H), 1.56 (s, 3H), 1.06 (t, *J* = 7.4 Hz, 3H); ^{13}C NMR (125 MHz, $CDCl_3$) δ 212.9, 185.1, 164.8, 149.2, 149.2, 128.1, 97.6, 88.4, 74.4, 26.3, 20. 8, 13.2; HRMS (ESI) m/z calcd for $C_{13}H_{14}NaO_3$ $[M+Na]^+$ =241.0835, found = 241.0844.

1-Methyl-4-oxocyclohexa-2,5-dien-1-yl 5-methylhexa-2,3-dienoate (**6c**)

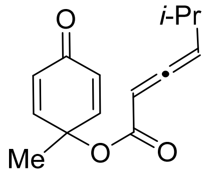

15% yield, white solid; ^{1}H NMR (500 MHz, $CDCl_3$) δ 6.91 (ddd, *J* = 10.6, 9.0, 3.5 Hz, 2H), 6.26 – 6.17 (m, 2H), 5.65 (t, *J* = 6.1 Hz, 1H), 5.57 (dd, *J* = 6.1, 3.0 Hz, 1H), 2.47 (dqd, *J* = 13.3, 6.7, 3.0 Hz, 1H), 1.56 (s, 3H), 1.08 (dd, *J* = 6.7, 0.8 Hz, 6H); ^{13}C NMR (125 MHz, $CDCl_3$) δ 212.0, 185.1, 164.8, 149.3, 128.1, 128.1, 103.0, 88.8, 74.4, 27.7, 26.3, 22.3, 22.3; HRMS (ESI) m/z calcd for $C_{14}H_{16}NaO_3$ $[M+Na]^+$ =255.0992, found = 255.1003.

1-Methyl-4-oxocyclohexa-2,5-dien-1-yl octa-2,3-dienoate (**6d**)

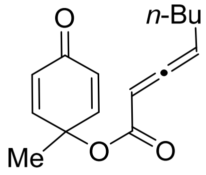

18% yield, pale yellow oil; ^{1}H NMR (500 MHz, CDCl$_3$) δ 6.90 (ddd, *J* = 9.6, 7.7, 3.5 Hz, 2H), 6.23 (d, *J* = 9.7 Hz, 2H), 5.62 (dd, *J* = 13.3, 7.1 Hz, 1H), 5.52 (dt, *J* = 6.0, 2.9 Hz, 1H), 2.13 (qd, *J* = 7.1, 3.0 Hz, 2H), 1.56 (s, 3H), 1.47 – 1.41 (m, 2H), 1.38 (dt, *J* = 13.6, 6.7 Hz, 2H), 0.90 (t, *J* = 7.2 Hz, 3H); ^{13}C NMR (125 MHz, CDCl$_3$) δ 213.0, 185.1, 164.8, 149.2, 128.1, 95.8, 87.8, 74.4, 30.8, 27.1, 26.3, 22.0, 13.8; HRMS (ESI) m/z calcd for $C_{15}H_{18}NaO_3$ [M+Na]$^+$ = 269.1148, found = 269.1143.

1-Methyl-4-oxocyclohexa-2,5-dien-1-yl 6-phenylhexa-2,3-dienoate (**6e**)

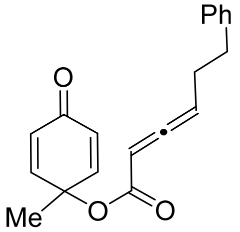

15% yield, pale yellow oil;^{1}H NMR (500 MHz, CDCl$_3$) δ 7.29 (t, *J* = 7.5 Hz, 2H), 7.23 – 7.17 (m, 3H), 6.99 – 6.75 (m, 2H), 6.24 (dd, *J* = 9.9, 1.6 Hz, 2H), 5.68 (q, *J* = 6.8 Hz, 1H), 5.55 (dt, *J* = 6.0, 2.9 Hz, 1H), 3.01 – 2.63 (m, 2H), 2.58 – 2.27 (m, 2H), 1.56 (s, 3H); ^{13}C NMR (125 MHz, CDCl$_3$) δ 213.0, 185.0, 164.6, 149.2, 140.8, 128.5, 128.4, 128.1, 126.2, 95.2, 88.2, 74.5, 35.1, 29.0, 26.3; HRMS (ESI) m/z calcd for $C_{19}H_{17}O_3$ [M-H]$^-$ = 293.1183, found = 293.1192.

1-Methyl-4-oxocyclohexa-2,5-dien-1-yl 4-cyclohexylbuta-2,3-dienoate (**6f**)

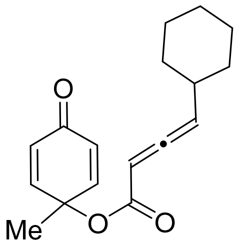

15% yield, colorless oil; ^{1}H NMR (500 MHz, CDCl$_3$) δ 6.94 – 6.85 (m, 2H), 6.21 (dd, *J* = 10.0, 1.4 Hz, 2H), 5.60 (t, *J* = 6.1 Hz, 1H), 5.54 (dd, *J* = 6.1, 3.0 Hz, 1H), 2.28 – 2.07 (m, 1H), 1.80 – 1.75 (m, 2H), 1.74 – 1.67 (m, 2H), 1.61 (dd, *J* = 9.1, 3.4 Hz, 1H), 1.54 (d, *J* = 7.2 Hz, 3H), 1.33 – 1.03 (m, 5H); ^{13}C NMR (125 MHz, CDCl$_3$) δ 212.4, 185.0, 164.8, 149.3, 128.1, 101.5, 88.5, 74.3, 36.6, 32.7, 32.7, 26.3, 25.9, 25.7, 25.7; HRMS (ESI) m/z calcd for $C_{17}H_{20}NaO_3$ [M+Na]$^+$ = 295.1305, found = 295.1296.

1-Methyl-4-oxocyclohexa-2,5-dien-1-yl 5,5-dimethylhexa-2,3-dienoate (**6g**)

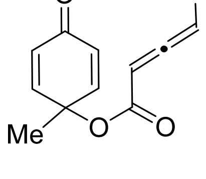

11% yield, colorless oil; ^{1}H NMR (500 MHz, $CDCl_3$) δ 6.98 – 6.81 (m, 2H), 6.22 (dd, *J* = 10.3, 1.6 Hz, 2H), 5.59 (dd, *J* = 24.8, 6.0 Hz, 2H), 1.55 (s, 3H), 1.10 (s, 9H); ^{13}C NMR (125 MHz, $CDCl_3$) δ 211.2, 185.0, 164.7, 149.3, 149.2, 128.1, 128.1, 107.2, 89.3, 74.3, 32.8, 30.0, 26.3; HRMS (ESI) m/z calcd for $C_{15}H_{18}NaO_3$ $[M+Na]^+$ = 269.1148, found = 269.1139. Optical resolution was carried out by use of a chiral stationary phase column [Chiralpak IA (2.0 cm I.D. × 25 cm), hexane/CH_2Cl_2 = 3:1, flow 8 mL/min, five cycles, fifth cycle: t_2= 102-105 min to get optically pure **6g-1**. with ee value >99%, t_R (major) =12.9 min (Chiralpak IE, λ = 254 nm, 10% *i*-PrOH/hexane, flow rate = 1.0 mL/min).

4-Oxo-[1,1'-biphenyl]-1(4H)-yl 5-methylhexa-2,3-dienoate (**6h**)

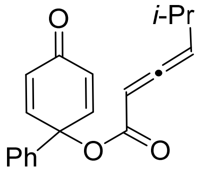

15% yield, pale yellow solid; ^{1}H NMR (500 MHz, $CDCl_3$) δ 7.55 – 7.42 (m, 2H), 7.41 – 7.31 (m, 3H), 7.02 – 6.85 (m, 1H), 6.39 – 6.20 (m, 1H), 5.80 – 5.62 (m, 1H), 2.62 – 2.47 (m, 1H), 1.12 (ddd, *J* = 6.8, 3.1, 1.1 Hz, 6H); ^{13}C NMR (125 MHz, $CDCl_3$) δ 212.1, 185.6, 164.2, 147.8, 147.8, 136.4, 129.0, 128.7, 128.0, 128.0, 125.4, 103.0, 89.101, 77.4, 27.8, 22.4, 22.2; HRMS (ESI) m/z calcd for $C_{19}H_{18}NaO_3$ $[M+Na]^+$ = 317.1148, found = 317.1154.

1,2-Dimethyl-4-oxocyclohexa-2,5-dien-1-yl buta-2,3-dienoate (**8**)

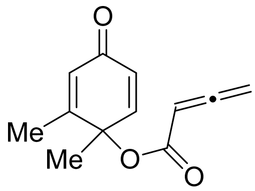

28% yield, white solid; ^{1}H NMR (500 MHz, CDCl3) δ 6.85 (d, J = 10.1 Hz, 1H), 6.19 (dd, J = 10.1, 1.9 Hz, 1H), 6.09 – 6.02 (m, 1H), 5.60 (t, J = 6.5 Hz, 1H), 5.23 (d, J = 6.5 Hz, 2H), 1.92 (d, J = 1.3 Hz, 3H), 1.50 (s, 3H); ^{13}C NMR (125 MHz, CDCl3) δ 216.4, 185.4, 163.8, 158.9, 149.7, 127.7, 126.8, 87.4, 79.6, 76.7, 26.1, 17.7; HRMS (ESI) m/z calcd for $C_{12}H_{12}NaO_3$ $[M+Na]^+$ = 227.0679, found = 227.0668.

Representative procedure of the intramolecular allenoate Rauhut–Currier reaction

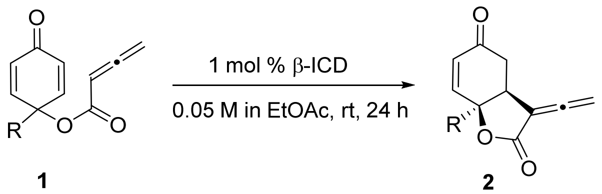

1 **2**

To a flame dried round bottle flask with a magnetic stirring bar at room temperature under N_2 were added allenoate **1** (0.15 mmol) and EtOAc (3 mL), followed by the addition of β-ICD (1 mol %, 0.5 mg). The resulting mixture was stirred for 24 h. The solvent was removed under reduced pressure and the residue was purified by column chromatography on silica gel to afford annulation adduct **2**.

Analytical data products 2

(3aS,7aS)-7a-Methyl-3-vinylidene-3a,7a-dihydrobenzofuran-2,5(3H,4H)-dione (**2a**)

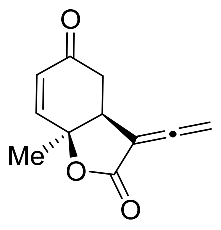

White solid, $[\alpha]^{25}_D$ = +122 (c 1.0, EtOAc); ^{1}H NMR (500 MHz, $CDCl_3$) δ 6.76 – 6.40 (m, 1H), 6.03 (d, *J* = 10.3 Hz, 1H), 5.49 (d, *J* = 6.2 Hz, 2H), 3.58 – 3.37 (m, 1H), 2.86 – 2.58 (m, 2H), 1.72 (s, 3H); ^{13}C NMR (125 MHz, $CDCl_3$) δ 208.2, 194.4, 167.3, 146.4, 128.9, 98.8, 85.0, 80.6, 44.1, 35.9, 23.9; HRMS (ESI) m/z calcd for $C_{11}H_9O_3$ $[M-H]^-$ = 189.0557, found = 189.0555. The ee value was 96%, t_R (major) = 22.08 min, t_R (minor) = 25.12 min (Chiralpak IE, λ = 215 nm, 20% *i*-PrOH/hexane, flow rate = 1.0 mL/min).

(3aS,7aS)-7a-Ethyl-3-vinylidene-3a,7a-dihydrobenzofuran-2,5(3H,4H)-dione (**2b**)

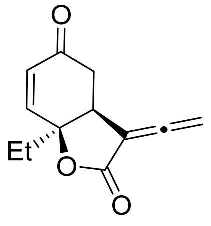

White solid, $[\alpha]^{25}_D$ = +113 (c 1.0, EtOAc); ^{1}H NMR (500 MHz, $CDCl_3$) δ 6.61 (dd, *J* = 10.4, 1.7 Hz, 1H), 6.08 (d, *J* = 10.4 Hz, 1H), 5.48 (dd, *J* = 6.2, 1.6 Hz, 2H), 3.58 (dtd, *J* = 6.0, 4.0, 1.8 Hz, 1H), 2.71 (d, *J* = 4.2 Hz, 2H), 2.22 – 1.80 (m, 2H), 1.11 (t, *J* = 7.5 Hz, 3H); ^{13}C NMR (125 MHz, $CDCl_3$) δ 208.3, 194. 7, 167.4, 145.6, 129.8, 99.1, 85.0, 83.1, 41.6, 36.4, 30.5, 7.9; HRMS (ESI) m/z calcd for $C_{12}H_{12}NaO_3$ $[M+Na]^+$ =227.0679, found = 227.0688. The ee value was 98%, t_R (major) = 11.12 min, t_R (minor) = 12.43 min (Chiralpak IE, λ = 215 nm, 35% *i*-PrOH/hexane, flow rate = 1.0 mL/min).

(3aS,7aS)-7a-Propyl-3-vinylidene-3a,7a-dihydrobenzofuran-2,5(3H,4H)-dione (**2c**)

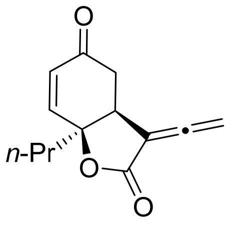

Colorless oil, $[\alpha]^{25}_D$ = +101 (c 0.6, EtOAc);^{1}H NMR (500 MHz, $CDCl_3$) δ 6.60 (dd, *J* = 10.4, 1.1 Hz, 1H), 6.05 (d, *J* = 10.4 Hz, 1H), 5.46 (d, *J* = 6.2 Hz, 2H), 3.81 – 3.26 (m, 1H), 2.87 – 2.51 (m, 2H), 2.02 – 1.85 (m, 2H), 1.59 – 1.49 (m, 2H), 1.00 (t, *J* = 7.3 Hz, 3H); ^{13}C NMR (125 MHz, $CDCl_3$) δ 208.3, 194.7, 167.4, 145.8, 129.6, 99.0, 85.0, 82.9, 42.1, 39.8, 36.3, 17.0, 14.3; HRMS (ESI) m/z calcd for $C_{13}H_{14}NaO_3$ $[M+Na]^+$

=241.0835, found = 241.0827. The ee value was 96%, t_R (minor) = 18.14 min, t_R (major) = 19.73 min (Chiralpak IA, λ = 215 nm, 5% *i*-PrOH/hexane, flow rate = 1.0 mL/min).

(3aS, 7aS)-7a-Isopropyl-3-vinylidene-3a,7a-dihydrobenzofuran-2,5(3H,4H)-dione (**2d**)

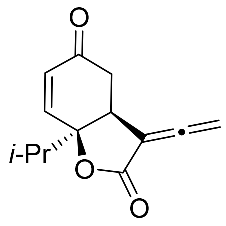

Colorless oil, $[\alpha]^{25}_D$ = +83 (c 1.0, EtOAc); ^{1}H NMR (500 MHz, $CDCl_3$) δ 6.61 (dd, *J* = 10.5, 1.7 Hz, 1H), 6.14 (d, *J* = 10.5 Hz, 1H), 5.55 – 5.40 (m, 2H), 3.77 – 3.51 (m, 1H), 2.80 – 2.59 (m, 2H), 2.24 (hept, *J* = 6.9 Hz, 1H), 1.10 (t, *J* = 6.9 Hz, 6H); ^{13}C NMR (125 MHz, $CDCl_3$) δ 208.5, 194.9, 167.3, 144.8, 130.7, 99.8, 85.3, 85.1, 39.3, 37.5, 35.8, 17.4, 16.6; HRMS (ESI) m/z calcd for $C_{13}H_{14}NaO_3$ $[M+Na]^+$ =241.0835, found = 241.0843. The ee value was 91%, t_R (major) = 10.48 min, t_R (minor) = 11.68 min (Chiralpak IE, λ = 215 nm, 35% *i*-PrOH/hexane, flow rate = 1.0 mL/min).

(3aS,7aS)-7a-Butyl-3-vinylidene-3a,7a-dihydrobenzofuran-2,5(3H,4H)-dione (**2e**)

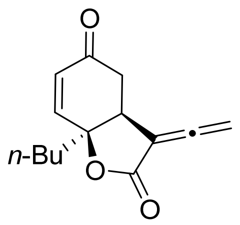

Colorless oil, $[\alpha]^{25}_D$ = +73 (c 1.0, EtOAc); ^{1}H NMR (500 MHz, $CDCl_3$) δ 6.61 (dd, *J* = 10.4, 1.7 Hz, 1H), 6.08 (d, *J* = 10.4 Hz, 1H), 5.71 – 5.23 (m, 2H), 3.73 – 3.37 (m, 1H), 3.03 – 2.57 (m, 2H), 2.05 – 1.97 (m, 1H), 1.96 – 1.89 (m, 1H), 1.54 – 1.46 (m, 2H), 1.45 – 1.37 (m, 2H), 0.95 (t, *J* = 7.2 Hz, 3H); ^{13}C NMR (125 MHz, $CDCl_3$) δ 208.3, 194.7, 167.4, 145.9, 129.6, 99.1, 85.0, 82.9, 42.1, 37.4, 36.4, 25.6, 22.9, 13.8; HRMS (ESI) m/z calcd for $C_{14}H_{15}O_3$ $[M-H]^-$ = 231.1027, found = 231.1015. The ee value was 96%, t_R (minor) = 12.23 min, t_R (major) = 13.05 min (Chiralpak IB, λ = 215 nm, 15% *i*-PrOH/hexane, flow rate = 1.0 mL/min).

(3aS, 7aS)-7a-Benzyl-3-vinylidene-3a,7a-dihydrobenzofuran-2,5(3H,4H)-dione (**2f**)

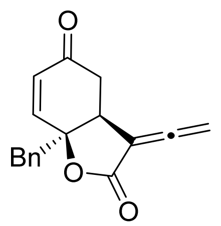

White solid, $[\alpha]^{25}_D$ = +39 (c 1.0, EtOAc); ^{1}H NMR (500 MHz, $CDCl_3$) δ 7.36 – 7.28 (m, 3H), 7.28 – 7.21 (m, 2H), 6.58 (dt, *J* = 10.4, 1.5 Hz, 1H), 6.05 (d, *J* = 10.4 Hz, 1H), 5.70 – 5.19 (m, 2H), 3.60 (dt, *J* = 8.2, 6.0 Hz,

1H), 3.36 (dd, J = 20.2, 14.1, 2H), 2.66 – 2.43 (m, 1H), 2.30 (ddd, J = 17.2, 5.8, 1.1 Hz, 1H); ^{13}C NMR (125 MHz, $CDCl_3$) δ 208.3, 194.6, 167.2, 145.5, 133.3, 130.4, 129.8, 128.8, 127.9, 98.8, 85.1, 82.5, 43.4, 41.4, 36.0; HRMS (ESI) m/z calcd for $C_{17}H_{13}O_3$ $[M-H]^-$ = 265.0870, found = 265.0878. The ee value was 94%, t_R (major) = 11.24 min, t_R (minor) = 12.45 min (Chiralpak IE, λ = 215 nm, 35% *i*-PrOH/hexane, flow rate = 1.0 mL/min).

(3aS,7aS)-7a-Phenethyl-3-vinylidene-3a,7a-dihydrobenzofuran-2,5(3H,4H)-dione (**2g**)

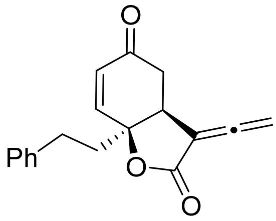

Colorless oil, $[\alpha]^{25}_D$ = +31 (c 1.0, EtOAc); ^{1}H NMR (500 MHz, $CDCl_3$) δ 7.31 (t, J = 7.4 Hz, 2H), 7.25 – 7.16 (m, 3H), 6.67 (dd, J = 10.4, 1.8 Hz, 1H), 6.10 (d, J = 10.4 Hz, 1H), 5.66 – 5.39 (m, 2H), 3.70 – 3.58 (m, 1H), 2.72 (d, J = 4.2 Hz, 2H), 2.34 (ddd, J = 14.4, 11.2, 6.0 Hz, 1H), 2.24 (ddd, J = 14.4, 11.2, 5.8 Hz, 1H); ^{13}C NMR (125 MHz, $CDCl_3$) δ 208.4, 194.5, 167.3, 145.3, 140.1, 129.8, 128.8, 128.2, 126.6, 98.8, 85.1, 82.5, 42.2, 39.3, 36.2, 29.8; HRMS (ESI) m/z calcd for $C_{18}H_{15}O_3$ $[M-H]^-$ = 279.1027, found = 279.1033. The ee value was 94%, t_R (minor) = 13.03 min, t_R (major) = 14.18 min (Chiralpak IE, λ = 215 nm, 35% *i*-PrOH/hexane, flow rate = 1.0 mL/min).

Methyl 3-((3aS,7aS)-2,5-dioxo-3-vinylidene-3,3a,4,5-tetrahydrobenzofuran-7a(2H)-yl)propanoate (**2h**)

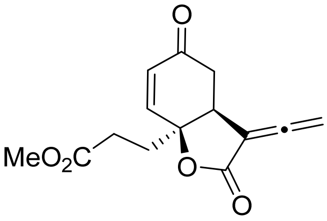

Colorless oil, $[\alpha]^{25}_D$ = +72 (c 1.0, EtOAc); ^{1}H NMR (500 MHz, $CDCl_3$) δ 6.57 (dd, J = 10.4, 1.8 Hz, 1H), 6.07 (d, J = 10.4 Hz, 1H), 5.49 (d, J = 6.2 Hz, 2H), 3.69 (s, 3H), 3.63 – 3.51 (m, 1H), 2.79 – 2.66 (m, 2H), 2.63 – 2.51 (m, 2H), 2.41 – 2.23 (m, 2H); ^{13}C NMR (125 MHz, $CDCl_3$) δ 208.4, 194.3, 172.6, 166.9, 144.7, 130.0, 98.6, 85.2, 81.8, 52.1, 42.0, 35.9, 31.9, 28.1; HRMS (ESI) m/z calcd for $C_{14}H_{14}NaO_5$ $[M+Na]^+$ = 285.0733, found = 285.0742. The ee value was 96%, t_R (minor) = 29.87 min, t_R (major) = 37.44 min (Chiralpak IE, λ = 215 nm, 35% *i*-PrOH/hexane, flow rate = 1.0 mL/min).

(3aS,7aS)-7a-Phenyl-3-vinylidene-3a,7a-dihydrobenzofuran-2,5(3H,4H)-dione (**2i**)

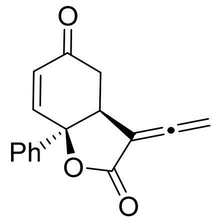

White solid, $[\alpha]^{25}_{D}$ = -14 (c 0.7, EtOAc); ^{1}H NMR (500 MHz, $CDCl_3$) δ 7.56 – 7.37 (m, 5H), 6.73 (dd, *J* = 10.3, 1.8 Hz, 1H), 6.29 (d, *J* = 10.3 Hz, 1H), 5.77 – 5.32 (m, 2H), 3.79 – 3.54 (m, 1H), 2.81 (dd, *J* = 17.3, 5.5 Hz, 1H), 2.73 (dd, *J* = 17.3, 2.6 Hz, 1H); ^{13}C NMR (125 MHz, $CDCl_3$) δ 208.2, 194.6, 167.1, 144.5, 137. 8, 130.1, 129.3, 129.3, 124.9, 98.8, 85.2, 83.6, 46.1, 35.5; HRMS (ESI) m/z calcd for $C_{16}H_{11}O_3$ $[M-H]^-$ = 251.0714, found = 251.0711. The ee value was 96%, t_R (minor) = 15.34 min, t_R (major) = 19.15 min (Chiralpak IE, λ = 215 nm, 35% *i*-PrOH/hexane, flow rate = 1.0 mL/min).

4-((3aS,7aS)-2,5-Dioxo-3-vinylidene-3,3a,4,5-tetrahydrobenzofuran-7a(2H)-yl)benzonitrile (**2j**)

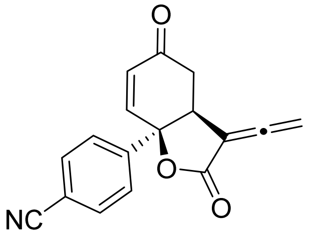

Pale yellow solid, $[\alpha]^{25}_{D}$ = -41 (c 1.0, EtOAc); ^{1}H NMR (500 MHz, $CDCl_3$) δ 7.76 (d, *J* = 8.3 Hz, 2H), 7.62 (d, *J* = 8.4 Hz, 2H), 6.67 (dd, *J* = 10.3, 1.7 Hz, 1H), 6.32 (d, *J* = 10.3 Hz, 1H), 5.70 – 5.41 (m, 2H), 3.82 – 3.55 (m, 1H), 2.98 – 2.50 (m, 2H); ^{13}C NMR (125 MHz, $CDCl_3$) δ 208.4, 193.8, 166.4, 143.1, 142.9, 133.1, 130.9, 125.9, 117.9, 113.4, 98.1, 85.6, 82.7, 45.7, 35.4; RMS (ESI) m/z calcd for $C_{17}H_{10}NO_3$ $[M-H]^-$ = 276.0666, found = 276.0658. The ee value was 96%, t_R (minor) = 23.33 min, t_R (major) = 46.17 min (Chiralpak IE, λ = 215 nm, 35% *i*-PrOH/hexane, flow rate = 1.0 mL/min).

(3aS,7aS)-7a-(3-Fluorophenyl)-3-vinylidene-3a,7a-dihydrobenzofuran-2,5(3H,4H)-dione (**2k**)

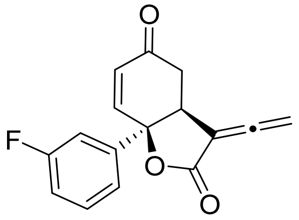

Pale yellow solid, $[\alpha]^{25}_{D}$ = -8 (c 1.0, EtOAc);^{1}H NMR (500 MHz, $CDCl_3$) δ 7.43 (td, *J* = 8.0, 5.9 Hz, 1H), 7.25 – 7.18 (m, 2H), 7.12 (td, *J* = 8.3, 2.5 Hz, 1H), 6.69 (dd, *J* = 10.3, 1.7 Hz, 1H), 6.29 (d, *J* = 10.3 Hz, 1H), 5.63 – 5.44 (m, 2H), 3.79 – 3.59 (m, 1H), 2.80 (dd, *J* = 17.4, 5.4 Hz, 1H), 2.73 (dd, *J* = 17.4, 2.7 Hz, 1H); RMS (ESI) m/z calcd for $C_{16}H_{10}FO_3$ $[M-H]^-$ = 269.0619, found = 269.0630; The ee value was 96%, t_R (minor) = 12.6 min, t_R (major) = 30.54 min (Chiralpak IE, λ = 215 nm, 35% *i*-PrOH/hexane, flow rate = 1.0 mL/min).

(3aS,7aS)-7a-(4-Chlorophenyl)-3-vinylidene-3a,7a-dihydrobenzofuran-2,5(3H,4H)-dione (**2l**)

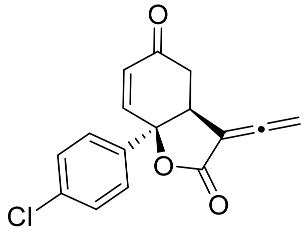

Pale yellow solid, $[\alpha]^{25}_D$ = -27 (c 1.0, EtOAc);^{1}H NMR (500 MHz, $CDCl_3$) δ 7.49 – 7.36 (m, 4H), 6.69 (dd, *J* = 10.3, 1.7 Hz, 1H), 6.29 (d, *J* = 10.3 Hz, 1H), 5.55 (dd, *J* = 13.7, 4.8 Hz, 1H), 5.52 (dd, *J* = 13.6, 4.8 Hz, 1H), 3.72 – 3.57 (m, 1H), 2.77 (dd, *J* = 17.4, 5.2 Hz, 1H), 2.72 (dd, *J* = 17.4, 2.9 Hz, 1H); ^{13}C NMR (125 MHz, $CDCl_3$) δ 208.2, 194.2, 166.8, 143.9, 136.4, 135.5, 130.4, 129.5, 126.5, 98.58, 85.3, 83.0, 46.0, 35.4; RMS (ESI) m/z calcd for $C_{16}H_{10}ClO_3$ $[M-H]^-$ = 285.0324, found = 285.0317; The ee value was 96%, t_R (minor) = 12.19 min, t_R (major) = 18.90 min (Chiralpak IE, λ = 215 nm, 35% *i*-PrOH/hexane, flow rate = 1.0 mL/min).

(3aS,7aS)-7a-(o-Tolyl)-3-vinylidene-3a,7a-dihydrobenzofuran-2,5(3H,4H)-dione (**2m**)

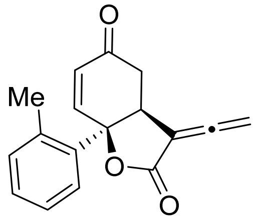

Colorless oil, $[\alpha]^{25}_D$ = +92 (c 1.0, EtOAc);^{1}H NMR (500 MHz, $CDCl_3$) δ 7.37 – 7.13 (m, 4H), 6.82 (dd, *J* = 10.3, 1.8 Hz, 1H), 6.29 (d, *J* = 10.3 Hz, 1H), 5.60 – 5.44 (m, 2H), 4.03 – 3.90 (m, 1H), 2.76 – 2.60 (m, 2H), 2.50 (s, 3H); ^{13}C NMR (125 MHz, $CDCl_3$) δ 208.5, 194.9, 167.3, 145.0, 136.6, 134. 5, 133.7, 129.7, 129.6, 127.0, 126.2, 98.4, 85.2, 85.1, 43.7, 35.8, 21.8; RMS (ESI) m/z calcd for $C_{17}H_{13}O_3$ $[M-H]^-$ = 265.0870, found = 265.0877; The ee value was 90%, t_R (major) = 10.61 min, t_R (minor) = 12.01 min (Chiralpak IE, λ = 215 nm, 35% *i*-PrOH/hexane, flow rate = 1.0 mL/min).

(3aS,7aS)-7a-(Naphthalen-2-yl)-3-vinylidene-3a,7a-dihydrobenzofuran-2,5(3H,4H)-dione (**2n**)

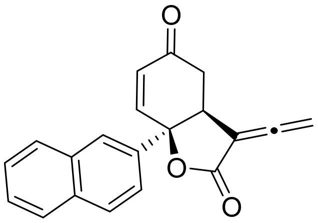

Pale yellow solid, $[\alpha]^{25}_D$ = -83 (c 1.0, EtOAc); ^{1}H NMR (500 MHz, $CDCl_3$) δ 7.95 (d, *J* = 8.6 Hz, 1H), 7.91 (s, 1H), 7.87 (ddd, *J* = 10.2, 6.8, 3.5 Hz, 2H), 6.82 (dd, *J* = 10.3, 1.8 Hz, 1H), 6.36 (d, *J* = 10.3 Hz, 1H), 5.66 – 5.44 (m, 2H), 3.94 – 3.67 (m, 1H), 2.85 (dd, *J* = 17.3, 5.5 Hz, 1H), 2.79 – 2.69 (m, 1H); ^{13}C NMR (125 MHz, $CDCl_3$) δ 208.3, 194.7, 167.2, 144.4, 135.0, 133.4, 132.9, 130.4, 129.5, 128.3, 127.8, 127.2, 127.1, 124.6, 122.1, 98.8, 85.2, 83.8, 46.0, 35.6; RMS (ESI) m/z calcd for $C_{20}H_{13}O_3$ $[M-H]^-$ = 301.0870, found = 301.0879; The ee value was 94%, t_R (minor) = 18.13 min, t_R (major) = 48.56 min (Chiralpak IE, λ = 215 nm, 35% *i*-PrOH/hexane, flow rate = 1.0 mL/min).

(3aS,7aS)-7a-Ethynyl-3-vinylidene-3a,7a-dihydrobenzofuran-2,5(3H,4H)-dione (**2o**)

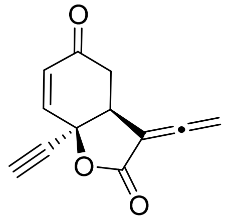

white solid, $[\alpha]^{25}_{D}$ = +4 (c 1.0, EtOAc); ^{1}H NMR (500 MHz, $CDCl_3$) δ 6.79 – 6.54 (m, 1H), 6.10 (d, *J* = 10.2 Hz, 1H), 5.54 (d, *J* = 6.1 Hz, 2H), 3.96 (ddt, *J* = 8.0, 5.6, 2.7 Hz, 1H), 2.92 (dd, *J* = 17.0, 6.0 Hz, 1H), 2.90 (s, 1H), 2.75 (dd, *J* = 17.0, 2.8 Hz, 1H); ^{13}C NMR (125 MHz, $CDCl_3$) δ 208.4, 193.6, 165. 8, 141.8, 129.2, 96.9, 85.5, 78.5, 77.9, 73.7, 44.5, 35.6; RMS (ESI) m/z calcd for $C_{12}H_7O_3$ $[M-H]^-$ = 199.0401, found = 199.0410; The ee value was 97%, t_R (minor) = 11.35 min, t_R (major) = 12.13 min (Chiralpak IE, λ = 215 nm, 35% *i*-PrOH/hexane, flow rate = 1.0 mL/min).

(3aS,7aS)-3a,7,7a-Trimethyl-3-vinylidene-3a,7a-dihydrobenzofuran-2,5(3H,4H)-dione (**2p**)

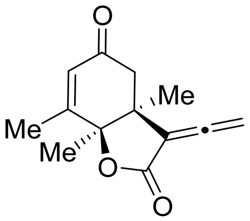

white solid, $[\alpha]^{25}_{D}$ = +113 (c 1.0, EtOAc, 96% ee); ^{1}H NMR (500 MHz, $CDCl_3$) δ 5.90 (d, *J* = 0.7 Hz, 1H), 5.64 – 5.27 (m, 2H), 2.64 (d, *J* = 17.0 Hz, 1H), 2.46 (d, *J* = 17.0 Hz, 1H), 2.04 (s, 3H), 1.58 (s, 3H), 1.31 (s, 3H); ^{13}C NMR (125 MHz, $CDCl_3$) δ 206.8, 194.6, 167.4, 158.1, 127.8, 103.9, 85. 5, 85.2, 47.0, 43.3, 29.7, 24.8, 18.6, 17.8; RMS (ESI) m/z calcd for $C_{13}H_{13}O_3$ $[M-H]^-$ = 217.0870, found = 217.0880; The ee value was 67%, t_R (major) = 8.60 min, t_R (minor) = 9.82 min (Chiralpak IE, λ = 215 nm, 35% *i*-PrOH/hexane, flow rate = 1.0 mL/min), after simple recrystallization the ee value was increased to 96% with 60% yield.

Representative procedure of dynamic kinetic resolution of allenes via RC reaction

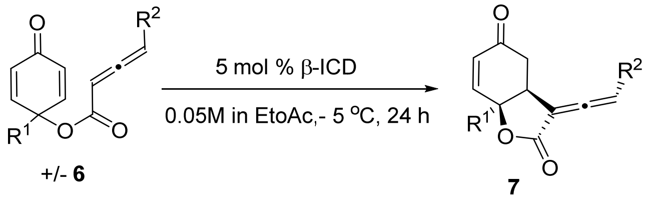

To a flame dried round bottle flask with a magnetic stirring bar at -5 °C under N_2 were added allenoate **6** (0.15 mmol) and EtOAc (3 mL), followed by the addition of β-ICD (5 mol %, 2.3 mg). The resulting mixture was stirred for 24 h. The solvent was removed under reduced pressure and the residue was purified by column chromatography on silica gel to afford annulation adduct **7**.

Analytical data products 7

(3aS,7aS)-7a-Methyl-3-((S)-prop-1-en-1-ylidene)-3a,7a-dihydrobenzofuran-2,5(3H,4H)-dione (**7a**)

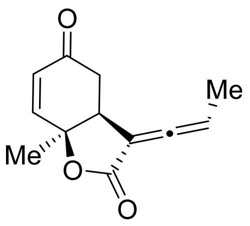

From crude ^{1}H NMR, the d.r. value is 5.5:1; and the unseparated isomers were isolated in 90% yield as a white solid; ^{1}H NMR (500 MHz, $CDCl_3$) δ 6.57 (dt, *J* = 10.3, 1.6 Hz, 1H), 6.00 (d, *J* = 10.1 Hz, 1H), 5.90 – 5.83 (m, 1H), 3.56 – 3.39 (m, 1H), 2.77 – 2.56 (m, 2H), 1.81 (d, *J* = 7.4 Hz, 3H), 1.71 (s, 3H); ^{13}C NMR (125 MHz, $CDCl_3$) the major isomer: δ 205.4, 194.8, 167. 8, 146.5, 128.9, 98.5, 96.5, 80.4, 44.0, 36.0, 24.0, 13.0; the minor isomer: δ 205. 6, 194.8, 167.2, 146.4, 128.9, 98.4, 80.6, 44.8, 36.3, 23.8, 13.4; RMS (ESI) m/z calcd for $C_{12}H_{11}O_3$ $[M-H]^-$ = 203.0714, found = 203.0722; The ee value of major isomer was 99%, t_R (major) = 10.34 min, t_R (minor) = 12.35 min; the ee value of minor isomer was 91%, t_R (major) = 9.66 min, t_R (minor) = 11.56 min; (Chiralpak IE, λ = 215 nm, 35% *i*-PrOH/hexane, flow rate = 1.0 mL/min).

(3aS,7aS)-3-((S)-But-1-en-1-ylidene)-7a-methyl-3a,7a-dihydrobenzofuran-2,5(3H,4H)-dione (**7b**)

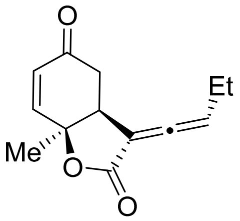

From crude ^{1}H NMR, the d.r. value is 7:1; the two isomers were isolated in 92% yield and the major isomer could be separated by simple column chromatography in 75% yield as a white solid, $[\alpha]^{25}_D$ = +70 (c 1.0, Ea); ^{1}H NMR (500 MHz, $CDCl_3$) δ 6.57 (dd, *J* = 10.3, 1.8 Hz, 1H), 6.00 (d, *J* = 10.3 Hz, 1H), 5.96 (dd, *J* = 12.1, 6.4 Hz, 1H), 3.56 – 3.38 (m, 1H), 2.90 – 2.50 (m, 2H), 2.35 – 2.00 (m, 2H), 1.71 (s, 3H), 1.06 (t, *J* = 7.4 Hz, 3H); ^{13}C NMR (125 MHz, $CDCl_3$) δ 204.7, 194.7, 167.7, 146.5, 128.8, 103.5, 99.6, 80.3, 44.1, 36.0, 24.0, 21.2, 13.1; RMS (ESI) m/z calcd for $C_{13}H_{13}O_3$ $[M-H]^-$ = 217.0870, found = 217.0865; The ee value of the major isomer was 99.5%, t_R (minor) = 19.65 min, t_R (major) = 29.98 min; The ee value of the minor isomer was 98%, t_R (minor) = 21.46 min, t_R (major) = 24.94 min (Chiralpak IA, λ = 215 nm, 5% *i*-PrOH/hexane, flow rate = 0.75 mL/min).

(3aS,7aS)-7a-Methyl-3-((S)-3-methylbut-1-en-1-ylidene)-3a,7a-dihydrobenzofuran-2,5(3H,4H)-dione (**7c**)

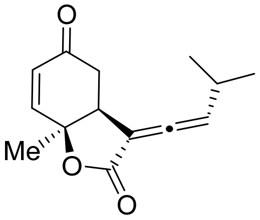

From crude ^{1}H NMR, the d.r. value is > 19:1; the product was isolated in 93% yield as a white solid, $[\alpha]^{25}_D$ = +69 (c 0.6, Ea); ^{1}H NMR (500 MHz, $CDCl_3$) δ 6.58 (dd, *J* = 10.3, 1.8 Hz, 1H), 6.00 (d, *J* = 10.4 Hz, 1H), 5.93 (t, *J* = 5.8 Hz, 1H), 3.54 – 3.41 (m, 1H), 2.76 – 2.63 (m, 2H), 2.52 (dq, *J* = 13.3, 6.7 Hz, 1H), 1.71 (s, 3H), 1.08 (dd, *J* = 6.8, 1.4 Hz, 6H); ^{13}C NMR (125 MHz, $CDCl_3$) δ 203.8, 194.7, 167.7, 146.6, 128.8, 108.9,

100.00, 80.3, 44.1, 35.9, 28.3, 24.0, 22.4, 22.2; RMS (ESI) m/z calcd for $C_{14}H_{15}O_3$ $[M-H]^-$ = 217.0870, found = 217.0865; The ee value was 98%, t_R (major) = 7.60 min, t_R (minor) = 9.23 min (Chiralpak IE, λ = 215 nm, 35% *i*-PrOH/hexane, flow rate = 1.0 mL/min).

(3aS,7aS)-3-((S)-Hex-1-en-1-ylidene)-7a-methyl-3a,7a-dihydrobenzofuran-2,5(3H,4H)-dione (**7d**)

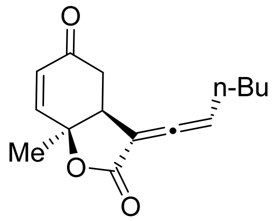

From crude ^{1}H NMR, the d.r. value is 10:1; the two isomers were isolated in 91% yield and the major isomer could be separated by simple column chromatography in 80% yield as a white solid, $[\alpha]^{25}_D$ = +43 (c 0.8, Ea); ^{1}H NMR (500 MHz, $CDCl_3$) δ 6.58 (dd, *J* = 10.3, 1.8 Hz, 1H), 6.01 (d, *J* = 10.3 Hz, 1H), 5.91 (dd, *J* = 12.6, 6.9 Hz, 1H), 3.60 – 3.29 (m, 1H), 2.79 – 2.61 (m, 2H), 2.17 (dd, *J* = 14.3, 7.1 Hz, 2H), 1.72 (s, 3H), 1.53 – 1.39 (m, 2H), 1.39 – 1.34 (m, 2H), 0.90 (t, *J* = 7.2 Hz, 3H); ^{13}C NMR (125 MHz, $CDCl_3$) δ 204.9, 194.7, 167.8, 146.6, 128.8, 101.7, 98.9, 80.3, 44.1, 36.0, 30.7, 27.4, 24.0, 22.0, 13.7; RMS (ESI) m/z calcd for $C_{15}H_{17}O_3$ $[M-H]^-$ = 245.1183, found = 245.1192; The ee value was 97%, t_R (major) = 8.15 min, t_R (minor) = 10.31 min (Chiralpak IE, λ = 215 nm, 35% *i*-PrOH/hexane, flow rate = 1.0 mL/min).

(3aS,7aS)-7a-Methyl-3-((S)-4-phenylbut-1-en-1-ylidene)-3a,7a-dihydrobenzofuran-2,5(3H,4H)-dione (**7e**)

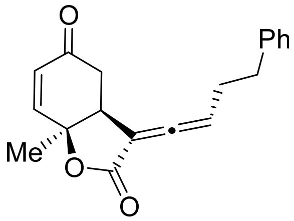

From crude ^{1}H NMR, the d.r. value is 16:1; and the major isomer could be separated by simple column chromatography in 90% yield as a white solid, $[\alpha]^{25}_D$ = +41 (c 1.0, Ea); ^{1}H NMR (500 MHz, $CDCl_3$) δ 7.32 – 7.27 (m, 2H), 7.20 (t, *J* = 6.7 Hz, 3H), 6.59 (dd, *J* = 10.3, 1.7 Hz, 1H), 6.04 (d, *J* = 10.3 Hz, 1H), 5.93 (dd, *J* = 12.9, 6.5 Hz, 1H), 3.49 (td, *J* = 5.3, 2.3 Hz, 1H), 2.76 – 2.63 (m, 4H), 2.42 – 2.34 (m, 2H), 1.72 (s, 3H); ^{13}C NMR (125 MHz, $CDCl_3$) δ 204.8, 194.8, 167.7, 146. 6, 140.6, 128.9, 128.5, 128.5, 126.2, 101.1, 99.4, 80.5, 44.7, 36.2, 34.9, 29.8, 23.9; RMS (ESI) m/z calcd for $C_{19}H_{17}O_3$ $[M-H]^-$ = 293.1183, found = 293.1188; The ee value was 98%, t_R (major) = 11.07 min, t_R (minor) = 13.12 min (Chiralpak IE, λ = 215 nm, 35% *i*-PrOH/hexane, flow rate = 1.0 mL/min).

(3aS,7aS)-3-((S)-2-Cyclohexylvinylidene)-7a-methyl-3a,7a-dihydrobenzofuran-2,5(3H,4H)-dione (**7f**)

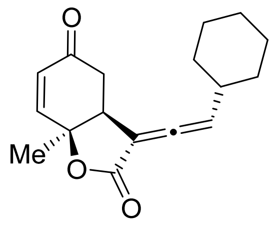

From crude ^{1}H NMR, the d.r. value is > 19:1; the product was isolated in 85% yield as a pale yellow solid, $[\alpha]^{25}_D$ = +29 (c 1.0, Ea); ^{1}H NMR (500 MHz, $CDCl_3$) δ 6.56 (dd, *J* = 10.3, 1.6 Hz, 1H), 5.98 (d, *J* = 10.3 Hz, 1H), 5.88 (t, *J* = 5.8 Hz, 1H), 3.47 (dt, *J* = 8.0, 2.5 Hz, 2H), 2.74 – 2.61 (m, 2H), 2.28 – 2.13 (m, 1H), 1.78 (d, *J* = 12.8 Hz, 2H), 1.74 – 1.66 (m, 6H), 1.61 (d, *J* = 12.4 Hz, 1H), 1.28 (dd, *J* = 24.7, 12.3 Hz, 2H), 1.16 (dt, *J* = 24.8, 7.8 Hz, 2H); ^{13}C NMR (126 MHz, $CDCl_3$) δ 204.3, 194.7, 167.8, 146.6, 128.8, 107.3, 99.7, 80.3, 44.1, 37.1, 36.0, 32.8, 32.7, 25.8, 25.7, 25.7, 24.0; RMS (ESI) m/z calcd for $C_{17}H_{19}O_3$ $[M-H]^-$ = 271.1340, found = 271.1344; The ee value was 98%, t_R (major) = 8.88 min, t_R (minor) = 11.83 min (Chiralpak IE, λ = 215 nm, 35% *i*-PrOH/hexane, flow rate = 1.0 mL/min).

(3aS,7aS)-3-((S)-3-Methylbut-1-en-1-ylidene)-7a-phenyl-3a,7a-dihydrobenzofuran-2,5(3H,4H)-dione **(7h)**

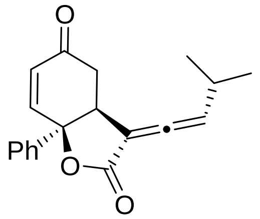

From crude ^{1}H NMR, the d.r. value is 7.5:1; the two isomers were isolated in 91% and the major isomer could be separated by simple column chromatography in 76% yield as a white solid, $[\alpha]^{25}_D$ = -54 (c 1.0, Ea); ^{1}H NMR (500 MHz, $CDCl_3$) δ 7.53 – 7.37 (m, 5H), 6.71 (dd, *J* = 10.3, 1.8 Hz, 1H), 6.25 (d, *J* = 10.3 Hz, 1H), 5.98 (t, *J* = 5.8 Hz, 1H), 3.66 (ddd, *J* = 7.7, 5.5, 2.2 Hz, 1H), 2.79 (dd, *J* = 17.3, 5.6 Hz, 1H), 2.69 (dd, *J* = 17.3, 2.3 Hz, 1H), 2.53 (dq, *J* = 13.3, 6.7 Hz, 1H), 1.08 (d, *J* = 6.8 Hz, 6H); ^{13}C NMR (125 MHz, $CDCl_3$) δ 203.8, 194.8, 167.5, 144.6, 138.1, 130.0, 129.2, 124.9, 109.1, 100.0, 83.3, 46.1, 35.6, 28.3, 22.4, 22.2; RMS (ESI) m/z calcd for $C_{19}H_{17}O_3$ $[M-H]^-$ = 293.1183, found = 293.1173; The ee value was 98%, t_R (minor) = 9.66 min, t_R (major) = 10.19 min (Chiralpak IB, λ = 215 nm, 10% *i*-PrOH/hexane, flow rate = 1.0 mL/min).

Parallel Kinetic Resolution of 8

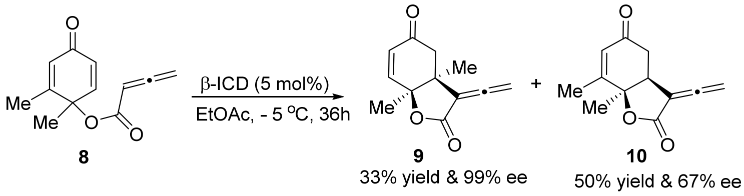

To a flame dried round bottle flask with a magnetic stirring bar at – 5 °C under N_2 were added allenoate **8** (36 mg) and EtOAc (3.6 mL), followed by the addition of β-ICD (5 mol %, 3 mg). The resulting mixture was stirred for 36 h. The solvent was removed under reduced pressure and the residue was purified by column chromatography on silica gel to afford annulation adduct **9** (12 mg, 33% yield) as a white solid and **10** (18 mg, 50% yield) as a white solid. Analytical data of **9**: $[\alpha]^{25}_D$ = +228 (c 0.6, EtOAc); ^{1}H NMR (500 MHz, $CDCl_3$) δ 6.60 (d, *J* = 10.2 Hz, 1H), 6.03 (d, *J* = 10.2 Hz, 1H), 5.58 – 5.37 (m, 2H), 2.67 (d, *J* = 16.8 Hz, 1H), 2.47 (d, *J* = 16.8 Hz, 1H), 1.57 (s, 3H), 1.31 (s, 3H); ^{13}C NMR (125 MHz, $CDCl_3$) δ 207.2, 195.4, 167.2, 147.8, 128.6, 104.0, 85.2, 83.1, 46.2, 43.7, 24.4, 19.2; HRMS (ESI) m/z calcd for $C_{12}H_{11}O_3$ $[M-H]^-$ = 203.0714, found = 203.0726; The ee value was 99%, t_R (major) = 9.13 min, t_R (minor) = 10.46 min (Chiralpak IE, λ = 215 nm, 35% *i*-PrOH/hexane, flow rate = 1.0 mL/min). Analytical data of **10**: $[\alpha]^{25}_D$ = +54 (c 0.9, EtOAc); ^{1}H NMR (500 MHz, $CDCl_3$) δ 5.90 (s, 1H), 5.47 (d, *J* = 6.3 Hz, 1H), 3.59 – 3.46 (m, 1H), 2.71 (d,

J = 3.9 Hz, 2H), 2.03 (s, 3H), 1.73 (s, 3H); ^{13}C NMR (125 MHz, $CDCl_3$) δ 207.9, 194.1, 167.3, 156.9, 127.6, 98.6, 84.9, 82.8, 45.1, 35.5, 22.8, 18.3; HRMS (ESI) m/z calcd for $C_{12}H_{11}O_3$ [M-H]$^-$ = 203.0714, found = 203.0716; The ee value was 67%, t_R (major) = 10.19 min, t_R (minor) = 11.20 min (Chiralpak IE, λ = 215 nm, 35% *i*-PrOH/hexane, flow rate = 1.0 mL/min).

DKR process involving chirally pure 6c-1treated by DABCO

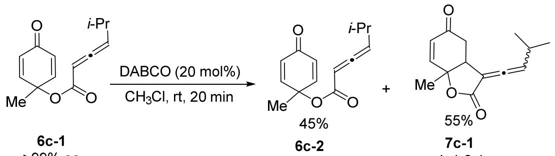

6c-1
>99% ee

6c-2
33% ee

7c-1
dr 1.2:1
ee 0

To a flame dried round bottle flask with a magnetic stirring bar at room temperature under N_2 were added allenoate **6c-1** (0.1 mmol, 23.2 mg) and CH_3Cl (1 mL), followed by the addition of DABCO (20 mol %, 2.2 mg). The resulting mixture was stirred for 20 min quenched by filtered through a short pad of silica gel to afford a mixture. From crude ^{1}H NMR, the ratio of **6c-2** to **7c-1** was 45 : 55. The mixture was purified by column chromatography on silica gel to afford **6c-2** to **7c-1**. The ee value of **6c-2** was 33% and **7c-1** was absolutely racemic with dr value 1.2 : 1.

Preparation of 13 from 2e

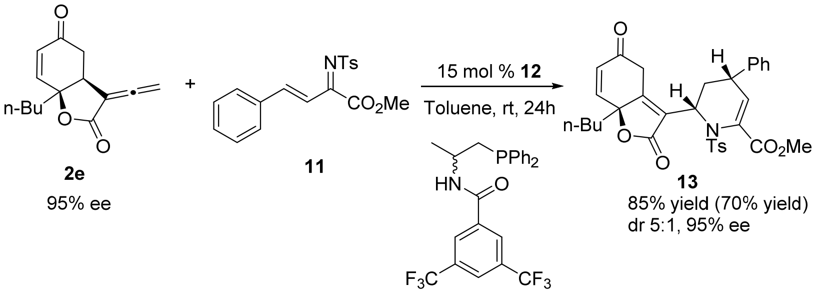

2e
95% ee

11

+/- **12**

13
85% yield (70% yield)
dr 5:1, 95% ee

To the solution of **2e** (0.1 mmol, 23 mg) and imine **11** (0.15 mmol, 51 mg) in toluene (1 mL) at room temperature was added **12** (0.015 mmol, 7.2 mg). The resulting mixture was stirred for 24 h, and the mixture was directly filtered through a short pad of silica gel with hexane/ EtOAc 3:1 as the elute to afford crude **13** (50 mg, 85% yield). From crude ^{1}H NMR, the d.r. value is 5:1. The major isomer was isolated by column chromatography with hexane /acetone 10:1 as the elute in 70% yield as a white solid. The absolute configuration was determined by NOESY spectrum. $[\alpha]^{25}_D$ = +138 (c 1.0, EtOAc); ^{1}H NMR (500 MHz, $CDCl_3$) δ 7.76 (d, J = 8.1 Hz, 2H), 7.35 (d, J = 8.1 Hz, 2H), 7.30 – 7.25 (m, 2H), 7.22 (d, J = 7.1 Hz, 1H), 7.07 (d, J = 10.0 Hz, 1H), 7.00 (d, J = 7.5 Hz, 2H), 6.68 (d, J = 3.1 Hz, 1H), 6.03 (d, J = 9.9 Hz, 1H), 4.67 (dd, J = 10.9, 6.2 Hz, 1H), 4.62 (d, J = 18.6 Hz, 1H), 3.94 (s, 3H), 3.20 (d, J = 18.6 Hz, 1H), 2.67 (dt, J = 10.9, 3.3 Hz, 1H), 2.46 (s, 3H), 2.37 – 2.30 (m, 1H), 2.01 – 1.93 (m, 1H), 1.83 (dd, J = 24.0, 11.1 Hz, 1H), 1.58 – 1.48 (m, 1H), 1.34 – 1.23 (m, 4H), 0.86 (t, J = 6.8 Hz, 3H); ^{13}C NMR (125 MHz, $CDCl_3$) δ 192.6, 170.5, 165.5, 159.0, 146.3, 144.9, 141.3, 133.8, 133.1, 132.5, 130.0, 129.0, 128. 9, 128.3, 127.3, 127.0, 125.7, 84.3, 52.8, 52.0, 39.3, 38.4, 38.1, 38.0, 25.2, 22.5, 21.7, 13.7; HRMS (ESI) m/z calcd for $C_{32}H_{33}NNaO_7S$ [M+Na]$^+$ = 598.1870, found = 598.1881; The ee value was 95%, t_R (major) = 26.77 min, t_R

(minor) = 29.15 min (Chiralpak IE, λ = 215 nm, 35% *i*-PrOH/hexane, flow rate = 1.0 mL/min).

Supplementary References

1. Sheldrick, G. M., University of Göttingen, Germany, 1996.
2. Sheldrick, G. M. A short history of SHELX. *Acta Crystallogr. Sect. A.* **64**, 112–122 (2008).
3. Müller, P., Herbst-Irmer, R., Spek, A. L., Schneider, T. & Sawaya, M. *Crystal Structure Refinement: A Crystallographer's Guide to SHELXL.* Chapter 3, Oxford, UK, Oxford University Press/International Union of Crystallography, 2006.
4. Carreño, M. C., González-López, M. & Urbano, A. Oxidative de-aromatization of para-alkyl phenols into para-peroxyquinols and para-quinols mediated by oxone as a source of Singlet oxygen. *Angew. Chem. In. Ed.* **45**, 2737–2741 (2006).
5. McCarroll, A. J., Bradshaw,T. D., Westwell, A. D., Matthews, C. S. & Stevens, M. F. G. Quinols as novel therapeutic agents. 7.[1] synthesis of antitumor 4-[1-(Arylsulfonyl-1H-indol-2-yl)]-4-hydroxycyclohexa-2,5-dien-1-ones by Sonogashira reactions. *J. Med. Chem.* **50**, 1707–1710 (2007)
6. Stern, A. J. & Swenton, J. S. Addition of organolithium reagents to quinone silyl methyl monoketals. A useful expedient in the synthesis of p-quinols having acid-sensitive groups. *J. Org. Chem*. **53**, 2465–2468 (1988).
7. Yakura, T., Omoto, M., Yamauchi, Y., Tian, Y. & Ozono, A. Hypervalent iodine oxidation of phenol derivatives using a catalytic amount of 4-iodophenoxyacetic acid and Oxone® as a co-oxidant. *Tetrahedron* **66**, 5833–5840 (2010).
8. McKinley, J., Aponick, A., Raber, J. C., Fritz, C., Montgomery, D. & Wigal, C. T. Reactions of alkyllithium and Grignard reagents with benzoquinone: evidence for an electron-transfer mechanism. *J. Org. Chem.* **62***,* 4874–4876 (1997).
9. Kwon, O., Tamanoi, F., Fiji, H. & Watanabe, M. WO 2010014054, 2010
